# Gain- and loss-of-function alleles within signaling pathways lead to phenotypic diversity among individuals

**DOI:** 10.1016/j.isci.2024.110860

**Published:** 2024-08-31

**Authors:** Matthew D. Vandermeulen, Sakshi Khaiwal, Gabriel Rubio, Gianni Liti, Paul J. Cullen

**Affiliations:** 1 Department of Biological Sciences, University at Buffalo, Buffalo, NY 14260-1300, USA; 2 Université Côte d’Azur, CNRS, INSERM, IRCAN, Nice, France

**Keywords:** Biochemistry, Genetics, Molecular biology

## Abstract

Understanding how phenotypic diversity is generated is an important question in biology. We explored phenotypic diversity among wild yeast isolates (*Saccharomyces cerevisiae*) and found variation in the activity of MAPK signaling pathways as a contributing mechanism. To uncover the genetic basis of this mechanism, we identified 1957 SNPs in 62 candidate genes encoding signaling proteins from a MAPK signaling module within a large collection of yeast (>1500 individuals). Follow-up testing identified functionally relevant variants in key signaling proteins. Loss-of-function (LOF) alleles in a PAK kinase impacted protein stability and pathway specificity decreasing filamentous growth and mating phenotypes. In contrast, gain-of-function (GOF) alleles in G-proteins that were hyperactivating induced filamentous growth. Similar amino acid substitutions in G-proteins were identified in metazoans that in some cases were fixed in multicellular lineages including humans, suggesting hyperactivating GOF alleles may play roles in generating phenotypic diversity across eukaryotes. A mucin signaler that regulates MAPK activity was also found to contain a prevalance of presumed GOF alleles amoung individuals based on changes in mucin repeat numbers. Thus, genetic variation in signaling pathways may act as a reservoir for generating phenotypic diversity across eukaryotes.

## Highlights

• MAPK activity varies across wild yeast isolates and generates phenotypic diversity
• LOF alleles in a PAK cause variation in MAPK activity, PAK stability and specificity
• GOF alleles identified in G-proteins prevalently contributed to phenotypic diversity
• Signaling proteins may be a reservoir for generating phenotypic diversity in eukaryotes

## Graphical abstract

**Figure:**
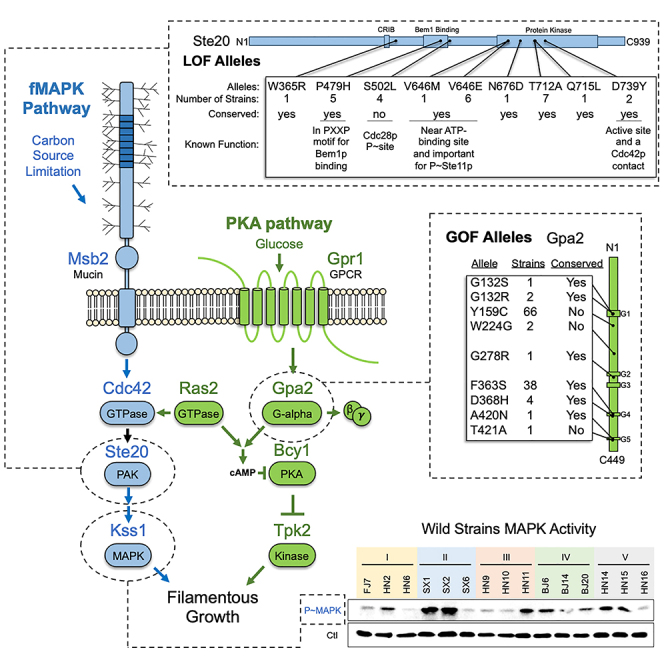

## Introduction

Phenotypic diversity is important in the evolution and stability of biological systems.^1^ A fundamental goal in biology is to understand how phenotypic diversity is generated through differences in genotypes (i.e., genotype-to-phenotype^2^^,^^3^^,^^4^^,^^5^). Genotypic differences arising from loss-of-function (LOF) mutations have been established as a major contributor toward phenotypic diversity.^6^^,^^7^^,^^8^^,^^9^^,^^10^ Dominant gain-of-function (GOF) mutations can also occur.^11^^,^^12^^,^^13^^,^^14^^,^^15^^,^^16^ However, they are more difficult to identify by computational methods^7^^,^^17^^,^^18^ and are often associated with the disease state for certain types of proteins (e.g., G-proteins such as RAS,^19^^,^^20^ tumor suppressor proteins such as p53,^21^^,^^22^^,^^23^ kinases such as ALPK1,^24^ membrane channels such as PIEZO1^25^ and KCNJ6,^26^ and transcription factors such as STAT6^27^).

Advances in understanding genotype-to-phenotype have come from exploring natural variation within populations to link genetic variants with phenotypic differences among individuals. For example, phenotypic differences between individuals can result from genetic variation affecting gene expression^28^^,^^29^^,^^30^^,^^31^^,^^32^ and signaling pathways.^33^^,^^34^^,^^35^^,^^36^^,^^37^ Signaling pathways are of particular interest because they control major processes related to human health. Genetic variation in signaling pathways contributes to congenital diseases,^38^^,^^39^^,^^40^^,^^41^ inherited cancers,^42^^,^^43^ and the severity of symptoms during COVID-19 infections.^44^^,^^45^

Signaling pathways, such as evolutionarily conserved mitogen-activated protein kinase (MAPK) pathways, typically function by sensing environmental cues through receptors or sensors at the plasma membrane. Once activated, receptors control the activity of relay molecules, second messengers, and transcription factors that control gene expression of a subset of the genome.^46^ MAPK pathways regulate many kinds of phenotypes including morphogenesis during development, cell differentiation, and stress responses to the environment.^47^^,^^48^^,^^49^^,^^50^^,^^51^^,^^52^^,^^53^ Moreover, changes in the activity of a MAPK pathway can have dramatic consequences on the resulting phenotype.

Even though much is known about how signaling pathways operate, fundamental questions remain surrounding MAPK pathway activity and function, particularly at the population level. For example, is the activity of MAPK pathways the same between individuals (fixed in a population) or does it vary, and if so, how much? What type of genetic variants (i.e., alleles) occur in MAPK pathway components across a population and do they have similar or different effects on phenotypic diversity? The saprotrophic yeast *Saccharomyces cerevisiae* is a good model to address these questions. For example, a large, growing global collection of more than 1,600 individuals has been isolated and sequenced worldwide from wild and domesticated habitats.^54^^,^^55^^,^^56^^,^^57^

**Figure 1:**
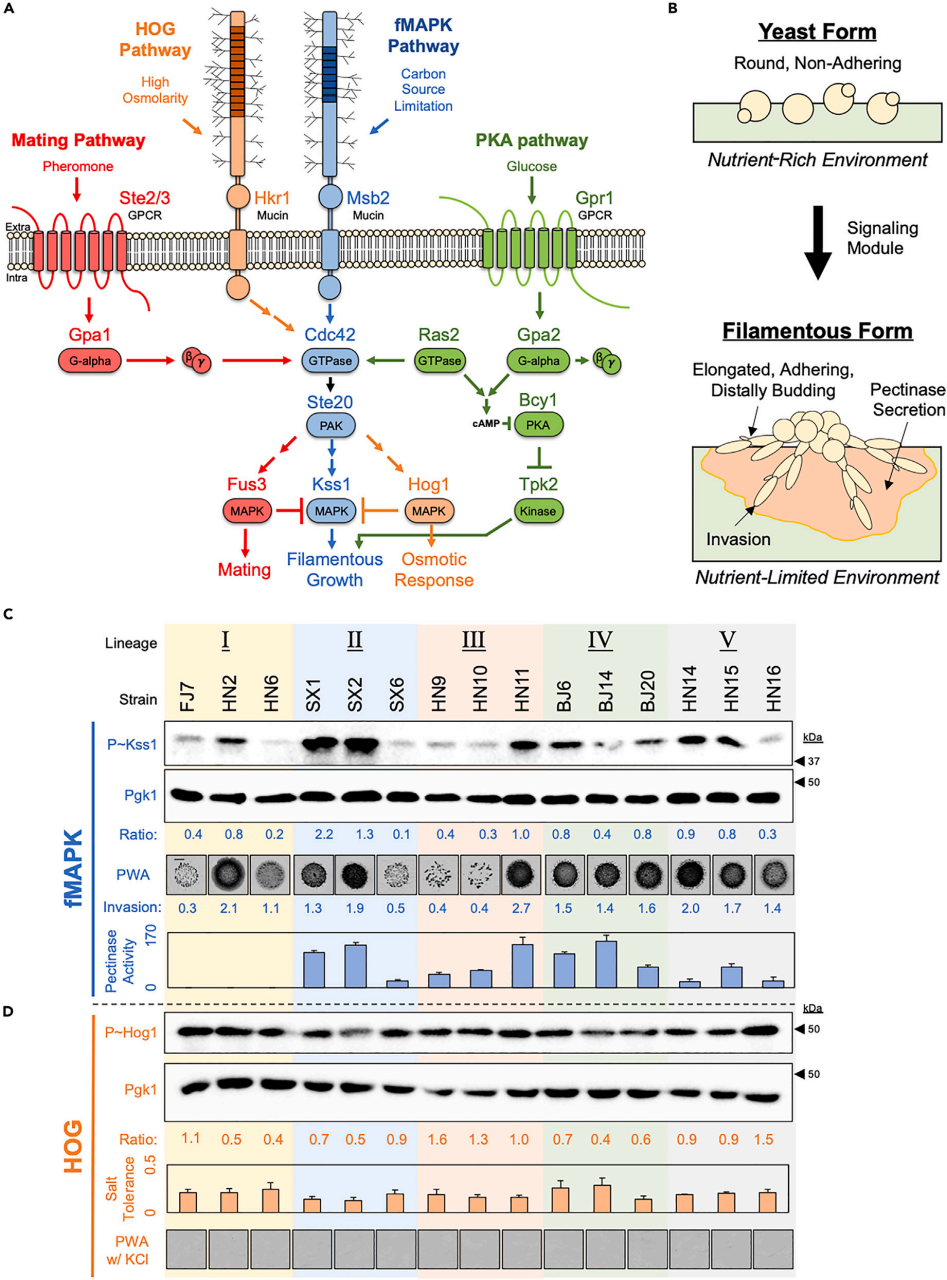
Individuals show variation in MAPK pathway signaling (A) Model of the integrated fMAPK, Mating, HOG, and PKA pathways. Blue, fMAPK or shared MAPK components. Red, Mating components. Orange, HOG components. Green, PKA components. Cdc42p and Ste20p are shared components among the MAPK pathways. Not all components are shown. (B) Model of filamentous growth. Top, yeast-form growth in a nutrient-rich environment. Bottom, filamentous-form growth in a nutrient-limited environment. The filamentous form includes an increase in cell-to-cell adhesion, an increase in cell length, and a switch from axial to distal budding (in haploids). Cells also secret a pectinase enzyme to break down plant material and invade into surfaces. (C) Analysis of wild strains (PC7324-PC7338). Three representative strains were analyzed from five lineages. Immunoblot analysis of fMAPK pathway activity of wild strains grown in 5 mL YPGAL medium for 6 h. Cell extracts were probed with antibodies to detect phosphorylated Kss1p (P ∼ Kss1p) as a readout of fMAPK pathway activity, and Pgk1p as a control for protein levels. Ratio refers to the ratio of P ∼ Kss1p to Pgk1p with HN11 values set to 1. Black arrows, molecular weight markers (kDa). PWA, plate-washing assay after growth of cells on YPGAL medium for 4 days. Inverted images of invasive scars are displayed. Bar = 0.5 cm. Invasion, quantitation of at least five biological replicates (*n* ≥ 5) of the PWA. Pectinase activity, quantitation of the polygalacturnase activity assay of at least three biological replicates (*n* ≥ 3) with error bars representing standard deviation. (D) Immunoblot analysis of HOG pathway activity of wild strains grown in 5 mL YPD+0.5M KCl medium for 5 min. Cell extracts were probed with antibodies to detect phosphorylated Hog1p (P ∼ Hog1p) as a readout of HOG pathway activity, and Pgk1p as a control for protein levels. Ratio, refers to the ratio of P ∼ Hog1p to Pgk1p with HN11 values set to 1. Black arrows, molecular weight markers (kDa). Salt tolerance, growth of cells in YPD+1M KCl medium relative to growth in YPD medium. Three biological replicates (*n* = 3) of the final OD_600_ after 16h were measured, and the average values are reported with error bars representing standard deviation. PWA w/KCl, same as in panel B but cells were grown on YPD + 1M KCl.

We explored the variation in well-established regulatory pathways (Figure 1A) that control an easily quantifiable and complex phenotype called filamentous growth.^58^^,^^59^ Filamentous growth is a conserved phenotype in fungi,^60^ and in many animal and plant pathogens, filamentous growth is critical for virulence.^61^^,^^62^^,^^63^ In yeast, filamentous growth (Figure 1B) is a presumed scavenging response that can be triggered by nutrient limitation. It is characterized by a change in cell morphology, where round cells switch to adhesion-linked “chains” of elongated cells to produce filament-like structures that can invade surfaces (i.e., invasive growth^64^). Additionally, some yeasts secrete pectinase(s) [Pgu1p in *S. cerevisiae*] that break down a major component of plant cell walls (i.e., pectin) during filamentous growth.^65^^,^^66^^,^^67^

Two of the main pathways that regulate filamentous growth are the filamentous growth or fMAPK pathway (Figure 1A, blue^68^^,^^69^^,^^70^) and the cAMP-dependent Protein Kinase A (PKA) pathway (Figure 1A, green^71^^,^^72^^,^^73^^,^^74^). The fMAPK pathway shares components (e.g., Ste20p) with other pathways that control mating [Figure 1A, Mating pathway, red^75^^,^^76^] and the response to high osmolarity [Figure 1A, High Osmolarity Glycerol response (HOG) pathway, orange^77^]. The Mating, HOG, and PKA pathways also impact fMAPK pathway activity in an integrated regulatory module. For example, the Mating and HOG pathways can negatively impact fMAPK pathway activity and filamentous growth,^78^^,^^79^^,^^80^^,^^81^^,^^82^^,^^83^^,^^84^ whereas a component of the PKA pathway (i.e., Ras2p^85^^,^^86^) can positively regulate fMAPK pathway activity (Figure 1A).

Here, we explored variation in MAPK pathway activity across a subset of diverse, wild strains of *S. cerevisiae*. This revealed unexpectedly high levels of variation in MAPK pathway activity among individuals. To identify the genetic basis for signaling variation, we interrogated the integrated regulatory module (Figure 1A) for single nucleotide polymorphisms (SNPs) predicted to be functionally relevant across a global collection of yeast strains using the mutfunc resource.^87^ Unique SNPs (1957 SNPs) were mapped across 62 genes, with a typical individual carrying ≥16 SNPs. Three genes were chosen for follow-up testing, which provided strong validation of the predictions from mutfunc as 54% of the SNPs tested produced a phenotype. Follow-up testing also revealed new LOF and GOF alleles that impact the function, levels, and/or specificity of PAK kinases, G***α***-proteins, and mucin signalers. Similar types of mutations found in yeast G***α***-proteins were identified in populations of multicellular organisms, and in several cases these changes became fixed in metazoans. Overall, the study reveals that genetic variation arising from LOF and GOF alleles in signaling pathways represents one way by which phenotypic diversity is generated.

## Results

### Exploring variation in mitogen-activated protein kinase pathway activity across yeast populations

To compare the activity of MAPK pathways across *S. cerevisiae* strains, wild strains from China/Far East Asia were examined.^88^^,^^89^ These strains were chosen relative to other wild or domesticated strains because their large amount of genetic diversity reflects the ancestral state.^54^^,^^55^^,^^56^ Fifteen strains were examined consisting of three individuals from five separate lineages (Figure S1;^88^^,^^89^).

To examine fMAPK pathway activity, the level of phosphorylated MAP kinase Kss1p was measured under inducing conditions (6h YPGAL^67^^,^^90^). The activity of the fMAPK pathway showed 22-fold variation across individuals (Figure 1C, P ∼ Kss1, full blots in Figure S2), even within lineages (e.g., compare SX2 to SX6). Another MAPK pathway (HOG), which senses osmotic stress, was also examined by measuring the phosphorylation of the MAP kinase Hog1p under inducing conditions for this pathway (5min YPD +0.5M KCl^91^^,^^92^^,^^93^^,^^94^). The HOG pathway also showed variation among individuals (4-fold, Figure 1D, P ∼ Hog1, full blot in Figure S3), although to a lesser degree than the fMAPK pathway. A third MAPK pathway that senses cell wall integrity (P ∼ Slt2p^95^) also showed variation across individuals (7-fold, Figure S4, full blot in Figure S2).

In addition to activated states, MAPK pathways operate in basal/low activity states, which is important for preparing cells to encounter stimuli and facilitating a rapid response.^68^^,^^96^^,^^97^^,^^98^ Basal activity for the HOG pathway also varied across individuals (Figure S5, compared to Figure 1, full blots in Figure S6). These basal patterns did not match the patterns seen in the activated states. The fMAPK pathway basal patterns also varied and did not match the activated states, but detecting variation was difficult due to low levels of basal P ∼ Kss1p (Figure S5). Moreover, basal level activity for both MAPK pathways was lower in all strains, which indicates that the variation in the activity of MAPK pathways is not likely due to changes in basal activity or from mutations that cause constitutive pathway activation. P ∼ Slt2p levels also showed condition-dependent changes across strains (Figure S5). Thus, diverse populations of yeast strains show variation in the activity of MAPK pathways. This variation may arise due to selection of advantageous traits in different environments or from genetic drift due to reproductive isolation.

The fMAPK pathway regulates filamentous growth – a phenotype that is known to vary across strain backgrounds.^99^^,^^100^^,^^101^^,^^102^^,^^103^^,^^104^^,^^105^^,^^106^ To test if variation in filamentous growth corresponds to variation in fMAPK pathway activity, three key aspects of filamentous growth were examined across the wild strains: invasive growth, pectinase activity, and cell elongation (Figure 1B). The wild strains varied in invasive growth (Figure 1C, plate-washing assay (PWA), before washing images in Figure S7). The wild strains also varied for pectinase activity (Figure 1C, pectinase activity, images in Figure S8A, lineage I shows pectinase activity at a later timepoint in Figure S8B) and cell elongation (Figure S9). The phenotypic variation was even more evident when exploring additional environments (i.e., genotype-environment interactions, Figures S7–S9). Filamentous growth mostly corresponded to fMAPK pathway activity (Figure S10), especially within lineages (Figure 1C, e.g., for P ∼ Kss1p, invasive growth, pectinase activity: SX1 and SX2 are higher than SX6; HN9 and HN10 are less than HN11). Any differences between filamentous growth and fMAPK pathway activity may be because filamentous growth is regulated by multiple pathways.^58^^,^^107^^,^^108^^,^^109^ Thus, the high amount of variation in the fMAPK pathway activity results in a high amount of phenotypic variation in filamentous growth.

HOG-dependent phenotypes, such as the ability to grow in high-osmolarity media, also varied but at a lower level compared to fMAPK-dependent phenotypes. For example, when comparing the growth of wild strains in rich medium containing salt (0.5M KCl) relative to rich medium alone, the wild strains showed a 2-fold range in growth [Figure 1D, salt tolerance, other concentrations (1M KCl) and osmolytes (1M sorbitol and 1M glucose) were tested, see Figure S11]. Therefore, compared to the fMAPK pathway, lower levels of variation in HOG pathway activity correspond to lower phenotypic variation in HOG-dependent phenotypes. The HOG pathway may vary less because it plays an essential role in surviving high-osmolarity stress. High-osmolarity media also inhibits invasive growth.^107^^,^^110^^,^^111^ All of the wild strains tested grew on a rich medium containing 1M KCl (Figure S7), but did not show invasive growth (Figure 1D, PWA w/KCl). Collectively, this section shows that the variation in signaling pathway activity and effector phenotypes represent one way for phenotypic diversity to be generated across individuals.

### Identification of impactful single nucleotide polymorphisms in a signaling module that regulates filamentous growth

**Figure 2:**
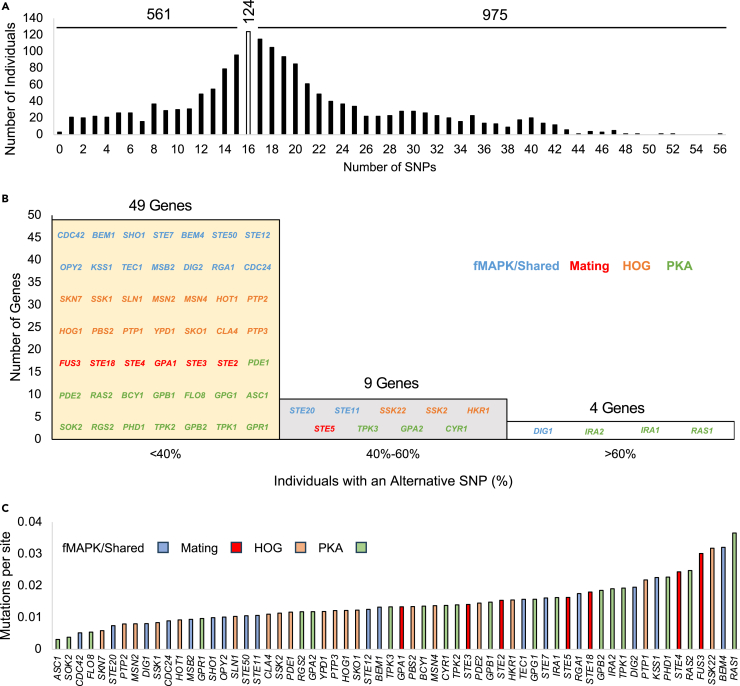
An integrated signaling module harbors many SNPs across yeast strains (A) Histogram displaying the number of individuals (out of 1660) that carry a specified number of identified SNPs. White bar, mode, 124. 561 individuals are less than the mode. 975 individuals are greater than the mode. (B) Histogram displaying the percentage of individuals that carry an alternative SNP in a specified number of signaling genes. Gene names are displayed within a bar graph. Blue, fMAPK/shared; red, Mating; orange, HOG; green, PKA. (C) Bar graph displaying the mutations per site ratio for 62 signaling genes. Blue, fMAPK/shared; red, Mating; orange, HOG; green, PKA.

Based on the immunoblot analysis, the variation in MAPK pathway activity likely did not result from changes in the MAPKs themselves or their ability to become phosphorylated (phosphosites). Thus, we infer that the variation may result from changes in proteins comprising the signaling module that regulates protein kinase activity. To identify the basis of signaling pathway variation, a candidate gene approach was used to identify functionally relevant single nucleotide polymorphisms (SNPs) across 1660 wild and domesticated strains of *S. cerevisiae*.^54^^,^^57^ Genes in a regulatory module that controls filamentous growth (Figure 1A) were tested using the mutfunc resource^87^ [62 genes total, all genes tested and their pathway models in Figure S12A]. A total of 1957 impactful SNPs (i.e., SNPs that putatively affect protein function and/or stability) were identified in open reading frames and were mapped onto proteins including characterized domains (File S1). Overlaying alleles onto this protein map highlighted that a wide range of genetic variation is distributed across proteins. Most individuals carried impactful SNPs, with a typical individual harboring 16 SNPs or more (Figure 2A, white bar, mode at 16). Multiple SNPs in the same strain were commonly associated with different genes rather than occurring in the same gene (File S2), suggesting that multiple alleles could be additive and function to amplify pathway activity. Alternatively, multiple alleles could lead to compensatory effects that modulate pathway activity.

Some genes showed more variation across the collection than others. For example, <40% of individuals carried an alternative SNP in 49 genes [Figure 2B, yellow bar, specific percentages in Figure S12B], whereas 40%–60% of individuals carried an alternative SNP in 9 genes (Figure 2B, gray bar). For example, 729 (44%) and 940 (57%) individuals carry at least one mutation in either the p21-activated kinase (PAK) *STE20* or G***α***-protein *GPA2*, respectively (Figure S12B). Four genes that had >60% of individuals carrying an alternative SNP may reflect an uncommon SNP in the reference strain S288c (Figure 2B, white bar).

The number of SNPs in each gene ranged from 3 to 183 (Figure S12B). To adjust for gene size, the number of SNPs relative to gene length was calculated (i.e., mutations per site). By this method, some genes showed a higher SNP frequency (Figure 2C). For example, the essential GTPase *CDC42* showed a few SNPs (3 SNPs, 0.005 mutations per site), whereas *RAS1,* a nonessential and redundant GTPase, showed more SNPs (35 SNPs, 0.037 mutations per site) [∼7.4-fold difference, Figure 2C]. Impactful SNPs thus occurred at a higher frequency in some signaling genes compared to others.

How the above signaling genes compare to the broader genomic background for signals of possible selection (i.e., dN/dS) was also investigated; however, evidence for positive or balancing selection on signaling genes was not found (Figure S13). Collectively, this data revealed extensive standing genetic variation within signaling pathways. Furthermore, a protein-based map (File S1) of standing genetic variation in a signaling module provides a resource for understanding the range of phenotypic variation found in nature.

### Loss-of-function alleles in a p21-activated kinase show graded effects on mitogen-activated protein kinase pathway activity by impacting protein stability and pathway specificity

**Figure 3:**
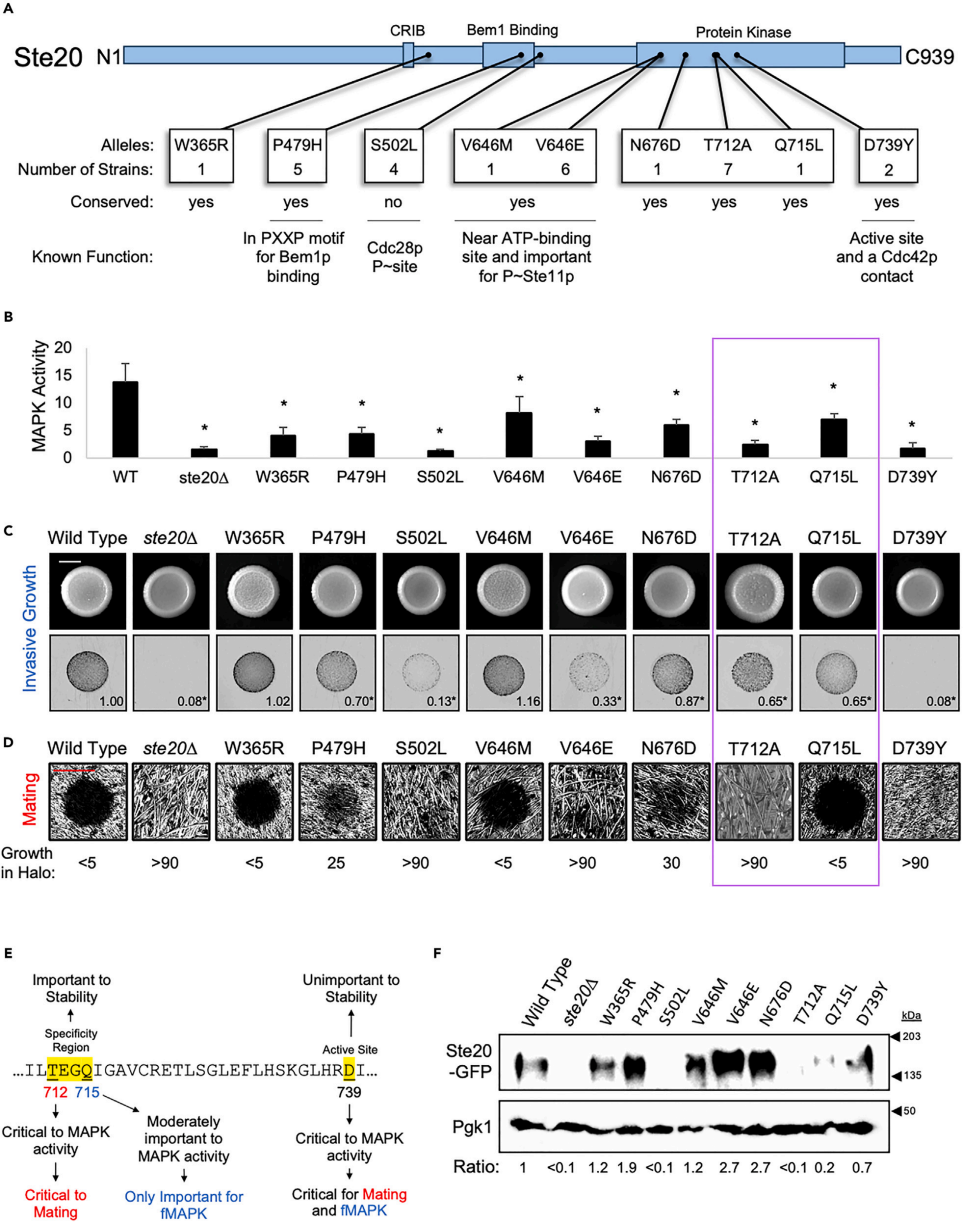
SNPs within a PAK kinase showed graded LOF effects on downstream effector phenotypes Analysis of SNPs generated by site-directed mutagenesis in p*STE20-GFP*. (A) Protein map of Ste20p showing a subset of substitutions found to cause a phenotypic effect. Number of strains represents the number of individuals out of 1660 that carry the amino acid change. Conserved, yes, represent sites that are found conserved in a human PAK kinase (e.g., PAK1, STK3, or STK4). Function, site D739 is the conserved PAK kinase active site of the kinase domain and important for making extended contacts with Cdc42p beyond the CRIB domain^119^; site P479 lies within a PXXP motif that has been shown to interact with the SH3 domain of the polarity scaffold Bem1p^120^; site V646 is a residue near the ATP-binding site (649) that is also important for the phosphorylation of Ste11p^121^; site S502 is a phosphorylation site of the cyclin-dependent kinase Cdc28p.^122^ (B) β-galactosidase assays measuring MAPK activity. Cells of the *ste20*Δ mutant (PC673) were grown harboring p*STE20-GFP* with no change (WT), harboring p*STE20-GFP* with the indicated amino acid change, or harboring p*RS316* as the control vector. Cells were grown in 2 mL YPD for 17 h before harvesting. Average relative MAPK activity for at least 3 biological replicates (*n* ≥ 3) is reported, with WT values set to 1. Error bars represent standard deviation. Asterisk, *p*-value <0.05 by Student’s t test compared to wild type. The purple box encloses alleles within the newly identified specificity region. (C) Plate-washing assay. Performed with the same strains as panel B. Cells were grown for 3 days on YPD medium. Top row, before wash images, bar = 0.5 cm. Bottom row, inverted after wash images of invasive scar. Number value represents the average relative invasive growth across three biological replicates (*n* = 3), with WT values set to 1. Standard deviation is displayed in Figure S15. Asterisk, *p*-value <0.05 by Student’s t test compared to wild type. (D) Halo assay. Performed with the same plasmids as panel B, but in the *ste20*Δ mutant (PC7871). Cells were grown for 1 day on an SD-URA medium. 10 μl of ***α***-factor was spotted. Bar = 1.5 cm. Growth in Halo refers to measuring the degree of growth of cells within the halo measured as a percentage by ImageJ. A value of >90% represents full growth. A value of <5% represents no detectable growth. (E) Model depicting the functions of the newly identified specificity region (T712 and Q715) and the known active site (D739). (F) Immunoblot analysis of Ste20p-GFP levels. Same strain and plasmids were used from panel D. Cells were grown at low cell density, harvested, and cell extracts were probed with antibodies to detect GFP (Ste20p-GFP) and Pgk1p as a control for protein levels. Ratio indicates the quantified ratio of the relative band intensities of Ste20p-GFP/Pgk1p normalized to wild-type values that were set to a value of 1. Black arrows, molecular markers (kDa). Full blot image in Figure S17.

To functionally test the SNP data, several genes were chosen for follow-up analysis. One gene chosen was *STE20*, which encodes a p21-activated kinase (PAK). PAK kinases are evolutionarily conserved regulators of signaling and morphogenetic processes across eukaryotes.^112^^,^^113^^,^^114^ In yeast, *STE20* is a component of the fMAPK pathway. Ste20p also regulates the Mating pathway [Figure 1A,^115^^,^^116^], which controls the ability of haploid cells to mate and form diploids. Ste20p was chosen because of the large number of individuals carrying alternative SNPs (Figure 2B, 43.9% of the collection), even though the *STE20* gene has a relatively low number of mutations per site (Figure 2C, 0.007). Furthermore, studying Ste20p provides an opportunity to explore how variation in a protein that is shared between pathways may influence different types of effector phenotypes [i.e., specificity^76^^,^^117^^,^^118^]. In *STE20*, 21 SNPs were identified and mapped to established domains and uncharacterized regions of the protein (File S1, a subset of alleles in Figure 3A).

To determine the functional impact of the SNPs in *STE20*, each SNP was introduced into a plasmid harboring a functional epitope-tagged version of *STE20-GFP* by site-directed mutagenesis and evaluated in a haploid strain (**Σ**1278b background) lacking Ste20p (*ste20*Δ) for MAPK pathway activity, invasive growth, and mating. The **Σ**1278b background is a standard model to study filamentous growth^58^^,^^59^; however, it is worth noting that some allelic effects might vary across strain backgrounds as has been previously reported.^100^^,^^101^^,^^102^^,^^103^^,^^104^^,^^105^^,^^123^^,^^124^ MAPK pathway activity was evaluated using a transcriptional (*FUS1*-*lacZ*) reporter by the β-galactosidase assay. This test showed that 9 of the 21 SNPs (43%) had reduced MAPK pathway activity (Figure 3B, full dataset in Figure S14). These 9 SNPs can be considered loss-of-function (LOF) alleles, although some were more defective than others resulting in a graded effect on MAPK pathway activity. The 9 SNPs are carried by 28 strains (File S3, 1.7% of the collection) with 24 of the 28 being heterozygous for the allele (File S3). Furthermore, 8 of the 9 SNPs (89%) occurred in amino acid residues that are conserved in PAK kinases in humans (Figure 3A, conserved, alignments in Data S1), suggesting their functional significance may be conserved (Figure 3A, Known Function).

By the PWA, 7 of the 21 SNPs (33%), which overlap with the SNPs that showed reduced MAPK activity, also showed reduced invasive growth (Figure 3C, full dataset and quantification in Figure S15). As for MAPK activity, the SNPs showed a range of invasive growth defects (Figure 3C, 1.2-fold to >10-fold). For example, *STE20*^*D739Y*^ showed no MAPK pathway activity or invasive growth, whereas *STE20*^*P479H*^ showed modest defects (Figures 3A–3C).

In the Mating and fMAPK pathways, Ste20p is activated by the same protein, Cdc42p, and phosphorylates the same MAPKKK, Ste11p. To determine whether alleles of *STE20* also impact mating, cells were examined by the halo assay, where the addition of pheromone (***α***-factor) arrests the cell cycle and causes a halo of “no growth” on semisolid agar medium.^125^ The halo assay showed that 7 out of 21 SNPs (33%), again overlapping with those showing reduced MAPK activity, showed a reduced mating response (Figure 3D, full dataset in Figure S16A, *STE20*^*V646M*^ shows reduced mating at a later timepoint in Figure S16B). As for invasive growth and MAPK activity, the alleles showed a graded effect on halo formation. To show the graded effect, growth within the halo was measured by ImageJ. Haloes where no growth occurred, like in wild type, were represented by a value of <5% (out of 100%). Haloes where full growth occurred, like for the *ste20*Δ mutant, were represented by a value of >90% (Figure 3D). Several alleles were fully defective in the response to α-factor (Figure 3D, >90%, *STE20*^*S502L*^, *STE20*^*V646E*^, *STE20*^*T712A*^, and *STE20*^*D739Y*^), whereas others showed a moderate reduction [*STE20*^*P479H*^, 25%; and *STE20*^*N676D*^, 30%], and one showed a slight reduction at a later time point (Figure S15B, *STE20*^*V646M*^). Furthermore, different amino acid substitutions at the same site (e.g., V646) had drastically different effects on phenotype. *STE20*^*V646M*^ showed a slight reduction in MAPK activity (Figure 3B) and mating (Figure S16B), whereas *STE20*^*V646E*^ was fully defective (Figures 3B–3D).

Some SNPs impacted one pathway more than another. *STE20*^*V646E*^ and *STE20*^*S502L*^ showed a larger defect in mating compared to filamentous growth (Figure 3). Two SNPs in a conserved region between helices αD and αE (site T712 and Q715) near the active site (D739) showed striking differential effects on the fMAPK and Mating pathways (Figures 3B–3D, purple box). For example, *STE20*^*Q715L*^ reduced invasion but not halo formation (Figures 3C and 3D), suggesting it is involved in fMAPK pathway specificity. By comparison, *STE20*^*T712A*^ showed a moderate reduction in invasive growth (Figure 3C) and a complete loss of halo formation (Figure 3D), suggesting it may be involved in specificity to the Mating pathway. Thus, we have uncovered a new functional region that affects Ste20p specificity (Figure 3E), which may bind different pathway-specific factors. This region is separate from the site where Ste20p and another PAK kinase, Cla4p, differ,^126^ and from the binding site of the G_β_ subunit Ste4p, which directs Ste20p to the mating pathway.^127^

Protein turnover is one way to modulate pathway activity. Ste20p is turned over by the 26S proteasome when not associated with the active or GTP-bound conformation of Cdc42p.^128^ The 9 alleles that showed phenotypic consequences on MAPK activity were tested for effects on Ste20p protein levels. *STE20*^*S502L*^, *STE20*^*T712A*^, and *STE20*^*Q715L*^ showed reduced Ste20p-GFP levels (Figure 3F, full blot in Figure S17A). These results suggest that the function of the specificity region containing sites T712 and Q715 may be tied to protein stability (Figure 3E). *STE20*^*S502L*^ in particular showed a truncated product of Ste20p-GFP that may suggest this allele leads to a truncated version of the protein (Figure S17A). Some variation in protein stability depended on growth conditions, suggesting some alleles may regulate stability only in a specific context (Figures S17B-S17D, e.g., *STE20*^*W365R*^, *STE20*^*V646E*^, *STE20*^*Q715L*^). Collectively, the results highlight the possible natural phenotypic diversity that exists due to LOF alleles in a PAK kinase. Moreover, analyzing natural alleles is a valid approach to uncover new determinants of PAK kinase function, specification, and stability.

### Gain-of-function alleles in a G-protein alpha subunit stimulate filamentous growth

As a separate test, alleles in a different type of signaling protein were examined. G-proteins are structurally and functionally distinct from protein kinases and may harbor different types of impactful alleles. One type of G-protein are members of the heterotrimeric G-protein family (α, β, γ) that functions as an effector of G-protein coupled receptors (GPCRs).^46^ The G-protein α-subunit Gpa2p is a regulator of the protein kinase A (PKA) pathway that controls filamentous growth (Figure 1A^129^^,^^130^). G***α***-proteins contain five recognizable GTP-binding motifs (G1, G2, G3, G4, and G5) that are universally conserved across G***α***-proteins (Data S2). Our pipeline identified 16 SNPs in *GPA2* across the collection that mapped to GTP-binding motifs and uncharacterized domains of the protein (File S1).

**Figure 4:**
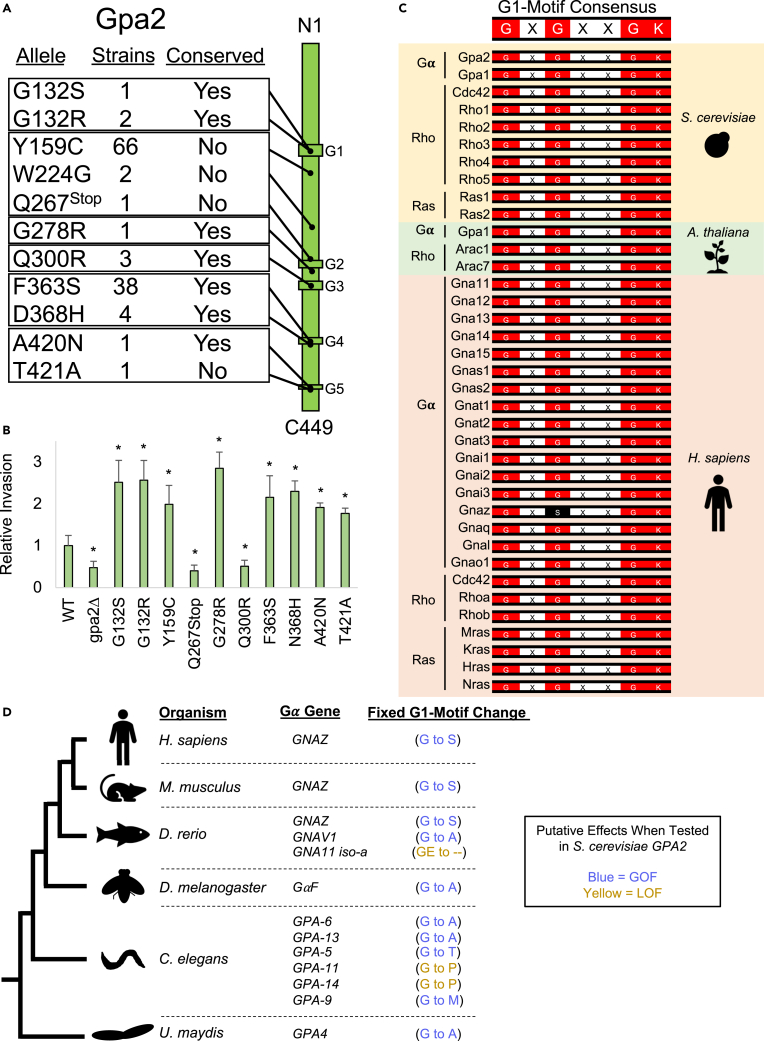
SNPs within a G*α*-protein reveal activating GOF mutations, and the same types of alleles are fixed in some metazoans Analysis of SNPs generated by site-directed mutagenesis in p*GPA2*. (A) Protein map of Gpa2p showing a subset of substitutions found to cause a phenotypic effect. Number of strains represents the number of individuals out of 1660 that carry the amino acid change. Conserved, yes, represent sites that are found conserved in G***α***-proteins across eukaryotes (alignments in Data S2). (B) Plate-washing assay. Cells of the *gpa2*Δ mutant (PC7893) were grown harboring p*GPA2* with no change (WT), harboring p*GPA2* with the indicated amino acid change, or harboring p*RS315* as the control vector. Cells were grown for 3 days on YPGAL medium after which the PWA was performed. Average relative invasive growth for at least 3 biological replicates (*n* ≥ 3) is reported, with WT values set to 1. Error bars represent standard deviation. Asterisk, *p*-value <0.05 by Student’s t test compared to wild type. Images in Figure S18. (C) Alignment of the consensus sequence of the G1-motif for a subset of GTP-binding proteins in *S. cerevisiae*, *Arabidopsis thaliana*, and *Homo sapiens*. Red, conserved amino acid sites. Black, fixed amino acid change (G to S) in the GNAZ protein. G***α***, G***α***-protein; Ras, Ras protein; Rho, Rho GTPase. Additional sequences and species in File S4. (D) Left, cladogram of *Ustilago maydis*, *Caenorhabditis elegans*, *Drosophila melanogaster*, *Danio rerio*, *Mus musculus*, and *H. sapiens*. G***α***-gene, genes encoding G***α***-proteins with a fixed amino acid change in the third amino acid of the G1-motif consensus sequence (GXGXXGK). Fixed G1-motif change, shows the fixed amino acid substitutions. Blue, GOF change; yellow, LOF change. Supporting plate-washing assay data in Figure S21.

To determine the functional impact of these alleles, each SNP was introduced into a plasmid harboring a functional version of *GPA2* by site-directed mutagenesis and evaluated in a haploid strain (**Σ**1278b background) lacking Gpa2p (*gpa2*Δ) for invasive growth. Of the 16 SNPs present in the collection, 8 (50%) showed a GOF phenotype by conferring higher levels of invasive growth (Figures 4A and 4B, e.g., *GPA2*^*G132S*^ and *GPA2*^*G132R*^, PWA images in Figure S18), whereas 2 (13%) showed a LOF phenotype with reduced invasive growth (Figure 4B, e.g., *GPA2*^*Q300R*^). Of the 8 GOF SNPs, only 1 was outside of a GTP-binding motif (Figures 4A and 4B, *GPA2*^*Y159C*^). In total, 11 of the 16 SNPs (70%) had a detectable phenotype and were carried by 120 strains (File S3, 7.2% of the collection) with 58 of the 120 being heterozygous for the allele (File S3). Thus, 7% of the global collection carries a GOF allele in *GPA2*, whereas only 0.2% carries a LOF allele.

G-proteins become activated by a conformational change that results from GTP binding.^46^ Alleles in G-proteins have been previously identified that “lock” the proteins in their active or GTP-bound state, such as the canonical hyperactive allele *GPA2*^*G132V*^ that hyper-activates the PKA pathway.^131^^,^^132^ Although the *GPA2*^*G132V*^ allele was not present in the global collection, we generated it using CRISPR-Cas9 and compared it to the *GPA2*^*G132S*^ allele also generated by CRIPSR-Cas9. The increase in filamentous growth induced by the *GPA2*^*G132S*^ allele shown above matched *GPA2*^*G132V*^ (Figure S19). Furthermore, the deletion of Rgs2p – the GTPase-activating protein (GAP) and negative regulator for Gpa2p^132^ – matched the invasion for *GPA2*^*G132S*^ and *GPA2*^*G132V*^ (*rgs2*Δ, Figure S19). Thus, GOF alleles that lock G***α***-proteins in an active state occur in natural populations and provide a mechanism for activating signaling pathways, presumably by expanding phenotypic diversity.

*GPA2* was more prone to carry GOF alleles than *STE20* (*STE20* – 0% GOF, *GPA2* – 50% GOF). This may be explained by the prevalence of alleles in GTP-binding sites that presumably lock the Gpa2p protein in its active state. An enrichment for this type of allele was evident because 50% of the SNPs (8/16) in *GPA2* occurred in the conserved GTP-binding sites (File S1), which only make up 9% of the protein (42aa/449aa). Moreover, 7 of the 8 SNPs (88%) that fell into GTP-binding sites were GOF (excludes *GPA2*^*Q300R*^). Thus, different types of signaling proteins display different compositions of the types of alleles they harbor.

### Substitutions in the G1 and G3 motifs of G-proteins occur in populations of other species

The prevalence of GOF alleles in a G-protein subunit prompted us to examine this question more broadly. Of the 11 impactful SNPs in *GPA2* discussed above, 7 occurred in amino acid sites conserved in G***α***-proteins across eukaryotes (Figure 4A, conservation shown in Data S2). Moreover, the G1 and G3 motifs are conserved not only across heterotrimeric G-proteins but also across a larger group of GTP-binding proteins that encompass members of the monomeric (Rho and Ras) superfamily of G-proteins (Figure 4C, additional sequences in File S4). Mutations that lock these proteins in their GTP-bound conformation are also conserved. Therefore, GOF alleles in the G1 and G3 motif of *GPA2* may be expected to reflect the same effect in other G-proteins from other organisms. This is further suggested by corresponding sites in human RAS, for example, where substitutions in the G1 motif (G12D, G12V, G12C, G13D) and G3 motif (Q61R) hyperactivate RAS-MEK-ERK signaling and cause cancer in humans.^19^^,^^20^

We asked if substitutions in the G1 and G3 motifs in G-proteins occur at the population level in other species of microorganisms, such as *Candida albicans*. *C. albicans* is a major human fungal pathogen that causes infections, particularly in immunocompromised individuals.^62^^,^^133^^,^^134^^,^^135^^,^^136^ By examining genome assemblies of 102 clinically derived strains of *C. albicans,* a single isolate (1%) was found that contained an amino acid substitution in the G1 motif of Ras1p [Data S3, G13R, strain ATCC10231]. Ras1p regulates signaling-dependent phenotypes related to virulence,^137^^,^^138^^,^^139^ and based on the phenotype of the same substitution in *S. cerevisiae* Gpa2p (*GPA2*^*G132R*^, Figure 4B), *RAS1*^*G13R*^ may be a hyperactive protein that induces Ras- and PKA-dependent phenotypes in *C. albicans*.

We also examined G-proteins across populations of a multicellular organism. In the model invertebrate nematode *Caenorhabditis elegans*, use of the CaeNDR database (https://caendr.org) identified a single individual (1/550, 0.2%, strain ECA189) that harbored an amino acid substitution in the G3-motif (Q215E) in the G***α***-protein gpa-6, a protein that may be involved in chemotaxis.^140^ When introduced into the *S. cerevisiae* Gpa2p, the equivalent change of Q215E (i.e., Q300E) increased invasive growth (Figure S20). This may imply that this substitution would increase signaling in *C. elegans* as well, although this possibility was not tested. Thus, alleles that might be expected to result in activating GOF G-proteins potentially exist in populations of other species.

Alleles resulting in amino acid substitutions in the G1 motif (corresponding to site G132 in *S. cerevisiae* Gpa2p discussed above) of G***α***-proteins were also identified as fixed alleles (i.e., alleles found in all individuals of a species/population) in several species. One fixed allele resulted in a G to S substitution in the G1 motif of a G***α***-protein called GNAZ that is conserved across vertebrates, including humans (Figure 4C, third column, black; Figure 4D). In vertebrates, this protein is mostly found in the brain, adrenal medulla, and platelets, and has been shown *in vitro* to be a GOF allele due to its elevated level of GTPase activity.^141^^,^^142^^,^^143^ As discussed above, this substitution in the *S. cerevisiae* Gpa2p showed hyperactivity (Figure 4B, *GPA2*^*G132S*^), which is consistent with these previous findings performed with mammalian homologs. Thus, at least one example of a GOF allele resulting in a hyperactive G-protein became fixed in the vertebrate lineage.

Additional examples of fixed substitutions in the G1 motif expected to produce GOF alleles in G-proteins were found in other eukaryotes. In *Ustilago maydis,* a fungal pathogen also known as corn smut, the GPA4 protein contains a G to A change as the predominant allele (Figure 4D). The same substitution was also present in the G***α***-proteins of other species including two G***α***-proteins of nematodes (*C. elegans,* gpa-6 and gpa-13), a G***α***-protein in fruit flies (*Drosophila melanogaster*, G***α***F), and a G***α***-protein in zebrafish (*Danio rerio*, GNAV1) [Figure 4D]. The substitution of G to A in the corresponding site of *S. cerevisiae* Gpa2p (G132A) increased invasive growth (Figure S21); therefore, this change might also cause G-protein hyperactivity in other G***α***-protein homologs (Figure 4D, blue). Additionally, two other substitutions were found that had become fixed in different *C. elegans* G***α***-proteins (Figure 4D, blue, G to T in gpa-5 and G to M in gpa-9). The corresponding substitutions led to an increase in invasive growth when tested in *S. cerevisiae* Gpa2p (Figure S21). Two other substitutions in G***α***-proteins in nematodes and one G***α***-protein in zebrafish (Figure 4D, G to P in gpa-11 and gpa-14, GE to -- in GNA11 iso-a) also led to reduced invasive growth when tested in *S. cerevisiae* Gpa2p (Figure S21). Thus, several examples of substitutions in the G1 motif in GTP-binding proteins that potentially affect their activity have become fixed in other species (Figure 4D). Several of these examples may act as GOF alleles (Figure 4D, blue) but further testing will be needed to explore this possibility.

### Structural changes in mucin tandem repeats identified in mitogen-activated protein kinase pathway mucin signalers

Structural variation can also contribute to phenotypic diversity. One example comes from changes to the repeat region of proteins.^144^^,^^145^^,^^146^ Mucins for example contain S/T/P-rich repeat regions,^147^^,^^148^ which can vary in number across individuals. In the fungal pathogen *C. albicans*, adhesin mucin repeat variation occurs in healthy yeast but has no clear phenotypic consequence.^149^ In humans, variation in the repeat region of the signaling mucin MUC1 has been linked to kidney disease^150^^,^^151^^,^^152^ and gut bacterial infections,^153^ however, the function of variation outside of the disease state is less clear. Therefore, how variation in signaling mucin repeat regions contributes to healthy phenotypic diversity across a population is a remaining question.

**Figure 5:**
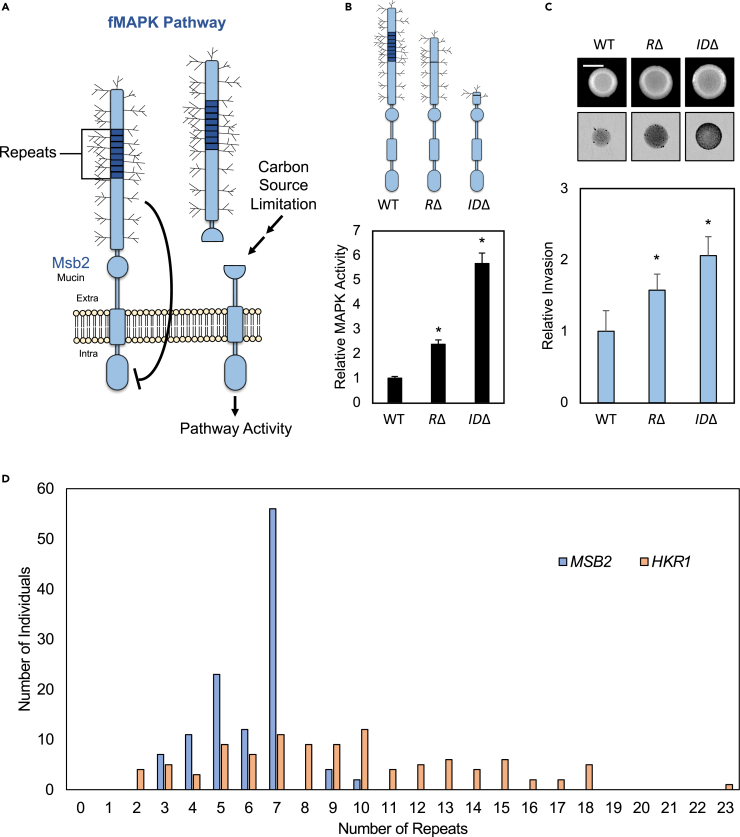
Losses of mucin repeats in a mucin signaler generate GOF alleles that contribute to MAPK signaling variation (A) Model of fMAPK pathway activation by Msb2p after cleavage of its extracellular domain. (B) β-galactosidase assays measuring MAPK pathway activity. Cells of wild type (PC538), cells with the deletion of the inhibitory extracellular domain of Msb2p [IDΔ (PC3384)], and cells with the deletion of the mucin repeat region [RΔ (PC7834)] were grown in 5 mL SD medium for 16 h before harvesting. Average relative MAPK pathway activity for at least 3 biological replicates (*n* = 3) is reported, with WT values set to 1. Error bars represent standard deviation. Asterisk, *p*-value <0.05 by Student’s t test compared to wild type. (C) Plate-washing assay. Same strains as panel B were spotted onto SD medium for 3 days. Top images, before wash, Bar = 0.5 cm. Bottom row, inverted images of invasive scar after wash. Bar graph, the average of at least 3 biological replicates (*n* = 3) is reported, with WT values set to 1. Error bars represent standard deviation. Asterisk, *p*-value <0.05 by Student’s t test compared to wild type. (D) Histogram displaying the number of individuals (out of 117) with an indicated number of tandem repeats for the signaling mucins *MSB2* (blue) and *HKR1* (orange).

The fMAPK pathway is regulated by the mucin sensor Msb2p,^154^ which contains an S/T/P-rich repeat region (Figure 5A, left). Carbon source limitation triggers proteolytic cleavage and shedding of the inhibitory extracellular domain^154^^,^^155^^,^^156^ to activate the fMAPK pathway (Figure 5A, right). The deletion of the repeat region by CRISPR-CAS9 resulted in elevated MAPK pathway activity (Figure 5B, RΔ), although not to the extent of deleting the entire inhibitory extracellular domain (Figure 5B, IDΔ). The increase in pathway activity resulted in increased invasive growth (Figure 5C) demonstrating that the repeat region of Msb2p functions to inhibit fMAPK pathway activity and filamentous growth.

To address if variation in the repeat number of a mucin signaler occurs across healthy yeast strains, long-read sequences from a subset of diverse *S. cerevisiae* isolates were examined.^56^ Repeat numbers determined by long-read sequences in several strains were validated by PCR analysis (Table S1, PCR gels in Figure S22). Moreover, PCR analysis alone was used for several strains where long reads were not available (Table S1; Figure S22). Across 117 strains tested, Msb2p showed variation in repeat number (Figure 5D; Table S1). Most strains showed seven repeats as is found in the reference strain; however, strains with three (7 strains), four (11 strains), five (23 strains), six (12 strains), nine (4 strains), and ten (2 strains) repeats occurred (Figure 5D, blue). Because elevated MAPK pathway activity can occur by loss of a single repeat,^154^ strains containing three to six repeats (53 out of 117 strains, 45%) would be predicted to show a modest but detectable increase in fMAPK pathway activity. Similarly, because the addition of a single repeat can dampen MAPK pathway activity,^157^ individuals with nine and ten repeats (6 out of 117 strains, 5%) would be predicted to show reduced MAPK pathway activity.

The related signaling mucin Hkr1p regulates the HOG pathway^158^ and also showed variation across strains, even more so than Msb2p (Figure 5D, orange, range from 2 to 23 repeats). Changes in Msb2p and Hkr1p repeat numbers did not correlate with each other (Figure S23A). Which mucin had more repeats also varied between strains, with Msb2p showing more repeats than Hkr1p in some strains and Hkr1p showing more repeats in others (Figure S23B). Thus, Msb2p and Hkr1p repeat regions vary independently of each other. Overall, these results identify structural variation of the mucin repeat region as a separate mechanism by which signaling pathway variation occurs across populations of individuals.

## Discussion

How phenotypic diversity is generated is a critical question in biology. We found that phenotypic diversity can be caused by wide variability in the activity of different signaling pathways across individuals. Surveying the global yeast collection to identify the genetic basis of signaling variation identified 1957 predicted impactful SNPs in a regulatory module that controls cell differentiation. These included loss-of-function (LOF) alleles in a PAK kinase, activating gain-of-function (GOF) alleles in a G***α***-protein, and LOF and GOF alleles in the repeat region of a signaling mucin. The LOF alleles revealed new insights into PAK function and stability, and uncovered a region (A712 to Q715) in the kinase domain related to specificity. The GOF alleles revealed a role for hyperactivated signaling G-proteins in generating phenotypic diversity outside of the disease state in natural populations. In one case, a GOF allele has become fixed in vertebrates (i.e., GNAZ). Collectively, our results show how signaling pathways act as reservoirs of standing genetic variation to generate phenotypic diversity across individuals.

LOF alleles are major drivers of phenotypic variation.^6^^,^^7^^,^^8^ GOF alleles can also generate phenotypic variation but are less well understood at the population level. Here, we report different types of GOF alleles that exist in a signaling module within the global collection of yeast. Of the SNPs that yielded a phenotype in Ste20p and Gpa2p, 45% (9/20) were GOF alleles while 55% (11/20) were LOF alleles. For the mucin Msb2p, 45% (53/117) of individuals tested harbored putative GOF alleles. The fact that nearly half of the alleles tested produced GOF phenotypes suggests that GOF mutations can be widespread in populations.

GOF alleles that switch signaling pathways by locking a component in its active state might be expected to have detrimental consequences on fitness, and this is certainly true for some alleles; however, evidence suggests this may not always be detrimental and possibly even adaptive or compensatory. One piece of evidence is the prevalence of GOF alleles uncovered in a global population of individuals that do not show defects in growth. A second piece of evidence comes from the fact that GOF alleles become fixed in populations. Additional evidence comes from previous studies that have shown that hyperactivating mutations of the PKA pathway can have beneficial consequences on fitness in constant environments^159^^,^^160^^,^^161^^,^^162^ and that hyperactivating PKA mutations (e.g., *IRA1/2* LOF) have been found in populations of the wild yeast *Saccharomyces paradoxus*.^34^ GOF mutations can also be beneficial when compensating for LOF mutations in an interacting partner, as has been seen for proteins that function in multi-protein complexes.^163^ Thus, GOF alleles in signaling pathways (especially the PKA pathway in yeast) can be tolerated in some contexts and possibly offer an adaptive or compensatory strategy in natural populations.

MAPK pathways differed from the PKA pathway in this regard, however. For MAPK pathways, although we found structural variations in a mucin that are predicted to increase MAPK pathway activity, we did not find evidence of GOF alleles that lock the pathway in an activated state. For example, we found no constitutively activated MAPK pathways when directly testing wild strains. Furthermore, mutations identified in genetic screens that constitutively activate the MAPK pathway were not found in the global collection (e.g., *STE11-1*, *STE11-4*,^164^ or *SHO1*^P120L^^156^). These findings suggest two things. First, alleles identified by natural variation can differ from alleles identified by genetic approaches (e.g., high-throughput screens^165^ and genetic screens^164^), revealing the utility of the different approaches. Second, alleles that constitutively active MAPK pathways may be detrimental to fitness in the wild, and may only be tolerated in laboratory settings. Therefore, MAPK pathway levels can vary across the population but may never enter a hyperactive state such as seen for the PKA pathway.

Several SNPs in *STE20* reduced filamentous growth as well as the cell’s response to pheromone. Therefore, one cost to modulating filamentous growth may be a defect in sexual reproductive fitness. This cost may be mitigated in some strains with polyploidy or aneuploidy, which occurs in numerous strains.^54^ This is because polyploidy and aneuploidy increase fitness in some contexts^166^^,^^167^^,^^168^ while making mating challenging or impossible, and therefore obsolete. Of the strains carrying *STE20* alleles from this study that reduce pheromone sensitivity, 27% (7/26) have aneuploidy (File S3). In other contexts, sexual reproduction defects may be mitigated because cells presumably gain a subtle advantage in growth when mating is compromised.^169^ In domesticated contexts, strains may experience relaxed selection on sexual reproduction, as domestication impacts the yeast life cycle (e.g., reduces sporulation efficiency), due to an increase in aneuploidies and LOF mutations.^170^ Thus, LOF alleles in mating may arise due to a trade-off with filamentous growth, relaxed selection in contexts where mating is obsolete, and/or by positive selection where a loss of mating is advantageous.

In summary, the prevalent role of signaling pathways in generating phenotypic diversity was revealed by exploring natural variation. 54% (20/37) of tested SNPs in this study showed a functional consequence, which by extrapolation to all 1957 SNPs, suggests more than a thousand SNPs (1057) in the signaling module may contribute to phenotypic diversity. This large number of SNPs may be an underestimate, as SNPs with no identified function may show a phenotype by exploring other environments and assays.^105^^,^^107^^,^^171^ We suggest that variation in signaling pathways across individuals is a major driver of phenotypic diversity in other organisms as well. In humans, this variation may be relevant to health. This is evident because disease prognosis and treatment regimens during individualized medicine are developed based on genetic differences among individuals (i.e., precision medicine^172^^,^^173^^,^^174^^,^^175^). Overall, exploring natural genetic diversity in signaling pathways creates a resource that can reveal new insights into protein function and the genetic basis underlying genotype to phenotype.

### Limitations of the study

Our findings suggest that more than one thousand SNPs identified in the study have the potential to be functionally relevant and may contribute to phenotypic diversity. However, because of the limited number of genes fully explored (*STE20*, *GPA2*, and *MSB2*), future testing on a larger set of genes will be needed to determine the functional/phenotypic relevance at larger scales. For functional testing of different alleles, a single strain background (**Σ**1278) was used as a standard model to study filamentous growth. This strain enabled screening SNP function by generating and testing 42 unique SNPs for phenotypes related to filamentous growth. However, some allelic effects might vary across strain backgrounds as has been reported,^100^^,^^101^^,^^102^^,^^103^^,^^104^^,^^105^^,^^123^^,^^124^ suggesting that testing alleles in different backgrounds would further define the functional relevance of any given SNP.

## Resource availability

### Lead contact

Further information and requests for resources should be directed to Paul J. Cullen (pjcullen@buffalo.edu).

### Materials availability

Plasmids and strains generated in this study are available upon request.

### Data and code availability

• **Data:** Original western blot images are supplied in the supplemental information of this study. Microscopy data reported in this article will be shared by the lead contact upon request.
• **Code:** All original code is available in this article’s supplemental information. For further information and requests, please contact Paul J. Cullen (pjcullen@buffalo.edu).
• **Additional information:** Any additional information required to reanalyze the data reported in this article is available from the lead contact upon request.

## Acknowledgments

Thanks to Feng-Yan Bai from the 10.13039/501100021831Institute of Microbiology, Chinese Academy of Science, Aimee Dudley from the Pacific Northwest Research Institute, and Charlie Boone from the 10.13039/501100003579University of Toronto for providing strains and plasmids. Thanks to Omer Gokcumen, Nate Bakenstose, and Trevor Krabbenhoft from the 10.13039/100008209University at Buffalo for helpful discussions. Thanks to members of the Cullen Lab for providing feedback on the project. Thanks to Katie and Avery Vandermeulen for their support.

The work was supported by a grant from the 10.13039/100000002NIH (GM098629).

## Author contributions

MDV proposed questions, designed and performed experiments, analyzed data, wrote the article, and edited the article; SK designed and performed experiments and data analysis; GR performed experiments; GL designed experiments; PJC designed experiments, edited the article, and acquired funding for the project.

## Declaration of interests

The authors have no competing interests in the study.

## STAR★Methods

### Key resources table

**Table:** 

REAGENT or RESOURCE	SOURCE	IDENTIFIER
**Antibodies**		

p42/p44 primary antibodies	Cell Signaling Technology, Danvers, MA	#4370; RRID: AB_2315112
Phospho-p38 primary antibodies	Cell Signaling Technology, Danvers, MA	#9211; RRID: AB_331641
Pgk1p primary antibodies	Thermo Fisher Scientific, Rockford, IL	#459250; RRID: AB_2532235
Goat anti-rabbit IgG-HRP	Jackson Immuno- Research Laboratories, West Grove, PA	#111-035-144; RRID: AB_2307391
Goat anti-mouse	Bio-Rad Laboratories	#170-6516; RRID: AB_2921252
Mouse anti-GFP	Fisher Scientific	Roche #11814460001; RRID: AB_390913

**Experimental models: organisms/strains**		

*Saccharomyces cerevisiae* lab strains	Parental strains (Liu et al.^176^; Cullen et al.^154^)	See Table S2
*Saccharomyces cerevisiae* wild strains	Chinese primeval forests (Duan et al.^88^)	See Table S2

**Oligonucleotides**		

Primers for PCR amplification	MilliporeSigma (https://www.sigmaaldrich.com/US/en)	See Table S3

**Recombinant DNA**		

*STE20* Plasmid	(Leberer et al.^177^)	p*STE20-GFP*
*GPA2* Plasmid	https://horizondiscovery.com/en/non-mammalian-research-tools/products/molecular-barcoded-yeast-moby-orf-library	p*GPA2*

**Software and algorithms**		

Seaview	PRABI-Doua	https://doua.prabi.fr/software/seaview
Scripts for code	This study	See Data S4 and S5
Image Lab	Bio-Rad	https://www.bio-rad.com/en-us/product/image-lab-software?ID=KRE6P5E8Z

### Experimental model and study participant details

#### Yeast strains and plasmids

Yeast strains (*Saccharomyces cerevisiae*) are listed in (Table S2). FJ7, HN2, HN6, SX1, SX2, SX6, HN9, HN10, HN11, BJ6, BJ14, BJ20, HN14, HN15, and HN16 (PC7324-PC7338) are diploid wild primeval forest strains described previously.^89^ Gene deletions in the haploid **Σ**1278b strain background were made through homologous recombination, constructed using an antibiotic resistance marker (neurotactin or gentamicin) amplified by polymerase chain reaction (PCR) with primers in (Table S3). *GPA2* alleles that were made directly in the genome of the **Σ**1278b strain background (PC7851 and PC7877) were made using the CRISPR-Cas9 system.^178^^,^^179^ The CRISPR-Cas9 system was also used to generate the deletion of the *MSB2* mucin repeat region in the **Σ**1278b strain background. A lithium acetate transformation as described previously^180^ was used to introduce DNA templates and plasmids into yeast. All gene deletions were verified by PCR amplification and gel electrophoresis of deletion site and by phenotype when possible.

For CRISPR-Cas9, the sgRNA sequence was designed as described previously^67^ using CRISPRdirect [https://crispr.dbcls.jp/]. The sgRNA was cloned into the p*Cas* plasmid by PCR as described previously.^179^ The sgRNA sequences were verified by sequencing the plasmid with GENEWIZ (https://www.genewiz.com/) using the sequencing primer – CGGAATAGGAACTTCAAAGCG.^179^ Primers to generate the p*Cas9*-sg*GPA2* plasmid and HDR templates of *GPA2* containing SNPs are in (Table S3). To delete the *MSB2* mucin repeat region, the forward primer began 60 base pairs upstream of the start of the mucin repeat region and ended at the PAM sequence located 21 nucleotides into the first 54 base pair repeat. The reverse primer was the reverse complement of the 60 base pairs immediately downstream of the Msb2 mucin repeat sequence. This excised most of the first repeat (33/54bp) as well as the entirety of the other 6 repeats.

The p*STE20-GFP*^177^ and the p*GPA2*^181^ plasmids were used for site-directed mutagenesis experiments to generate alleles. The p*STE20-GFP* gene is under the control of its native promoter.^177^ The p*GPA2* gene is also under control of its native promoter and is part of the MoBY ORF 2.0 plasmid collection available at (https://horizondiscovery.com/en/non-mammalian-research-tools/products/molecular-barcoded-yeast-moby-orf-library). Site-directed mutagenesis plasmids were generated using the GeneArt Site-Directed Mutagenesis System Kit from Invitrogen (Catalog # A13282). Primers to generate different alleles can be found in (Table S3). Plasmids were verified by sequencing with GENEWIZ. For experiments with *STE20*, SNPs were introduced into p*STE20-GFP* and then transformed into an indicated *ste20*Δ mutant (PC7871 or PC673). Unaltered p*STE20-GFP* was used for wild-type. p*RS316* was used as a control vector when testing *ste20*Δ. For experiments with *GPA2*, SNPs were introduced into p*GPA2* and then transformed into a *gpa2*Δ mutant (PC7893). Unaltered p*GPA2* was used for wild-type. p*RS315* was used as a control vector when testing *gpa2*Δ.

### Method details

#### Media

YPD: 1% yeast extract, 2% peptone, 2% dextrose; YPGAL: YPD with 2% galactose instead of dextrose; YPD+KCl: YPD with 0.5M or 1M KCl as indicated; YPD+Sorbitol: YPD with 1M Sorbitol; YPD+GLU: YPD with an additional 1M dextrose; SLAD: synthetic low ammonium as described,^59^ 0.17% yeast nitrogen base without amino acids or ammonium, 2% dextrose; SOE: synthetic oak extract,^182^ 1% sucrose, 0.5% fructose, 0.5% glucose, 0.1% yeast extract, 0.15% peptone; SD: synthetic complete medium, 0.67% yeast nitrogen base without amino acids, amino acids, and 2% dextrose (minus uracil when selecting for p*STE20-GFP*, minus leucine when selecting for the p*GPA2* plasmid).

#### Variant effect prediction across the global collection

We used the 1660 natural *S. cerevisiae* strains to study the genetic variations and their functional impact (https://github.com/nicolo-tellini/S.cerevisiaeData/tree/main). We mapped all the SNPs in the 62 genes (belonging to the four pathways examined here) in the natural population against the mutfunc database (http://mutfunc.com) to extract all the impactful mutations affecting protein function and stability. All the SNPs of the population are annotated using SnpEff (https://pcingola.github.io/SnpEff/) and are given in File S6. Predictions on all the annotated SNPs were then extracted from the Mutfunc datasets using the script in Data S4 and the predicted impact of all variants is given in File S7. The deleterious SNPs present in the population in the gene list tested (File S8) is done using the script in Data S5 which outputs the files with mutations with deleterious impact. In our analysis, we focused on mutations occurring in the conserved region (functional impact), mutations impacting protein structure, and mutations impacting interaction interfaces that can affect protein stability. We categorized the mutations as deleterious if they had a sift score of less than 0.05 (for conserved region) and a ddG score (FoldX score) greater than 2 (for protein structure and protein interaction surfaces). All the deleterious mutations are provided in File S1. To note, we observed that the gene *FLO8* was not annotated by snpEff which could be due to the reference genome (i.e., S288C background) containing an internal in-frame stop at codon 142 and hence a poor mapping of the gene to the reference. This could explain why few SNPs were predicted in mutfunc for this gene as it also uses a S288C reference-based system.

The ratio of nonsynonymous to synonymous polymorphisms (dN/dS) was calculated as previously described using a sub-collection of 1,011 yeast strains.^54^ dN/dS was computed using PAML software with the yn00 program for the signaling genes of interest and for all other genes and their distributions were plotted as separate boxplots. A Wilcoxon test was used to compare the distributions and generate a *p*-value. Both Mean and Median values were used for comparison separately.

#### Quantitation of mucin repeats

Mucin repeat number of *MSB2* and *HKR1* were determined by visual inspection from genomes sequenced by long-read methods.^56^ Sequences were downloaded from the NCBI website (https://www.ncbi.nlm.nih.gov/bioproject/PRJEB50706), Accession: PRJEB50706. Repeats were measured by identification of the mucin repeat region in the gene sequence, using conserved regions adjacent to the repeat regions in SnapGene Viewer (https://www.snapgene.com/snapgene-viewer). Sequences flanking the mucin repeat region [the equivalent of two repeat lengths upstream including the repeat region and two repeat lengths downstream (102 for *MSB2* and 168 for *HKR1*)], was pasted into a new FASTA file and opened in Seaview (https://doua.prabi.fr/software/seaview) to convert the DNA into protein sequence. Repeats were inspected using the consensus sequences from UniProt (https://www.uniprot.org/) and compared to the S288C reference sequence with 7 Msb2p repeats and 12 Hkr1p repeats. Msb2p protein consensus sequence (https://www.uniprot.org/uniprotkb/P32334/entry): 17 aa, S-Q-V-S-D-T-[P or S]-V-[P or S]-[Y or S]-T-[T or S]-S-[S or R]-S-S-V. Hkr1p protein consensus sequence (https://www.uniprot.org/uniprotkb/P41809/entry): 28 aa, S-[A or V]-P-V-A-V-S-S-T-Y-T-S-S-P-S-A-P-A-A-I-S-S-T-Y-T-S-S-P. Some strain repeat numbers were also quantified by PCR amplification of the mucin repeat region. PCR was done with the following upstream and downstream primers outside of the repeat regions. For *MSB2*: forward – 5′-CACCCTCTCAGACGACAACT-3’; reverse – 5′-GCGTAGACGTTGCCACTTCC-3’. For *HKR1*: forward – 5′-GTGCTGGCAACTATCCTGAC-3’; reverse – 5′-TGACAGGACAACAAGAGCCG-3’. Repeat size was determined based on the reference genome of S288c on the *Saccharomyces Genome Database* [SGD (https://www.yeastgenome.org/)]. *MSB2* repeat size is 51 bp and *HKR1* repeat size is 84 bp.

#### Making protein maps and alignments of GTP-binding proteins

Protein maps were generated using protein sequence and domain data from the databases *Uniprot* and SGD. Protein lengths and domains were made proportionally using a ratio of 0.0198 cm per amino acid in Microsoft PowerPoint.

G***α***-proteins and PAK kinases were aligned by downloading protein sequences from *Uniprot* and aligning them using Seaview using the basic muscle alignment option. A subset of sequences downloaded from Uniprot for G***α***-proteins and other GTP-binding proteins (Rho and Ras) were manually input into an excel file for ease of observing the aligned G-motifs in File S4. For the alignment of the Ras GTPase *RAS1* from 102 *Candida albicans* isolates, genome data was downloaded from the NCBI website (https://www.ncbi.nlm.nih.gov) and accession numbers and strain names are in File S5. *RAS1* sequences were aligned with the program Seaview using the basic muscle alignment option.

#### Quantification of phenotypes

To measure cell length-to-width ratios, cells were spotted onto the indicated medium for 3 days. The starting OD_600_ of spotted cells was the same across samples (at ∼0.08). For each strain, 10 μL of cells were spotted onto plates. After growth, cells were washed off the surface and cells within the invasive scar were observed by microscopy. Images of cells were taken at 100X magnification by microscopy using differential interference contrast (DIC) imaging with a Zeiss Axioplan 2 microscope. Digital images were acquired with the Axiocam MRm camera. For analysis, Axiovision 4.4 software was used. At least 30 cells near the periphery of the invasive scar were measured per strain and the average cell length-to-width ratio was reported with the standard deviation indicated.

To measure invasive growth, cells were spotted onto indicated medium and grown for indicated number of days. The starting OD_600_ of spotted cells was the same across samples (∼0.08 for wild strains and ∼0.1 for laboratory strains). For each strain, 5 μL of cells were spotted onto plates. The plate-washing assay (PWA) was then performed as previously described,^64^^,^^183^ where a stream of water is used to wash cells off the surface to reveal invasive scars. Invasive growth was then quantified as previously described [Using the program Image Lab https://www.bio-rad.com/en-us/product/image-lab-software?ID=KRE6P5E8Z to calculate (volume/area)/10000,^107^]. Invasion values were averaged across at least three biological replicates and error was determined by standard deviation.

Pectinase activity was assessed by a colorimetric assay previously described^66^^,^^184^ that measures the activity of Pgu1p, a secreted enzyme (polygalacturnase) that degrades plant material. Cells were spotted onto indicated medium [YPD, YPGAL, or SOE] which had 1% polygalacturonic acid added. The starting OD_600_ of spotted cells was the same across samples (∼0.08). For each strain, 3 μL of cells were spotted onto plates. Cells were grown for 2 days and then the plates were covered in a 1% solution of ruthenium red. The plates were left at room temperature for 3 h and then were washed under a light stream of water. Images of plates were captured by ChemiDoc XRS+ molecular imager (Bio-Rad laboratories) under blot/chemicoloric setting with no filter or a Nikon D3000 digital camera. The area of the secretion pattern was determined by using the program GIMP2 (https://www.gimp.org/downloads/) to measure the total area (outer circle) and subtracting from it the colony size (inner circle). Pectinase secretion area was measured in mm^2^ and averaged across three biological replicates and error was determined by standard deviation.

Osmolyte tolerance was determined by growing cells in YPD or YPD with indicated osmolytes added in polystyrene wells (Falcon Microtest Tissue culture plate, 96 Well) for 16 h. Each sample was inoculated at the same initial OD_600_ of ∼0.01. The 16h OD_600_ measurement of growth in the osmolyte stress media was normalized to the 16h OD_600_ measurement of growth in YPD. Three separate biological replicates were measured, and the averages are reported. Error was determined by standard deviation.

Mating sensitivity was determined by the halo assay.^125^ Cells were spread across the top of SD-URA medium and allowed to dry. Next, 3ul and 10ul of ***α***-factor was spotted onto the left and right side, respectively. Cells were allowed to grow for 1 day (or 4 days when indicated) and images were captured using ChemiDoc XRS+ molecular imager as done for invasive growth images. The wild-type *MAT*a mating response is to undergo growth arrest in the presence of ***α***-factor, which forms the halo of no growth. To quantify the mating assay, images of haloes were opened in the program ImageJ (https://imagej.net/ij/). The background was subtracted (set at 200 pixels). Each image was converted to 8-bit and a threshold of 25 was applied to convert it to a binary black and white image. A circle was drawn inside the halo with a pixel height and width of 297x297. The analyze particle tool with default settings was used to calculate the total area of particles inside the halo. The *ste20Δ* mutant value was set to 100% representing a filled in halo, and wild type was set to 0% representing no growth. Values for other strains were converted into a percentage relative to the *ste20Δ* mutant.

#### Measurement of MAPK pathway activity

The fMAPK pathway activity was analyzed by phosphoblot as previously described^171^ by established protocols.^84^^,^^185^ Cells were grown in the indicated medium for the times indicated before harvesting for analysis. Each sample was inoculated at the same initial OD_600_ of 0.16. p42/p44 primary antibodies (#4370; Cell Signaling Technology, Danvers, MA) were used to detect phosphorylated Kss1p. Phospho-p38 primary antibodies (#9211; Cell Signaling Technology, Danvers, MA) were used to detect phosphorylated Hog1p. Goat anti-rabbit IgG-HRP (#111-035-144; Jackson Immuno- Research Laboratories, West Grove, PA) antibodies were used as the secondary antibodies. The loading control, Pgk1p, was detected by mouse ***α***-Pgk1p antibodies (#459250; Thermo Fisher Scientific, Rockford, IL) as the primary antibody and goat ***α***-mouse (#170–6516; Bio-Rad Laboratories) as the secondary antibody. Phosphorylated Slt2p was detected in the same blots by the same antibodies as phosphorylated Kss1p. The blot was imaged by a ChemiDoc XRS+ molecular imager and signal intensity was measured by using the volume tool in the program Image Lab (https://www.bio-rad.com/en-us/product/image-lab-software?ID=KRE6P5E8Z).

MAPK activity was analyzed by the β-galactosidase (*lacZ*) assay as previously described^186^^,^^187^ using the *FUS1-lacZ* mating transcriptional reporter as the readout of MAPK pathway activity. The *FUS1-lacZ* reporter can reflect the activity of the fMAPK pathway in cells lacking an intact mating pathway [*ste4Δ*^154^]. Cells were grown in the indicated medium for the times indicated after an initial starting OD_600_ of 0.002. Cells were harvested by centrifugation and stored at −80° for at least 30 min prior to analysis. The β-galactosidase (*lacZ*) assay was then performed with at least three biological replicates where the average is reported, and error bars represent standard deviation.

#### Measurement of Ste20-GFP levels

Ste20p-GFP levels were measured by immunoblot analysis. Cells were grown overnight, shaking, at 30C in SD-URA (5 mL) to select for the p*Ste20-GFP* plasmid. Each sample was grown for 16 h to a final OD_600_ of ∼1.2 before transferring to fresh media. Cells were then washed and transferred to fresh YPD medium for 4 h. In the initial trial, cells were grown at a low cell density; a 0.6 mL subculture from the overnights were washed and added to YPD (5 mL) medium cultures. In the alternative growth condition trial, cells were grown at high cell density; the full 5 mL overnight culture was washed and resuspended in YPD (10 mL) medium cultures. Cells were then harvested and froze down at −80C. Proteins were extracted and the immunoblot analysis was performed as described.^128^ Briefly, protein extracts were prepared by mechanical disruption with beads followed by a trichloroacetic acid precipitation.^90^ Protein precipitates were analyzed by SDS-PAGE and transferred to a nitrocellulose membrane (Cat#10600003, Amersham Protran Premium 0.45 μm NC, GE Healthcare Life sciences). Primary incubations were performed at 4°C for 16 h and secondary at 20°C for 1 h. Monoclonal mouse anti-GFP antibodies (Fisher Scientific, Roche Cat#11814460001) were used as the primary to detect GFP. Goat ***α***-mouse (#170–6516; Bio-Rad Laboratories) was used as the secondary antibody.

### Quantification and statistical analysis

Statistical tests, parameters, and *p*-values are reported in the figure legends.

## Supplemental information

Document S1. Figures S1–S23, Tables S1–S3, and Data S1–S3

File S1. Protein maps displaying identified alleles within the fMAPK, Mating, HOG, and PKA pathways, related to Figure 2 Proteins and domains are relatively proportional. Amino acid substitutions are displayed followed by the number of strains that carry that substitution within the collection. Domain locations are displayed under the protein name. Blue, fMAPK or shared components; red, Mating components; orange, HOG components; green, PKA components.

File S2. Quantitation of intragenic variation, related to Figure 2 Histograms of the 62 genes tested display the number of individuals carrying no SNPs, one SNP, or multiple SNPs within the same gene. Blue, fMAPK or shared components; red, Mating components; orange, HOG components; green, PKA components.

File S3. Details of strains carrying STE20 and GPA2 alleles that showed a phenotypic consequence, related to Figure 3 and Figure 4 respectively

File S4. Conservation of G-motifs across eukaryotes in Gα-proteins, Rho GTPases, and Ras GTPases, related to Figure 4 *GPA2* G-motifs are in red text. Strongly conserved amino acid sites across all GTP-binding proteins are highlighted in orange. Strongly conserved amino acid sites across G***α***-proteins are highlighted in yellow. Not conserved amino acid changes are highlighted in blue.

File S5. Accession numbers for 102 Candida albicans genomes used in this study, related to Figure 4

File S6. All SNPs of the 1600 strain population annotated using SnpEff, related to Figure 2

File S7. Predicted impact of variants extracted, related to Figure 2

File S8. List of genes tested in this study, related to Figure 2

Data S4. Script for extracting annotated SNPs with predicted impact from the Mutfunc datasets, related to Figure 2

Data S5. Script to extract the deleterious SNPs present in the population in the gene list which outputs the files with mutations with deleterious impact, related to Figure 2
