# Supplementary material for: Gain- and loss-of-function alleles within signaling pathways lead to phenotypic diversity among individuals
Source: iScience. 2024 Aug 31;27(10):110860. doi: 10.1016/j.isci.2024.110860 (PMC11460476; doi:10.1016/j.isci.2024.110860)
Supplement: Document S1. Figures S1–S23, Tables S1–S3, and Data S1–S3 [file mmc1.pdf]

iScience, Volume *27*

Supplemental information

Gain- and loss-of-function alleles within signaling pathways lead to phenotypic diversity among individuals

Matthew D. Vandermeulen, Sakshi Khaiwal, Gabriel Rubio, Gianni Liti, and Paul J. Cullen

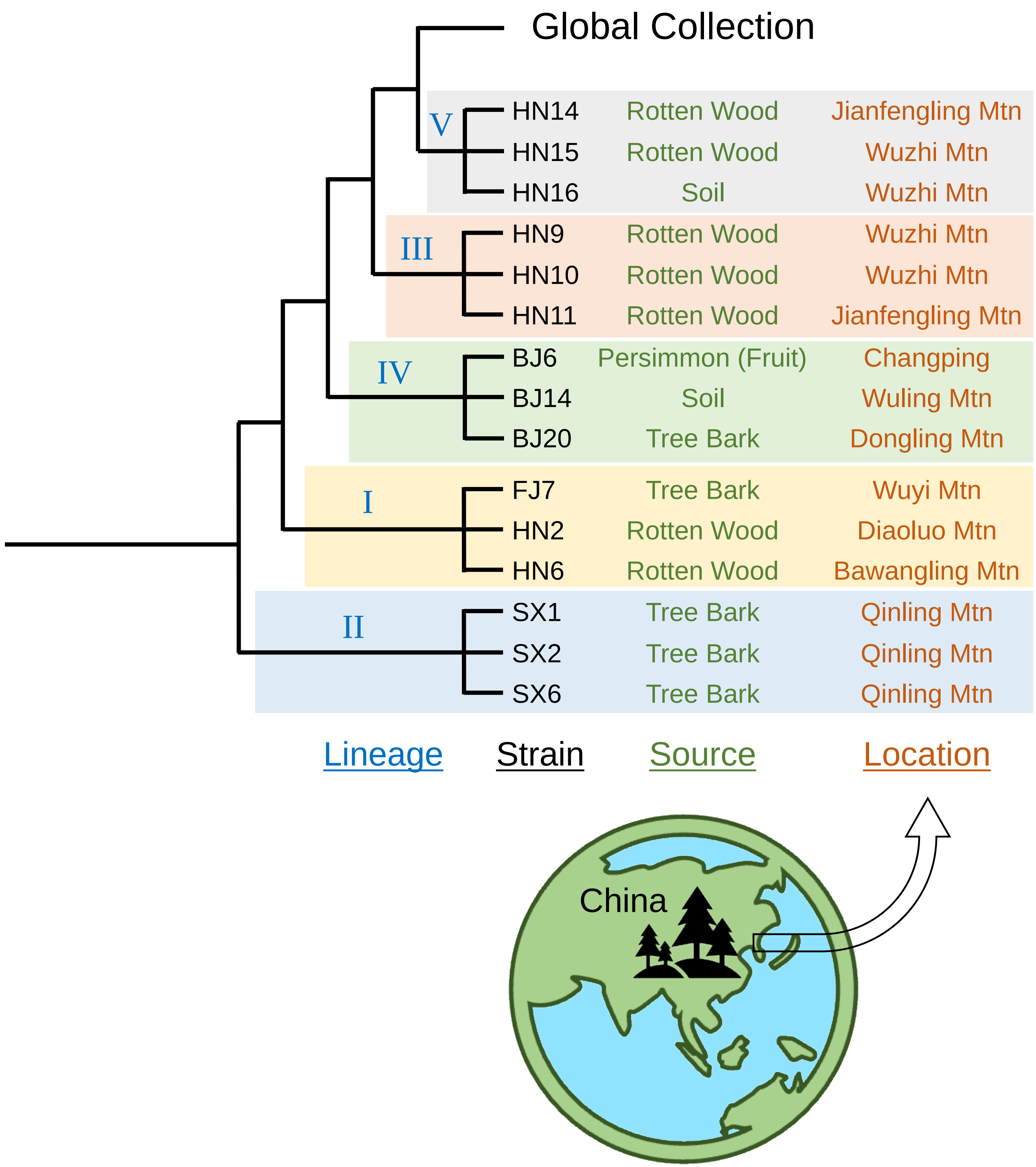

Figure S1. Cladogram of wild primeval forest *S. cerevisiae* strains used in this study, related to Figure 1. Cladogram is based on phylogenetic data determined in [88]. Lineage, lineages I – V were used with three representative individuals from each lineage. Source and location of isolation are displayed from [89].

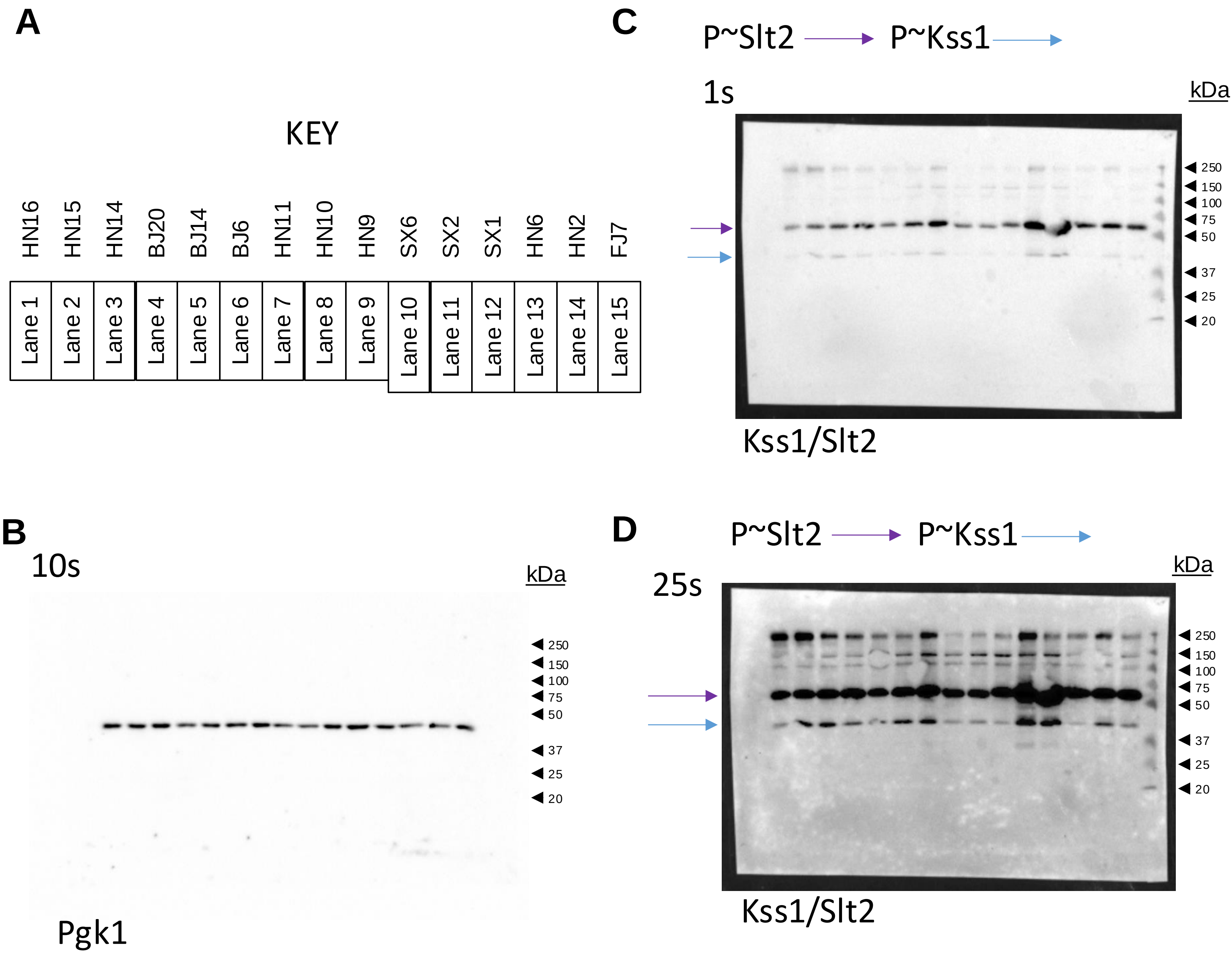

Figure S2. Full data set of immunoblots for P~Kss1p and P~Slt2p, related to Figure 1. Analysis of wild strains (PC7324-PC7338) by immunoblot analysis. Wild strains were grown in 5 mL YPGAL medium for 6 hr. Cell extracts were probed with antibodies to detect phosphorylated Kss1p (P~Kss1p) as a readout of fMAPK pathway activity, P~Slt2p as a readout of the PKC pathway, and Pgk1p as a control for protein levels. **A)** Key for loading order in panels B-D. **B)** Pgk1 Immunoblot after 10s of exposure. Black arrows, ladder. Protein ladder estimated from ladder in Panel C. **C)** P~Kss1p/P~Slt2p immunoblot after 1s of exposure. Purple arrow, P~Slt2p; blue arrow, P~Kss1p; black arrows, ladder. **D)** Same image as panel C, except 25s exposure time.

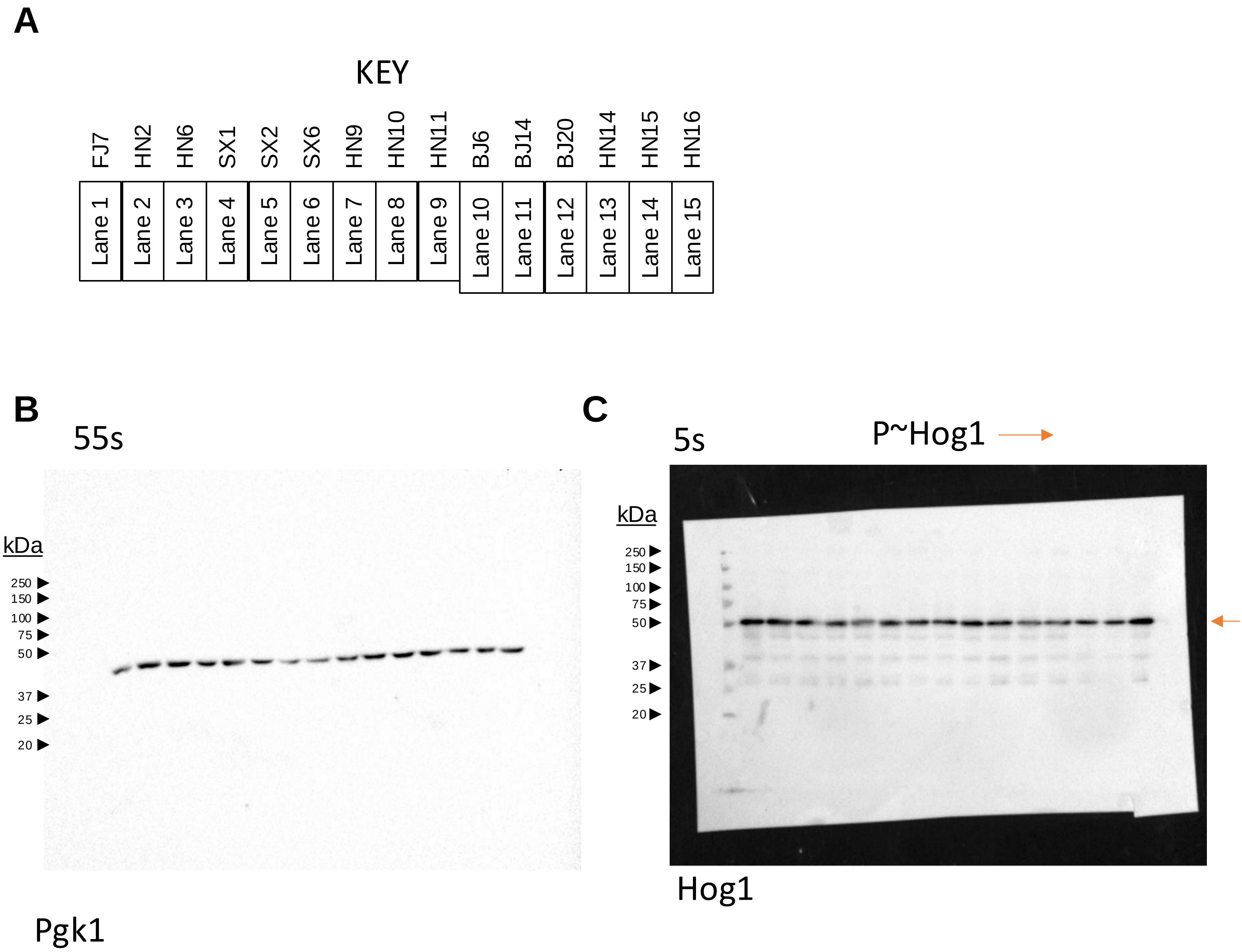

Figure S3. Full data set of immunoblots for P~Hog1p, related to Figure 1. Analysis of wild strains (PC7324-PC7338) by immunoblot analysis. Wild strains were grown in 5 mL YPD+0.5M KCl medium for 5 min. Cell extracts were probed with antibodies to detect phosphorylated Hog1p (P~Hog1p) as a readout of HOG pathway activity and Pgk1p as a control for protein levels. **A)** Key for loading order in panels B-C. **B)** Pgk1 Immunoblot after 55s of exposure. Black arrows, ladder. Protein ladder estimated from ladder in Panel C. **C)** P~Hog1p immunoblot after 5s of exposure. Orange arrow, P~Hog1p.

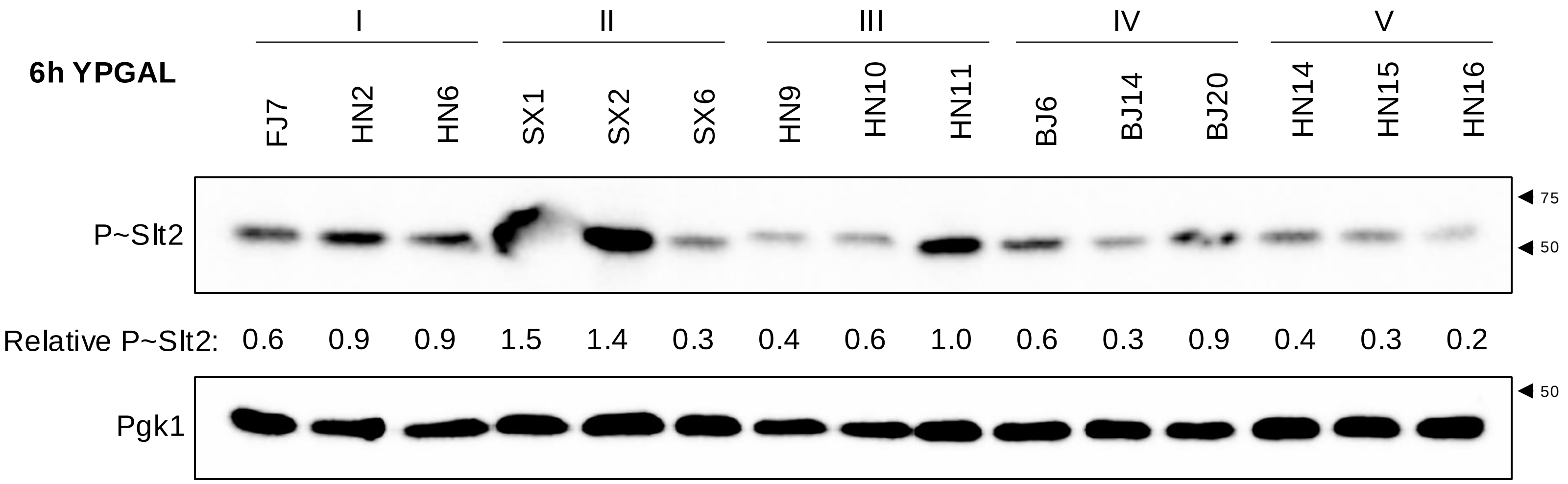

Figure S4. Yeast show variation in P~Slt2p levels, related to Figure 1. Immunoblot analysis of P~Slt2p in wild strains (PC7324-PC7338) after growing cells in 5 mL YPGAL medium for 6 hr. Cell extracts were probed with antibodies to detect phosphorylated Slt2p (P~Slt2p) as a readout of PKC pathway activity and Pgk1p as a control for protein levels. Numbers refer to the ratio of P~Slt2p to Pgk1p with HN11 values set to 1. Full blots in *Fig S2*. Black arrows, molecular markers (kDa).

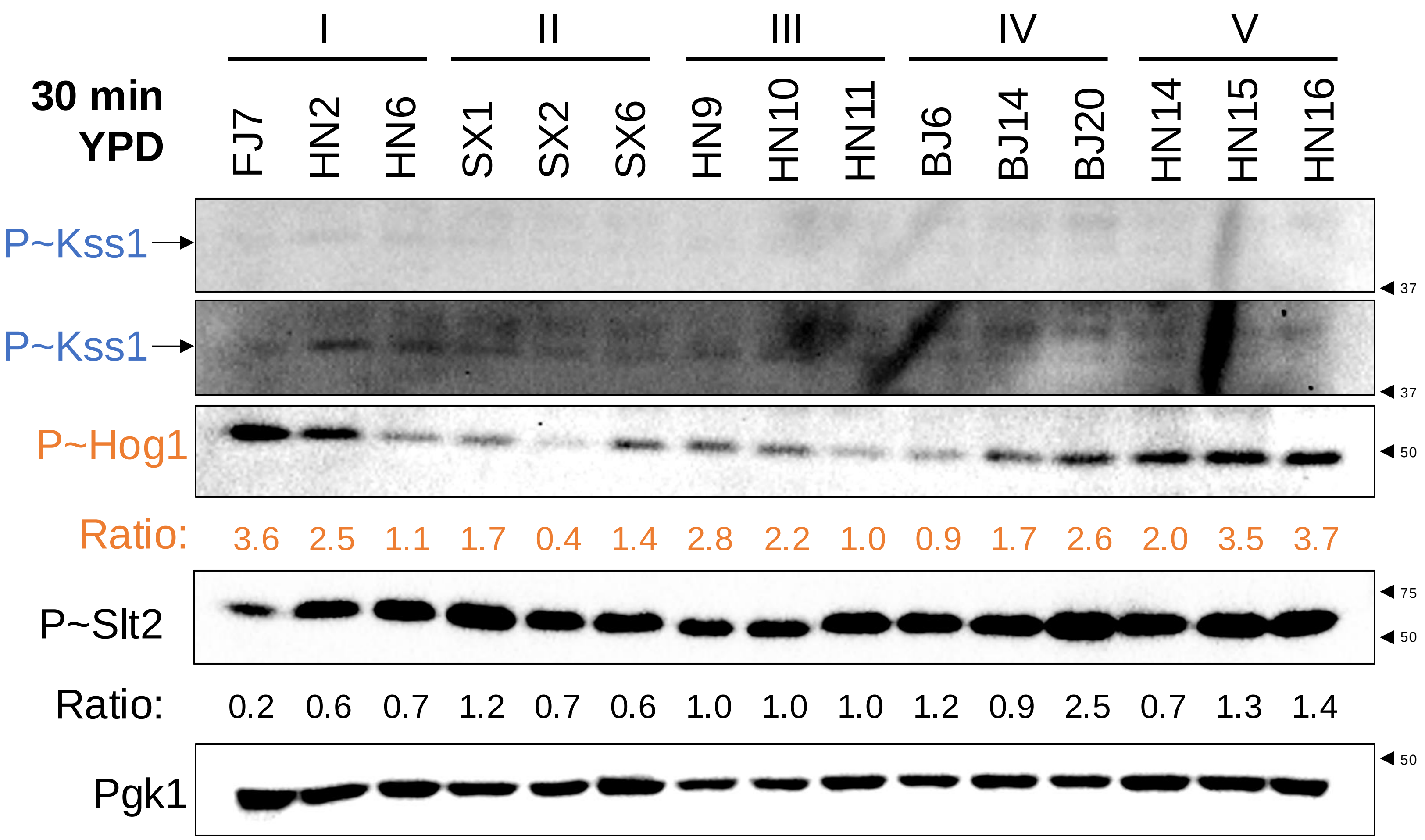

Figure S5. Immunoblots of basal MAPK pathway activity, related to Figure 1. Immunoblot analysis of wild strains (PC7324-PC7338) after growing cells in 5 mL YPD medium for 30 min. Cell extracts were probed with antibodies to detect phosphorylated Kss1p and Slt2p (P~Kss1p and P~Slt2p), phosphorylated Hog1p (P~Hog1p), or Pgk1p as a control for protein levels. Numbers refer to the ratio of the indicated P~kinase to Pgk1p with HN11 values set to 1. The two P~Kss1 images are the same except the lower image has a higher contrast. Full blots in *Fig S6*. Black arrows, molecular markers (kDa).

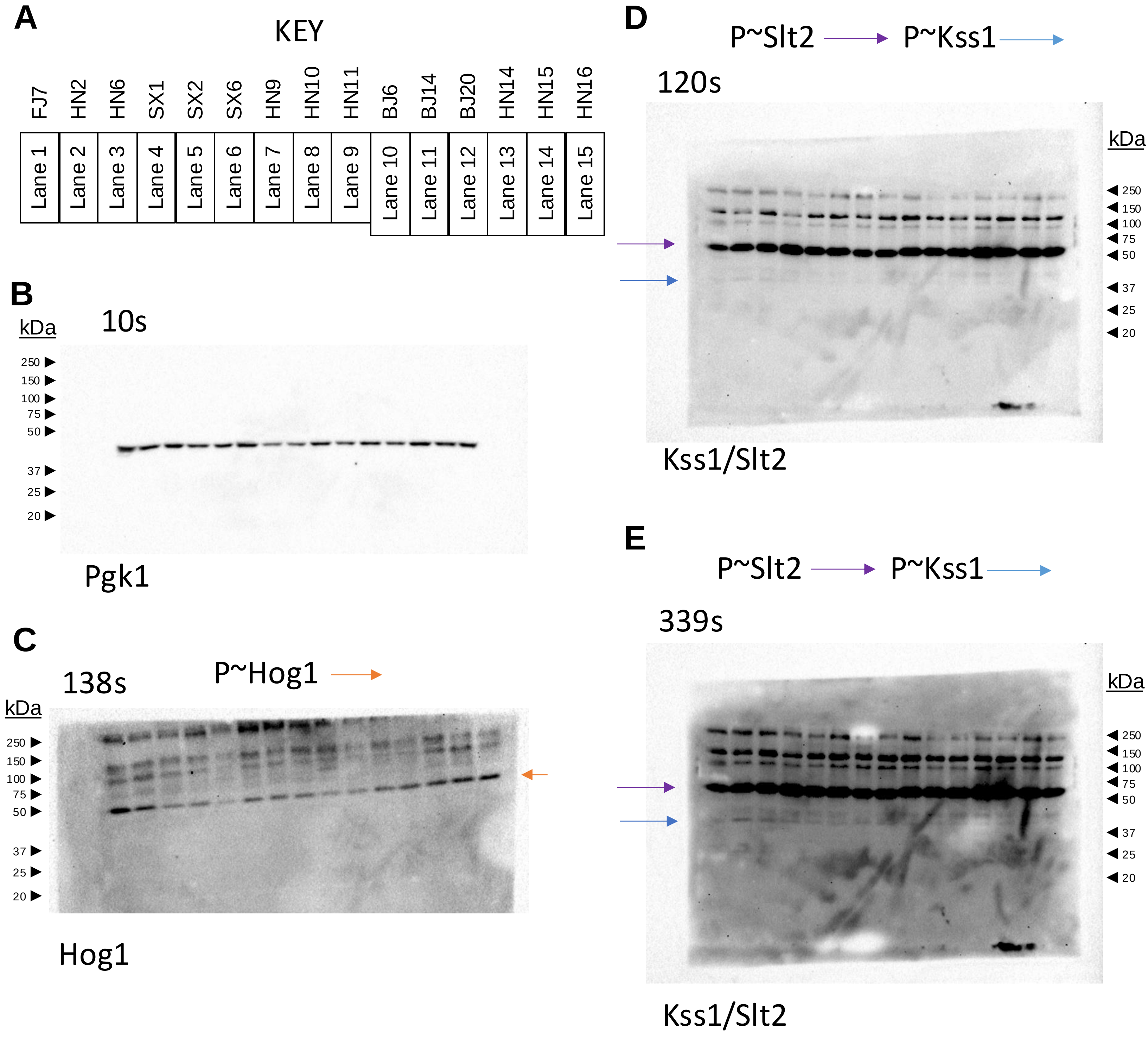

Figure S6. Full data set of immunoblots for basal MAPK pathway activity, related to Figure 1. Analysis of wild strains (PC7324-PC7338) by immunoblot analysis. Wild strains were grown in 5 mL YPD medium for 30 min. Cell extracts were probed with antibodies to detect phosphorylated Kss1p and Slt2p (P~Kss1p and P~Slt2p), phosphorylated Hog1p (P~Hog1p), or Pgk1p as a control for protein levels. **A)** Key for loading order in panels B-E. **B)** Pgk1 Immunoblot after 10s of exposure. Black arrows, ladder. Protein ladder estimated from ladder in Panel C. **C)** P~Hog1p immunoblot after 138s of exposure. Orange arrow, P~Hog1p; black arrows, ladder. Protein ladder estimated from ladder in *Figure S3*. **D)** P~Kss1p/P~Slt2p immunoblot after 120s of exposure. Purple arrow, P~Slt2p; blue arrow, P~Kss1p; black arrows, ladder. Protein ladder estimated from ladder in *Figure S2*. **E)** Same image as panel C, except 339s of exposure.

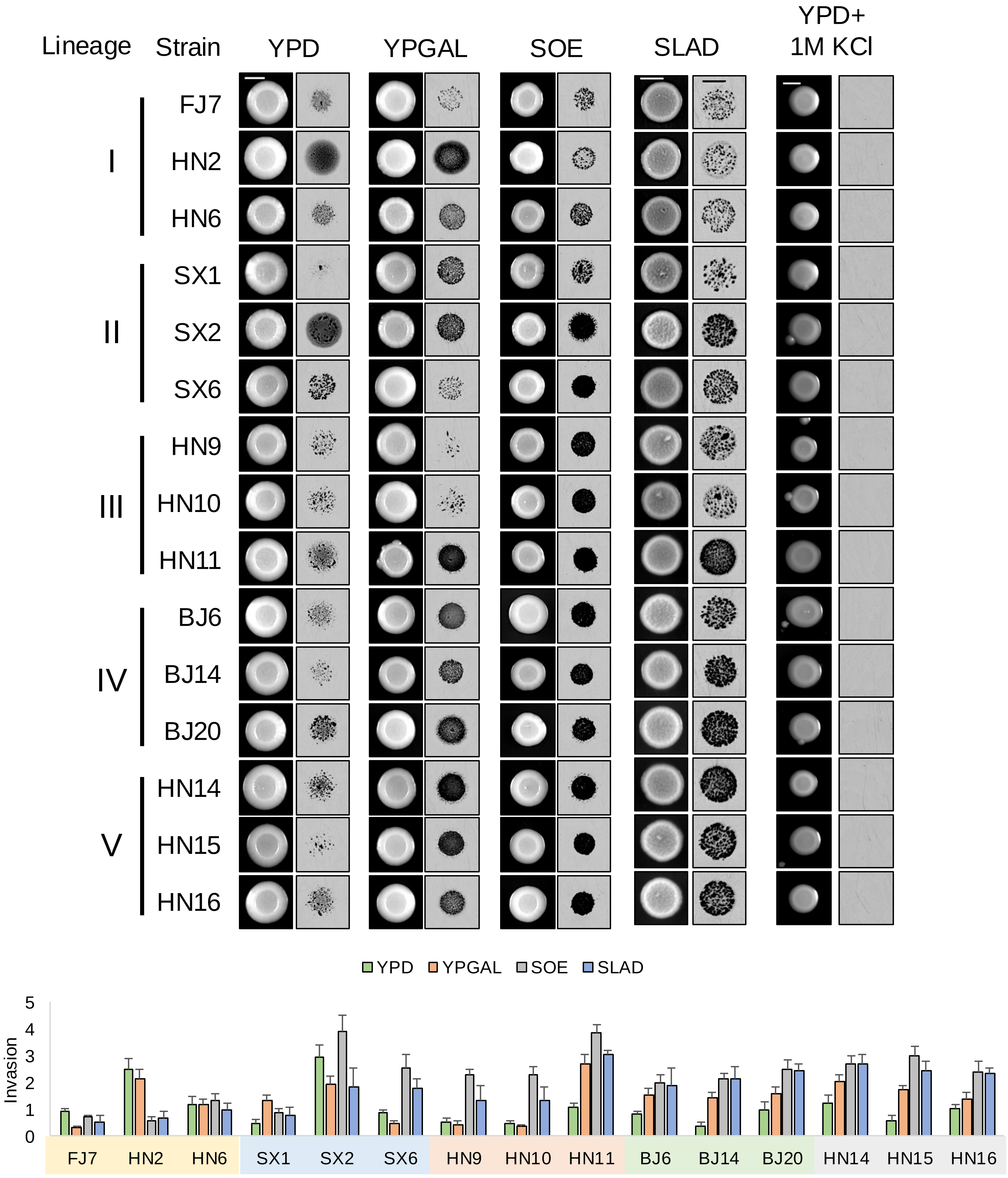

Figure S7. Full data set of the plate-washing assay of wild strains, related to Figure 1. The plate-washing assay of wild strains (PC7324-PC7338) was performed on the indicated media after 4 d of growth. Left columns, before wash images of colonies. Right images, inverted images of invasive scars are displayed. Bar = 0.5 cm. Bar graph, average invasive growth for at least 5 biological replicates ($n \geq 5$) is reported. Error bars represent standard deviation.

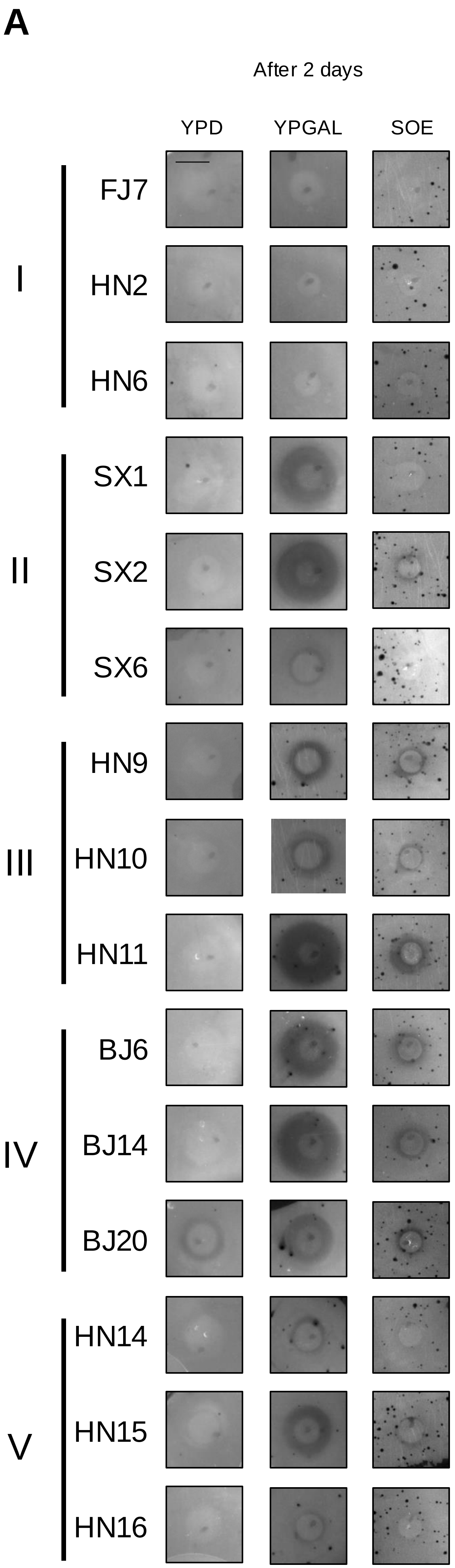

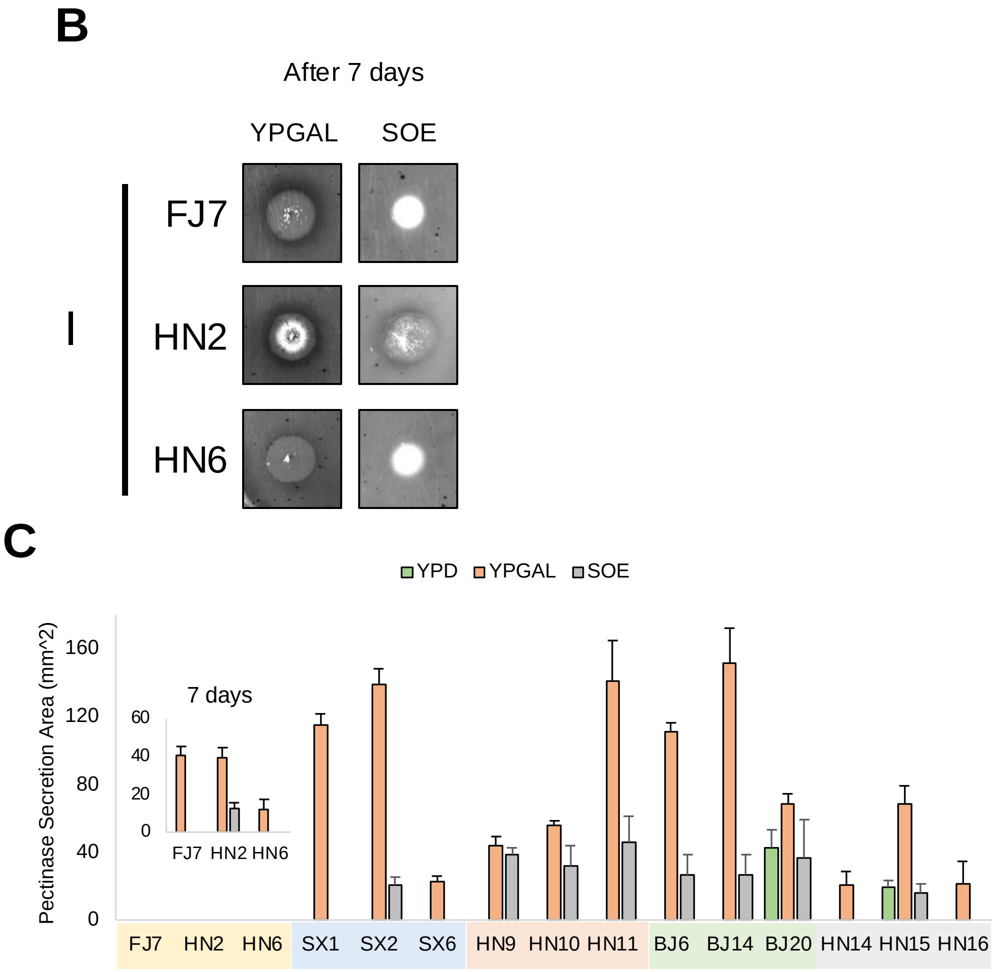

Figure S8. Full data set of the pectinase assay of wild strains, related to Figure 1. Pectinase activity of wild strains (PC7324-PC7338). **A)** Pectinase activity was determined on the indicated environments after 2 d. Bar = 1 cm. **B)** Same as panel A, except after 7 d. **C)** Quantitation of pectinase activity for panels A and B. The average pectinase activity for at least 3 biological replicates ($n \geq 3$) is reported with error bars representing the standard deviation.

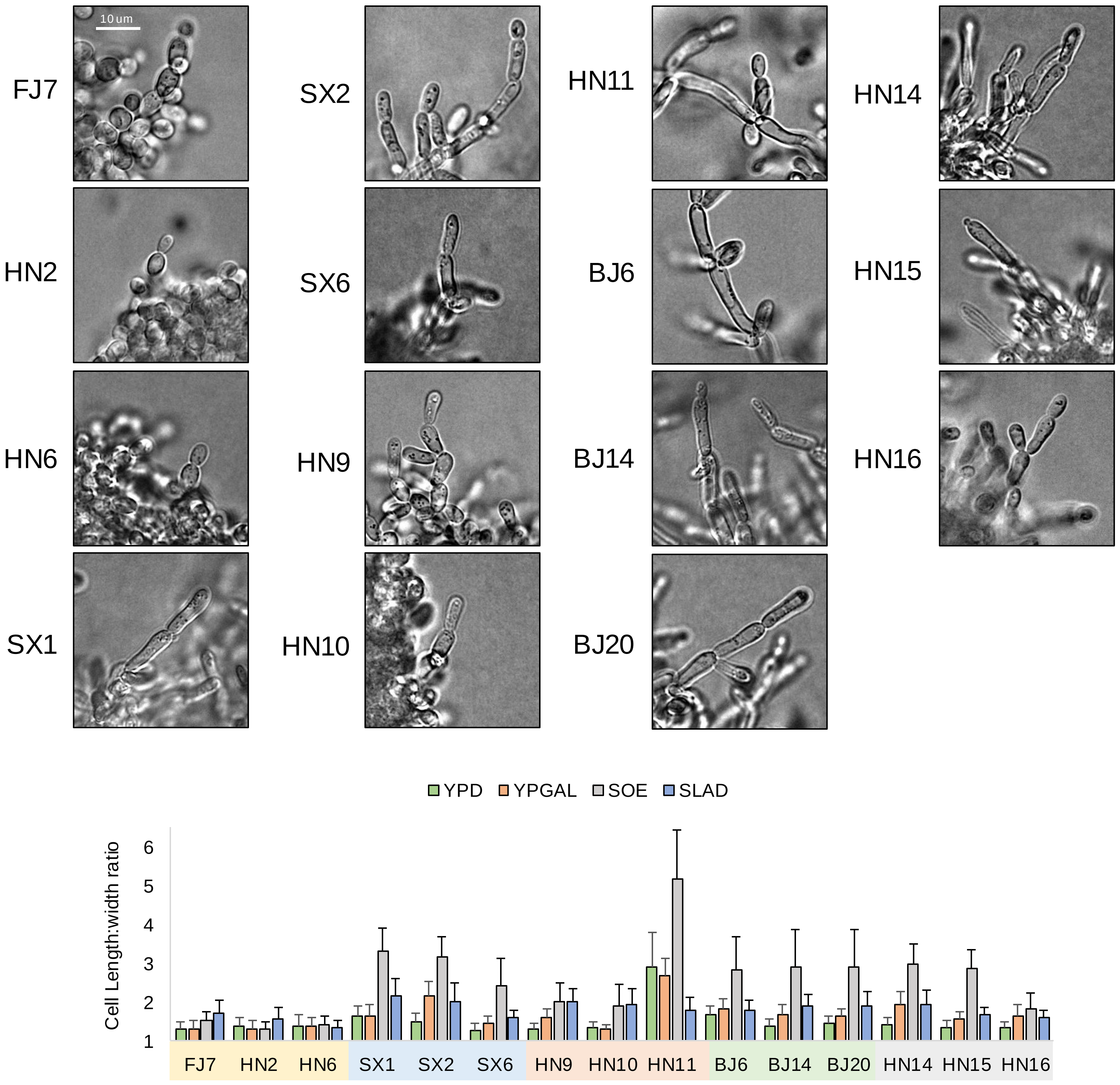

Figure S9. Cell elongation assay of wild strains, related to Figure 1. Cell elongation imaged and quantified for the wild strains (PC7324-PC7338). Cells were spotted onto the indicated medium and grown for 3 d. After, cells were washed off of the surface and cells within the invasive scar were observed by microscopy at 100X magnification. A representative image is displayed for each strain on SOE medium. Bar = 10 um. Bar graph, the average cell length-to-width ratio of at least 30 cells (n $\geq$ 30). Error bars represent the standard deviation.

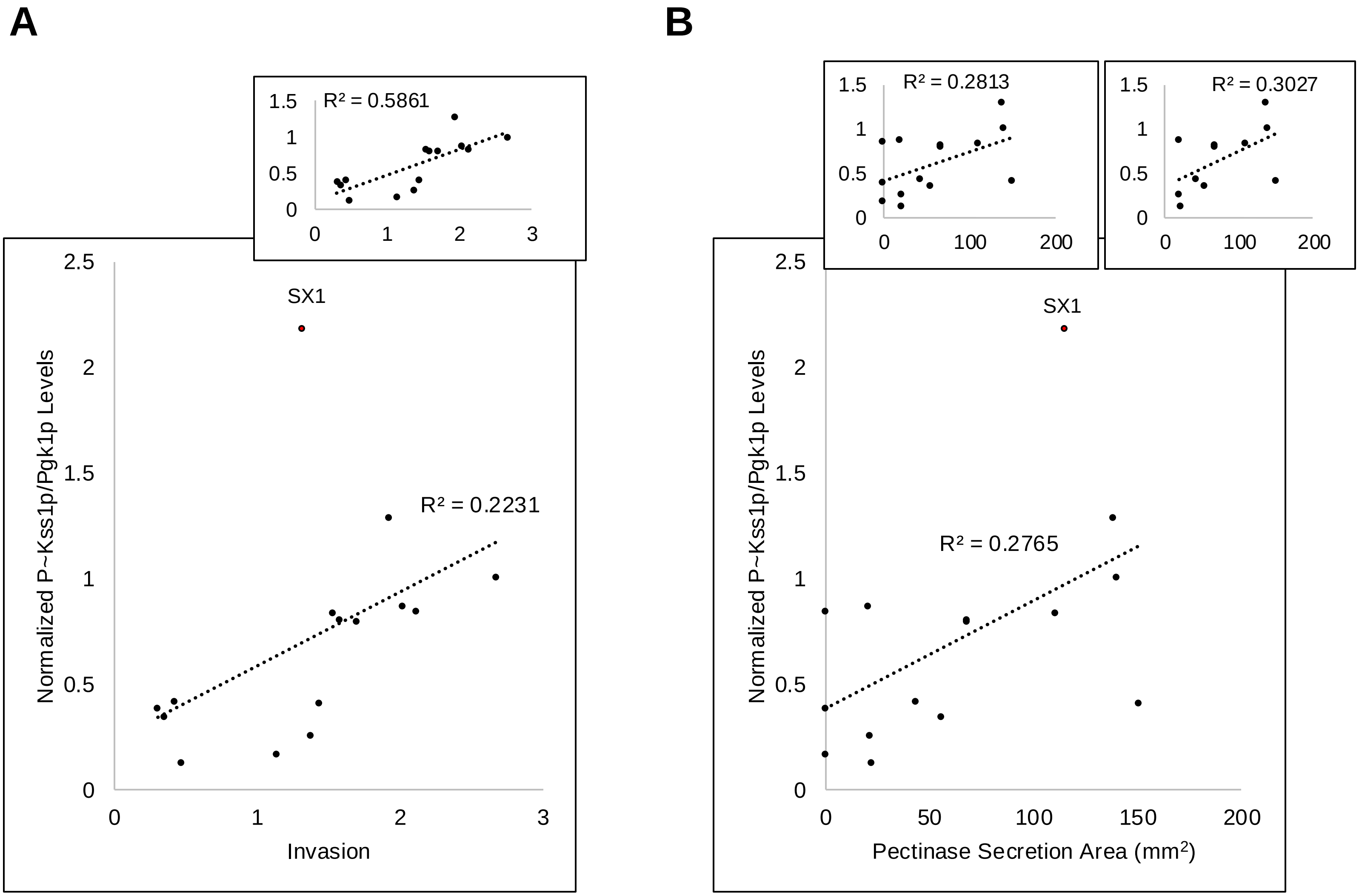

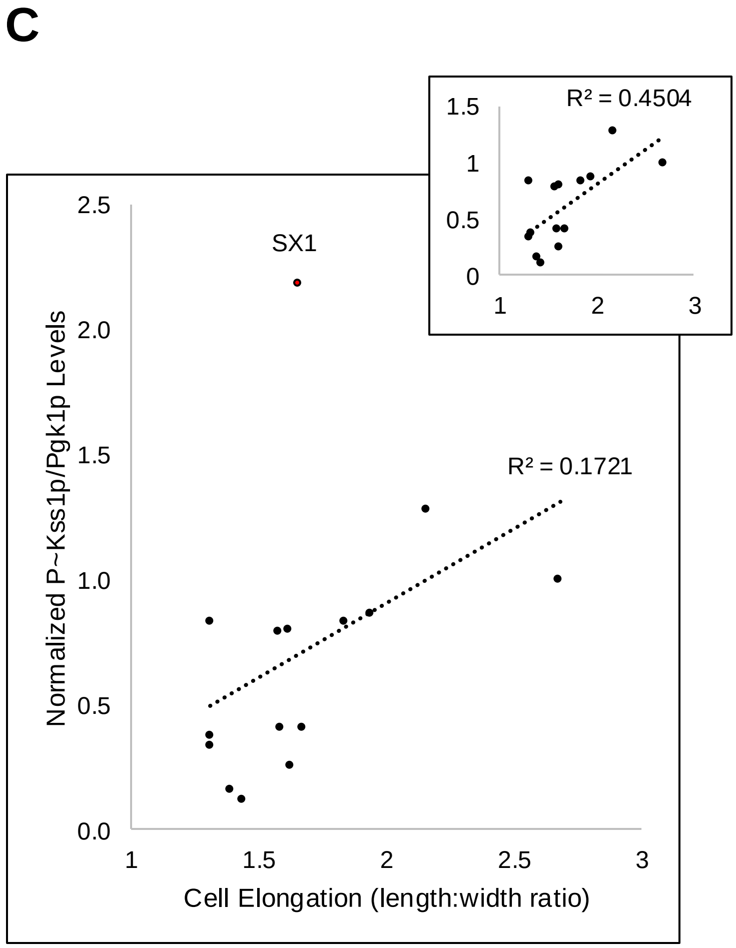

Figure S10. Correlation of fMAPK pathway activity to filamentous growth, related to Figure 1. Correlation analysis of wild strains (PC7324-PC7338). Normalized P~Kss1p/Pgk1 levels, invasion values, and pectinase activity values are the same as **Fig 1B**. Normalized cell elongation values are from *Fig S9*. **A)** Normalized P~Kss1p/Pgk1 levels versus invasion shows R^2 = 0.2231 due to SX1 (red dot) being an outlier. Inset, calculated without SX1, R^2 = 0.5861. **B)** Normalized P~Kss1p/Pgk1 levels versus pectinase activity shows R^2 = 0.2765 due to SX1 (red dot) being an outlier. Inset, left, calculated without SX1, R^2 = 0.2813. Inset, right, calculated without SX1 or Lineage I, R^2 = 0.3027. **C)** Normalized P~Kss1p/Pgk1 levels versus cell elongation (length:width ratios) shows R^2 = 0.1721 due to SX1 (red dot) being an outlier. Inset, calculated without SX1, R^2 = 0.4504.

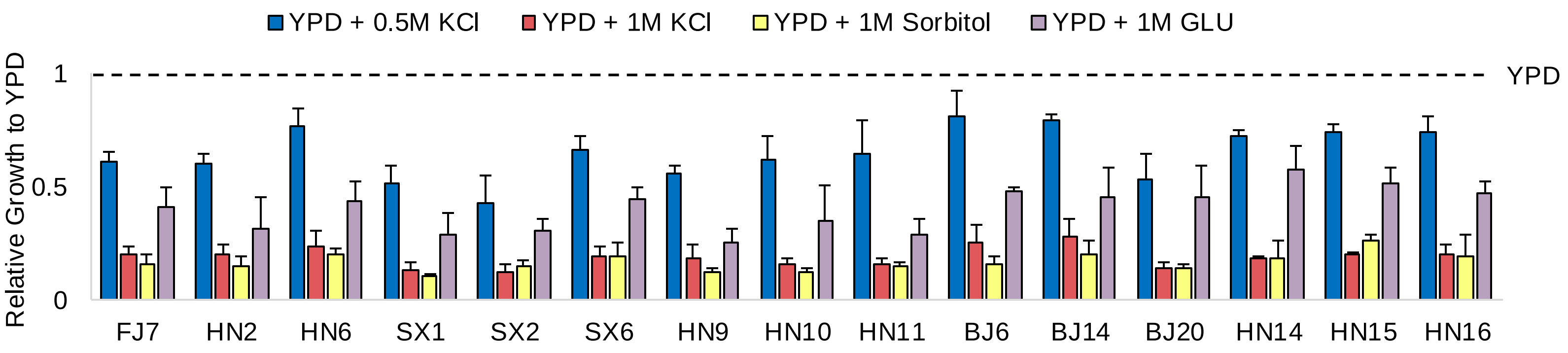

Figure S11. Full dataset of osmolarity tolerance of the wild strains, related to Figure 1. Osmolarity tolerance was determined for the wild strains (PC7324-PC7338). Cells were grown in YPD medium and YPD medium with an indicated osmolyte. Growth in the presence of an osmolyte was quantified relative to growth in YPD medium, with growth in YPD medium set to 1. Three biological replicates (n = 3) of the final OD_{600} after 16h were measured and the averages are reported with error bars representing standard deviation.

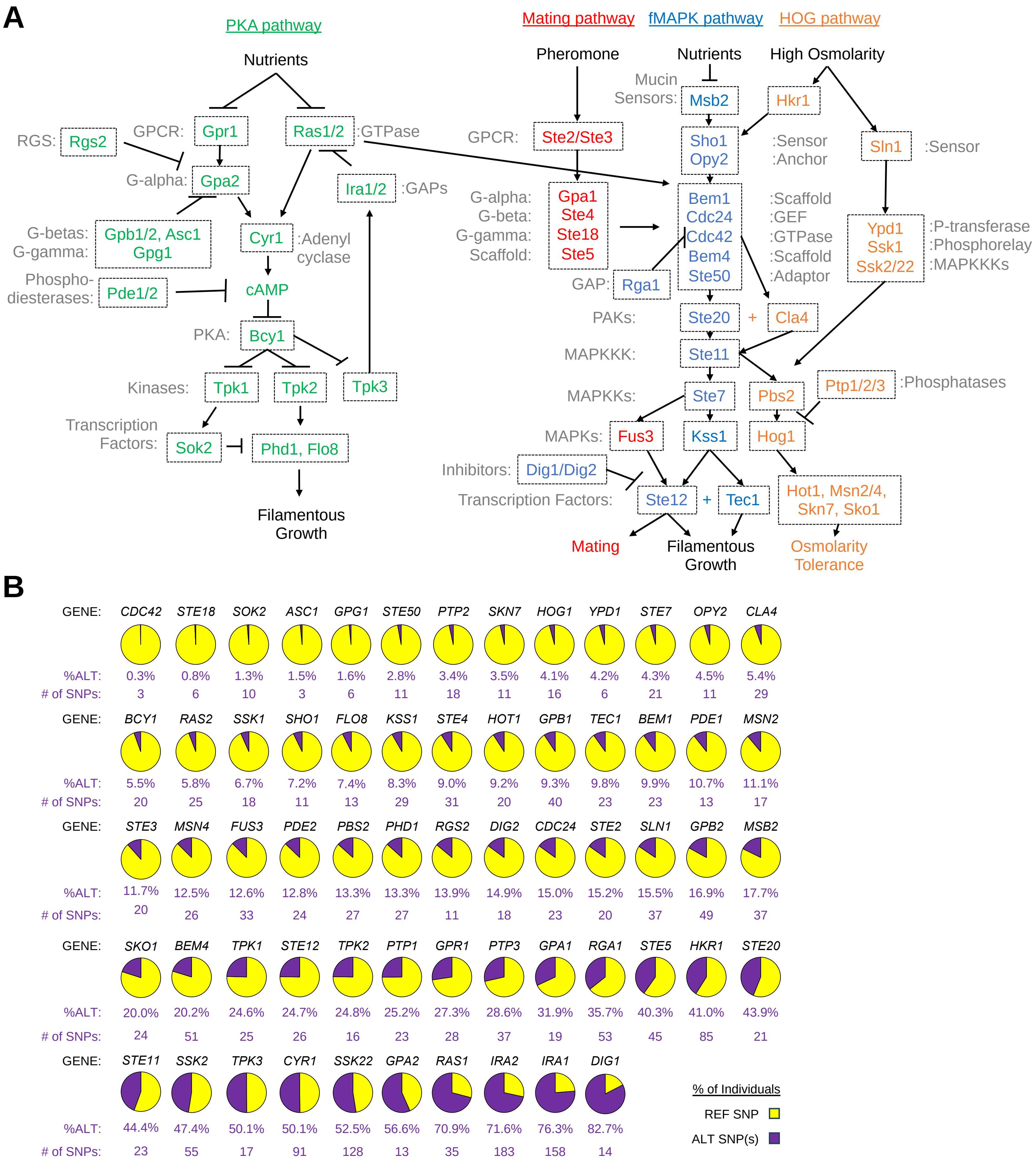

Figure S12. Extended data for the analysis of SNPs within signaling genes, related to Figure 2. A) Model of all 62 genes tested from the fMAPK, Mating, HOG, and PKA pathways. **B)** % alternative SNP analysis for each gene. Pie graphs, the % of individuals (out of 1660) to carry an alternative SNP (purple) compared to no alternative SNP (i.e. reference allele, yellow) was determined. % ALT, the % of individuals that carry an alternative SNP within the indicated gene. # of SNPs, the total number of different SNPs found across the collection for an indicated gene.

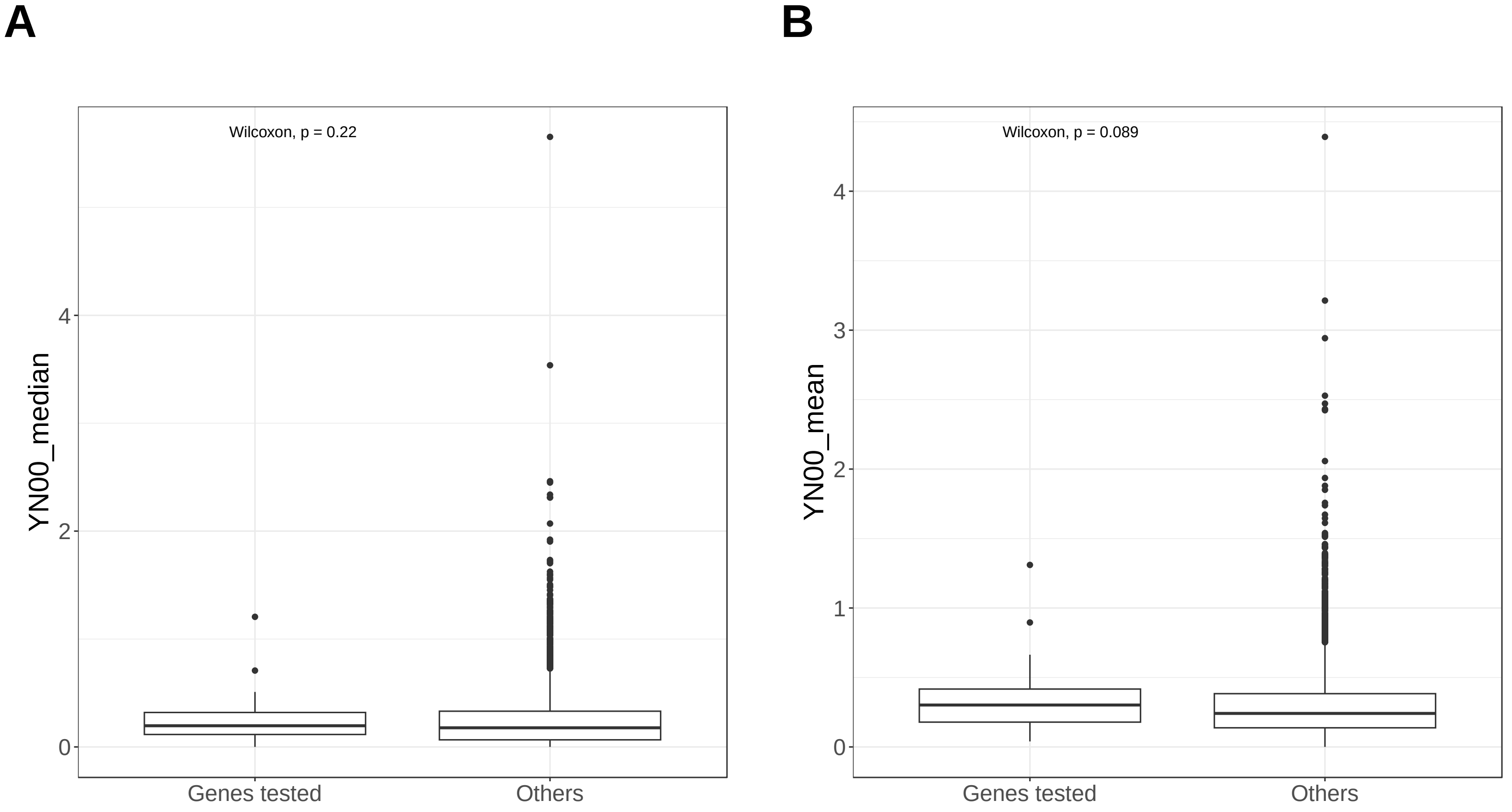

Figure S13. Selection tests comparing signaling genes to the genomic background, related to Figure 2. Box plots for the distribution of dN/dS values for signaling genes of interest (left, Genes tested) and the remaining genes in the genome (right, Others). **A)** The median dN/dS value. **B)** The mean dN/dS value.

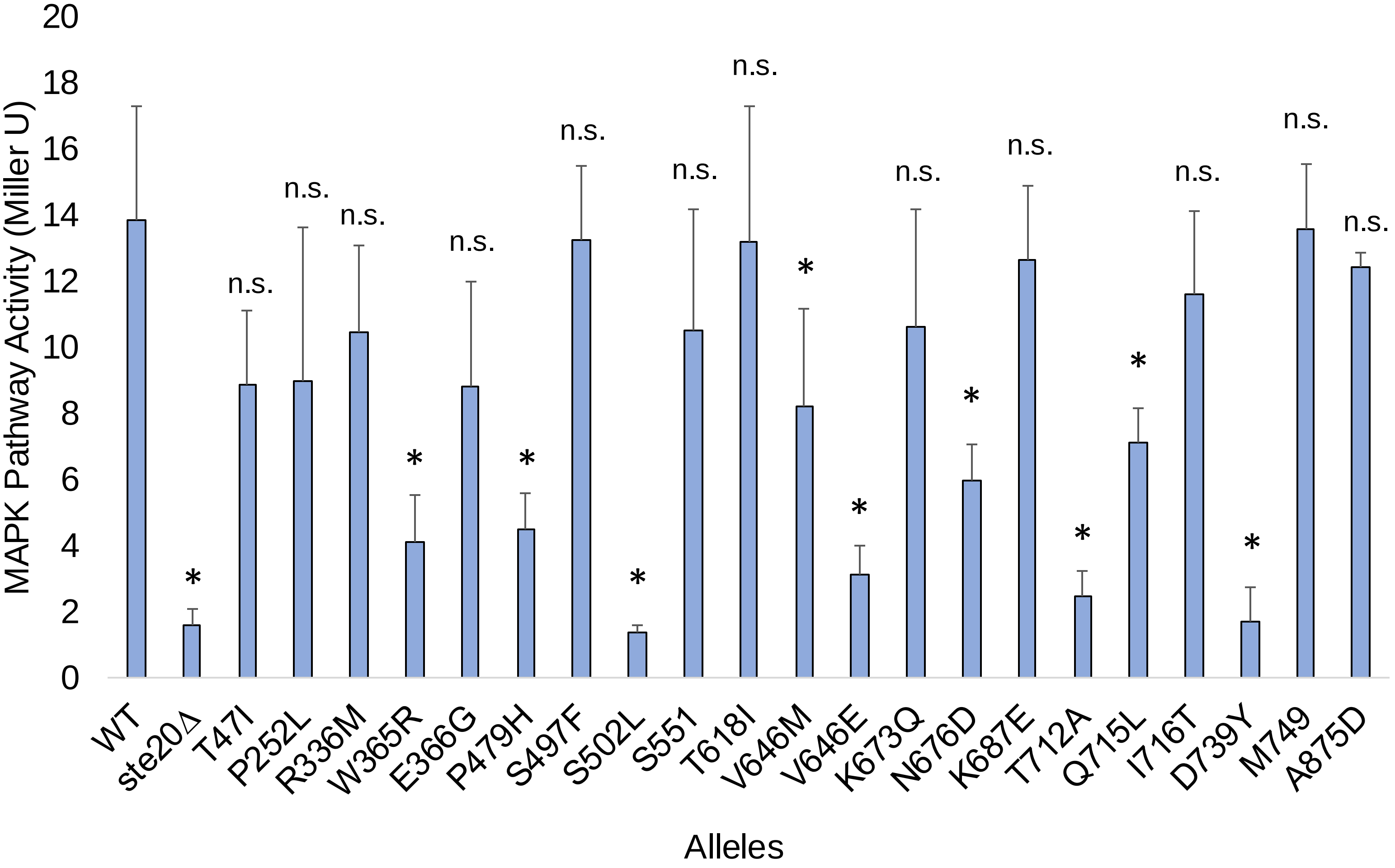

Figure S14. Full dataset of the ß-galactosidase assay for the *STE20* alleles, related to Figure 3. ß-galactosidase assay measuring MAPK pathway activity. Cells of the *ste20*Δ mutant (PC673) were grown harboring p*STE20-GFP* with no change (WT), harboring p*STE20-GFP* with the indicated amino acid change, or harboring p*RS316* as the control vector. Cells were grown in 2 mL YPD for 17 h before harvesting. Average relative fMAPK pathway activity for at least 3 biological replicates (n $\geq$ 3) is reported, with WT values set to 1. Error bars represent standard deviation. Asterisk, p-value < 0.05 by Student's t-test compared to wild type. n.s. means not significant for p-value > 0.05.

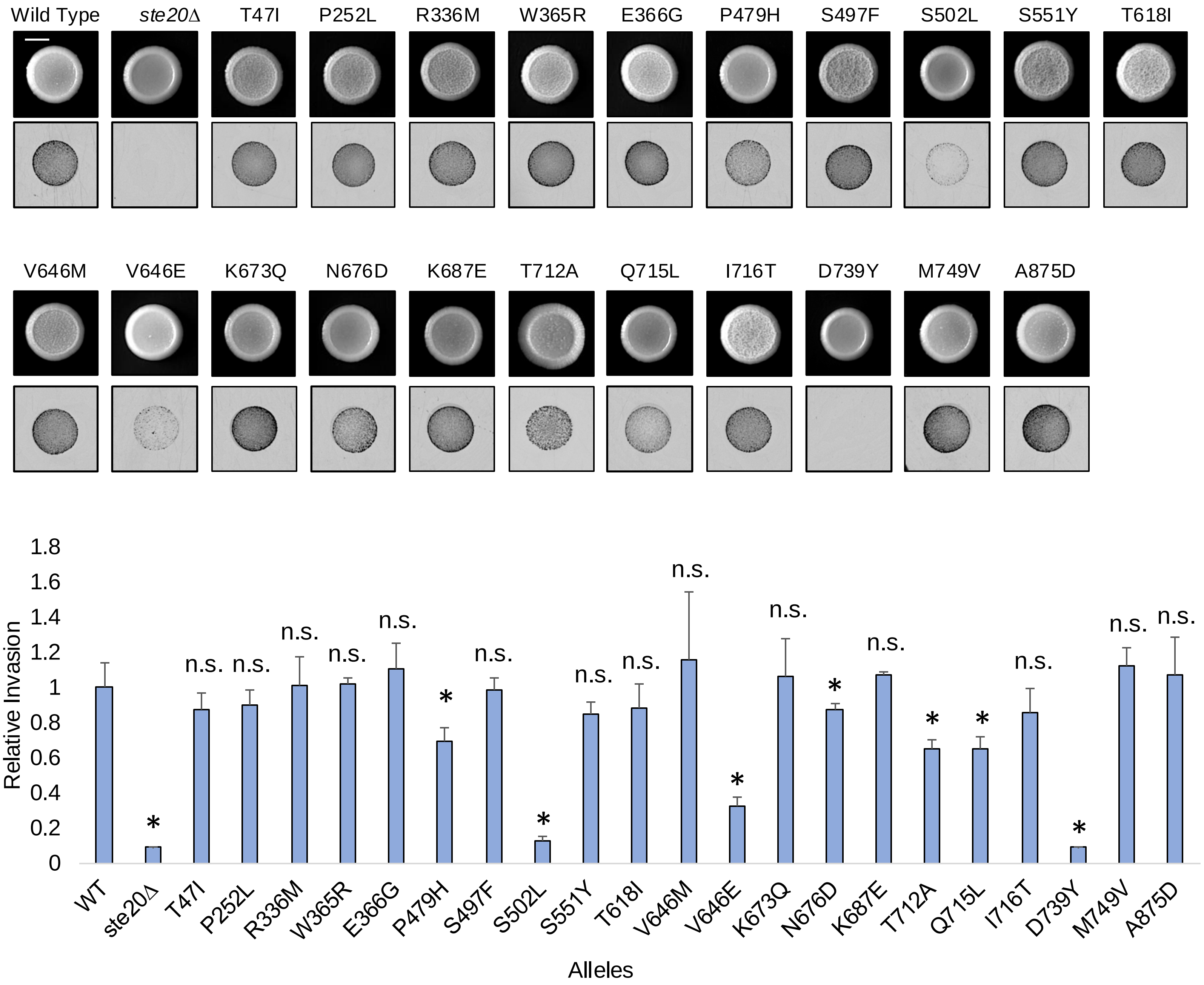

Figure S15. Full dataset of the plate-washing assay of the *STE20* alleles, related to Figure 3. Cells of the *ste20*Δ mutant (PC673) were grown harboring p*STE20-GFP* with no change (WT), harboring p*STE20-GFP* with the indicated amino acid change, or harboring p*RS316* as the control vector. The plate-washing assay was performed after 3 d on YPD medium. Top row, before wash images, bar = 0.5 cm. bottom row, inverted after wash images of invasive scar. Bar graph, represents the average relative invasive growth across 3 biological replicates (n = 3), with WT values set to 1. Error bars represent the standard deviation. Asterisk, p-value < 0.05 by Student's t-test compared to wild type. n.s. means not significant for p-value > 0.05.

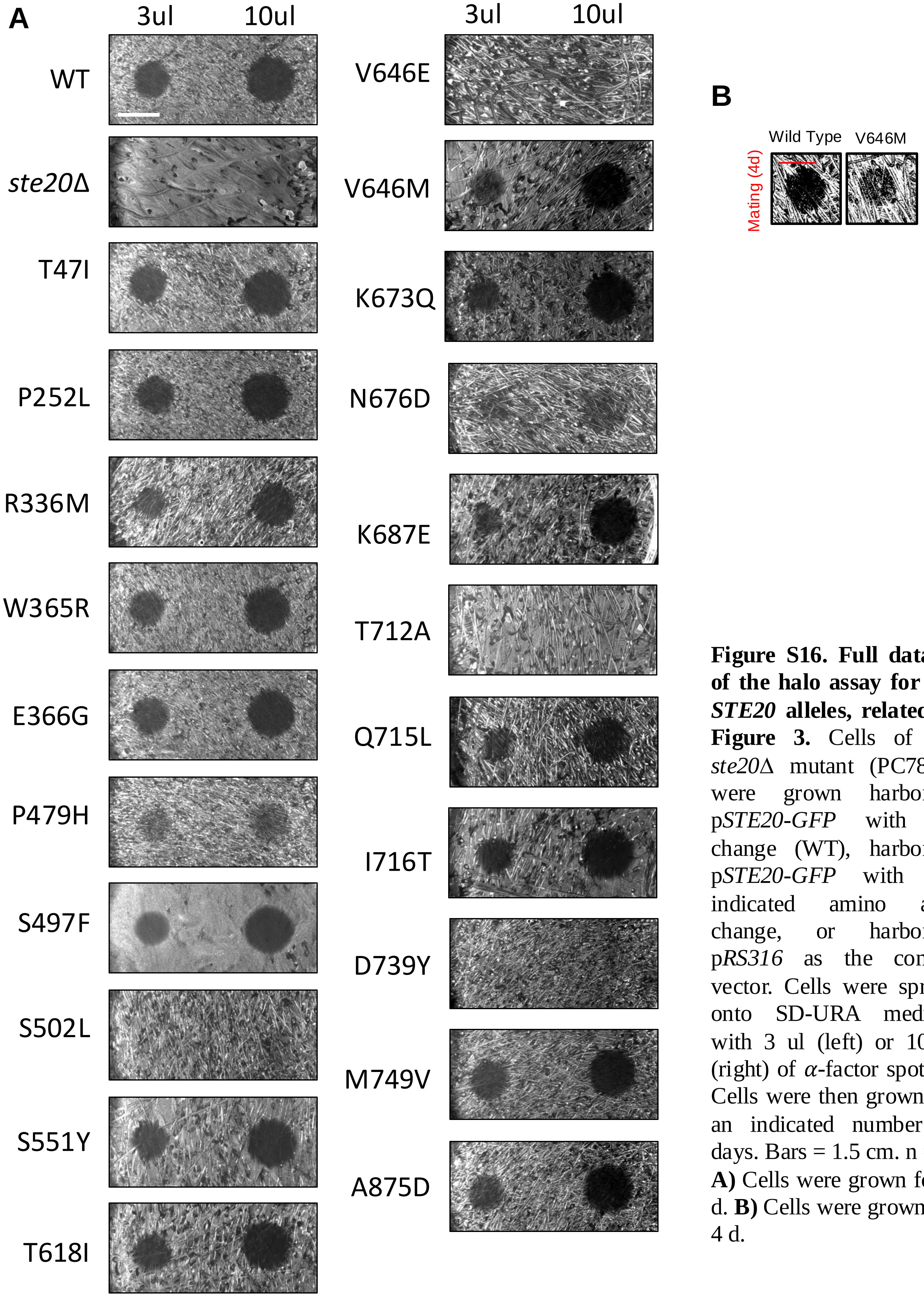

Figure S16. Full dataset of the halo assay for the *STE20* alleles, related to Figure 3. Cells of the *ste20*Δ mutant (PC7871) were grown harboring p*STE20-GFP* with no change (WT), harboring p*STE20-GFP* with the indicated amino acid change, or harboring p*RS316* as the control vector. Cells were spread onto SD-URA medium with 3 ul (left) or 10 ul (right) of α-factor spotted. Cells were then grown for an indicated number of days. Bars = 1.5 cm. n = 1. **A)** Cells were grown for 1 d. **B)** Cells were grown for 4 d.

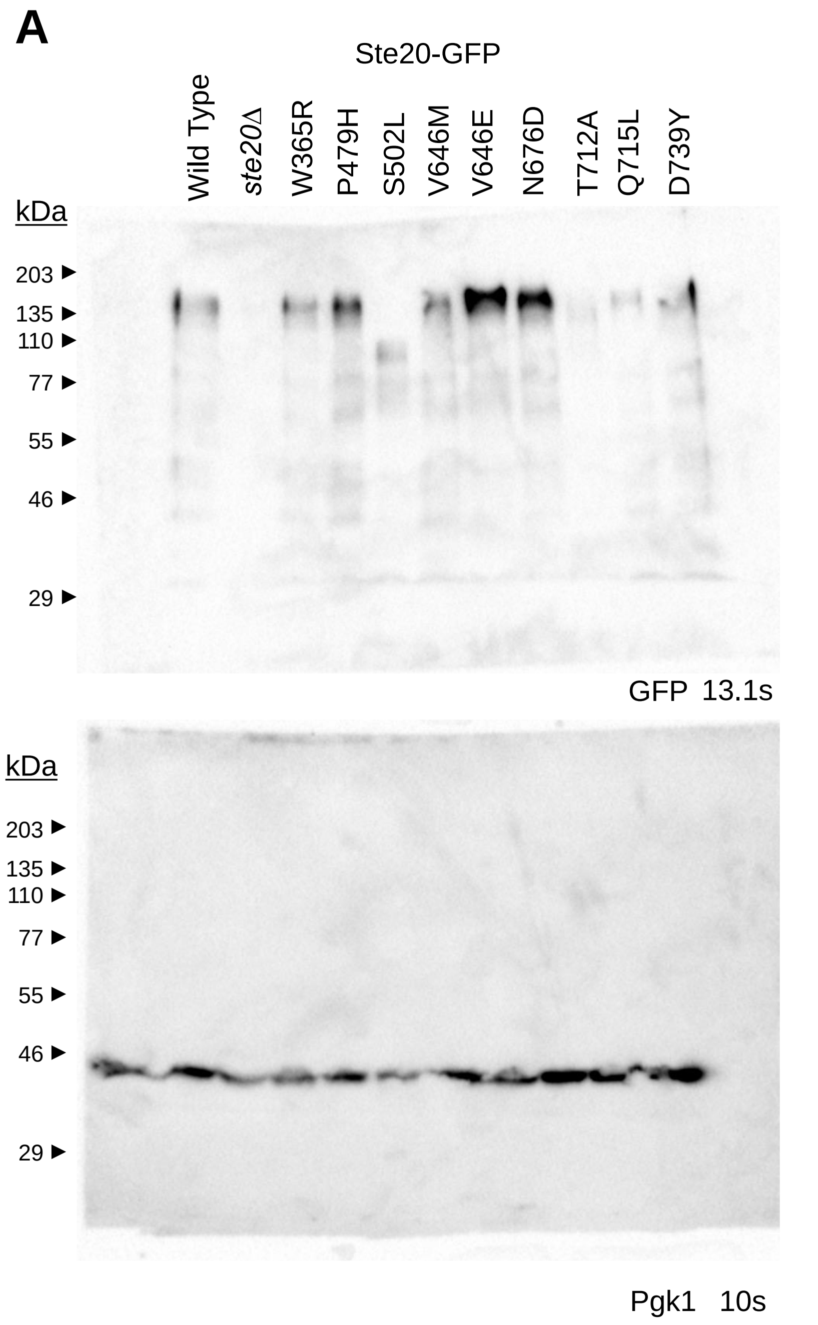

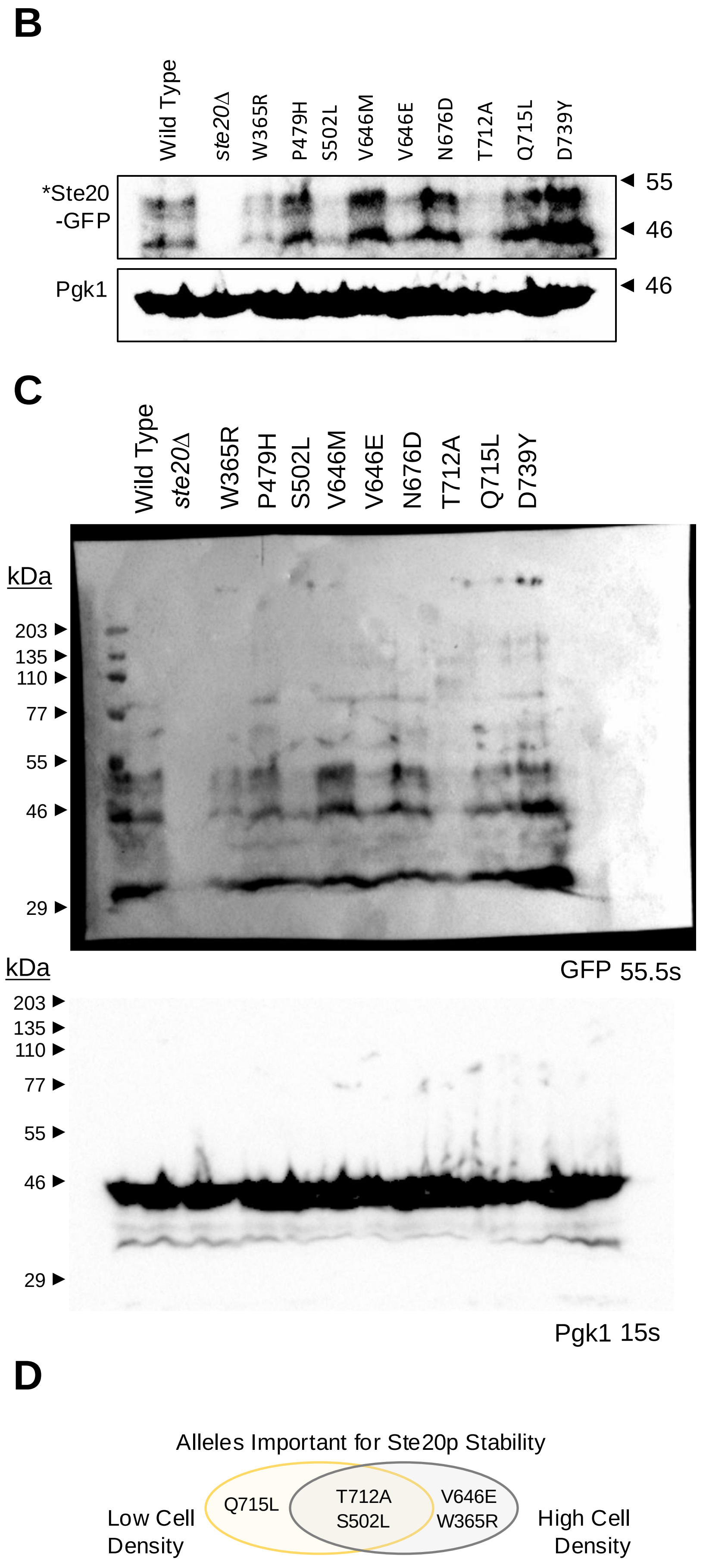

Figure S17. Full dataset for immunoblots of GFP for the *STE20* alleles, related to Figure 3. Immunoblot analyses of Ste20p-GFP levels. Cells of the *ste20*Δ mutant (PC7871) were grown harboring p*STE20-GFP* with no change (WT), harboring p*STE20-GFP* with the indicated amino acid change, or harboring p*RS316* as the control vector. Cells were grown, harvested, and cell extracts were probed with antibodies to detect GFP (Ste20-GFPp) and Pgk1p as a control for protein levels. **A)** Full immunoblots for **Fig 3F**. Cells were grown at low cell density. Top, immunoblot of anti-GFP after 13.1s of exposure. Bottom, immunoblot of Pgk1p after 10s of exposure. Black arrows, ladder. Protein ladder estimated from ladder in panel C. **B)** Cells were grown at high cell density. *Ste20p-GFP does not represent full length Ste20p, but degradation products of Ste20p. Black arrows, molecular markers (kDa) **C)** Full immunoblots for panel B. Top, immunoblot of anti-GFP after 55.5s of exposure. Full length Ste20-GFPp (between 135 and 203 kDa) and its degradation products can be seen throughout each lane. Black arrows, ladder. Bottom, immunoblot of Pgk1p after 15s of exposure. Black arrows, ladder. Protein ladder estimated from ladder in panel C. **D)** Venn diagram displaying alleles that are important for maintaining Ste20p levels based on data from both the low cell density and high cell density trials. Sites T712 and S502 were critical for stability in both environments, whereas sites Q715, V646, and W365 were only important to one condition.

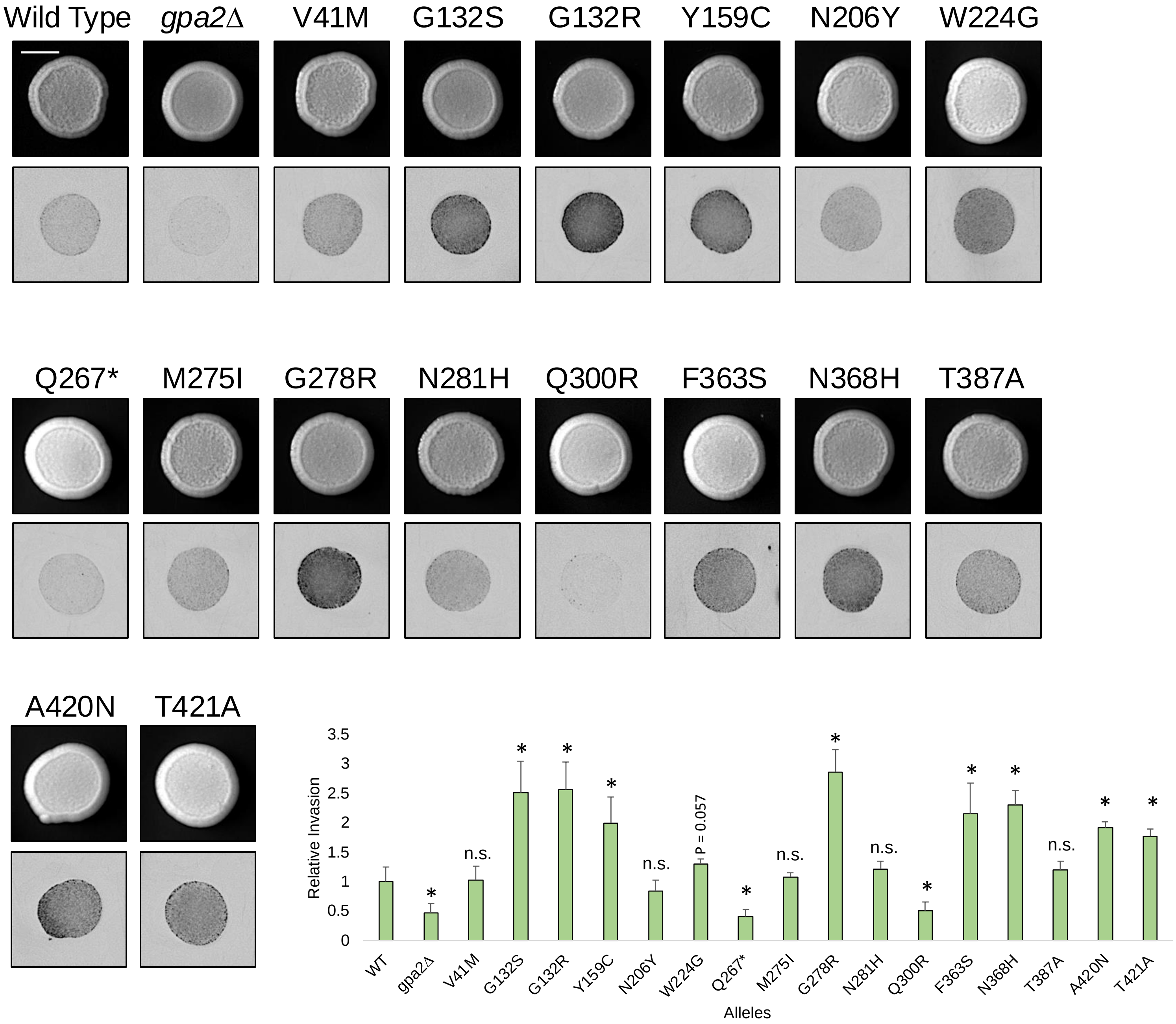

Figure S18. Full dataset of the plate-washing assay of the *GPA2* alleles, related to Figure 4. Cells of the *gpa2*Δ mutant (PC7893) were grown harboring p*GPA2* with no change (WT), harboring p*GPA2* with the indicated amino acid change, or harboring p*RS315* as the control vector. The plate-washing assay was performed after 3 d on YPGAL medium. Top row, before wash images, bar = 0.5 cm. bottom row, inverted after wash images of invasive scar. Bar graph, represents the average relative invasive growth across 3 biological replicates (n = 3), with WT values set to 1. Error bars represent the standard deviation. Asterisk, p-value < 0.05 by Student's t-test compared to wild type. n.s. means not significant for p-value > 0.05.

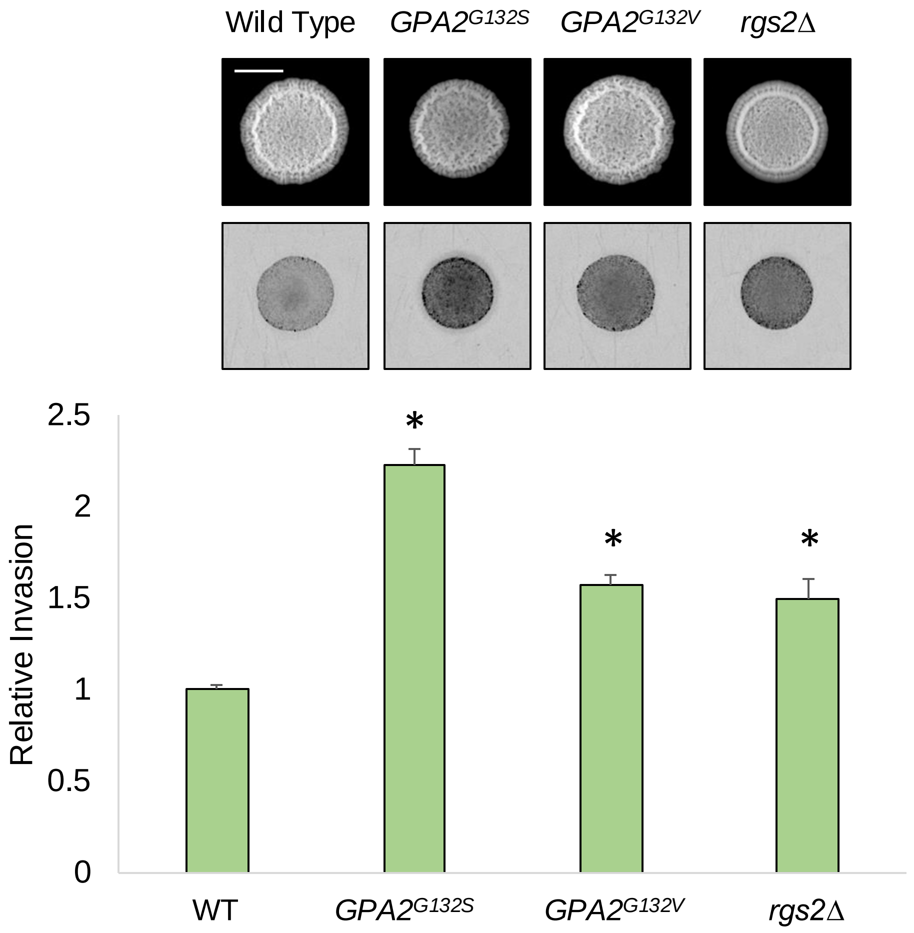

Figure S19. Plate-washing assay of CRISPR-Cas9 generated *GPA2*^{G132S} and *GPA2*^{G132V} alleles, related to Figure 4. The plate-washing assay was performed with wild-type (PC313), *GPA2*^{G132S} (PC7851), *GPA2*^{G132V} (PC7877), and the *rgs2*Δ mutant (PC7876) strains after spotting cells onto YPGAL medium and growing them for 3 d. Top row, before wash images, bar = 0.5 cm. bottom row, inverted after wash images of invasive scar. Bar graph, represents the average relative invasive growth across 3 biological replicates (n = 3), with WT values set to 1. Error bars represent the standard deviation. Asterisk, p-value < 0.05 by Student's t-test compared to wild type.

A

Organism	Gene	G3-Motif	Site
S. cerevisiae	*GPA2*	DXXGQ	Q300
C. elegans	gpa-6	DXXGQ	Q215

B

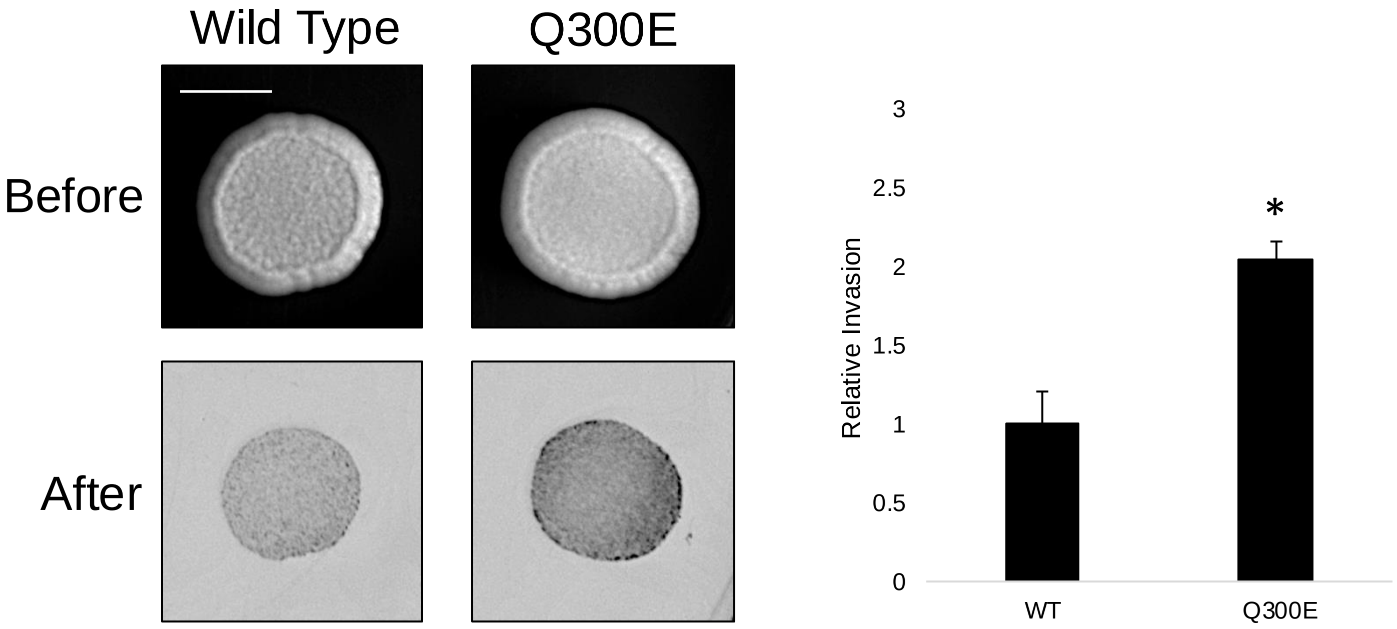

Figure S20. The amino acid change of Q to E at site 215 of *Caenorhabditis elegans* gpa-6 is predicted to be a GOF effect, related to Figure 4. A) Site Q215 in gpa-6 from *C. elegans* corresponds to site Q300 in *GPA2* in *S. cerevisiae*. **B)** Plate-washing assay. Cells of the *gpa2*Δ mutant (PC7893) were grown harboring p*GPA2* with no change (WT) or harboring p*GPA2* with the amino acid change of Q300E. Cells were spotted onto YPGAL medium and grown for 3 d. Top row, before wash images, bar = 0.5 cm. bottom row, inverted after wash images of invasive scar. Bar graph, represents the average relative invasive growth across 3 biological replicates (n = 3), with WT values set to 1. Error bars represent the standard deviation. Asterisk, p-value < 0.05 by Student's t-test compared to wild type.

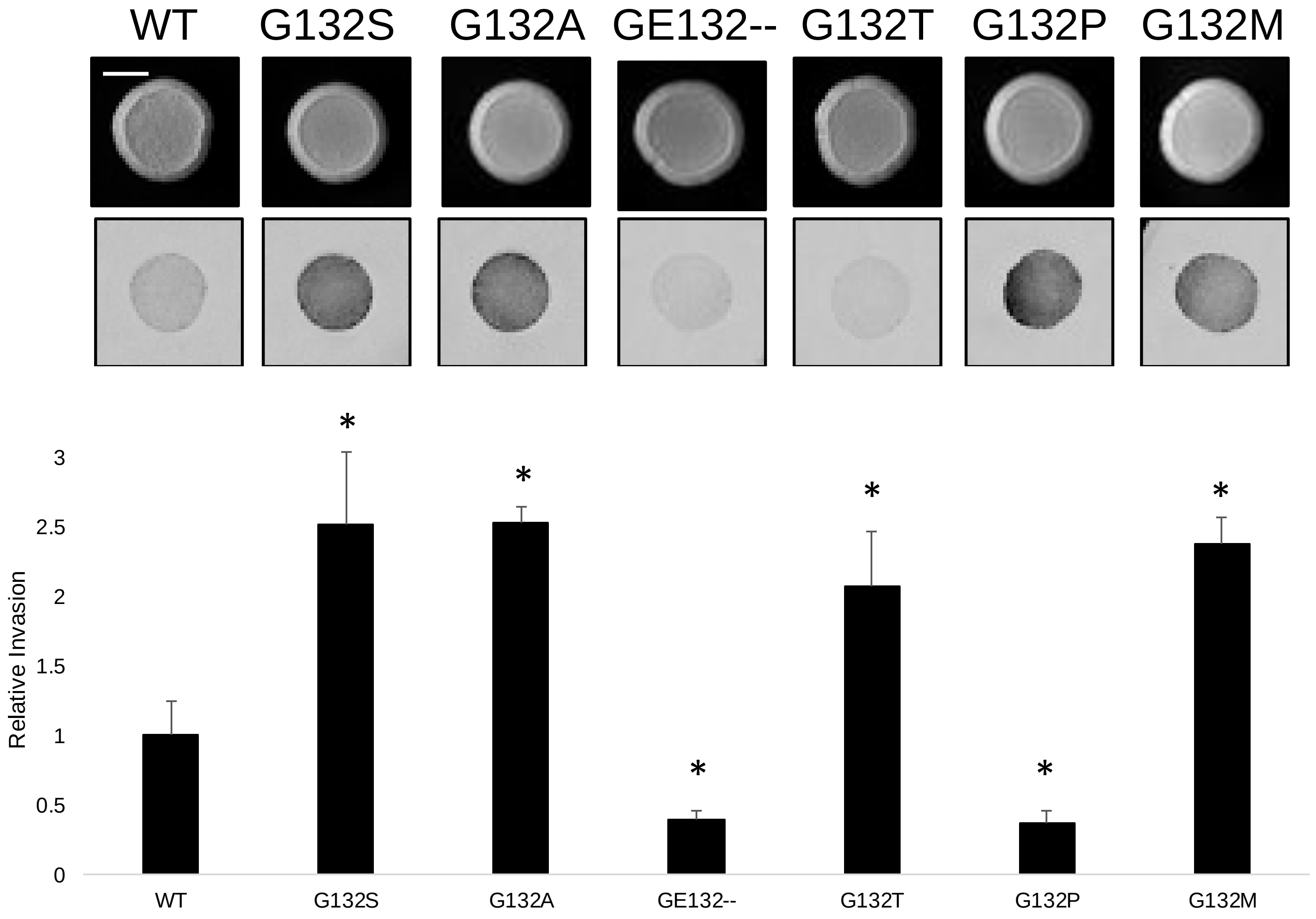

Figure S21. Fixed amino acid changes that occur in Gα-proteins in higher eukaryotes are mostly predicted to have GOF effects, related to Figure 4. Cells of the *gpa2*Δ mutant (PC7893) were grown harboring p*GPA2* with no change (WT), harboring p*GPA2* with the indicated amino acid change, or harboring p*RS315* as the control vector. The plate-washing assay was performed after 3 d on YPGAL medium. Top row, before wash images, bar = 0.5 cm. bottom row, inverted after wash images of invasive scar. Bar graph, represents the average relative invasive growth across 3 biological replicates (n = 3), with WT values set to 1. Error bars represent the standard deviation. Asterisk, p-value < 0.05 by Student's t-test compared to wild type.

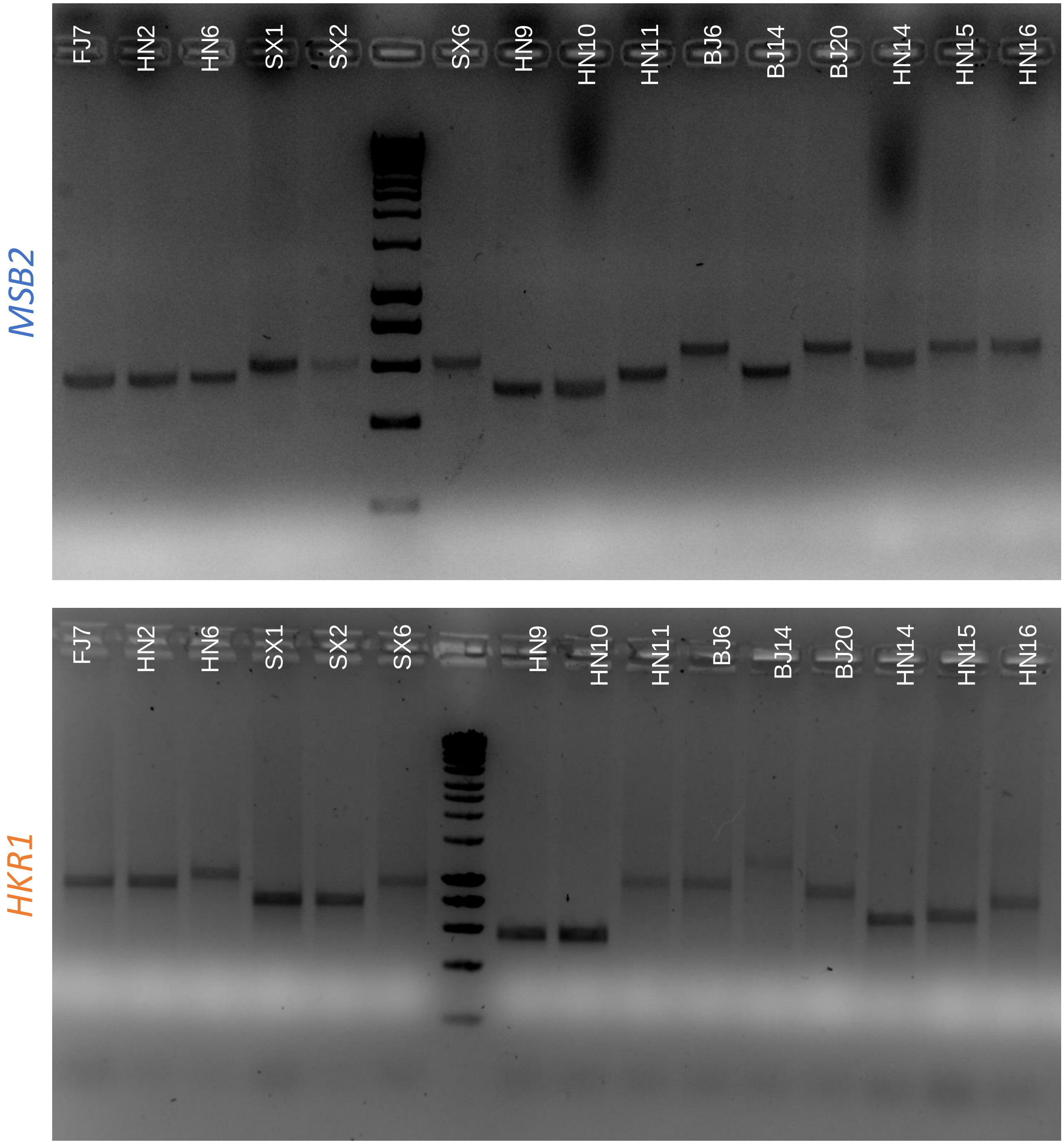

Figure S22. PCR amplification of mucin repeat regions of *MSB2* and *HKR1* for the wild strains, related to Figure 5. Amplification of the repeat region of *MSB2* (top) or *HKR1* (bottom) for wild strains (PC7324-PC7338). *MSB2* Primers capture an additional 326 bp outside of the repeat region. A single repeat in *MSB2* is 51 bp. *HKR1* Primers capture an additional 420 bp outside of the repeat region. A single repeat in *HKR1* is 84 bp. Ladder is 1kb. Numerical values can be found in *Table S1*.

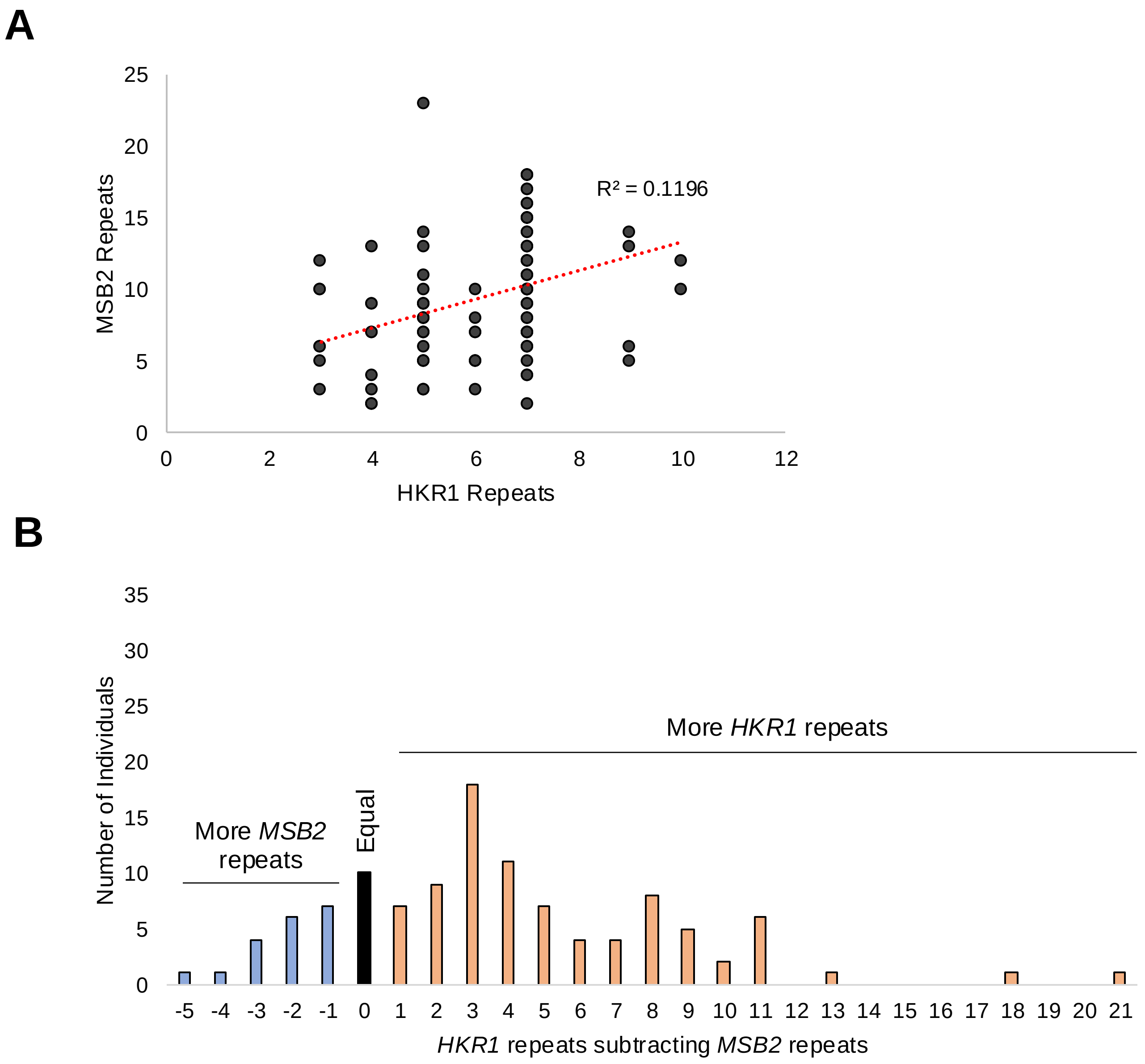

Figure S23. There is no correlation between *MSB2* and *HKR1* repeat numbers, related to Figure 5. Mucin repeat data to perform the following analyses comes from *Table S1*. **A)** Comparison of the number of repeats in *MSB2* versus *HKR1* for each strain. No correlation seen. $R^2 = 0.1196$. **B)** Comparison of the number of repeats in *HKR1* compared to *MSB2* for each strain. Repeat differences were calculated by subtracting the total number of *HKR1* repeats from the total number of *MSB2* repeats for each strain. The results are displayed as a histogram showing the number of individuals that contain more *HKR1* repeats (positive values), the same number of repeats for both mucins (0 value), or more *MSB2* repeats (negative values).

Table S1. Mucin repeat number analysis, related to Figure 5.

Source	Strain	*MSB2* repeat number	*HKR1* repeat number	Notes	Reference
Reference	S288c	7	12		SGD database
PCR only	FJ7	5	7		Duan et al., 2019
	HN2	5	7		Duan et al., 2019
	SX2	6	5		Duan et al., 2019
	SX6	6	7		Duan et al., 2019
	HN9	4	2		Duan et al., 2019
	HN11	5	8		Duan et al., 2019
	BJ14	5	11		Duan et al., 2019
	HN14	6	3		Duan et al., 2019
	HN15	7	4		Duan et al., 2019
Visualization of sequence and PCR	BAG (SX1)	6	5	Sequencing matches PCR method	Duan et al., 2019; O'Donnell et al., 2023
	BAM (HN10)	4	2	Sequencing matches PCR method	Duan et al., 2019; O'Donnell et al., 2023
	BAL (HN6)	5	8	Sequencing matches PCR method	Duan et al., 2019; O'Donnell et al., 2023
	BAK (BJ20)	7	7	Sequencing matches PCR method	Duan et al., 2019; O'Donnell et al., 2023
	BAI (BJ6)	7	8	Sequencing matches PCR method	Duan et al., 2019; O'Donnell et al., 2023
	BAP (HN16)	4	2	Sequencing does not match PCR method, PCR shows 7 and 5 respectively	Duan et al., 2019; O'Donnell et al., 2023
Visualization of sequence	BCN	5	9		O'Donnell et al., 2023
	CEL	7	11		O'Donnell et al., 2023
	CCC	7	12		O'Donnell et al., 2023
	ADE	5	5		O'Donnell et al., 2023
	CCT	9	6		O'Donnell et al., 2023
	AFI	7	8		O'Donnell et al., 2023
	CFS	7	16		O'Donnell et al., 2023
	CDN	4	9		O'Donnell et al., 2023
	CFA	7	4		O'Donnell et al., 2023
	ABH	4	9		O'Donnell et al., 2023
	AQG	7	10		O'Donnell et al., 2023
	ADS	7	11		O'Donnell et al., 2023
	CRL	7	15		O'Donnell et al., 2023
	AIE	7	14		O'Donnell et al., 2023
	AMP	5	23		O'Donnell et al., 2023
	CNB	7	6		O'Donnell et al., 2023
	CCQ	5	8		O'Donnell et al., 2023
	CHS	7	10		O'Donnell et al., 2023
	CQI	7	8		O'Donnell et al., 2023
	AKH	5	3		O'Donnell et al., 2023
	AGK	5	10		O'Donnell et al., 2023
	CPA	4	4		O'Donnell et al., 2023
	CLN	3	10		O'Donnell et al., 2023
	BGN	4	3		O'Donnell et al., 2023
	BDM	7	14		O'Donnell et al., 2023
	AAR	3	12		O'Donnell et al., 2023
	BGP	7	13		O'Donnell et al., 2023
	BCE	5	9		O'Donnell et al., 2023
	BMC	5	9		O'Donnell et al., 2023
	BDC	6	10		O'Donnell et al., 2023
	CPG	10	12		O'Donnell et al., 2023
	CAS	7	9		O'Donnell et al., 2023
	CRB	7	17		O'Donnell et al., 2023
	CEQ	9	5		O'Donnell et al., 2023
	ACH	7	15		O'Donnell et al., 2023
	CDG	7	2		O'Donnell et al., 2023
	AEG	7	15		O'Donnell et al., 2023
	AKR	7	18		O'Donnell et al., 2023
	ASN	7	13	Msb2 sequence is inverted	O'Donnell et al., 2023
	CBM	7	5		O'Donnell et al., 2023
	CNT	7	6		O'Donnell et al., 2023
	AAC	5	5		O'Donnell et al., 2023
	AEL	5	6		O'Donnell et al., 2023
	AFH	7	18		O'Donnell et al., 2023
	AIF	7	12	HKR1 sequence is inverted	O'Donnell et al., 2023
	AIS	5	14		O'Donnell et al., 2023
	ALI	5	5		O'Donnell et al., 2023
	ANL	3	5	HKR1 sequence is inverted	O'Donnell et al., 2023
	BAF	7	9		O'Donnell et al., 2023
	BFH	4	13		O'Donnell et al., 2023
	BPK	7	10		O'Donnell et al., 2023
	CKB	7	17		O'Donnell et al., 2023
	CLL	6	8		O'Donnell et al., 2023
	CMF	10	10		O'Donnell et al., 2023
	BBF	7	15		O'Donnell et al., 2023
	BHH	7	16		O'Donnell et al., 2023
	AMM	7	6	HKR1 sequence is inverted	O'Donnell et al., 2023
	BMB	5	9		O'Donnell et al., 2023
	BAQ	7	7		O'Donnell et al., 2023
	CPI	3	6		O'Donnell et al., 2023
	AIG	7	10		O'Donnell et al., 2023
	AMH	3	6		O'Donnell et al., 2023
	AGA	7	15		O'Donnell et al., 2023
	AVI	7	7		O'Donnell et al., 2023
	AHG	7	13		O'Donnell et al., 2023
	BAH	6	5		O'Donnell et al., 2023
	BDN	3	3		O'Donnell et al., 2023
	ABA	6	7		O'Donnell et al., 2023
	BDF	7	10		O'Donnell et al., 2023
	CQS	5	8		O'Donnell et al., 2023
	ASG	7	18		O'Donnell et al., 2023
	BBL	4	7		O'Donnell et al., 2023
	BBM	5	13		O'Donnell et al., 2023
	ATM	7	11		O'Donnell et al., 2023
	AVB	9	13		O'Donnell et al., 2023
	APG	7	15		O'Donnell et al., 2023
	CEI	4	7		O'Donnell et al., 2023
	CDA	5	7		O'Donnell et al., 2023
	CBK	7	10		O'Donnell et al., 2023
	ALH	7	18		O'Donnell et al., 2023
	ADI	7	10		O'Donnell et al., 2023
	BPG	7	18		O'Donnell et al., 2023
	ANE	7	7		O'Donnell et al., 2023
	CCN	7	9		O'Donnell et al., 2023
	BDH	5	3		O'Donnell et al., 2023
	ADQ	3	10		O'Donnell et al., 2023
	AHL	9	14		O'Donnell et al., 2023
	ASB	6	10*	*6th repeat has an C insertion (63bp) causing a frameshift	O'Donnell et al., 2023
	ACA	7	10*	*8th repeat has a C insertion (63bp) causing a frameshift	O'Donnell et al., 2023
	BFP	5	8*	* 3rd repeat has C insertion (63bp) causing frameshift	O'Donnell et al., 2023
	BLD	7	15*	*6th repeat has an C insertion (63bp) causing a frameshift	O'Donnell et al., 2023
	CIH	6	27*	**HKR1 sequence is inverted, *4th, 8th, 10th, and 14th repeats are identifcal with G insertion (62bp)	O'Donnell et al., 2023
	CIC	5*	**	*3rd repeat has A insertion (42bp) causing frameshift, **HKR1 sequence is ambiguous (N)	O'Donnell et al., 2023
	GFF	6	10*	*4th repeat has C insertion (22bp) causing frameshift	O'Donnell et al., 2023
	ADM	7*	8	*5th repeat has T insertion (19bp) causing frameshift	O'Donnell et al., 2023
	AIC	7	10*	*5th repeat has C insertion (22bp) causing frameshift	O'Donnell et al., 2023
	AEH	7	18*	* 3rd repeat has C insertion (63bp) causing frameshift	O'Donnell et al., 2023
	ALS	7	20*	*14th repeat has AC deleted (73/74 bp) causing frameshift	O'Donnell et al., 2023
	ATV	7	**	**HKR1 not found	O'Donnell et al., 2024
	CGH	4	**	**HKR1 not found	O'Donnell et al., 2025
	CFC	6	**	**HKR1 not found	O'Donnell et al., 2026

Table S2. Yeast strains used in the study, related to the STAR methods

Strain (PC#)	Background	Description	Reference
313	∑1278b background	*MAT***a** SY3089 *ura3-52*	(Liu et al., 1993)
538	∑1278b background	*MAT***a** *ste4 FUS1-lacZ FUS1-HIS3 ura3-52*	(Cullen *et al.,* 2004)
586	∑1278b background	MAT**α** SY3090 *ura3-52 leu2*	(Chavel *et al.,* 2010)
673	∑1278b background	*MAT***a** *ste4 FUS1-lacZ FUS1-HIS3 ura3-52 ste20::KanMX6*	(Cullen *et al.,* 2004)
999	∑1278b background	*MAT***a** *ura3-52 Msb2-HA at 500 aa*	(Cullen *et al.,* 2004)
3384	∑1278b background	*MAT***a** *ura3-52 Msb2-HA at 500 aa* IDΔ [*MSB2Δ100-818aa*]	(Vadaie *et al.,* 2008)
7324	Wild Chinese Isolate	Original Alias FJ7 from lineage CHN I	(Wang *et al.,* 2012)
7325	Wild Chinese Isolate	Original Alias HN2 from lineage CHN I	(Wang *et al.,* 2012)
7326	Wild Chinese Isolate	Original Alias HN6 from lineage CHN I	(Wang *et al.,* 2012)
7327	Wild Chinese Isolate	Original Alias SX1 from lineage CHN II	(Wang *et al.,* 2012)
7328	Wild Chinese Isolate	Original Alias SX2 from lineage CHN II	(Wang *et al.,* 2012)
7329	Wild Chinese Isolate	Original Alias SX6 from lineage CHN II	(Wang *et al.,* 2012)
7330	Wild Chinese Isolate	Original Alias HN9 from lineage CHN III	(Wang *et al.,* 2012)
7331	Wild Chinese Isolate	Original Alias HN10 from lineage CHN III	(Wang *et al.,* 2012)
7332	Wild Chinese Isolate	Original Alias HN11 from lineage CHN III	(Wang *et al.,* 2012)
7333	Wild Chinese Isolate	Original Alias BJ6 from lineage CHN IV	(Wang *et al.,* 2012)
7334	Wild Chinese Isolate	Original Alias BJ14 from lineage CHN IV	(Wang *et al.,* 2012)
7335	Wild Chinese Isolate	Original Alias BJ20 from lineage CHN IV	(Wang *et al.,* 2012)
7336	Wild Chinese Isolate	Original Alias HN14 from lineage CHN V	(Wang *et al.,* 2012)
7337	Wild Chinese Isolate	Original Alias HN15 from lineage CHN V	(Wang *et al.,* 2012)
7338	Wild Chinese Isolate	Original Alias HN16 from lineage CHN V	(Wang *et al.,* 2012)
7834	∑1278b background	*MAT***a** *ura3-52 Msb2-HA at 500 aa* RΔ [*MSB2Δ705-816aa*]	This study
7851	∑1278b background	*MAT***a** SY3089 *ura3-52 GPA2-G132S*	This study
7866	∑1278b background	*MAT***a** SY3089 *ura3-52 gpa2*Δ::NAT	This study
7871	∑1278b background	*MAT***a** SY3089 *ura3-52 ste20*Δ::NAT	(Vandermeulen and Cullen, 2023)
7876	∑1278b background	*MAT***a** SY3089 *ura3-52 rgs2*Δ::NAT	This study
7877	∑1278b background	*MAT***a** SY3089 *ura3-52 GPA2-G132V*	This study
7893	∑1278b background	MAT**α** SY3090 *ura3-52 leu2 gpa2*Δ::GENT	This study

Table S3. Primers used in the study for PCR amplification, related to the STAR methods

Purpose	Target	Forward Primer - 5' to 3'	Reverse Primer - 5' to 3'
CRISPR	p*Cas9-sgGPA2*	AAAGTGCTACTGCTGGGTGCGTTTTAGAGCTAGAAATAGC	GCACCCAGCAGTAGCACTTTAAAGTCCCATTCGCCACCCG
	GPA2-G132S HDR template	GACAAAGAACTGAAAGTGCTACTGCTGGGTGCCAGTGAAAGTGGTAAGTCCACGGTATTG	GCTCGCTAAACCCGTTCTGGTGTAAAATCTTCAACTGCTGCAATACCGTGGACTTACCAC
	GPA2-G132V HDR template	GACAAAGAACTGAAAGTGCTACTGCTGGGTGCCGTTGAAAGTGGTAAGTCCACGGTATTG	GCTCGCTAAACCCGTTCTGGTGTAAAATCTTCAACTGCTGCAATACCGTGGACTTACCAC
p*GPA2* SDM	V41M	AGGAGCCACAGAAGACTATGAGAACAGTCAACACA	TGTGTTGACTGTTCTCATAGTCTTCTGTGGCTCCT
	G132S	ACTGCTGGGTGCCAGTGAAAGTGGTAAGTCCA	TGGACTTACCACTTTCACTGGCACCCAGCAGT
	G132R	ACTGCTGGGTGCCCGTGAAAGTGGTAAGTCCA	TGGACTTACCACTTTCACGGGCACCCAGCAGT
	G132A	ACTGCTGGGTGCCGCTGAAAGTGGTAAGTCCA	TGGACTTACCACTTTCAGCGGCACCCAGCAGT
	G132P	ACTGCTGGGTGCCCCTGAAAGTGGTAAGTCCA	TGGACTTACCACTTTCAGGGGCACCCAGCAGT
	G132M	AGTGCTACTGCTGGGTGCCATGGAAAGTGGTAAGTCCACG	CGTGGACTTACCACTTTCCATGGCACCCAGCAGTAGCACT
	G132T	ACTGCTGGGTGCCACTGAAAGTGGTAAGTCCA	TGGACTTACCACTTTCAGTGGCACCCAGCAGT
	GE132--	AGTGCTACTGCTGGGTGAAAGTGGTAAGTCCACG	CGTGGACTTACCACTTTCACCCAGCAGTAGCACT
	Y159C	AGCAGGAAATTAAAGAGTGCATCCCCTTGATCTAT	ATAGATCAAGGGGATGCACTCTTTAATTTCCTGCT
	N206Y	TGTCCTATGAAATGCCCTATAACTACACGGG	CCCGTGTAGTTATAGGGCATTTCATAGGACA
	W224G	CGCGGGCGTAATATCTACGTTGGGGGCCTTGCC	GGCAAGGCCCCCAACGTAGATATTACGCCCGC
	Q267Stop	ACAGACCCACCCAGTAGGACATATTAAGATCG	CGATCTTAATATGTCCTACTGGGTGGGTCTGT
	M275I	TAAGATCGAGACAGATAACGTCAGGGATTTTT	AAAAATCCCTGACGTTATCTGTCTCGATCTTA
	G278R	AGATGACGTCAAGGATTTTTGACACCGTCAT	ATGACGGTGTCAAAAATCCTTGACGTCATCT
	N281H	TCAGGGATTTTTCACACCGTCATTGATATGG	CCATATCAATGACGGTGTGAAAAATCCCTGA
	Q300R	TACGACGTGGGTGGACGGCGTTCCGAAAGAA	TTCTTTCGGAACGCCGTCCACCCACGTCGTA
	Q300E	TACGACGTGGGTGGAGAGCGTTCCGAAAGAA	TTCTTTCGGAACGCTCTCCACCCACGTCGTA
	F363S	ACGTCTGTCGTACTCTCTCTGAATAAAATCGACCT	AGGTCGATTTTATTCAGAGAGAGTACGACAGACG
	N368H	CTGAATAAAATCCACCTTTTTGCTGAAAAAC	GTTTTTCAGCAAAAAGGTGGATTTTATTCAG
	T387A	TACTTCCCAGACTACGCCGGCGGGTCAGACAT	ATGTCTGACCCGCCGGCGTAGTCTGGGAAGTA
	A420N	CTCACGTGACACAGAACACAGACACGTCGAAT	ATTCGACGTGTCTGTGTTCTGTGTCACGTGAG
	T421A	GTGACACAGGCCGCAGACACGTCGAATATAAGATTA	TAATCTTATATTCGACGTGTCTGCGGCCTGTGTCAC
p*STE20-GFP* SDM	T47I	ACCAAGGACCACTGGAATTTTGAACGTCAATGC	GCATTGACGTTCAAAATTCCAGTGGTCCTTGGT
	P252L	AAGCAAAAAGTAAACTGGTTTCTGTGAAAAA	TTTTTCACAGAAACCAGTTTACTTTTTGCTT
	R336M	TATAACCACCGCTTTGATGATATCTACGCCATACA	TGTATGGCGTAGATATCATCAAAGCGGTGGTTATA
	W365R	GTTTGCCGGAGGAAAGGGAAAAATTGTTGAC	GTCAACAATTTTTCCCTTTCCTCCGGCAAAC
	E366G	GTTTGCCGGAGGAATGGGGAAAATTGTTGAC	GTCAACAATTTTCCCCATTCCTCCGGCAAAC
	P479H	AGACCGGCTCCTAAGCACCCATCTTCTGCGTCCGC	GCGGACGCAGAAGATGGGTGCTTAGGAGCCGGTCT
	S497F	ACCCGTCATGAATTTTGCCGCCAATGTTTCG	CGAAACATTGGCGGCAAAATTCATGACGGGT
	S502L	TCTGCCGCCAATGTTTTGCCCTTGAAGCAGAC	GTCTGCTTCAAGGGCAAAACATTGGCGGCAGA
	S551Y	ACGTCTCCAATCATCTACACAGCTCACACACC	GGTGTGTGAGCTGTGTAGATGATTGGAGACG
	T618I	ACGGTGACCCAAGTATAAAATATGCCAATTTA	TAAATTGGCATATTTTATACTTGGGTCACCGT
	V646M	AGGTACGAATGTCTCAATGGCCATTAAGCAAATGA	TCATTTGCTTAATGGCCATTGAGACATTCGTACCT
	V646E	AGGTACGAATGTCTCAGAGGCCATTAAGCAAATGA	TCATTTGCTTAATGGCCTCTGAGACATTCGTACCT
	K673Q	TGGTCATGAAGGGTAGCCAACACCCTAATATAGTT	AACTATATTAGGGTGTTGGCTACCCTTCATGACCA
	N676D	TAGCAAACACCCTGATATAGTTAATTTCAT	ATGAAATTAACTATATCAGGGTGTTTGCTA
	K687E	TTCTTACGTTTTAGAAGGCGACCTTTGGGT	ACCCAAAGGTCGCCTTCTAAAACGTAAGAA
	T712A	ACCCATTGTATTTTGGCAGAAGGTCAAATTGGTGC	GCACCAATTTGACCTTCTGCCAAAATACAATGGGT
	Q715L	GTATTTTGACAGAAGGTCTAATTGGTGCCGTTTGT	ACAAACGGCACCAATTAGACCTTCTGTCAAAATAC
	I716T	TATTTTGACAGAAGGTCAAACTGGTGCCGTTTGTAGAGAA	TTCTCTACAAACGGCACCAGTTTGACCTTCTGTCAAAATA
	D739Y	AAAGGTGTTCTTCACAGATATATCAAATCCGATAAC	GTTATCGGATTTGATATATCTGTGAAGAACACCTTT
	M749V	AACATCCTATTGTCCGTGGAAGGGGATATTAAGTT	AACTTAATATCCCCTTCCACGGACAATAGGATGTT
	A875D	GAATATATCACGGAGATAGATGAAGCCAATTCCTC	GAGGAATTGGCTTCATCTATCTCCGTGATATATTC
HDR Knockouts	*rgs2*Δ	TACCAAGTCTATGCGACATACTGATACCGCTAGAAAATAGGCCACTAGTGGATCTG	CTATCTTTGTTGATGACTGTTTTGTCCTTTCAAACGGTTTTGAACAAGCTGAAGCTTCGTACGC
	*gpa2*Δ	ACAAATCACGCGTATTTTCAAGCAAATATCATGGGTCTCTGCGCATATAGGCCACTAGTGGATCTG	TCATTGTAACACTCCAGAGTCTTTCAATGTATTTTCCAAAATTGTTAGCTGAAGCTTCGTACGC

Data S1. Seaview alignment of the yeast PAK kinase Ste20p and the human PAK kinases Pak1p, Stk3p, and Stk4p, related to Figure 3. Amino acid sites that correspond to alleles identified in this study are boxed in red.

```
                       1
STE20                  MSNDPSAVSE LPDKDSLDNG ISNDNERAMG GNGDGGDGLR LPRTTGTLNV NALQKGTNAA
sp|Q13153|PAK1_HUMAN   MSNNGLDIQD KPPAPPM--- ---RNTSTMI GAG------- -SKDAGTLN- ----------
sp|Q13188|STK3_HUMAN   ---------- ---------- ---------- ---------- ---------- ----------
sp|Q13043|STK4_HUMAN   ---------- ---------- ---------- ---------- ---------- ----------

                      61
STE20                  HEAGGYKSMD PAKNAETTND DDNNVVSLDD PIQFTRVSSS SVISGMSSSM SPHSNIDETK
sp|Q13153|PAK1_HUMAN   ---HGSKPLP PNPEEKKKKD ---------- ---------- ---------- ----------
sp|Q13188|STK3_HUMAN   ------MEQP PAPKSKLKK- ---------- ---------- ---------- ----------
sp|Q13043|STK4_HUMAN   ---METVQLR NPPRRQLKK- ---------- ---------- ---------- ----------

                     121
STE20                  SLEAVTPNIN TSNITPDHSA DNTFSTINAS ESDHQFNDTL LSKLSLTDST ETIENNATVK
sp|Q13153|PAK1_HUMAN   ---------- ---------- ---------- ----RFYRSI L--------- ----------
sp|Q13188|STK3_HUMAN   ---------- ---------- ---------- ---------- ---------- ----------
sp|Q13043|STK4_HUMAN   ---------- ---------- ---------- ---------- ---------- ----------

                     181
STE20                  HQQPVASSTV NSNKSSTDIR RATPVSTPVI SKPSMTTTPR QINSASHSLS NPKHKQHKPK
sp|Q13153|PAK1_HUMAN   ---------- ---------- ---------- ---------- ---------- --------PG
sp|Q13188|STK3_HUMAN   ---------- ---------- ---------- ---------- ---------- ----------
sp|Q13043|STK4_HUMAN   ---------- ---------- ---------- ---------- ---------- ----------

                     241
STE20                  VKPSKPEAKS KPVSVKKSFP SKNPLKNSSP PKKQTEKSYY SSSSKKRKSG SNSGTLRMKD
sp|Q13153|PAK1_HUMAN   DKTNKKKEKE RP-------- ---------- ---------- ---------- ----------
sp|Q13188|STK3_HUMAN   ---------- ---------- ---------- ---------- ---------- ----------
sp|Q13043|STK4_HUMAN   ---------- ---------- ---------- ---------- ---------- ----------

                     301
STE20                  VFTSFVQNIK RNSQDDKRAS SSSNNSSSSS ITTALRISTP YNAKHIHHVG VDSKTGEYTG
sp|Q13153|PAK1_HUMAN   ---------- ---------- ---------- -----EISLP SDFEHTIHVG FDAVTGEFTG
sp|Q13188|STK3_HUMAN   ---------- ---------- ---------- ---------- ---------- ----------
sp|Q13043|STK4_HUMAN   ---------- ---------- ---------- ---------- ---------- ----------

                            365
                     361
STE20                  LPEEWEKLLT SSGISKREQQ QNMQAVMDIV KFYQDVTETN GEDKMFKTFN TTTGLPGSPQ
sp|Q13153|PAK1_HUMAN   MPEQWARLLQ TSNITKSEQK KNPQAVLDVL EFYNSKKTSN SQKYM----- ---------S
sp|Q13188|STK3_HUMAN   ---------- ---LSEDSLT KQPEEVFDVL E--------- ---------- ----------
sp|Q13043|STK4_HUMAN   ---------- ---LDEDSLT KQPEEVFDVL E--------- ---------- ----------

                                                                                      479
                     421
STE20                  VSTPPANSFN KFPPSTSDSH NYGSRTGTPM SNHVMSPTLN TDSSSANGKF IPSRPAPKPP
sp|Q13153|PAK1_HUMAN   FTDKSAEDYN -----SSNAL NVKAVSETP- ---AVPPVSE DEDDDDDDAT PPPVIAPRPE
sp|Q13188|STK3_HUMAN   ---------- ---------- ---------- ---------- ---------- ----------
sp|Q13043|STK4_HUMAN   ---------- ---------- ---------- ---------- ---------- ----------

                                              502
                     481
STE20                  SSASASAPII KSPVMNSAAN VSPLKQTHAP TTPNRTSPNR SSISRNATLK KEEQPLPPIP
sp|Q13153|PAK1_HUMAN   HTKSVYTRSV IEPL------ ---------P VTPTR----- ---------- --DVATSPIS
sp|Q13188|STK3_HUMAN   ---------- ---------- ---------- ---------- ---------- ----------
sp|Q13043|STK4_HUMAN   ---------- ---------- ---------- ---------- ---------- ----------

                     541
STE20                  PTKSKTSPII STAHTPQQVA QSPKAPAQET VTTPTSKPAQ ARSLSKELNE KKREERERRK
sp|Q13153|PAK1_HUMAN   PTENNTTP-- ---------- --PDALTRNT ---------- ---------E KQKKKPKMSD
sp|Q13188|STK3_HUMAN   ---------- ---------- ---------- ---------- ---------- ----------
sp|Q13043|STK4_HUMAN   ---------- ---------- ---------- ---------- ---------- ----------

                                                                         646
                     601
STE20                  KQLYAKLNEI CSDGDPSTKY ANLVKIGQGA SGGVYTAYEI GTNVSVAIKQ MNLEKQPKKE
sp|Q13153|PAK1_HUMAN   EEILEKLRSI VSVGDPKKKY TRFEKIGQGA SGTVYTAMDV ATGQEVAIKQ MNLQQQPKKE
sp|Q13188|STK3_HUMAN   ---------- ---------- ----KLGEGS YGSVFKAIHK ESGQVVAIKQ VPVESDLQE-
sp|Q13043|STK4_HUMAN   ---------- ---------- ----KLGEGS YGSVYKAIHK ETGQIVAIKQ VPVESDLQE-

                                    676                                          712 715
                     661
STE20                  LIINEILVMK GSKHPNIVNF IDSYVLKGDL WVIMEYMEGG SLTDVV--TH CILTEGQIGA
sp|Q13153|PAK1_HUMAN   LIINEILVMR ENKNPNIVNY LDSYLVGDEL WVVMEYLAGG SLTDVV--TE TCMDEGQIAA
sp|Q13188|STK3_HUMAN   -IIKEISIMQ QCDSPYVVKY YGSYFKNTDL WIVMEYCGAG SVSDIIRLRN KTLIEDEIAT
sp|Q13043|STK4_HUMAN   -IIKEISIMQ QCDSPHVVKY YGSYFKNTDL WIVMEYCGAG SVSDIIRLRN KTLTEDEIAT
```

```
                                          739
                         721
STE20                    VCRETLSGLE FLHSKGVLHR DIKSDNILLS MEGDIKLTDF GFCAQINELN LKRTTMVGTP
sp|Q13153|PAK1_HUMAN     VCRECLQALE FLHSNQVIHR DIKSDNILLG MDGSVKLTDF GFCAQITPEQ SKRSTMVGTP
sp|Q13188|STK3_HUMAN     ILKSTLKGLE YLHFMRKIHR DIKAGNILLN TEGHAKLADF GVAGQLTDTM AKRNTVIGTP
sp|Q13043|STK4_HUMAN     ILQSTLKGLE YLHFMRKIHR DIKAGNILLN TEGHAKLADF GVAGQLTDTM AKRNTVIGTP

                         781
STE20                    YWMAPEVVSR KEYGPKVDIW SLGIMIIEMI EGEPPYLNET PLRALYLIAT NGTPKLKEPE
sp|Q13153|PAK1_HUMAN     YWMAPEVVTR KAYGPKVDIW SLGIMAIEMI EGEPPYLNEN PLRALYLIAT NGTPELQNPE
sp|Q13188|STK3_HUMAN     FWMAPEVIQE IGYNCVADIW SLGITSIEMA EGKPPYADIH PMRAIFMIPT NPPPTFRKPE
sp|Q13043|STK4_HUMAN     FWMAPEVIQE IGYNCVADIW SLGITAIEMA EGKPPYADIH PMRAIFMIPT NPPPTFRKPE

                         841
STE20                    NLSSSLKKFL DWCLCVEPED RASATELLHD EYITEIAEAN SSLAPLVKLA R----LKKVA
sp|Q13153|PAK1_HUMAN     KLSAIFRDFL NRCLEMDVEK RGSAKELLQH QFL-KIAKPL SSLTPLIAAA K---------
sp|Q13188|STK3_HUMAN     LWSDDFTDFV KKCLVKNPEQ RATATQLLQH PFI-KNAKPV SILRDLITEA MEIKAKRHEE
sp|Q13043|STK4_HUMAN     LWSDNFTDFV KQCLVKSPEQ RATATQLLQH PFV-RSAKGV SILRDLINEA MDVKLKRQES

                         901
STE20                    ENMDADEDND DDNDNEHINK TNNCDDNNDS KETVN----- ---------- ----------
sp|Q13153|PAK1_HUMAN     ---------- EATKNNH--- ---------- ---------- ---------- ----------
sp|Q13188|STK3_HUMAN     QQRELE-EEE ENSDEDELDS HTMVKTSVES VGTMRATSTM SEGAQTMIEH NSTMLESDLG
sp|Q13043|STK4_HUMAN     QQREVDQDDE ENSEEDEMDS GTMVRAVGDE MGTVRVASTM TDGANTMIEH DDT-LPSQLG

                         961
STE20                    ---------- ---------- ---------- ---------- ---------- ----------
sp|Q13153|PAK1_HUMAN     ---------- ---------- ---------- ---------- ---------- ----------
sp|Q13188|STK3_HUMAN     TMVINSEDEE EEDGTMKRNA TSPQVQRPSF MDYFDKQDFK NKSHENCNQN MHEPFPMSKN
sp|Q13043|STK4_HUMAN     TMVINAEDEE EE-GTMKRRD ETMQPAKPSF LEYFEQKE-K ENQINSFGKS VPGPLKNS--

                        1021
STE20                    ---------- ---------- ---------- ---------- ---------- ----LDVTED
sp|Q13153|PAK1_HUMAN     ---------- ---------- ---------- ---------- ---------- ----------
sp|Q13188|STK3_HUMAN     VFPDNWKVPQ DGDFDFLKNL SLEELQMRLK ALDPMMEREI EELRQRYTAK RQPILDAMDA
sp|Q13043|STK4_HUMAN     ---SDWKIPQ DGDYEFLKSW TVEDLQKRLL ALDPMMEQEI EEIRQKYQSK RQPILDAIEA

                        1081
STE20                    DKQK*---
sp|Q13153|PAK1_HUMAN     --------
sp|Q13188|STK3_HUMAN     KKRRQQNF
sp|Q13043|STK4_HUMAN     KKRRQQNF
```

Data S2. Seaview alignment of Gα-proteins in *Arabidopsis thaliana*, *Candida albicans*, *Caenorhabditis elegans*, *Candida glabrata*, *Drosophila melanogaster*, *Danio rerio*, *Homo sapiens*, *Neurospora crassa*, *Saccharomyces cerevisiae*, *Saccharomyces paradoxus*, and *Ustilago maydis*, related to Figure 4. G-motifs 1-5 are labeled in red. Amino acid sites that correspond to alleles identified in this study are denoted by red arrows.

```
                           1
AthalianaGPA1              ---------- ---------- ---------- ---------- ---------- ----------
CalbicansGPA1              ---------- ---------- ---------- ---------- ---------- ----------
CalbicansGPA2              ---------- ---------- ---------- ---------- ---------- ----------
Celeganségl30              ---------- ---------- ---------- ---------- ---------- ----------
Celegansgoa1               ---------- ---------- ---------- ---------- ---------- ----------
Celegansgpa1               ---------- ---------- ---------- ---------- ---------- ----------
Celegansgpa10              ---------- ---------- ---------- ---------- ---------- ----------
Celegansgpa11              ---------- ---------- ---------- ---------- ---------- ----------
Celegansgpa12              ---------- ---------- ---------- ---------- ---------- ----------
Celegansgpa13              ---------- ---------- ---------- ---------- ---------- ----------
Celegansgpa14              ---------- ---------- ---------- ---------- ---------- ----------
Celegansgpa15              ---------- ---------- ---------- ---------- ---------- ----------
Celegansgpa16              ---------- ---------- ---------- ---------- ---------- ----------
Celegansgpa17              ---------- ---------- ---------- ---------- ---------- ----------
Celegansgpa2               ---------- ---------- ---------- ---------- ---------- ----------
Celegansgpa3               ---------- ---------- ---------- ---------- ---------- ----------
Celegansgpa4               ---------- ---------- ---------- ---------- ---------- ----------
Celegansgpa5               ---------- ---------- ---------- ---------- ---------- ----------
Celegansgpa6               ---------- ---------- ---------- ---------- ---------- ----------
Celegansgpa7               ---------- ---------- ---------- ---------- ---------- ----------
Celegansgpa8               ---------- ---------- ---------- ---------- ---------- ----------
Celegansgpa9               ---------- ---------- ---------- ---------- ---------- ----------
Celegansgsa1               ---------- ---------- ---------- ---------- ---------- ----------
CglabrataGPA1              ---------- ---------- ---------- ---------- ---------- ----------
CglabrataGPA2              ---------- ---------- ---------- ---------- ---------- ----------
DmelanogasterCTA           ---------- ---------- ---------- ---------- ---------- ----------
DmelanogasterGalphaf       ---------- ---------- ---------- ---------- ---------- ----------
DmelanogasterGalphai       ---------- ---------- ---------- ---------- ---------- ----------
DmelanogasterGalphao       ---------- ---------- ---------- ---------- ---------- ----------
DmelanogasterGalphaq       ---------- ---------- ---------- ---------- ---------- ----------
DmelanogasterGalphas       ---------- ---------- ---------- ---------- ---------- ----------
Dreriognal1                ---------- ---------- ---------- ---------- ---------- ----------
Dreriognal1a               ---------- ---------- ---------- ---------- ---------- ----------
Dreriognal2                ---------- ---------- ---------- ---------- ---------- ----------
Dreriognal3                ---------- ---------- ---------- ---------- ---------- ----------
Dreriognal4a               ---------- ---------- ---------- ---------- ---------- ----------
Dreriognal2                ---------- ---------- ---------- ---------- ---------- ----------
Dreriognas                 ---------- ---------- ---------- ---------- ---------- ----------
Dreriognav1                ---------- ---------- ---------- ---------- ---------- ----------
Dreriognaz                 ---------- ---------- ---------- ---------- ---------- ----------
HsapiensGNA11              ---------- ---------- ---------- ---------- ---------- ----------
HsapiensGNA12              ---------- ---------- ---------- ---------- ---------- ----------
HsapiensGNA13              ---------- ---------- ---------- ---------- ---------- ----------
HsapiensGNA14              ---------- ---------- ---------- ---------- ---------- ----------
HsapiensGNA15              ---------- ---------- ---------- ---------- ---------- ----------
HsapiensGNAI1              ---------- ---------- ---------- ---------- ---------- ----------
HsapiensGNAI2              ---------- ---------- ---------- ---------- ---------- ----------
HsapiensGNAI3              ---------- ---------- ---------- ---------- ---------- ----------
HsapiensGNAL               ---------- ---------- ---------- ---------- ---------- ----------
HsapiensGNAO1              ---------- ---------- ---------- ---------- ---------- ----------
HsapiensGNAQ               ---------- ---------- ---------- ---------- ---------- ----------
HsapiensGNAS1              MGVRNCLYGN NMSGQRDIPP EIGEQPEQPP LEAPGAAAPG AGPSPAEEME TEPPHNEPIP
HsapiensGNAS2              ---------- ---------- ---------- ---------- ---------- ----------
HsapiensGNAT1              ---------- ---------- ---------- ---------- ---------- ----------
HsapiensGNAT2              ---------- ---------- ---------- ---------- ---------- ----------
HsapiensGNAT3              ---------- ---------- ---------- ---------- ---------- ----------
HsapiensGNAZ               ---------- ---------- ---------- ---------- ---------- ----------
Ncrassagna1                ---------- ---------- ---------- ---------- ---------- ----------
Ncrassagna2                ---------- ---------- ---------- ---------- ---------- ----------
Ncrassagna3                ---------- ---------- ---------- ---------- ---------- ----------
ScervisiaeGPA1             ---------- ---------- ---------- ---------- ---------- ----------
ScervisiaeGPA2             ---------- ---------- ---------- ---------- ---------- ----------
SparadoxusGPA1             ---------- ---------- ---------- ---------- ---------- ----------
SparadoxusGPA2             ---------- ---------- ---------- ---------- ---------- ----------
Umaydisgpa1                ---------- ---------- ---------- ---------- ---------- ----------
Umaydisgpa2                ---------- ---------- ---------- ---------- ---------- ----------
Umaydisgpa3                ---------- ---------- ---------- ---------- ---------- ----------
Umaydisgpa4                ---------- ---------- ---------- ---------- ---------- ----------
```

	61					
AthalianaGPA1	----------	----------	----------	----------	----------	----------
CalbicansGPA1	----------	----------	----------	----------	----------	----------
CalbicansGPA2	----------	----------	----------	----------	----------	----------
Celeganssegl30	----------	----------	----------	----------	----------	----------
Celegansgoa1	----------	----------	----------	----------	----------	----------
Celegansgpa1	----------	----------	----------	----------	----------	----------
Celegansgpa10	----------	----------	----------	----------	----------	----------
Celegansgpa11	----------	----------	----------	----------	----------	----------
Celegansgpa12	----------	----------	----------	----------	----------	----------
Celegansgpa13	----------	----------	----------	----------	----------	----------
Celegansgpa14	----------	----------	----------	----------	----------	----------
Celegansgpa15	----------	----------	----------	----------	----------	----------
Celegansgpa16	----------	----------	----------	----------	----------	----------
Celegansgpa17	----------	----------	----------	----------	----------	----------
Celegansgpa2	----------	----------	----------	----------	----------	----------
Celegansgpa3	----------	----------	----------	----------	----------	----------
Celegansgpa4	----------	----------	----------	----------	----------	----------
Celegansgpa5	----------	----------	----------	----------	----------	----------
Celegansgpa6	----------	----------	----------	----------	----------	----------
Celegansgpa7	----------	----------	----------	----------	----------	----------
Celegansgpa8	----------	----------	----------	----------	----------	----------
Celegansgpa9	----------	----------	----------	----------	----------	----------
Celegansgsa1	----------	----------	----------	----------	----------	----------
CglabrataGPA1	----------	----------	----------	----------	----------	----------
CglabrataGPA2	----------	----------	----------	----------	----------	----------
DmelanogasterCTA	----------	----------	----------	----------	----------	----------
DmelanogasterGalphaf	----------	----------	----------	----------	----------	----------
DmelanogasterGalphai	----------	----------	----------	----------	----------	----------
DmelanogasterGalphao	----------	----------	----------	----------	----------	----------
DmelanogasterGalphaq	----------	----------	----------	----------	----------	----------
DmelanogasterGalphas	----------	----------	----------	----------	----------	----------
Dreriognal1	----------	----------	----------	----------	----------	----------
Dreriognal1a	----------	----------	----------	----------	----------	----------
Dreriognal2	----------	----------	----------	----------	----------	----------
Dreriognal3	----------	----------	----------	----------	----------	----------
Dreriognal4a	----------	----------	----------	----------	----------	----------
Dreriognal2	----------	----------	----------	----------	----------	----------
Dreriognas	----------	----------	----------	----------	----------	----------
Dreriognav1	----------	----------	----------	----------	----------	----------
Dreriognaz	----------	----------	----------	----------	----------	----------
HsapiensGNA11	----------	----------	----------	----------	----------	----------
HsapiensGNA12	----------	----------	----------	----------	----------	----------
HsapiensGNA13	----------	----------	----------	----------	----------	----------
HsapiensGNA14	----------	----------	----------	----------	----------	----------
HsapiensGNA15	----------	----------	----------	----------	----------	----------
HsapiensGNAI1	----------	----------	----------	----------	----------	----------
HsapiensGNAI2	----------	----------	----------	----------	----------	----------
HsapiensGNAI3	----------	----------	----------	----------	----------	----------
HsapiensGNAL	----------	----------	----------	----------	----------	----------
HsapiensGNAO1	----------	----------	----------	----------	----------	----------
HsapiensGNAQ	----------	----------	----------	----------	----------	----------
HsapiensGNAS1	VENDGEACGP	PEVSRPNFQV	LNPAFREAGA	HGSYSPPPEE	AMPFEAEQPS	LGGFWPTLEQ
HsapiensGNAS2	----------	----------	----------	----------	----------	----------
HsapiensGNAT1	----------	----------	----------	----------	----------	----------
HsapiensGNAT2	----------	----------	----------	----------	----------	----------
HsapiensGNAT3	----------	----------	----------	----------	----------	----------
HsapiensGNAZ	----------	----------	----------	----------	----------	----------
Ncrassagna1	----------	----------	----------	----------	----------	----------
Ncrassagna2	----------	----------	----------	----------	----------	----------
Ncrassagna3	----------	----------	----------	----------	----------	----------
ScervisiaeGPA1	----------	----------	----------	----------	----------	----------
ScervisiaeGPA2	----------	----------	----------	----------	----------	----------
SparadoxusGPA1	----------	----------	----------	----------	----------	----------
SparadoxusGPA2	----------	----------	----------	----------	----------	----------
Umaydisgpa1	----------	----------	----------	----------	----------	----------
Umaydisgpa2	----------	----------	----------	----------	----------	----------
Umaydisgpa3	----------	----------	----------	----------	----------	----------
Umaydisgpa4	----------	----------	----------	----------	----------	----------

```
                          121
AthalianaGPA1             ---------- ---------- ---------- ---------- ---------- ----------
CalbicansGPA1             ---------- ---------- ---------- ---------- ---------- ----------
CalbicansGPA2             ---------- ---------- ---------- ---------- ---------- ----------
Celeganseg130             ---------- ---------- ---------- ---------- ---------- ----------
Celegansgoa1              ---------- ---------- ---------- ---------- ---------- ----------
Celegansgpa1              ---------- ---------- ---------- ---------- ---------- ----------
Celegansgpa10             ---------- ---------- ---------- ---------- ---------- ----------
Celegansgpa11             ---------- ---------- ---------- ---------- ---------- ----------
Celegansgpa12             ---------- ---------- ---------- ---------- ---------- ----------
Celegansgpa13             ---------- ---------- ---------- ---------- ---------- ----------
Celegansgpa14             ---------- ---------- ---------- ---------- ---------- ----------
Celegansgpa15             ---------- ---------- ---------- ---------- ---------- ----------
Celegansgpa16             ---------- ---------- ---------- ---------- ---------- ----------
Celegansgpa17             ---------- ---------- ---------- ---------- ---------- ----------
Celegansgpa2              ---------- ---------- ---------- ---------- ---------- ----------
Celegansgpa3              ---------- ---------- ---------- ---------- ---------- ----------
Celegansgpa4              ---------- ---------- ---------- ---------- ---------- ----------
Celegansgpa5              ---------- ---------- ---------- ---------- ---------- ----------
Celegansgpa6              ---------- ---------- ---------- ---------- ---------- ----------
Celegansgpa7              ---------- ---------- ---------- ---------- ---------- ----------
Celegansgpa8              ---------- ---------- ---------- ---------- ---------- ----------
Celegansgpa9              ---------- ---------- ---------- ---------- ---------- ----------
Celegansgsa1              ---------- ---------- ---------- ---------- ---------- ----------
CglabrataGPA1             ---------- ---------- ---------- ---------- ---------- ----------
CglabrataGPA2             ---------- ---------- ---------- ---------- ---------- ----------
DmelanogasterCTA          ---------- ---------- ---------- ---------- ---------- ----------
DmelanogasterGalphaf      ---------- ---------- ---------- ---------- ---------- ----------
DmelanogasterGalphai      ---------- ---------- ---------- ---------- ---------- ----------
DmelanogasterGalphao      ---------- ---------- ---------- ---------- ---------- ----------
DmelanogasterGalphaq      ---------- ---------- ---------- ---------- ---------- ----------
DmelanogasterGalphas      ---------- ---------- ---------- ---------- ---------- ----------
Dreriognal1               ---------- ---------- ---------- ---------- ---------- ----------
Dreriognal1a              ---------- ---------- ---------- ---------- ---------- ----------
Dreriognal2               ---------- ---------- ---------- ---------- ---------- ----------
Dreriognal3               ---------- ---------- ---------- ---------- ---------- ----------
Dreriognal4a              ---------- ---------- ---------- ---------- ---------- ----------
Dreriognal2               ---------- ---------- ---------- ---------- ---------- ----------
Dreriognas                ---------- ---------- ---------- ---------- ---------- ----------
Dreriognav1               ---------- ---------- ---------- ---------- ---------- ----------
Dreriognaz                ---------- ---------- ---------- ---------- ---------- ----------
HsapiensGNA11             ---------- ---------- ---------- ---------- ---------- ----------
HsapiensGNA12             ---------- ---------- ---------- ---------- ---------- ----------
HsapiensGNA13             ---------- ---------- ---------- ---------- ---------- ----------
HsapiensGNA14             ---------- ---------- ---------- ---------- ---------- ----------
HsapiensGNA15             ---------- ---------- ---------- ---------- ---------- ----------
HsapiensGNAI1             ---------- ---------- ---------- ---------- ---------- ----------
HsapiensGNAI2             ---------- ---------- ---------- ---------- ---------- ----------
HsapiensGNAI3             ---------- ---------- ---------- ---------- ---------- ----------
HsapiensGNAL              ---------- ---------- ---------- ---------- ---------- ----------
HsapiensGNAO1             ---------- ---------- ---------- ---------- ---------- ----------
HsapiensGNAQ              ---------- ---------- ---------- ---------- ---------- ----------
HsapiensGNAS1             PGFPSGVHAG LEAFGPALME PGAFSGARPG LGGYSPPPEE AMPFEFDQPA QRGCSQLLLQ
HsapiensGNAS2             ---------- ---------- ---------- ---------- ---------- ----------
HsapiensGNAT1             ---------- ---------- ---------- ---------- ---------- ----------
HsapiensGNAT2             ---------- ---------- ---------- ---------- ---------- ----------
HsapiensGNAT3             ---------- ---------- ---------- ---------- ---------- ----------
HsapiensGNAZ              ---------- ---------- ---------- ---------- ---------- ----------
Ncrassagna1               ---------- ---------- ---------- ---------- ---------- ----------
Ncrassagna2               ---------- ---------- ---------- ---------- ---------- ----------
Ncrassagna3               ---------- ---------- ---------- ---------- ---------- ----------
ScervisiaeGPA1            ---------- ---------- ---------- ---------- ---------- ----------
ScervisiaeGPA2            ---------- ---------- ---------- ---------- ---------- ----------
SparadoxusGPA1            ---------- ---------- ---------- ---------- ---------- ----------
SparadoxusGPA2            ---------- ---------- ---------- ---------- ---------- ----------
Umaydisgpa1               ---------- ---------- ---------- ---------- ---------- ----------
Umaydisgpa2               ---------- ---------- ---------- ---------- ---------- ----------
Umaydisgpa3               ---------- ---------- ---------- ---------- ---------- ----------
Umaydisgpa4               ---------- ---------- ---------- ---------- ---------- ----------
```

```
                          181
AthalianaGPA1             ---------- ---------- ---------- ---------- ---------- ----------
CalbicansGPA1             ---------- ---------- ---------- ---------- ---------- ----------
CalbicansGPA2             ---------- ---------- ---------- ---------- ---------- ----------
Celeganségl30             ---------- ---------- ---------- ---------- ---------- ----------
Celegansgoa1              ---------- ---------- ---------- ---------- ---------- ----------
Celegansgpa1              ---------- ---------- ---------- ---------- ---------- ----------
Celegansgpa10             ---------- ---------- ---------- ---------- ---------- ----------
Celegansgpa11             ---------- ---------- ---------- ---------- ---------- ----------
Celegansgpa12             ---------- ---------- ---------- ---------- ---------- ----------
Celegansgpa13             ---------- ---------- ---------- ---------- ---------- ----------
Celegansgpa14             ---------- ---------- ---------- ---------- ---------- ----------
Celegansgpa15             ---------- ---------- ---------- ---------- ---------- ----------
Celegansgpa16             ---------- ---------- ---------- ---------- ---------- ----------
Celegansgpa17             ---------- ---------- ---------- ---------- ---------- ----------
Celegansgpa2              ---------- ---------- ---------- ---------- ---------- ----------
Celegansgpa3              ---------- ---------- ---------- ---------- ---------- ----------
Celegansgpa4              ---------- ---------- ---------- ---------- ---------- ----------
Celegansgpa5              ---------- ---------- ---------- ---------- ---------- ----------
Celegansgpa6              ---------- ---------- ---------- ---------- ---------- ----------
Celegansgpa7              ---------- ---------- ---------- ---------- ---------- ----------
Celegansgpa8              ---------- ---------- ---------- ---------- ---------- ----------
Celegansgpa9              ---------- ---------- ---------- ---------- ---------- ----------
Celegansgsa1              ---------- ---------- ---------- ---------- ---------- ----------
CglabrataGPA1             ---------- ---------- ---------- ---------- ---------- ----------
CglabrataGPA2             ---------- ---------- ---------- ---------- ---------- ----------
DmelanogasterCTA          ---------- ---------- ---------- ---------- ---------- ----------
DmelanogasterGalphaf      ---------- ---------- ---------- ---------- ---------- ----------
DmelanogasterGalphai      ---------- ---------- ---------- ---------- ---------- ----------
DmelanogasterGalphao      ---------- ---------- ---------- ---------- ---------- ----------
DmelanogasterGalphaq      ---------- ---------- ---------- ---------- ---------- ----------
DmelanogasterGalphas      ---------- ---------- ---------- ---------- ---------- ----------
Dreriogna11               ---------- ---------- ---------- ---------- ---------- ----------
Dreriogna11a              ---------- ---------- ---------- ---------- ---------- ----------
Dreriogna12               ---------- ---------- ---------- ---------- ---------- ----------
Dreriogna13               ---------- ---------- ---------- ---------- ---------- ----------
Dreriogna14a              ---------- ---------- ---------- ---------- ---------- ----------
Dreriognal2               ---------- ---------- ---------- ---------- ---------- ----------
Dreriognas                ---------- ---------- ---------- ---------- ---------- ----------
Dreriognav1               ---------- ---------- ---------- ---------- ---------- ----------
Dreriognaz                ---------- ---------- ---------- ---------- ---------- ----------
HsapiensGNA11             ---------- ---------- ---------- ---------- ---------- ----------
HsapiensGNA12             ---------- ---------- ---------- ---------- ---------- ----------
HsapiensGNA13             ---------- ---------- ---------- ---------- ---------- ----------
HsapiensGNA14             ---------- ---------- ---------- ---------- ---------- ----------
HsapiensGNA15             ---------- ---------- ---------- ---------- ---------- ----------
HsapiensGNAI1             ---------- ---------- ---------- ---------- ---------- ----------
HsapiensGNAI2             ---------- ---------- ---------- ---------- ---------- ----------
HsapiensGNAI3             ---------- ---------- ---------- ---------- ---------- ----------
HsapiensGNAL              ---------- ---------- ---------- ---------- ---------- ----------
HsapiensGNAO1             ---------- ---------- ---------- ---------- ---------- ----------
HsapiensGNAQ              ---------- ---------- ---------- ---------- ---------- ----------
HsapiensGNAS1             VPDLAPGGPG AAGVPGAPPE EPQALRPAKA GSRGGYSPPP EETMPFELDG EGFGDDSPPP
HsapiensGNAS2             ---------- ---------- ---------- ---------- ---------- ----------
HsapiensGNAT1             ---------- ---------- ---------- ---------- ---------- ----------
HsapiensGNAT2             ---------- ---------- ---------- ---------- ---------- ----------
HsapiensGNAT3             ---------- ---------- ---------- ---------- ---------- ----------
HsapiensGNAZ              ---------- ---------- ---------- ---------- ---------- ----------
Ncrassagna1               ---------- ---------- ---------- ---------- ---------- ----------
Ncrassagna2               ---------- ---------- ---------- ---------- ---------- ----------
Ncrassagna3               ---------- ---------- ---------- ---------- ---------- ----------
ScervisiaeGPA1            ---------- ---------- ---------- ---------- ---------- ----------
ScervisiaeGPA2            ---------- ---------- ---------- ---------- ---------- ----------
SparadoxusGPA1            ---------- ---------- ---------- ---------- ---------- ----------
SparadoxusGPA2            ---------- ---------- ---------- ---------- ---------- ----------
Umaydisgpa1               ---------- ---------- ---------- ---------- ---------- ----------
Umaydisgpa2               ---------- ---------- ---------- ---------- ---------- ----------
Umaydisgpa3               ---------- ---------- ---------- ---------- ---------- ----------
Umaydisgpa4               ---------- ---------- ---------- ---------- ---------- ----------
```

```
                         241
AthalianaGPA1            ---------- ---------- ---------- ---------- ---------- ----------
CalbicansGPA1            ---------- ---------- ---------- ---------- ---------- ----------
CalbicansGPA2            ---------- ---------- ---------- ---------- ---------- ----------
Celeganseg130            ---------- ---------- ---------- ---------- ---------- ----------
Celegansgoa1             ---------- ---------- ---------- ---------- ---------- ----------
Celegansgpa1             ---------- ---------- ---------- ---------- ---------- ----------
Celegansgpa10            ---------- ---------- ---------- ---------- ---------- ----------
Celegansgpa11            ---------- ---------- ---------- ---------- ---------- ----------
Celegansgpa12            ---------- ---------- ---------- ---------- ---------- ----------
Celegansgpa13            ---------- ---------- ---------- ---------- ---------- ----------
Celegansgpa14            ---------- ---------- ---------- ---------- ---------- ----------
Celegansgpa15            ---------- ---------- ---------- ---------- ---------- ----------
Celegansgpa16            ---------- ---------- ---------- ---------- ---------- ----------
Celegansgpa17            ---------- ---------- ---------- ---------- ---------- ----------
Celegansgpa2             ---------- ---------- ---------- ---------- ---------- ----------
Celegansgpa3             ---------- ---------- ---------- ---------- ---------- ----------
Celegansgpa4             ---------- ---------- ---------- ---------- ---------- ----------
Celegansgpa5             ---------- ---------- ---------- ---------- ---------- ----------
Celegansgpa6             ---------- ---------- ---------- ---------- ---------- ----------
Celegansgpa7             ---------- ---------- ---------- ---------- ---------- ----------
Celegansgpa8             ---------- ---------- ---------- ---------- ---------- ----------
Celegansgpa9             ---------- ---------- ---------- ---------- ---------- ----------
Celegansgsa1             ---------- ---------- ---------- ---------- ---------- ----------
CglabrataGPA1            ---------- ---------- ---------- ---------- ---------- ----------
CglabrataGPA2            ---------- ---------- ---------- ---------- ---------- ----------
DmelanogasterCTA         ---------- ---------- ---------- ---------- ---------- ----------
DmelanogasterGalphaf     ---------- ---------- ---------- ---------- ---------- ----------
DmelanogasterGalphai     ---------- ---------- ---------- ---------- ---------- ----------
DmelanogasterGalphao     ---------- ---------- ---------- ---------- ---------- ----------
DmelanogasterGalphaq     ---------- ---------- ---------- ---------- ---------- ----------
DmelanogasterGalphas     ---------- ---------- ---------- ---------- ---------- ----------
Dreriogna11              ---------- ---------- ---------- ---------- ---------- ----------
Dreriogna11a             ---------- ---------- ---------- ---------- ---------- ----------
Dreriogna12              ---------- ---------- ---------- ---------- ---------- ----------
Dreriogna13              ---------- ---------- ---------- ---------- ---------- ----------
Dreriogna14a             ---------- ---------- ---------- ---------- ---------- ----------
Dreriognal2              ---------- ---------- ---------- ---------- ---------- ----------
Dreriognas               ---------- ---------- ---------- ---------- ---------- ----------
Dreriognav1              ---------- ---------- ---------- ---------- ---------- ----------
Dreriognaz               ---------- ---------- ---------- ---------- ---------- ----------
HsapiensGNA11            ---------- ---------- ---------- ---------- ---------- ----------
HsapiensGNA12            ---------- ---------- ---------- ---------- ---------- ----------
HsapiensGNA13            ---------- ---------- ---------- ---------- ---------- ----------
HsapiensGNA14            ---------- ---------- ---------- ---------- ---------- ----------
HsapiensGNA15            ---------- ---------- ---------- ---------- ---------- ----------
HsapiensGNAI1            ---------- ---------- ---------- ---------- ---------- ----------
HsapiensGNAI2            ---------- ---------- ---------- ---------- ---------- ----------
HsapiensGNAI3            ---------- ---------- ---------- ---------- ---------- ----------
HsapiensGNAL             ---------- ---------- ---------- ---------- ---------- ----------
HsapiensGNAO1            ---------- ---------- ---------- ---------- ---------- ----------
HsapiensGNAQ             ---------- ---------- ---------- ---------- ---------- ----------
HsapiensGNAS1            GLSRVIAQVD GSSQFAAVAA SSAVRLTPAA NAPPLWVPGA IGSPSQEAVR PPSNFTGSSP
HsapiensGNAS2            ---------- ---------- ---------- ---------- ---------- ----------
HsapiensGNAT1            ---------- ---------- ---------- ---------- ---------- ----------
HsapiensGNAT2            ---------- ---------- ---------- ---------- ---------- ----------
HsapiensGNAT3            ---------- ---------- ---------- ---------- ---------- ----------
HsapiensGNAZ             ---------- ---------- ---------- ---------- ---------- ----------
Ncrassagna1              ---------- ---------- ---------- ---------- ---------- ----------
Ncrassagna2              ---------- ---------- ---------- ---------- ---------- ----------
Ncrassagna3              ---------- ---------- ---------- ---------- ---------- ----------
ScervisiaeGPA1           ---------- ---------- ---------- ---------- ---------- ----------
ScervisiaeGPA2           ---------- ---------- ---------- ---------- ---------- ----------
SparadoxusGPA1           ---------- ---------- ---------- ---------- ---------- ----------
SparadoxusGPA2           ---------- ---------- ---------- ---------- ---------- ----------
Umaydisgpa1              ---------- ---------- ---------- ---------- ---------- ----------
Umaydisgpa2              ---------- ---------- ---------- ---------- ---------- ----------
Umaydisgpa3              ---------- ---------- ---------- ---------- ---------- ----------
Umaydisgpa4              ---------- ---------- ---------- ---------- ---------- ----------
```

```
                          301
AthalianaGPA1             ---------- ---------- ---------- ---------- ---------- ----------
CalbicansGPA1             ---------- ---------- ---------- ---------- ---------- ----------
CalbicansGPA2             ---------- ---------- ---------- ---------- ---------- ----------
Celeganseg130             ---------- ---------- ---------- ---------- ---------- ----------
Celegansgoa1              ---------- ---------- ---------- ---------- ---------- ----------
Celegansgpa1              ---------- ---------- ---------- ---------- ---------- ----------
Celegansgpa10             ---------- ---------- ---------- ---------- ---------- ----------
Celegansgpa11             ---------- ---------- ---------- ---------- ---------- ----------
Celegansgpa12             ---------- ---------- ---------- ---------- ---------- ----------
Celegansgpa13             ---------- ---------- ---------- ---------- ---------- ----------
Celegansgpa14             ---------- ---------- ---------- ---------- ---------- ----------
Celegansgpa15             ---------- ---------- ---------- ---------- ---------- ----------
Celegansgpa16             ---------- ---------- ---------- ---------- ---------- ----------
Celegansgpa17             ---------- ---------- ---------- ---------- ---------- ----------
Celegansgpa2              ---------- ---------- ---------- ---------- ---------- ----------
Celegansgpa3              ---------- ---------- ---------- ---------- ---------- ----------
Celegansgpa4              ---------- ---------- ---------- ---------- ---------- ----------
Celegansgpa5              ---------- ---------- ---------- ---------- ---------- ----------
Celegansgpa6              ---------- ---------- ---------- ---------- ---------- ----------
Celegansgpa7              ---------- ---------- ---------- ---------- ---------- ----------
Celegansgpa8              ---------- ---------- ---------- ---------- ---------- ----------
Celegansgpa9              ---------- ---------- ---------- ---------- ---------- ----------
Celegansgsa1              ---------- ---------- ---------- ---------- ---------- ----------
CglabrataGPA1             ---------- ---------- ---------- ---------- ---------- ----------
CglabrataGPA2             ---------- ---------- ---------- ---------- ---------- ----------
DmelanogasterCTA          ---------- ---------- ---------- ---------- ---------- ----------
DmelanogasterGalphaf      ---------- ---------- ---------- ---------- ---------- ----------
DmelanogasterGalphai      ---------- ---------- ---------- ---------- ---------- ----------
DmelanogasterGalphao      ---------- ---------- ---------- ---------- ---------- ----------
DmelanogasterGalphaq      ---------- ---------- ---------- ---------- ---------- ----------
DmelanogasterGalphas      ---------- ---------- ---------- ---------- ---------- ----------
Dreriognal1               ---------- ---------- ---------- ---------- ---------- ----------
Dreriognal1a              ---------- ---------- ---------- ---------- ---------- ----------
Dreriognal2               ---------- ---------- ---------- ---------- ---------- ----------
Dreriognal3               ---------- ---------- ---------- ---------- ---------- ----------
Dreriognal4a              ---------- ---------- ---------- ---------- ---------- ----------
Dreriognal2               ---------- ---------- ---------- ---------- ---------- ----------
Dreriognas                ---------- ---------- ---------- ---------- ---------- ----------
Dreriognav1               ---------- ---------- ---------- ---------- ---------- ----------
Dreriognaz                ---------- ---------- ---------- ---------- ---------- ----------
HsapiensGNA11             ---------- ---------- ---------- ---------- ---------- ----------
HsapiensGNA12             ---------- ---------- ---------- ---------- ---------- ----------
HsapiensGNA13             ---------- ---------- ---------- ---------- ---------- ----------
HsapiensGNA14             ---------- ---------- ---------- ---------- ---------- ----------
HsapiensGNA15             ---------- ---------- ---------- ---------- ---------- ----------
HsapiensGNAI1             ---------- ---------- ---------- ---------- ---------- ----------
HsapiensGNAI2             ---------- ---------- ---------- ---------- ---------- ----------
HsapiensGNAI3             ---------- ---------- ---------- ---------- ---------- ----------
HsapiensGNAL              ---------- ---------- ---------- ---------- ---------- ----------
HsapiensGNAO1             ---------- ---------- ---------- ---------- ---------- ----------
HsapiensGNAQ              ---------- ---------- ---------- ---------- ---------- ----------
HsapiensGNAS1             WMEISGPPFE IGSAPAGVDD TPVNMDSPPI ALDGPPIKVS GAPDKRERAE RPPVEEEAAE
HsapiensGNAS2             ---------- ---------- ---------- ---------- ---------- ----------
HsapiensGNAT1             ---------- ---------- ---------- ---------- ---------- ----------
HsapiensGNAT2             ---------- ---------- ---------- ---------- ---------- ----------
HsapiensGNAT3             ---------- ---------- ---------- ---------- ---------- ----------
HsapiensGNAZ              ---------- ---------- ---------- ---------- ---------- ----------
Ncrassagna1               ---------- ---------- ---------- ---------- ---------- ----------
Ncrassagna2               ---------- ---------- ---------- ---------- ---------- ----------
Ncrassagna3               ---------- ---------- ---------- ---------- ---------- ----------
ScervisiaeGPA1            ---------- ---------- ---------- ---------- ---------- ----------
ScervisiaeGPA2            ---------- ---------- ---------- ---------- ---------- ----------
SparadoxusGPA1            ---------- ---------- ---------- ---------- ---------- ----------
SparadoxusGPA2            ---------- ---------- ---------- ---------- ---------- ----------
Umaydisgpa1               ---------- ---------- ---------- ---------- ---------- ----------
Umaydisgpa2               ---------- ---------- ---------- ---------- ---------- ----------
Umaydisgpa3               ---------- ---------- ---------- ---------- ---------- ----------
Umaydisgpa4               ---------- ---------- ---------- ---------- ---------- ----------
```

```
                      361
AthalianaGPA1          ---------- ---------- ---------- ---------- ---------- ----------
CalbicansGPA1          ---------- ---------- ---------- ---------- ---------- ----------
CalbicansGPA2          ---------- ---------- ---------- ---------- ---------- ----------
Celeganseg130          ---------- ---------- ---------- ---------- ---------- ----------
Celegansgoa1           ---------- ---------- ---------- ---------- ---------- ----------
Celegansgpa1           ---------- ---------- ---------- ---------- ---------- ----------
Celegansgpa10          ---------- ---------- ---------- ---------- ---------- ----------
Celegansgpa11          ---------- ---------- ---------- ---------- ---------- ----------
Celegansgpa12          ---------- ---------- ---------- ---------- ---------- ----------
Celegansgpa13          ---------- ---------- ---------- ---------- ---------- ----------
Celegansgpa14          ---------- ---------- ---------- ---------- ---------- ----------
Celegansgpa15          ---------- ---------- ---------- ---------- ---------- ----------
Celegansgpa16          ---------- ---------- ---------- ---------- ---------- ----------
Celegansgpa17          ---------- ---------- ---------- ---------- ---------- ----------
Celegansgpa2           ---------- ---------- ---------- ---------- ---------- ----------
Celegansgpa3           ---------- ---------- ---------- ---------- ---------- ----------
Celegansgpa4           ---------- ---------- ---------- ---------- ---------- ----------
Celegansgpa5           ---------- ---------- ---------- ---------- ---------- ----------
Celegansgpa6           ---------- ---------- ---------- ---------- ---------- ----------
Celegansgpa7           ---------- ---------- ---------- ---------- ---------- ----------
Celegansgpa8           ---------- ---------- ---------- ---------- ---------- ----------
Celegansgpa9           ---------- ---------- ---------- ---------- ---------- ----------
Celegansgsa1           ---------- ---------- ---------- ---------- ---------- ----------
CglabrataGPA1          ---------- ---------- ---------- ---------- ---------- ----------
CglabrataGPA2          ---------- ---------- ---------- ---------- ---------- ----------
DmelanogasterCTA       ---------- ---------- ---------- ---------- ---------- ----------
DmelanogasterGalphaf   ---------- ---------- ---------- ---------- ---------- ----------
DmelanogasterGalphai   ---------- ---------- ---------- ---------- ---------- ----------
DmelanogasterGalphao   ---------- ---------- ---------- ---------- ---------- ----------
DmelanogasterGalphaq   ---------- ---------- ---------- ---------- ---------- ----------
DmelanogasterGalphas   ---------- ---------- ---------- ---------- ---------- ----------
Dreriogna11            ---------- ---------- ---------- ---------- ---------- ----------
Dreriogna11a           ---------- ---------- ---------- ---------- ---------- ----------
Dreriogna12            ---------- ---------- ---------- ---------- ---------- ----------
Dreriogna13            ---------- ---------- ---------- ---------- ---------- ----------
Dreriogna14a           ---------- ---------- ---------- ---------- ---------- ----------
Dreriogna12            ---------- ---------- ---------- ---------- ---------- ----------
Dreriognas             ---------- ---------- ---------- ---------- ---------- ----------
Dreriognav1            ---------- ---------- ---------- ---------- ---------- ----------
Dreriognaz             ---------- ---------- ---------- ---------- ---------- ----------
HsapiensGNA11          ---------- ---------- ---------- ---------- ---------- ----------
HsapiensGNA12          ---------- ---------- ---------- ---------- ---------- ----------
HsapiensGNA13          ---------- ---------- ---------- ---------- ---------- ----------
HsapiensGNA14          ---------- ---------- ---------- ---------- ---------- ----------
HsapiensGNA15          ---------- ---------- ---------- ---------- ---------- ----------
HsapiensGNAI1          ---------- ---------- ---------- ---------- ---------- ----------
HsapiensGNAI2          ---------- ---------- ---------- ---------- ---------- ----------
HsapiensGNAI3          ---------- ---------- ---------- ---------- ---------- ----------
HsapiensGNAL           ---------- ---------- ---------- ---------- ---------- ----------
HsapiensGNAO1          ---------- ---------- ---------- ---------- ---------- ----------
HsapiensGNAQ           ---------- ---------- ---------- ---------- ---------- ----------
HsapiensGNAS1          MEGAADAAEG GKVPSPGYGS PAAGAASADT AARAAPAAPA DPDSGATPED PDSGTAPADP
HsapiensGNAS2          ---------- ---------- ---------- ---------- ---------- ----------
HsapiensGNAT1          ---------- ---------- ---------- ---------- ---------- ----------
HsapiensGNAT2          ---------- ---------- ---------- ---------- ---------- ----------
HsapiensGNAT3          ---------- ---------- ---------- ---------- ---------- ----------
HsapiensGNAZ           ---------- ---------- ---------- ---------- ---------- ----------
Ncrassagna1            ---------- ---------- ---------- ---------- ---------- ----------
Ncrassagna2            ---------- ---------- ---------- ---------- ---------- ----------
Ncrassagna3            ---------- ---------- ---------- ---------- ---------- ----------
ScervisiaeGPA1         ---------- ---------- ---------- ---------- ---------- ----------
ScervisiaeGPA2         ---------- ---------- ---------- ---------- ---------- ----------
SparadoxusGPA1         ---------- ---------- ---------- ---------- ---------- ----------
SparadoxusGPA2         ---------- ---------- ---------- ---------- ---------- ----------
Umaydisgpa1            ---------- ---------- ---------- ---------- ---------- ----------
Umaydisgpa2            ---------- ---------- ---------- ---------- ---------- ----------
Umaydisgpa3            ---------- ---------- ---------- ---------- ---------- ----------
Umaydisgpa4            ---------- ---------- ---------- ---------- ---------- ----------
```

```
                     421
AthalianaGPA1         ---------- ---------- ---------- ---------- ---------- ----------
CalbicansGPA1         ---------- ---------- ---------- ---------- ---------- ----------
CalbicansGPA2         ---------- ---------- ---------- ---------- ---------- ----------
Celeganseg130         ---------- ---------- ---------- ---------- ---------- ----------
Celegansgoa1          ---------- ---------- ---------- ---------- ---------- ----------
Celegansgpa1          ---------- ---------- ---------- ---------- ---------- ----------
Celegansgpa10         ---------- ---------- ---------- ---------- ---------- ----------
Celegansgpa11         ---------- ---------- ---------- ---------- ---------- ----------
Celegansgpa12         ---------- ---------- ---------- ---------- ---------- ----------
Celegansgpa13         ---------- ---------- ---------- ---------- ---------- ----------
Celegansgpa14         ---------- ---------- ---------- ---------- ---------- ----------
Celegansgpa15         ---------- ---------- ---------- ---------- ---------- ----------
Celegansgpa16         ---------- ---------- ---------- ---------- ---------- ----------
Celegansgpa17         ---------- ---------- ---------- ---------- ---------- ----------
Celegansgpa2          ---------- ---------- ---------- ---------- ---------- ----------
Celegansgpa3          ---------- ---------- ---------- ---------- ---------- ----------
Celegansgpa4          ---------- ---------- ---------- ---------- ---------- ----------
Celegansgpa5          ---------- ---------- ---------- ---------- ---------- ----------
Celegansgpa6          ---------- ---------- ---------- ---------- ---------- ----------
Celegansgpa7          ---------- ---------- ---------- ---------- ---------- ----------
Celegansgpa8          ---------- ---------- ---------- ---------- ---------- ----------
Celegansgpa9          ---------- ---------- ---------- ---------- ---------- ----------
Celegansgsa1          ---------- ---------- ---------- ---------- ---------- ----------
CglabrataGPA1         ---------- ---------- ---------- ---------- ---------- ----------
CglabrataGPA2         ---------- ---------- ---------- ---------- ---------- ----------
DmelanogasterCTA      ---------- ---------- ---------- ---------- ---------- ----------
DmelanogasterGalphaf  ---------- ---------- ---------- ---------- ---------- ----------
DmelanogasterGalphai  ---------- ---------- ---------- ---------- ---------- ----------
DmelanogasterGalphao  ---------- ---------- ---------- ---------- ---------- ----------
DmelanogasterGalphaq  ---------- ---------- ---------- ---------- ---------- ----------
DmelanogasterGalphas  ---------- ---------- ---------- ---------- ---------- ----------
Dreriognal1           ---------- ---------- ---------- ---------- ---------- ----------
Dreriognal1a          ---------- ---------- ---------- ---------- ---------- ----------
Dreriognal2           ---------- ---------- ---------- ---------- ---------- ----------
Dreriognal3           ---------- ---------- ---------- ---------- ---------- ----------
Dreriognal4a          ---------- ---------- ---------- ---------- ---------- ----------
Dreriognal2           ---------- ---------- ---------- ---------- ---------- ----------
Dreriognas            ---------- ---------- ---------- ---------- ---------- ----------
Dreriognav1           ---------- ---------- ---------- ---------- ---------- ----------
Dreriognaz            ---------- ---------- ---------- ---------- ---------- ----------
HsapiensGNA11         ---------- ---------- ---------- ---------- ---------- ----------
HsapiensGNA12         ---------- ---------- ---------- ---------- ---------- ----------
HsapiensGNA13         ---------- ---------- ---------- ---------- ---------- ----------
HsapiensGNA14         ---------- ---------- ---------- ---------- ---------- ----------
HsapiensGNA15         ---------- ---------- ---------- ---------- ---------- ----------
HsapiensGNAI1         ---------- ---------- ---------- ---------- ---------- ----------
HsapiensGNAI2         ---------- ---------- ---------- ---------- ---------- ----------
HsapiensGNAI3         ---------- ---------- ---------- ---------- ---------- ----------
HsapiensGNAL          ---------- ---------- ---------- ---------- ---------- ----------
HsapiensGNAO1         ---------- ---------- ---------- ---------- ---------- ----------
HsapiensGNAQ          ---------- ---------- ---------- ---------- ---------- ----------
HsapiensGNAS1         DSGAFAADPD SGAAPAAPAD PDSGAAPDAP ADPDSGAAPD APADPDAGAA PEAPAAPAAA
HsapiensGNAS2         ---------- ---------- ---------- ---------- ---------- ----------
HsapiensGNAT1         ---------- ---------- ---------- ---------- ---------- ----------
HsapiensGNAT2         ---------- ---------- ---------- ---------- ---------- ----------
HsapiensGNAT3         ---------- ---------- ---------- ---------- ---------- ----------
HsapiensGNAZ          ---------- ---------- ---------- ---------- ---------- ----------
Ncrassagna1           ---------- ---------- ---------- ---------- ---------- ----------
Ncrassagna2           ---------- ---------- ---------- ---------- ---------- ----------
Ncrassagna3           ---------- ---------- ---------- ---------- ---------- ----------
ScervisiaeGPA1        ---------- ---------- ---------- ---------- ---------- ----------
ScervisiaeGPA2        ---------- ---------- ---------- ---------- ---------- ----------
SparadoxusGPA1        ---------- ---------- ---------- ---------- ---------- ----------
SparadoxusGPA2        ---------- ---------- ---------- ---------- ---------- ----------
Umaydisgpa1           ---------- ---------- ---------- ---------- ---------- ----------
Umaydisgpa2           ---------- ---------- ---------- ---------- ---------- ----------
Umaydisgpa3           ---------- ---------- ---------- ---------- ---------- ----------
Umaydisgpa4           ---------- ---------- ---------- ---------- ---------- ----------
```

```
                           481
AthalianaGPA1              ---------- ---------- ---------- ---------- ---------- ----------
CalbicansGPA1              ---------- ---------- ---------- ---------- ---------- ----------
CalbicansGPA2              ---------- ---------- ---------- ---------- ---------- ----------
Celeganseg130              ---------- ---------- ---------- ---------- ---------- ----------
Celegansgoa1               ---------- ---------- ---------- ---------- ---------- ----------
Celegansgpa1               ---------- ---------- ---------- ---------- ---------- ----------
Celegansgpa10              ---------- ---------- ---------- ---------- ---------- ----------
Celegansgpa11              ---------- ---------- ---------- ---------- ---------- ----------
Celegansgpa12              ---------- ---------- ---------- ---------- ---------- ----------
Celegansgpa13              ---------- ---------- ---------- ---------- ---------- ----------
Celegansgpa14              ---------- ---------- ---------- ---------- ---------- ----------
Celegansgpa15              ---------- ---------- ---------- ---------- ---------- ----------
Celegansgpa16              ---------- ---------- ---------- ---------- ---------- ----------
Celegansgpa17              ---------- ---------- ---------- ---------- ---------- ----------
Celegansgpa2               ---------- ---------- ---------- ---------- ---------- ----------
Celegansgpa3               ---------- ---------- ---------- ---------- ---------- ----------
Celegansgpa4               ---------- ---------- ---------- ---------- ---------- ----------
Celegansgpa5               ---------- ---------- ---------- ---------- ---------- ----------
Celegansgpa6               ---------- ---------- ---------- ---------- ---------- ----------
Celegansgpa7               ---------- ---------- ---------- ---------- ---------- ----------
Celegansgpa8               ---------- ---------- ---------- ---------- ---------- ----------
Celegansgpa9               ---------- ---------- ---------- ---------- ---------- ----------
Celegansgsa1               ---------- ---------- ---------- ---------- ---------- ----------
CglabrataGPA1              ---------- ---------- ---------- ---------- ---------- ----------
CglabrataGPA2              ---------- ---------- ---------- ---------- ---------- ----------
DmelanogasterCTA           ---------- ---------- ---------- ---------- --------MS GITLTKLTQE
DmelanogasterGalphaf       ---------- ---------- ---------- ---------- ---------- ----------
DmelanogasterGalphai       ---------- ---------- ---------- ---------- ---------- ----------
DmelanogasterGalphao       ---------- ---------- ---------- ---------- ---------- ----------
DmelanogasterGalphaq       ---------- ---------- ---------- ---------- ---------- ----------
DmelanogasterGalphas       ---------- ---------- ---------- ---------- ---------- ----------
Dreriogna11                ---------- ---------- ---------- ---------- ---------- ----------
Dreriogna11a               ---------- ---------- ---------- ---------- ---------- ----------
Dreriogna12                ---------- ---------- ---------- ---------- ---------- ----------
Dreriogna13                ---------- ---------- ---------- ---------- ---------- ----------
Dreriogna14a               ---------- ---------- ---------- ---------- ---------- ----------
Dreriogna12                ---------- ---------- ---------- ---------- ---------- ----------
Dreriognas                 ---------- ---------- ---------- ---------- ---------- ----------
Dreriognav1                ---------- ---------- ---------- ---------- ---------- ----------
Dreriognaz                 ---------- ---------- ---------- ---------- ---------- ----------
HsapiensGNA11              ---------- ---------- ---------- ---------- ---------- ----------
HsapiensGNA12              ---------- ---------- ---------- ---------- ---------- ----------
HsapiensGNA13              ---------- ---------- ---------- ---------- ---------- ----------
HsapiensGNA14              ---------- ---------- ---------- ---------- ---------- ----------
HsapiensGNA15              ---------- ---------- ---------- ---------- ---------- ----------
HsapiensGNAI1              ---------- ---------- ---------- ---------- ---------- ----------
HsapiensGNAI2              ---------- ---------- ---------- ---------- ---------- ----------
HsapiensGNAI3              ---------- ---------- ---------- ---------- ---------- ----------
HsapiensGNAL               ---------- ---------- ---------- ---------- ---------- ----------
HsapiensGNAO1              ---------- ---------- ---------- ---------- ---------- ----------
HsapiensGNAQ               ---------- ---------- ---------- ---------- ---------- ----------
HsapiensGNAS1              ETRAAHVAPA APDAGAPTAP AASATRAAQV RRAASAAPAS GARRKIHLRP PSPEIQAADP
HsapiensGNAS2              ---------- ---------- ---------- ---------- ---------- ----------
HsapiensGNAT1              ---------- ---------- ---------- ---------- ---------- ----------
HsapiensGNAT2              ---------- ---------- ---------- ---------- ---------- ----------
HsapiensGNAT3              ---------- ---------- ---------- ---------- ---------- ----------
HsapiensGNAZ               ---------- ---------- ---------- ---------- ---------- ----------
Ncrassagna1                ---------- ---------- ---------- ---------- ---------- ----------
Ncrassagna2                ---------- ---------- ---------- ---------- ---------- ----------
Ncrassagna3                ---------- ---------- ---------- ---------- ---------- ----------
ScervisiaeGPA1             ---------- ---------- ---------- ---------- ---------- ----------
ScervisiaeGPA2             ---------- ---------- ---------- ---------- ---------- ----------
SparadoxusGPA1             ---------- ---------- ---------- ---------- ---------- ----------
SparadoxusGPA2             ---------- ---------- ---------- ---------- ---------- ----------
Umaydisgpa1                ---------- ---------- ---------- ---------- ---------- ----------
Umaydisgpa2                ---------- ---------- ---------- ---------- ---------- ----------
Umaydisgpa3                ---------- ---------- ---------- ---------- ---------- ----------
Umaydisgpa4                ---------- ---------- ---------- ---------- ---------- ----------
```

```
                          541
AthalianaGPA1             ---------- ---------- ---------- ---------- ---------- ----------
CalbicansGPA1             ---------- ---------- ---------- ---------- ---------- ----------
CalbicansGPA2             ---------- ---------- ---------- ---------- ---------- ----------
Celeganseg130             ---------- ---------- ---------- ---------- ---------- ----------
Celegansgoa1              ---------- ---------- ---------- ---------- ---------- ----------
Celegansgpa1              ---------- ---------- ---------- ---------- ---------- ----------
Celegansgpa10             ---------- ---------- ---------- ---------- ---------- ----------
Celegansgpa11             ---------- ---------- ---------- ---------- ---------- ----------
Celegansgpa12             ---------- ---------- ---------- ---------- ---------- ----------
Celegansgpa13             ---------- ---------- ---------- ---------- ---------- ----------
Celegansgpa14             ---------- ---------- ---------- ---------- ---------- ----------
Celegansgpa15             ---------- ---------- ---------- ---------- ---------- ----------
Celegansgpa16             ---------- ---------- ---------- ---------- ---------- ----------
Celegansgpa17             ---------- ---------- ---------- ---------- ---------- ----------
Celegansgpa2              ---------- ---------- ---------- ---------- ---------- ----------
Celegansgpa3              ---------- ---------- ---------- ---------- ---------- ----------
Celegansgpa4              ---------- ---------- ---------- ---------- ---------- ----------
Celegansgpa5              ---------- ---------- ---------- ---------- ---------- ----------
Celegansgpa6              ---------- ---------- ---------- ---------- ---------- ----------
Celegansgpa7              ---------- ---------- ---------- ---------- ---------- ----------
Celegansgpa8              ---------- ---------- ---------- ---------- ---------- ----------
Celegansgpa9              ---------- ---------- ---------- ---------- ---------- ----------
Celegansgsa1              ---------- ---------- ---------- ---------- ---------- ----------
CglabrataGPA1             ---------- ---------- ---------- ---------- ---------- ----------
CglabrataGPA2             ---------- ---------- ---------- ---------- ---------- ----------
DmelanogasterCTA          RISIPNNNVI TNGVENNIDS DTLSGTLTHL MEEHRTRVGA VTGPEAATTS TDGLISNGAE
DmelanogasterGalphaf      ---------- ---------- ---------- ---------- ---------- ----------
DmelanogasterGalphai      ---------- ---------- ---------- ---------- ---------- ----------
DmelanogasterGalphao      ---------- ---------- ---------- ---------- ---------- ----------
DmelanogasterGalphaq      ---------- ---------- ---------- ---------- ---------- ----------
DmelanogasterGalphas      ---------- ---------- ---------- ---------- ---------- ----------
Dreriogna11               ---------- ---------- ---------- ---------- ---------- ----------
Dreriogna11a              ---------- ---------- ---------- ---------- ---------- ----------
Dreriogna12               ---------- ---------- ---------- ---------- ---------- ----------
Dreriogna13               ---------- ---------- ---------- ---------- ---------- ----------
Dreriogna14a              ---------- ---------- ---------- ---------- ---------- ----------
Dreriogna12               ---------- ---------- ---------- ---------- ---------- ----------
Dreriognas                ---------- ---------- ---------- ---------- ---------- ----------
Dreriognav1               ---------- ---------- ---------- ---------- ---------- ----------
Dreriognaz                ---------- ---------- ---------- ---------- ---------- ----------
HsapiensGNA11             ---------- ---------- ---------- ---------- ---------- ----------
HsapiensGNA12             ---------- ---------- ---------- ---------- ---------- ----------
HsapiensGNA13             ---------- ---------- ---------- ---------- ---------- ----------
HsapiensGNA14             ---------- ---------- ---------- ---------- ---------- ----------
HsapiensGNA15             ---------- ---------- ---------- ---------- ---------- ----------
HsapiensGNAI1             ---------- ---------- ---------- ---------- ---------- ----------
HsapiensGNAI2             ---------- ---------- ---------- ---------- ---------- ----------
HsapiensGNAI3             ---------- ---------- ---------- ---------- ---------- ----------
HsapiensGNAL              ---------- ---------- ---------- ---------- ---------- ----------
HsapiensGNAO1             ---------- ---------- ---------- ---------- ---------- ----------
HsapiensGNAQ              ---------- ---------- ---------- ---------- ---------- ----------
HsapiensGNAS1             PTPRPTRASA WRGKSESSRG RRVYYDEGVA SSDDDSSGDE SDDGTSGCLR WFQHRRNRRR
HsapiensGNAS2             ---------- ---------- ---------- ---------- ---------- ----------
HsapiensGNAT1             ---------- ---------- ---------- ---------- ---------- ----------
HsapiensGNAT2             ---------- ---------- ---------- ---------- ---------- ----------
HsapiensGNAT3             ---------- ---------- ---------- ---------- ---------- ----------
HsapiensGNAZ              ---------- ---------- ---------- ---------- ---------- ----------
Ncrassagna1               ---------- ---------- ---------- ---------- ---------- ----------
Ncrassagna2               ---------- ---------- ---------- ---------- ---------- ----------
Ncrassagna3               ---------- ---------- ---------- ---------- ---------- ----------
ScervisiaeGPA1            ---------- ---------- ---------- ---------- ---------- ----------
ScervisiaeGPA2            ---------- ---------- ---------- ---------- ---------- ----------
SparadoxusGPA1            ---------- ---------- ---------- ---------- ---------- ----------
SparadoxusGPA2            ---------- ---------- ---------- ---------- ---------- ----------
Umaydisgpa1               ---------- ---------- ---------- ---------- ---------- ----------
Umaydisgpa2               ---------- ---------- ---------- ---------- ---------- ----------
Umaydisgpa3               ---------- ---------- ---------- ---------- ---------- ----------
Umaydisgpa4               ---------- ---------- ---------- ---------- ---------- ----------
```

```
                          601
AthalianaGPA1             ---------- ------MGL- -----LC--- ---------- ---------- ------SRSR
CalbicansGPA1             ---------- ------MGC- -G---AS--- ---------- ---------- ----------
CalbicansGPA2             ---------- ------MGS- -CASKSA--- ---------- ------DQGN GTSKQQQSAQ
Caleganseg130             ---------- ------MAC- -C---LS--- ---------- ---------- ----------
Celegansgoa1              ---------- ------MGC- -T---MS--- ---------- ---------- ----------
Celegansgpa1              ---------- ------MGN- -C---ES--- ---------- ---------- ----------
Celegansgpa10             ---------- ------MGV- -C---QS--- ---------- ---------- --------FV
Celegansgpa11             ---------- ---------- -----MS--- ---------- ---------- ----------
Celegansgpa12             ---------- ------MVC- -C---FG--- ---------- ---------- ----------
Celegansgpa13             ---------- ------MGC- -N---FS--- ---------- ---------- ---------S
Celegansgpa14             -----MAFSC FDKFSYTYCY QCMHGPD--- ---------- -----GCMVP SRNGEGTELY
Celegansgpa15             ---------- ------MGS- -----TC--- ---------- ---------- ----------
Celegansgpa16             ---------- ------MGC- -I---MS--- ---------- ---------- ----------
Celegansgpa17             ---------- ------MGS- -C---QS--- ---------- ---------- ----------
Celegansgpa2              ---------- ------MGL- -C---QS--- ---------- ---------- ----------
Celegansgpa3              ---------- ------MGL- -C---QS--- ---------- ---------- ----------
Celegansgpa4              ---------- ------MGC- -----FH--- ---------- ---------- ----------
Celegansgpa5              ---------- ------MGLV TC-------- ---------- ---------- ----------
Celegansgpa6              ---------- ------MGA- -GATGLR--- ---------- ---------- -------GAR
Celegansgpa7              ---------- ------MGH- -C---TS--- ---------- ---------- ----------
Celegansgpa8              ---------- ------MGA- LC---SS--- ---------- ---------- ---------E
Celegansgpa9              ---------- ------MGS- -----AG--- ---------- ---------- ----------
Celegansgsa1              ---------- ---MRLMGC- -----VG--- ---------- ---------- --------AG
CglabrataGPA1             ---------- ------MGC- -I---IS--- ---------- ---------- ----------
CglabrataGPA2             ---------- ------MGI- -CGSKPDKAG DTKPGTSKAR VRSDNRNSGG ASNGKSTTAG
DmelanogasterCTA          RLRLQGSRLQ TSRFACFRC- -CGNIIT--- ---------- ---------- ----YLVRLR
DmelanogasterGalphaf      ---------- -MKLRLLRC- -----LR--- ---------- ---------- ----------
DmelanogasterGalphai      ---------- ------MGC- -A---VS--- ---------- ---------- ----------
DmelanogasterGalphao      ---------- ------MGC- -A---QS--- ---------- ---------- ----------
DmelanogasterGalphaq      ---------- ------MEC- -C---LS--- ---------- ---------- ----------
DmelanogasterGalphas      ---------- ------MGC- -----FG--- ---------- ---------- -SPTSKQSDV
Dreriogna11               ---------- MTLESMMAC- -C---MS--- ---------- ---------- ----------
Dreriogna11a              ---------- MTLESMMAC- -C---MS--- ---------- ---------- ----------
Dreriogna12               --------MA GVVRTLSRC- -----LL--- ---------- -PAEAAHDDR STGKDRTRER
Dreriogna13               --MADFLPSR TAIVCIPNC- -L---LS--- ---------- ---------- ----------
Dreriogna14a              ---------- -----MAGC- -C---MS--- ---------- ---------- ----------
Dreriogna12               ---------- -----MMGC- -----LG--- ---------- ---------- ----SSKTED
Dreriognas                ---------- ------MGM- -CHSFRSRLS ESAITYTGGR SAAGAGIDAE SGGHSDSCDP
Dreriognav1               ---------- ------MGL- -C---LG--- ---------- ---------- -------SEV
Dreriognaz                ---------- ------MGC- -R---QS--- ---------- ---------- ----------
HsapiensGNA11             ---------- MTLESMMAC- -C---LS--- ---------- ---------- ----------
HsapiensGNA12             --------MS GVVRTLSRC- -L---LP--- ---------- ------AEAG GARERRAGSG
HsapiensGNA13             -MADFLPSRS VLSVCFPGC- -L---LT--- ---------- ---------- ----------
HsapiensGNA14             ---------- -----MAGC- -CC--LS--- ---------- ---------- ----------
HsapiensGNA15             ---------- MARSLTWRC- -CPWCLT--- ---------- ---------- ----------
HsapiensGNAI1             ---------- ------MGC- -T---LS--- ---------- ---------- ----------
HsapiensGNAI2             ---------- ------MGC- -T---VS--- ---------- ---------- ----------
HsapiensGNAI3             ---------- ------MGC- -T---LS--- ---------- ---------- ----------
HsapiensGNAL              ---------- ------MGC- -----LG--- ---------- ---------- --GNSKTTED
HsapiensGNAO1             ---------- ------MGC- -T---LS--- ---------- ---------- ----------
HsapiensGNAQ              ---------- MTLESIMAC- -C---LS--- ---------- ---------- ----------
HsapiensGNAS1             RKPQRNLLRN FLVQAFGGC- -----FGRSE SPQPKASRSL KVKKVPLAEK RRQMRKEALE
HsapiensGNAS2             ---------- ------MGC- -----LG--- ---------- ---------- ----NSKTED
HsapiensGNAT1             ---------- ------MGA- -G---AS--- ---------- ---------- ----------
HsapiensGNAT2             ---------- ------MGS- -G---AS--- ---------- ---------- ----------
HsapiensGNAT3             ---------- ------MGS- -G---IS--- ---------- ---------- ----------
HsapiensGNAZ              ---------- ------MGC- -R---QS--- ---------- ---------- ----------
Ncrassagna1               ---------- ------MGC- -G---MS--- ---------- ---------- ----------
Ncrassagna2               ---------- --------M- -C---FG--- ---------- ---------- ---------G
Ncrassagna3               ---------- ------MGA- -C---MS--- ---------- ---------- ----------
ScervisiaeGPA1            ---------- ------MGC- -T---VS--- ---------- ---------- ----------
ScervisiaeGPA2            ---------- ------MGL- -C---AS--- ---------- ----SEKNGS TPDTQTASAG
SparadoxusGPA1            ---------- ------MGC- -T---VS--- ---------- ---------- ----------
SparadoxusGPA2            ---------- ------MGL- -CASSEK--- ---------- ---NGSTPDT QTASAGSDNA
Umaydisgpa1               ---------- ------MGC- -G---AS--- ---------- ---------- ----------
Umaydisgpa2               ---------- ------MGA- -C---LS--- ---------- ---------- ---------A
Umaydisgpa3               ---------- ------MGN- -C---LS--- ---------- ---------- ----------
Umaydisgpa4               ----MSPSVS SPQLRHTKSN RAISRID--- ---------- ------RTDP LALALQPPAN
```

```
                    661
AthalianaGPA1        HHTEDT--D- ------ENTQ AAEIERRIEQ EAKAEK---- ---------- ----------
CalbicansGPA1        -VPVDD--DE IDPFLQDKRI NDAIEQSLQL RQQNSK---- ---------- ----------
CalbicansGPA2        STQGKR--QQ QSQAIQQKNK EKQQSRQKEQ QEKHQQQQQQ QPQPQSPSDS NADGTLKNGS
Celeganseg130        --EEAR--E- ------QKRI NQEIEKQLQR DKRNAR---- ---------- ----------
Celegansgoa1         --QEER--A- ------ALER SRMIEKNLKE DGMQAA---- ---------- ----------
Celegansgpa1         --RELV--A- ------QAKQ NKIINTELDK AKKTDE---- ---------- ----------
Celegansgpa10        LTDEMK--D- ------QIKV NKEIEKQLEK KKNMQL---- ---------- ----------
Celegansgpa11        ----AA--D- ------MARK NSLINRQLEK EKIDSK---- ---------- ----------
Celegansgpa12        --------K- ------KDER TKTIEKELHK ERKIMR---- ---------- ----------
Celegansgpa13        QSKLQV--P- ------EIRA SRSITPASQK SEDPY----- ---------- ----------
Celegansgpa14        AHSEEL--EA KLREL-ARRG HMEIEKELAL EKKTYG---- ---------- ----------
Celegansgpa15        STPESK--E- ------QKRI NSVIDKQIRK DEDDEI---- ---------- ----------
Celegansgpa16        --QEDE--A- ------AKRR SKKIDRLLKE DGENSM---- ---------- ----------
Celegansgpa17        --NENS--E- ------GNAR NKEIEKQLNA DKRAGS---- ---------- ----------
Celegansgpa2         --EEEK--V- ------GTLK SRAIDKEIKQ LQTSEE---- ---------- ----------
Celegansgpa3         --AEDK--E- ------LTLK SKAIDKEMMQ NHMSQQ---- ---------- ----------
Celegansgpa4         --STGS--E- ------AKKR SKLIDEQLRH DHERCV---- ---------- ----------
Celegansgpa5         --KQER--E- ------AELQ NRQIDTQIRI ENQANK---- ---------- ----------
Celegansgpa6         LSPEER--A- ------NSSK SRAIDRALSK DHTDDL---- ---------- ----------
Celegansgpa7         --KDQK--E- ------GKRL NRRIDEQIKK DQSMSL---- ---------- ----------
Celegansgpa8         TYMLDK--EE YKK---QVEH NKSIENDLEK DRKI------ ---------- ----------
Celegansgpa9         ---------- ---------- --SVDR---- ---------- ---------- ----------
Celegansgsa1         ADAEGR--E- ------ARKV NKQIEEQLAK DKQVMR---- ---------- ----------
CglabrataGPA1        -TSSNE--DE NDPFIKNKRA NDIIEQSLLK ERQKEK---- ---------- ----------
CglabrataGPA2        QKDGQD--ME GGQSGNSMSN EGDKKTTLEE ELQNTRGLPV DEQSSSQSGS NNQ-------
DmelanogasterCTA     STPEEL--E- ------QRYK SKEIDKFLEK EKHTFR---- ---------- ----------
DmelanogasterGalphaf -QQPAK--PA AVMTHKEDQY PVSLDHHVLK DMAKGVRD-- ---------- ----------
DmelanogasterGalphai -TARDK--E- ------AIER SKNIDRALRA EGERAA---- ---------- ----------
DmelanogasterGalphao --AEER--A- ------AAAR SRLIERNLKE DGIQAA---- ---------- ----------
DmelanogasterGalphaq --EEAK--E- ------QKRI NQEIEKQLRR DKRDAR---- ---------- ----------
DmelanogasterGalphas NSEDSK--S- ------QKRR SDAISRQLQK DKQLYR---- ---------- ----------
Dreriogna11          --EEAK--E- ------SKRI NAEIDKQLRR DKRDAR---- ---------- ----------
Dreriogna11a         --EEAK--E- ------SKRI NAEIDKQLRR DKRDAR---- ---------- ----------
Dreriogna12          DVNRER--E- ------AKRR SREIDSMLKR ERRSIR---- ---------- ----------
Dreriogna13          --SGEI--D- ------QIRK SKEIDKSLSR EKTYVK---- ---------- ----------
Dreriogna14a         --AEEK--E- ------RQRI NQEIDKQLRK DKKDSR---- ---------- ----------
Dreriogna12          QRIDEK--A- ------QREA NKKIEKQLQK ERQAYR---- ---------- ----------
Dreriognas           QLRERRRAEE RARVKAENKR SRNIDKSLKA EKREYK---- ---------- ----------
Dreriognav1          TTEEDK--K- ------AKIH SSQIDRDLYE YAKREL---- ---------- ----------
Dreriognaz           --TEEK--E- ------AARR SRRIDRHLRS ESQRQR---- ---------- ----------
HsapiensGNA11        --DEVK--E- ------SKRI NAEIEKQLRR DKRDAR---- ---------- ----------
HsapiensGNA12        ARDAER--E- ------ARRR SRDIDALLAR ERRAVR---- ---------- ----------
HsapiensGNA13        --SGEA--E- ------QQRK SKEIDKCLSR EKTYVK---- ---------- ----------
HsapiensGNA14        --AEEK--E- ------SQRI SAEIERQLRR DKKDAR---- ---------- ----------
HsapiensGNA15        --EDEK--A- ------AARV DQEINRILLE QKKQDR---- ---------- ----------
HsapiensGNAI1        --AEDK--A- ------AVER SKMIDRNLRE DGEKAA---- ---------- ----------
HsapiensGNAI2        --AEDK--A- ------AAER SKMIDKNLRE DGEKAA---- ---------- ----------
HsapiensGNAI3        --AEDK--A- ------AVER SKMIDRNLRE DGEKAA---- ---------- ----------
HsapiensGNAL         QGVDEK--E- ------RREA NKKIEKQLQK ERLAYK---- ---------- ----------
HsapiensGNAO1        --AEER--A- ------ALER SKAIEKNLKE DGISAA---- ---------- ----------
HsapiensGNAQ         --EEAK--E- ------ARRI NDEIERQLRR DKRDAR---- ---------- ----------
HsapiensGNAS1        KRAQKR--A- ------EKKR SKLIDKQLQD EKMGYM---- ---------- ----------
HsapiensGNAS2        QRNEEK--A- ------QREA NKKIEKQLQK DKQVYR---- ---------- ----------
HsapiensGNAT1        --------A- ------EEKH SRELEKKLKE DAEKDA---- ---------- ----------
HsapiensGNAT2        --AEDK--E- ------LAKR SKELEKKLQE DADKEA---- ---------- ----------
HsapiensGNAT3        --SESK--E- ------SAKR SKELEKKLQE DAERDA---- ---------- ----------
HsapiensGNAZ         --SEEK--E- ------AARR SRRIDRHLRS ESQRQR---- ---------- ----------
Ncrassagna1          --TEEK--E- ------GKAR NEEIENQLKR DRMQQR---- ---------- ----------
Ncrassagna2          RGKDDE--A- ------EASR SRELDKQIRA DEKRLS---- ---------- ----------
Ncrassagna3          KNDEET--E- ------QKKR SQKIDRDLEE DSKKLR---- ---------- ----------
ScervisiaeGPA1       -TQTIG--DE SDPFLQNKRA NDVIEQSLQL EKQRDK---- ---------- ----------
ScervisiaeGPA2       SDNVGK--AK VPPKQEPQKT VRTVNTANQQ EKQQQRQQQP SPHNVKDRKE QNGSINNAIS
SparadoxusGPA1       -TQTID--DE SDPFLQNKRA NDVIEQSLQL EKQRDK---- ---------- ----------
SparadoxusGPA2       GRAKVS--AQ QQPQRQPQKT VRTVNTASQQ EKQQQQQQQQ PFARNVKDNN TSINNAISPT
Umaydisgpa1          --KVDK--E- ------GQAR NDAIDAQLKK DRLAQK---- ---------- ----------
Umaydisgpa2          EQSHDT--P- ------EYKR SKALDRRIKE DEKNLS---- ---------- ----------
Umaydisgpa3          -SSDQK--E- ------AKDR SVAIDKQIEE DSRKFK---- ---------- ----------
Umaydisgpa4          EAPADKY-AR LHQEKLAKQR SDEIDKFLKQ HQEDRATHGL ASSPSTSVDG ANFKKG----
```

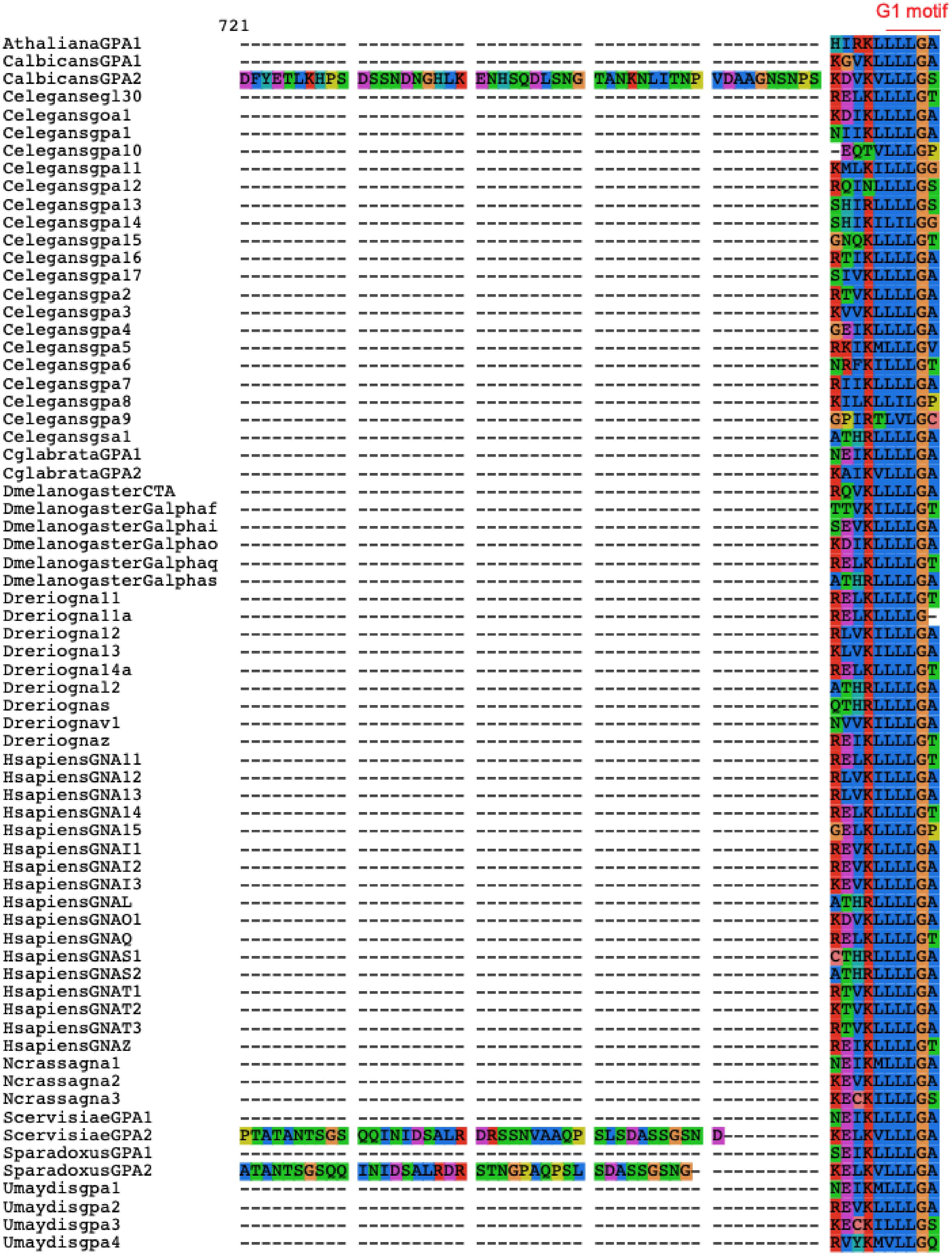
G1 motif
721
AthalianaGPA1 ---------- ---------- ---------- ---------- ---------- HIRKLLLLGA
CalbicansGPA1 ---------- ---------- ---------- ---------- ---------- KGVKLLLLGA
CalbicansGPA2 DFYETLKHPS DSSNDNGHLK ENHSQDLSNG TANKNLITNP VDAAGNSNPS KDVKVLLLGS
Celegansegl30 ---------- ---------- ---------- ---------- ---------- RELKLLLLGT
Celegansgoa1 ---------- ---------- ---------- ---------- ---------- KDIKLLLLGA
Celegansgpa1 ---------- ---------- ---------- ---------- ---------- NIIKLLLLGA
Celegansgpa10 ---------- ---------- ---------- ---------- ---------- -EQTVLLLGP
Celegansgpa11 ---------- ---------- ---------- ---------- ---------- KMLKILLLGG
Celegansgpa12 ---------- ---------- ---------- ---------- ---------- RQINLLLLGS
Celegansgpa13 ---------- ---------- ---------- ---------- ---------- SHIRLLLLGS
Celegansgpa14 ---------- ---------- ---------- ---------- ---------- SHIKILILGG
Celegansgpa15 ---------- ---------- ---------- ---------- ---------- GNQKLLLLGT
Celegansgpa16 ---------- ---------- ---------- ---------- ---------- RTIKLLLLGA
Celegansgpa17 ---------- ---------- ---------- ---------- ---------- SIVKLLLLGA
Celegansgpa2 ---------- ---------- ---------- ---------- ---------- RTVKLLLLGA
Celegansgpa3 ---------- ---------- ---------- ---------- ---------- KVVKLLLLGA
Celegansgpa4 ---------- ---------- ---------- ---------- ---------- GEIKLLLLGA
Celegansgpa5 ---------- ---------- ---------- ---------- ---------- RKIKMLLLGV
Celegansgpa6 ---------- ---------- ---------- ---------- ---------- NRFKILLLGT
Celegansgpa7 ---------- ---------- ---------- ---------- ---------- RIIKLLLLGA
Celegansgpa8 ---------- ---------- ---------- ---------- ---------- KILKLLILGP
Celegansgpa9 ---------- ---------- ---------- ---------- ---------- GPIRTLVLGC
Celegansgsa1 ---------- ---------- ---------- ---------- ---------- ATHRLLLLGA
CglabrataGPA1 ---------- ---------- ---------- ---------- ---------- NEIKLLLLGA
CglabrataGPA2 ---------- ---------- ---------- ---------- ---------- KAIKVLLLGA
DmelanogasterCTA ---------- ---------- ---------- ---------- ---------- RQVKLLLLGA
DmelanogasterGalphaf ---------- ---------- ---------- ---------- ---------- TTVKILLLGT
DmelanogasterGalphai ---------- ---------- ---------- ---------- ---------- SEVKLLLLGA
DmelanogasterGalphao ---------- ---------- ---------- ---------- ---------- KDIKLLLLGA
DmelanogasterGalphaq ---------- ---------- ---------- ---------- ---------- RELKLLLLGT
DmelanogasterGalphas ---------- ---------- ---------- ---------- ---------- ATHRLLLLGA
Dreriogna11 ---------- ---------- ---------- ---------- ---------- RELKLLLLGT
Dreriogna11a ---------- ---------- ---------- ---------- ---------- RELKLLLLG-
Dreriogna12 ---------- ---------- ---------- ---------- ---------- RLVKILLLGA
Dreriogna13 ---------- ---------- ---------- ---------- ---------- KLVKILLLGA
Dreriogna14a ---------- ---------- ---------- ---------- ---------- RELKLLLLGT
Dreriognal2 ---------- ---------- ---------- ---------- ---------- ATHRLLLLGA
Dreriognas ---------- ---------- ---------- ---------- ---------- QTHRLLLLGA
Dreriognav1 ---------- ---------- ---------- ---------- ---------- NVVKILLLGA
Dreriognaz ---------- ---------- ---------- ---------- ---------- REIKLLLLGT
HsapiensGNA11 ---------- ---------- ---------- ---------- ---------- RELKLLLLGT
HsapiensGNA12 ---------- ---------- ---------- ---------- ---------- RLVKILLLGA
HsapiensGNA13 ---------- ---------- ---------- ---------- ---------- RLVKILLLGA
HsapiensGNA14 ---------- ---------- ---------- ---------- ---------- RELKLLLLGT
HsapiensGNA15 ---------- ---------- ---------- ---------- ---------- GELKLLLLGP
HsapiensGNAI1 ---------- ---------- ---------- ---------- ---------- REVKLLLLGA
HsapiensGNAI2 ---------- ---------- ---------- ---------- ---------- REVKLLLLGA
HsapiensGNAI3 ---------- ---------- ---------- ---------- ---------- KEVKLLLLGA
HsapiensGNAL ---------- ---------- ---------- ---------- ---------- ATHRLLLLGA
HsapiensGNAO1 ---------- ---------- ---------- ---------- ---------- KDVKLLLLGA
HsapiensGNAQ ---------- ---------- ---------- ---------- ---------- RELKLLLLGT
HsapiensGNAS1 ---------- ---------- ---------- ---------- ---------- CTHRLLLLGA
HsapiensGNAS2 ---------- ---------- ---------- ---------- ---------- ATHRLLLLGA
HsapiensGNAT1 ---------- ---------- ---------- ---------- ---------- RTVKLLLLGA
HsapiensGNAT2 ---------- ---------- ---------- ---------- ---------- KTVKLLLLGA
HsapiensGNAT3 ---------- ---------- ---------- ---------- ---------- RTVKLLLLGA
HsapiensGNAZ ---------- ---------- ---------- ---------- ---------- REIKLLLLGT
Ncrassagna1 ---------- ---------- ---------- ---------- ---------- NEIKMLLLGA
Ncrassagna2 ---------- ---------- ---------- ---------- ---------- KEVKLLLLGA
Ncrassagna3 ---------- ---------- ---------- ---------- ---------- KECKILLLGS
ScervisiaeGPA1 ---------- ---------- ---------- ---------- ---------- NEIKLLLLGA
ScervisiaeGPA2 PTATANTSGS QQINIDSALR DRSSNVAAQP SLSDASSGSN D--------- KELKVLLLGA
SparadoxusGPA1 ---------- ---------- ---------- ---------- ---------- SEIKLLLLGA
SparadoxusGPA2 ATANTSGSQQ INIDSALRDR STNGPAQPSL SDASSGSNG- ---------- KELKVLLLGA
Umaydisgpa1 ---------- ---------- ---------- ---------- ---------- NEIKMLLLGA
Umaydisgpa2 ---------- ---------- ---------- ---------- ---------- REVKLLLLGA
Umaydisgpa3 ---------- ---------- ---------- ---------- ---------- KECKILLLGS
Umaydisgpa4 ---------- ---------- ---------- ---------- ---------- RVYKMVLLGQ

G1 motif

781 ← G132

```
AthalianaGPA1          GESGKSTIFK QIKLLFQTG- FDEG------ ---------E LKSYVPVIHA NVYQTIKLLH
CalbicansGPA1          GESGKSTVLK QLKLLHKGG- FTQQ------ ---------E RRQYSHVIWC DVIQSMKVLI
CalbicansGPA2          GESGKSTIVK QMKILHSDG- YTQD------ ---------E LEEYRPFVYK NILDCIKNVI
Celeganseg130          GESGKSTFIK QMRIIHGQG- YSEE------ ---------D KRAHIRLVYQ NVFMAIQSMI
Celegansgoa1           GESGKSTIVK QMKIIHESG- FTAE------ ---------D YKQYKPVVYS NTVQSLVAIL
Celegansgpa1           GESGKSTVLK QMKIIHNSG- FSQE------ ---------E ISNKRNVVCA NTVQAMGALL
Celegansgpa10          GESGKSTVMK QMRAMTGN-- YTKT------ ---------E LHERKVLIIQ NLCQFSEMLL
Celegansgpa11          PECGKSTIFK QMKIIHMNG- FSDL------ ---------D YVNFRYLIYS NIMQSMDQLL
Celegansgpa12          GESGKSTFVK QMHIIHGAGE FTAD------ ---------E VRAYRQQIYQ NAISAMRVLL
Celegansgpa13          AESGKTTVLE QVRLLYKQH- FTES------ ---------E YFHRRAFIYH NIFKSIKALC
Celegansgpa14          PLSGKSTIFK QMQIIHVDGF KTDQ------ ---------E LIQYRGLIDN NIRDIYLQLI
Celegansgpa15          GECGKSTILK QINILHSSG- FSKA------ ---------D LKNVAGTVYS NIIQGVATLL
Celegansgpa16          GESGKSTILK QMRIIHDVG- YTTE------ ---------E RKVFRGVVYG NIILSLNAII
Celegansgpa17          GECGKSTVLK QMQILHSNG- FTEE------ ---------E VNEKRAIVYN NTVSAMCTIL
Celegansgpa2           GECGKSTVLK QMRLLTSKQ- YTDE------ ---------E LLTQAKLVYT NIVIEMDHLV
Celegansgpa3           GECGKSTVLK QMRILHDHG- FTAE------ ---------E AEQQKSVVFN NTLQAMTAIL
Celegansgpa4           GESGKSTIVR QMRILHETG- FNKQ------ ---------E QMAYRPVVFS NMVQSMLAIL
Celegansgpa5           TDSGKSTIVK QMRVNYLDG- FNET------ ---------E VVNAIFVIRN NIIDAFKNIC
Celegansgpa6           AESGKSTIFK QMRVLHLDG- YAKE------ ---------D ALEYLSIIHS NCMEALTQLV
Celegansgpa7           GESGKSTILK QMRILHKDG- FSQQ------ ---------D LEMIRPVVYS NCIHSMLSIL
Celegansgpa8           GESGKSTTIK QIKIIHDEG- YSAE------ ---------E KMVRKHGIYM NILEGIEEIH
Celegansgpa9           MGAGKTSFIR QMVKNHTKS- ICLR------ ---------- RELYLYCVQL NLLNIYRELK
Celegansgsa1           GESGKSTIVK QMRILHING- FNEA------ ---------E KREKITDIRR NVRDAMQVIL
CglabrataGPA1          GESGKSTVLK QLKLLHKGG- FSHQ------ ---------E RLQYAQVIWA DVIQSMKILI
CglabrataGPA2          GESGKSTILQ QLKILHQNG- FTKQ------ ---------E LIDYTPLIYD NIIEIGKDII
DmelanogasterCTA       GESGKSTFLK QMRIIHGVN- FDYE------ ---------L LLEYQSVIYQ NVIRGMQVLL
DmelanogasterGalphaf   AESGKTTIIK QMRILHING- FTDD------ ---------E RREKIPEIYQ NIHESILQLV
DmelanogasterGalphai   GESGKSTIVK QMKIIHDTG- YSQE------ ---------E CEEYRRVVFS NTVQSLMVII
DmelanogasterGalphao   GESGKSTIVK QMKIIHESG- FTAE------ ---------D FKQYRPVVYS NTIQSLVAIL
DmelanogasterGalphaq   GESGKSTFIK QMRIIHGSG- YSDE------ ---------D KRGYIKLVFQ NIFMAMQSMI
DmelanogasterGalphas   GESGKSTIVK QMRILHVDG- FSDS------ ---------E KKQKIDDIKK NIRDAILTIT
Dreriogna11            GESGKSTFIK QMRIIHGTG- YTDE------ ---------D KRGYTKLVYQ NIFTSMQAMI
Dreriogna11a           -ESGKSTFIK QMRIIHGTG- YTDE------ ---------D KRGYTKLVYQ NIFTSMQAMI
Dreriogna12            GESGKSTFLK QMRIIHGKE- FDQK------ ---------A LLDFRDTIFE NVIKGMRVLV
Dreriogna13            GESGKSTFLK QMRIIHGQD- FDQR------ ---------A KEEFRATIYS NVIKGVRVLV
Dreriogna14a           GESGKSTFIK QMRIIHGSG- YTDD------ ---------D KKGFIKLVHQ NTLSAMQSMV
Dreriogna12            GESGKSTIVK QMKILHVNG- FNAE------ ---------E KKQKIQDIRK NVKDAIVTIV
Dreriognas             GESGKSTIVK QMRILHVNG- FNAE------ ---------E KKQKIQDIKN NIKEAIETIV
Dreriognav1            AESGKSTLVK QMKIIHSHG- FTKQ------ ---------E LTSFKPAVLD NLLTSMKFVL
Dreriognaz             SNSGKSTIVK QMKIIHSGG- FNLE------ ---------A CKEYKPLILY NAIDSLTRII
HsapiensGNA11          GESGKSTFIK QMRIIHGAG- YSEE------ ---------D KRGFTKLVYQ NIFTAMQAMI
HsapiensGNA12          GESGKSTFLK QMRIIHGRE- FDQK------ ---------A LLEFRDTIFD NILKGSRVLV
HsapiensGNA13          GESGKSTFLK QMRIIHGQD- FDQR------ ---------A REEFRPTIYS NVIKGMRVLV
HsapiensGNA14          GESGKSTFIK QMRIIHGSG- YSDE------ ---------D RKGFTKLVYQ NIFTAMQAMI
HsapiensGNA15          GESGKSTFIK QMRIIHGAG- YSEE------ ---------E RKGFRPLVYQ NIFVSMRAMI
HsapiensGNAI1          GESGKSTIVK QMKIIHEAG- YSEE------ ---------E CKQYKAVVYS NTIQSIIAII
HsapiensGNAI2          GESGKSTIVK QMKIIHEDG- YSEE------ ---------E CRQYRAVVYS NTIQSIMAIV
HsapiensGNAI3          GESGKSTIVK QMKIIHEDG- YSED------ ---------E CKQYKVVVYS NTIQSIIAII
HsapiensGNAL           GESGKSTIVK QMRILHVNG- FNPE------ ---------E KKQKILDIRK NVKDAIVTIV
HsapiensGNAO1          GESGKSTIVK QMKIIHEDG- FSGE------ ---------D VKQYKPVVYS NTIQSLAAIV
HsapiensGNAQ           GESGKSTFIK QMRIIHGSG- YSDE------ ---------D KRGFTKLVYQ NIFTAMQAMI
HsapiensGNAS1          GESGKSTIVK QMRILHVNG- FNGEGGEEDP QAARSNSDGE KATKVQDIKN NLKEAIETIV
HsapiensGNAS2          GESGKSTIVK QMRILHVNG- FNGEGGEEDP QAARSNSDGE KATKVQDIKN NLKEAIETIV
HsapiensGNAT1          GESGKSTIVK QMKIIHQDG- YSLE------ ---------E CLEFIAIIYG NTLQSILAIV
HsapiensGNAT2          GESGKSTIVK QMKIIHQDG- YSPE------ ---------E CLEFKAIIYG NVLQSILAII
HsapiensGNAT3          GESGKSTIVK QMKIIHKNG- YSEQ------ ---------E CMEFKAVIYS NTLQSILAIV
HsapiensGNAZ           SNSGKSTIVK QMKIIHSGG- FNLE------ ---------A CKEYKPLIIY NAIDSLTRII
Ncrassagna1            GESGKSTILK QMKLIHEGG- YSRD------ ---------E RESFKEIIFS NTVQSMRVIL
Ncrassagna2            GESGKSTILK QMKLIYAQG- FSKN------ ---------E KLEWRPVIFA NILQSFRLIF
Ncrassagna3            GESGKSTIVK QMKIIHLKG- YSDE------ ---------E LTNYRPTVYK NLLECAKAVV
ScervisiaeGPA1         GESGKSTVLK QLKLLHQGG- FSHQ------ ---------E RLQYAQVIWA DAIQSMKILI
ScervisiaeGPA2         GESGKSTVLQ QLKILHQNG- FSEQ------ ---------E IKEYIPLIYQ NLLEIGRNLI
SparadoxusGPA1         GESGKSTVLK QLKLLHQGG- FSHQ------ ---------E RLQYAQVIWA DAIQSMKILI
SparadoxusGPA2         GESGKSTVLQ QLKILHQNG- FSEQ------ ---------E IKEYIPLIYQ NLLEIGRNII
Umaydisgpa1            GESGKSTILK QMKLINHGS- YSAE------ ---------E RESYKEIIFS NTVQSMRVLL
Umaydisgpa2            GESGKSTILK SMRIIHHIP- FTDE------ ---------E RENFRRLVFL NLVQGMKTIL
Umaydisgpa3            GESGKSTIVK QMKIIHQNG- YTKD------ ---------E LLLYRLTVIK NLVDSAQAMV
Umaydisgpa4            AGAGKTTVLK QMRLLYDPP- AHER------ ---------E RRGWTKIVLL NLTSSVRVLL
```

```
                         841
AthalianaGPA1            DGTKEFA--Q NET------- ---------- ---------- --DS------ ----AKYMLS
CalbicansGPA1            IQARK-L--K IKLDCDQPNN SLIPYKQIIL R--------- --SD------ ----PLKQID
CalbicansGPA2            NAIID-LQPD LIRKPDSPQL EEDHEEDLKV TGLTSENNHV PTANGSVTNQ PPPPPKQRKH
Celeganseg130            RAMDT-L--D IKF------- ---------- ---------- --GN------ ----ESEELQ
Celegansgoa1             RAMSN-L--G VSF------- ---------- ---------- --GS------ ----ADREVD
Celegansgpa1             DGMKQ-L--Q FDF------- ---------- ---------- --ST------ ----RVCNAH
Celegansgpa10            EYVRE-Y--I LEM------- ---------- ---------- --TE------ ----DDDKRY
Celegansgpa11            EAAEF-F--H FPPD------ ---------- ---------- --DS------ ----PSIRRA
Celegansgpa12            DARNK-L--G IAW------- ---------- ---------- --ED------ ----PKRQVE
Celegansgpa13            RAMRM-S--D IQF------- ---------- ---------- --AD------ ----PINMGR
Celegansgpa14            AGSRV-V--G IPL------- ---------- ---------- --DP------ ----IEHITY
Celegansgpa15            KAKDH-F--Y YEL------- ---------- ---------- --ST------ ----PELDAD
Celegansgpa16            HAMEQ-L--K ISF------- ---------- ---------- --TT------ ----LDHESD
Celegansgpa17            RAMDGVL--H LPL------- ---------- ---------- --EN------ ----GQKEAE
Celegansgpa2             KAMPA-A--G LNF------- ---------- ---------- --SD------ ----PMREHD
Celegansgpa3             KGMEA-L--R MTF------- ---------- ---------- --DK------ ----PIREND
Celegansgpa4             KAMQP-L--N ISF------- ---------- ---------- --TD------ ----AAREED
Celegansgpa5             NIILH-S--D ITV------- ---------- ---------- --TQ------ ----EEK---
Celegansgpa6             DACTA-F--G IN-------- ---------- ---------- --HD------ ----ITVQED
Celegansgpa7             RAMFH-L--Q IEY------- ---------- ---------- --GE------ ----PDRVRD
Celegansgpa8             LSVGR-E--N KSY------- ---------- ---------- --KN------ ----PLSFDH
Celegansgpa9             NVIEA-L--E IPI------- ---------- ---------- --SE------ ----DQKQRF
Celegansgsa1             RAMDE-IVPK VSL------- ---------- ---------- --DD------ ----PSTAIS
CglabrataGPA1            VQARK-L--G ISLDCDDPVN HPDLFEHKRV LLS------- --AK------ ----ALNYVD
CglabrataGPA2            SARRK-F--N VDI------- ---------- ---------- --DD------ ----PTLTEE
DmelanogasterCTA         DAREK-L--N IAW------- ---------- ---------- --GS------ ----DGREQD
DmelanogasterGalphaf     GQMGV-L--G IDF------- ---------- ---------- --GS------ ----CTSERS
DmelanogasterGalphai     RAMGR-L--K IEF------- ---------- ---------- --AD------ ----PSRTDI
DmelanogasterGalphao     RAMPT-L--S IQY------- ---------- ---------- --SN------ ----NERESD
DmelanogasterGalphaq     KAMDM-L--K ISY------- ---------- ---------- --GQ------ ----GEHSEL
DmelanogasterGalphas     GAMST-LNPP VAL------- ---------- ---------- --EK------ ----KENEPR
Dreriognal1              RATEH-L--K IPF------- ---------- ---------- --RF------ ----EDNKKN
Dreriognal1a             RATEH-L--K IPF------- ---------- ---------- --RF------ ----EDNKKN
Dreriognal2              DARDK-L--G ISW------- ---------- ---------- --QN------ ----SENEKH
Dreriognal3              DAREK-L--H IPW------- ---------- ---------- --GN------ ----PENQAH
Dreriognal4a             RAMDM-L--K IAY------- ---------- ---------- --AN------ ----SENQAH
Dreriognal2              SAMST-LIPP IPL------- ---------- ---------- --AN------ ----PEDQFR
Dreriognas               TAMSV-LVPP VQL------- ---------- ---------- --AC------ ----PANKFR
Dreriognav1              HGMGV-L--R INL------- ---------- ---------- --AN------ ----PKNKVH
Dreriognaz               RALAT-L--K IDF------- ---------- ---------- --HN------ ----PDRAYD
HsapiensGNA11            RAMET-L--K ILY------- ---------- ---------- --KY------ ----EQNKAN
HsapiensGNA12            DARDK-L--G IPW------- ---------- ---------- --QY------ ----SENEKH
HsapiensGNA13            DAREK-L--H IPW------- ---------- ---------- --GD------ ----NSNQQH
HsapiensGNA14            RAMDT-L--R IQY------- ---------- ---------- --VC------ ----EQNKEN
HsapiensGNA15            EAMER-L--Q IPF------- ---------- ---------- --SR------ ----PESKHH
HsapiensGNAI1            RAMGR-L--K IDF------- ---------- ---------- --GD------ ----SARADD
HsapiensGNAI2            KAMGN-L--Q IDF------- ---------- ---------- --AD------ ----PSRADD
HsapiensGNAI3            RAMGR-L--K IDF------- ---------- ---------- --GE------ ----AARADD
HsapiensGNAL             SAMST-IIPP VPL------- ---------- ---------- --AN------ ----PENQFR
HsapiensGNAO1            RAMDT-L--G IEY------- ---------- ---------- --GD------ ----KERKAD
HsapiensGNAQ             RAMDT-L--K IPY------- ---------- ---------- --KY------ ----EHNKAH
HsapiensGNAS1            AAMSN-LVPP VEL------- ---------- ---------- --AN------ ----PENQFR
HsapiensGNAS2            AAMSN-LVPP VEL------- ---------- ---------- --AN------ ----PENQFR
HsapiensGNAT1            RAMTT-L--N IQY------- ---------- ---------- --GD------ ----SARQDD
HsapiensGNAT2            RAMTT-L--G IDY------- ---------- ---------- --AE------ ----PSCADD
HsapiensGNAT3            KAMTT-L--G IDY------- ---------- ---------- --VN------ ----PRSAED
HsapiensGNAZ             RALAA-L--R IDF------- ---------- ---------- --HN------ ----PDRAYD
Ncrassagna1              EAMES-L--E LPL------- ---------- ---------- --AD------ ----QRVEYH
Ncrassagna2              DAMNE-F--N IKL------- ---------- ---------- --ED------ ----EDNEKN
Ncrassagna3              NAMHQ-F--D IQP------- ---------- ---------- --AD------ ----PSLRPY
ScervisiaeGPA1           IQARK-L--G IQLDCDDPIN NKDLFACKRI LLK------- --AK------ ----ALDYIN
ScervisiaeGPA2           QARTR-F--N VNLE------ ---------- ---------- -PEC------ ----ELTQQD
SparadoxusGPA1           IQARK-L--G IQLDCDDPIK NKDLFACKRI LLK------- --AK------ ----ALDYIN
SparadoxusGPA2           QARTR-F--N VNLE------ ---------- ---------- -AKC------ ----ELTQQD
Umaydisgpa1              DAMER-L--D IPL------- ---------- ---------- --AD------ ----ATNAPR
Umaydisgpa2              DVMEE-W--S IDF------- ---------- ---------- --QD------ ----DSNIDH
Umaydisgpa3              LALRK-F--K MEP------- ---------- ---------- --EM------ ----PENREN
Umaydisgpa4              ETLSLYHDQR LERKSSELSR LESSTSASTS TSASASSPKH V-DT------ ----ESQPND
```

```
                        901
AthalianaGPA1           SESIAIGEKL ---SEIGGR- ---------- ---------- ---------- ----------
CalbicansGPA1           ADVAGGTDFL ---NDFVVK- -YSEENKNKR RLKSTGTTDI WGKDDDSNIN SDAINQALES
CalbicansGPA2           ILEYDMLNEI ---LDYDYP- ---------- ---------- ---------- ----------
Celegansegl30           E------KAA ---VVREVD- ---------- ---------- ---------- ----------
Celegansgoa1            A------KLV ---MDVVAR- ---------- ---------- ---------- ----------
Celegansgpa1            E------KLI ---RETLND- ---------- ---------- ---------- ----------
Celegansgpa10           T------VMC ---EELKMA- ---------- ---------- ---------- ----------
Celegansgpa11           L------NHY ---KSYKVR- ---------- ---------- ---------- ----------
Celegansgpa12           V------EKV ---MRFSVG- -DLLK----- ---------- ---------- ----------
Celegansgpa13           A------QSI -----IADE- ---------- ---------- ---------- ----------
Celegansgpa14           E--------I ---DEIYAP- ---------- ---------- ---------- ----------
Celegansgpa15           A------QHI ---LSLADS- ---------- ---------- ---------- ----------
Celegansgpa16           A------RKL ---LMFSTT- ---------- ---------- ---------- ----------
Celegansgpa17           K------AIV ---MKVQEN- ---------- ---------- ---------- ----------
Celegansgpa2            V------HML ---TLYIKD- ---------- ---------- ---------- ----------
Celegansgpa3            A------KFV ---MESHKM- ---------- ---------- ---------- ----------
Celegansgpa4            A------RMF ISHFLHVNN- ---------- ---------- ---------- ----------
Celegansgpa5            -------VLV ---KLFAYE- ---------- ---------- ---------- ----------
Celegansgpa6            V------NRF ---EDFKRK- ---------- ---------- ---------- ----------
Celegansgpa7            S------QLV ---FATVHA- ---------- ---------- ---------- ----------
Celegansgpa8            I------NEV ---RMFTEN- ---------- ---------- ---------- ----------
Celegansgpa9            T-------QL ---DEYRHR- ---------- ---------- ---------- ----------
Celegansgsa1            R------DYI ---LRITND- ---------- ---------- ---------- ----------
CglabrataGPA1           ASVAGGSGFL ---NDYVIKY SETYALKRRS QSTGKAKAFE DSFQYP---- ----------
CglabrataGPA2           D-----LDKI ---SNYTST- -RKNTDEDSS ---------- ---------- ----------
DmelanogasterCTA        A------YDA ---KLMECN- ---------- ---------- ---------- ----------
DmelanogasterGalphaf    A------DYI ---LSLPGS- ---------- ---------- ---------- ----------
DmelanogasterGalphai    A------RQF ---FTHASA- ---------- ---------- ---------- ----------
DmelanogasterGalphao    A------KMV ---FDVCQR- ---------- ---------- ---------- ----------
DmelanogasterGalphaq    A------DLV ---MSID--- ---------- ---------- ---------- ----------
DmelanogasterGalphas    V------EYI ---QDYASS- ---------- ---------- ---------- ----------
Dreriogna11             A------LLV ---REVD--- ---------- ---------- ---------- ----------
Dreriogna11a            A------LLV ---REVD--- ---------- ---------- ---------- ----------
Dreriogna12             G------MFV ---MSFENK- -AGM------ ---------- ---------- ----------
Dreriogna13             G------ETV ---MAFDTR- -SSLMAKG-- ---------- ---------- ----------
Dreriogna14a            S------ALV ---NDIE--- ---------- ---------- ---------- ----------
Dreriognal2             I------DYI ---KSIAPL- ---------- ---------- ---------- ----------
Dreriognas              I------DYI ---LNLANQ- ---------- ---------- ---------- ----------
Dreriognav1             A------HSV ---LSCGRC- ---------- ---------- ---------- ----------
Dreriognaz              A------VQL ---FALTGP- ---------- ---------- ---------- ----------
HsapiensGNA11           A------LLI ---REVD--- ---------- ---------- ---------- ----------
HsapiensGNA12           G------MFL ---MAFENK- -AGL------ ---------- ---------- ----------
HsapiensGNA13           G------DKM ---MSFDTR- -APMAAQG-- ---------- ---------- ----------
HsapiensGNA14           A------QII ---REVE--- ---------- ---------- ---------- ----------
HsapiensGNA15           A------SLV ---MSQDPY- ---------- ---------- ---------- ----------
HsapiensGNAI1           A------RQL ---FVLAGA- ---------- ---------- ---------- ----------
HsapiensGNAI2           A------RQL ---FALSCT- ---------- ---------- ---------- ----------
HsapiensGNAI3           A------RQL ---FVLAGS- ---------- ---------- ---------- ----------
HsapiensGNAL            S------DYI ---KSIAPI- ---------- ---------- ---------- ----------
HsapiensGNAO1           A------KMV ---CDVVSR- ---------- ---------- ---------- ----------
HsapiensGNAQ            A------QLV ---REVD--- ---------- ---------- ---------- ----------
HsapiensGNAS1           V------DYI ---LSVMNV- ---------- ---------- ---------- ----------
HsapiensGNAS2           V------DYI ---LSVMNV- ---------- ---------- ---------- ----------
HsapiensGNAT1           A------RKL ---MHMADT- ---------- ---------- ---------- ----------
HsapiensGNAT2           G------RQL ---NNLADS- ---------- ---------- ---------- ----------
HsapiensGNAT3           Q------RQL ---YAMANT- ---------- ---------- ---------- ----------
HsapiensGNAZ            A------VQL ---FALTGP- ---------- ---------- ---------- ----------
Ncrassagna1             V------QTI ---FMQPAQ- ---------- ---------- ---------- ----------
Ncrassagna2             M-----VQMM ---VDYEMR- ---------- ---------- ---------- ----------
Ncrassagna3             V------EFL ---QDYNME- ---------- ---------- ---------- ----------
ScervisiaeGPA1          ASVAGGSDFL ---NDYVLKY SERYETRRRV QSTGRAKAAF DEDGNISNVK SDTDRDAETV
ScervisiaeGPA2          L------SRT ---MSYEMP- ---------- ---------- ---------- ----------
SparadoxusGPA1          ASVAGGSEFL ---NDYVLKY SERYETRRRV QSTGRAKAAF DEDGNIPNTR SDTNKDAEAV
SparadoxusGPA2          L------SKA ---MSYEMP- ---------- ---------- ---------- ----------
Umaydisgpa1             A------EII ---LGLSPS- ---------- ---------- ---------- ----------
Umaydisgpa2             L------LLF ---VSYPDI- ---------- ---------- ---------- ----------
Umaydisgpa3             V------DAI ---LQYRVD- ---------- ---------- ---------- ----------
Umaydisgpa4             ATRLELAKQL GTTKKINTSS LPWLTHIPSV VQLERALRTE LGAFGEEAVL SASSDEAAIT
```

```
                       961
AthalianaGPA1          ---------- ---------- ---------- ---------- ---------- LDY-------
CalbicansGPA1          SL-------- ---------- ---------- ---------- ---------- NKDS------
CalbicansGPA2          ---------- ---------- ---------- ---------- ---------- LNLN------
Celegansegl30          ---------- ---------- ---------- ---------- ---------- FESV------
Celegansgoa1           ---------- ---------- ---------- ---------- ---------- MEDT------
Celegansgpa1           ---------- ---------- ---------- ---------- ---------- KAEY------
Celegansgpa10          ---------- ---------- ---------- ---------- ---------- VIHD------
Celegansgpa11          ---------- ---------- ---------- ---------- ---------- YSTSE-----
Celegansgpa12          ---------- ---------- ---------- ---------- ---------- GIDF------
Celegansgpa13          ---------- ---------- ---------- ---------- ---------- HGHY------
Celegansgpa14          ---------- ---------- ---------- ---------- ---------- MSDA------
Celegansgpa15          ---------- ---------- ---------- ---------- ---------- SKDA------
Celegansgpa16          ---------- ---------- ---------- ---------- ---------- GEE-------
Celegansgpa17          ---------- ---------- ---------- ---------- ---------- GEEG------
Celegansgpa2           ---------- ---------- ---------- ---------- ---------- MQH-------
Celegansgpa3           ---------- ---------- ---------- ---------- ---------- LQEA------
Celegansgpa4           ---------- ---------- ---------- ---------- ---------- AELS------
Celegansgpa5           ---------- ---------- ---------- ---------- ---------- SGKI------
Celegansgpa6           ---------- ---------- ---------- ---------- ---------- LRDPEG----
Celegansgpa7           ---------- ---------- ---------- ---------- ---------- NK--------
Celegansgpa8           ---------- ---------- ---------- ---------- ---------- FKKADDSE--
Celegansgpa9           ---------- ---------- ---------- ---------- ---------- ----------
Celegansgsa1           ---------- ---------- ---------- ---------- ---------- PE--------
CglabrataGPA1          ---------- ---------- ---------- ---------- ---------- YDDNEKLNLE
CglabrataGPA2          ---------- ---------- ---------- ---------- ---------- EGENSITVKP
DmelanogasterCTA       ---------- ---------- ---------- ---------- ---------- SLDV------
DmelanogasterGalphaf   ---------- ---------- ---------- ---------- ---------- AP--------
DmelanogasterGalphai   ---------- ---------- ---------- ---------- ---------- ADE-------
DmelanogasterGalphao   ---------- ---------- ---------- ---------- ---------- MHDT------
DmelanogasterGalphaq   ---------- ---------- ---------- ---------- ---------- YETV------
DmelanogasterGalphas   ---------- ---------- ---------- ---------- ---------- PD--------
Dreriogna11            ---------- ---------- ---------- ---------- ---------- VEKV------
Dreriogna11a           ---------- ---------- ---------- ---------- ---------- VEKV------
Dreriogna12            ---------- ---------- ---------- ---------- ---------- AVEP------
Dreriogna13            ---------- ---------- ---------- ---------- ---------- MMET------
Dreriogna14a           ---------- ---------- ---------- ---------- ---------- VDKI------
Dreriogna12            ---------- ---------- ---------- ---------- ---------- SD--------
Dreriognas             ---------- ---------- ---------- ---------- ---------- KD--------
Dreriognav1            ---------- ---------- ---------- ---------- ---------- FDED------
Dreriognaz             ---------- ---------- ---------- ---------- ---------- AESK------
HsapiensGNA11          ---------- ---------- ---------- ---------- ---------- VEKV------
HsapiensGNA12          ---------- ---------- ---------- ---------- ---------- PVEP------
HsapiensGNA13          ---------- ---------- ---------- ---------- ---------- MVET------
HsapiensGNA14          ---------- ---------- ---------- ---------- ---------- VDKV------
HsapiensGNA15          ---------- ---------- ---------- ---------- ---------- KV--------
HsapiensGNAI1          ---------- ---------- ---------- ---------- ---------- AEE-------
HsapiensGNAI2          ---------- ---------- ---------- ---------- ---------- AEEQ------
HsapiensGNAI3          ---------- ---------- ---------- ---------- ---------- AEE-------
HsapiensGNAL           ---------- ---------- ---------- ---------- ---------- TD--------
HsapiensGNAO1          ---------- ---------- ---------- ---------- ---------- MEDT------
HsapiensGNAQ           ---------- ---------- ---------- ---------- ---------- VEKV------
HsapiensGNAS1          ---------- ---------- ---------- ---------- ---------- PD--------
HsapiensGNAS2          ---------- ---------- ---------- ---------- ---------- PD--------
HsapiensGNAT1          ---------- ---------- ---------- ---------- ---------- IEE-------
HsapiensGNAT2          ---------- ---------- ---------- ---------- ---------- IEE-------
HsapiensGNAT3          ---------- ---------- ---------- ---------- ---------- LED-------
HsapiensGNAZ           ---------- ---------- ---------- ---------- ---------- AESK------
Ncrassagna1            ---------- ---------- ---------- ---------- ---------- IEG-------
Ncrassagna2            ---------- ---------- ---------- ---------- ---------- GD--------
Ncrassagna3            ---------- ---------- ---------- ---------- ---------- GCPPG-----
ScervisiaeGPA1         TQNEDADRNN SSRINLQDIC KDLN------ ---------- ---------- QEGDDQMFVR
ScervisiaeGPA2         ---------- ---------- ---------- ---------- ---------- NNYT------
SparadoxusGPA1         TQNEDDDRNN RSRLNLQDIC KDLN------ ---------- ---------- QEGDDQMFVR
SparadoxusGPA2         ---------- ---------- ---------- ---------- ---------- NNYT------
Umaydisgpa1            ---------- ---------- ---------- ---------- ---------- IES-------
Umaydisgpa2            ---------- ---------- ---------- ---------- ---------- SED-------
Umaydisgpa3            ---------- ---------- ---------- ---------- ---------- ADPG------
Umaydisgpa4            NQKVRTRLDA QGSSSIRGEK SPLVLRPGWQ ERLFTYARRS LSLTQNGRAA AARRETDGGD
```

```
                           1021
AthalianaGPA1              ---------- --PRLT--KD ---------- IAEGIETLWK -DPAIQETC- -ARGNELQVP
CalbicansGPA1              ---------- --EQFTR-LS ---------- IAEAIHKLWK LDSGIKKCF- -DRSNEFQLE
CalbicansGPA2              ---------- --QQFD--PD ---------- IAIKIKKVYE -TPEVKEFML -QRQGDFYLI
Celeganseg130              ---------- --TSFE--EP ---------- YVSYIKELWE -DSGIQECY- -DRRREYQLT
Celegansgoa1               ---------- --EPFS--EE ---------- LLSSMKRLWG -DAGVQDCF- -SRSNEYQLN
Celegansgpa1               ---------- --GPFS--DA ---------- MFNALTELWA -DKGVQCAY- -DKREFFYLH
Celegansgpa10              ---------- --GIMR--PE ---------- LADTLKEFWG -NSAIQDAY- -SKRDTFHLT
Celegansgpa11              ---------- --VELN--RE ---------- LADSLSKLYN -AEFIKSVL- -NRKNELKLL
Celegansgpa12              ---------- --TTF---VE ---------- VAPIISDFWN -DAAIRKTY- -EQRNLFQIS
Celegansgpa13              ---------- --GLFS--KD ---------- LAEKIKHIWN -DKSMQKLY- -ARRSQFNLN
Celegansgpa14              ---------- --FSVRTISE ---------- LLEPLTEFWN -SKQIQEIY- -KRRCEFELL
Celegansgpa15              ---------- --MPFI--PL ---------- TFNAIKRLWH -DPVVQKTF- -ERRAEFQMM
Celegansgpa16              ---------- --DELP--EE ---------- LVVLMKSVWS -DSGIQKAL- -ERSREYQLN
Celegansgpa17              ---------- --EALT--EE ---------- VSKAIQSLWA -DPGVKKAF- -EMRSEYQLP
Celegansgpa2               ---------- --KNFQ--QD ---------- AADHVEKLWK -DPVVKRLY- -AERKELNIR
Celegansgpa3               ---------- --KVFP--EE ---------- LANAIQALWN -DKAVQQVI- -AKGNEFQMP
Celegansgpa4               ---------- --EAFS--LE ---------- LSDLMKQLWM -DEGVKKCV- -KRAHEYQLN
Celegansgpa5               ---------- --ELMQE-VD ---------- ELNVINAVSG -YECIKQFF- -ERFAFHPMV
Celegansgpa6               ---------- --LVIP--VV ---------- IGRCMDRVWH -SPSLQLCYD -TRRFRFALL
Celegansgpa7               ---------- --EELT--EE ---------- LAAAMQRLWH -DPGVRECY- -RRSNEYQID
Celegansgpa8               ---------- --KLLG--PE ---------- VINAIQKYIK -DETIAMML- -RDKTVYNID
Celegansgpa9               ---------- --EVFP--PK ---------- IIEAMKELFE -SGLYELCR- -LRQRILPLP
Celegansgsa1               ---------- --DNYP--SE ---------- FYDHILTCWK -DKGVMACY- -ERSSEYQLI
CglabrataGPA1              EISKGLHEND DGSIFINSKQ IKSPTVNNKT IAAAISNLWL KDRGIKQCF- -ACANEFQLE
CglabrataGPA2              SEQANTTSIV SKDEFP--KE ---------- FYPILSKLWN -MKSTQELIM SEKSSQFYMM
DmelanogasterCTA           ---------- --PKF---ME ---------- YAPPISRLWQ -DRGIRRAF- -ERRREFQIS
DmelanogasterGalphaf       ---------- --EYMN--EE ---------- YCDHVTTLWN -DVGIRACY- -DRSNEFPLL
DmelanogasterGalphai       ---------- --GILL--PE ---------- IVLLMKKLWA -DGGVQQSF- -ARSREYQLN
DmelanogasterGalphao       ---------- --EPFS--EE ---------- LLAAMKRLWQ -DAGVQECF- -SRSNEYQLN
DmelanogasterGalphaq       ---------- --TTFE--DP ---------- YLNAIKTLWD -DAGIQECY- -DRRREYQLT
DmelanogasterGalphas       ---------- --FNYP--PE ---------- FYEHTEELWK -DKGVLQTY- -ERSNEYQLI
Dreriogna11                ---------- --CSFE--QP ---------- YVAAINKLWM -DPGIQEAY- -DRRREYQLS
Dreriogna11a               ---------- --CSFE--QP ---------- YVAAINKLWM -DPGIQEAY- -DRRREYQLS
Dreriogna12                ---------- --CTF---QL ---------- YVPALQALWN -DSGIQEAY- -GRRSEFQLS
Dreriogna13                ---------- --KVF---LN ---------- FLPAIRALWQ -DSGIQNAY- -DRRREFQLG
Dreriogna14a               ---------- --MSLD--ET ---------- QVKALSSLWS -DSGIQECY- -DRRREYQLT
Dreriogna12                ---------- --FDYT--QE ---------- FFDHAKKLWE -DEGVKACY- -ERSNEYQLI
Dreriognas                 ---------- --FEFT--TE ---------- FYEHTKTLWQ -DEGVKACF- -ERSNEYQLI
Dreriognav1                ---------- --QMLF--PF ---------- IAHALCCLWA -DPGVRSSA- -ARGYEYELN
Dreriognaz                 ---------- --GEIT--PE ---------- LLGVMKRLWA -DPGVQECF- -CRSNEYHLE
HsapiensGNA11              ---------- --TTFE--HQ ---------- YVSAIKTLWE -DPGIQECY- -DRRREYQLS
HsapiensGNA12              ---------- --ATF---QL ---------- YVPALSALWR -DSGIREAF- -SRRSEFQLG
HsapiensGNA13              ---------- --RVF---LQ ---------- YLPAIRALWA -DSGIQNAY- -DRRREFQLG
HsapiensGNA14              ---------- --SMLS--RE ---------- QVEAIKQLWQ -DPGIQECY- -DRRREYQLS
HsapiensGNA15              ---------- --TTFE--KR ---------- YAAAMQWLWR -DAGIRACY- -ERRREFHLL
HsapiensGNAI1              ---------- --GFMT--AE ---------- LAGVIKRLWK -DSGVQACF- -NRSREYQLN
HsapiensGNAI2              ---------- --GVLP--DD ---------- LSGVIRRLWA -DHGVQACF- -GRSREYQLN
HsapiensGNAI3              ---------- --GVMT--PE ---------- LAGVIKRLWR -DGGVQACF- -SRSREYQLN
HsapiensGNAL               ---------- --FEYS--QE ---------- FFDHVKKLWD -DEGVKACF- -ERSNEYQLI
HsapiensGNAO1              ---------- --EPFS--AE ---------- LLSAMMRLWG -DSGIQECF- -NRSREYQLN
HsapiensGNAQ               ---------- --SAFE--NP ---------- YVDAIKSLWN -DPGIQECY- -DRRREYQLS
HsapiensGNAS1              ---------- --FDFP--PE ---------- FYEHAKALWE -DEGVRACY- -ERSNEYQLI
HsapiensGNAS2              ---------- --FDFP--PE ---------- FYEHAKALWE -DEGVRACY- -ERSNEYQLI
HsapiensGNAT1              ---------- --GTMP--KE ---------- MSDIIQRLWK -DSGIQACF- -ERASEYQLN
HsapiensGNAT2              ---------- --GTMP--PE ---------- LVEVIRRLWK -DGGVQACF- -ERAAEYQLN
HsapiensGNAT3              ---------- --GGMT--PQ ---------- LAEVIKRLWR -DPGIQACF- -ERASEYQLN
HsapiensGNAZ               ---------- --GEIT--PE ---------- LLGVMRRLWA -DPGAQACF- -SRSSEYHLE
Ncrassagna1                ---------- --DVLP--PE ---------- VGNAIEALWR -DAGVQSCF- -KRSREYQLN
Ncrassagna2                ---------- --EPLP--LE ---------- YFEPAKKLWQ -DSGVRQAI- -EKGNEFALH
Ncrassagna3                ---------- --QSID--PK ---------- VGTAIQALWN -DPAKEQLM- -ERQTEFYLM
ScervisiaeGPA1             KTSREIQGQN RRNLIH--ED ---------- IAKAIKQLWN NDKGIKQCF- -ARSNEFQLE
ScervisiaeGPA2             ---------- --GQFP--ED ---------- IAGVISTLWA -LPSTQDLVN GPNASKFYLM
SparadoxusGPA1             KTSREIQGQN RQNLIH--ED ---------- IAKAIKQLWN NDKGIKQCF- -ARSNEFQLE
SparadoxusGPA2             ---------- --GQFP--EN ---------- IAGIISTLWA -LPSTQDLVN GPNASKFYLM
Umaydisgpa1                ---------- --SVLP--RQ ---------- VADAIHALWG -DAGVQACF- -GRSREYQLN
Umaydisgpa2                ---------- --EPFP--TN ---------- YLVALKDLWL -DQGVQSVY- -RRGNEAAVP
Umaydisgpa3                ---------- --ATLD--HA ---------- MARKVDSLWK -DPIVPAIM- -ERSSEFYLM
Umaydisgpa4                SQSESEKDNS ETLKLL--RA ---------- IRPEVLALWN DDAGCRA--- -LRKRGLFLD
```

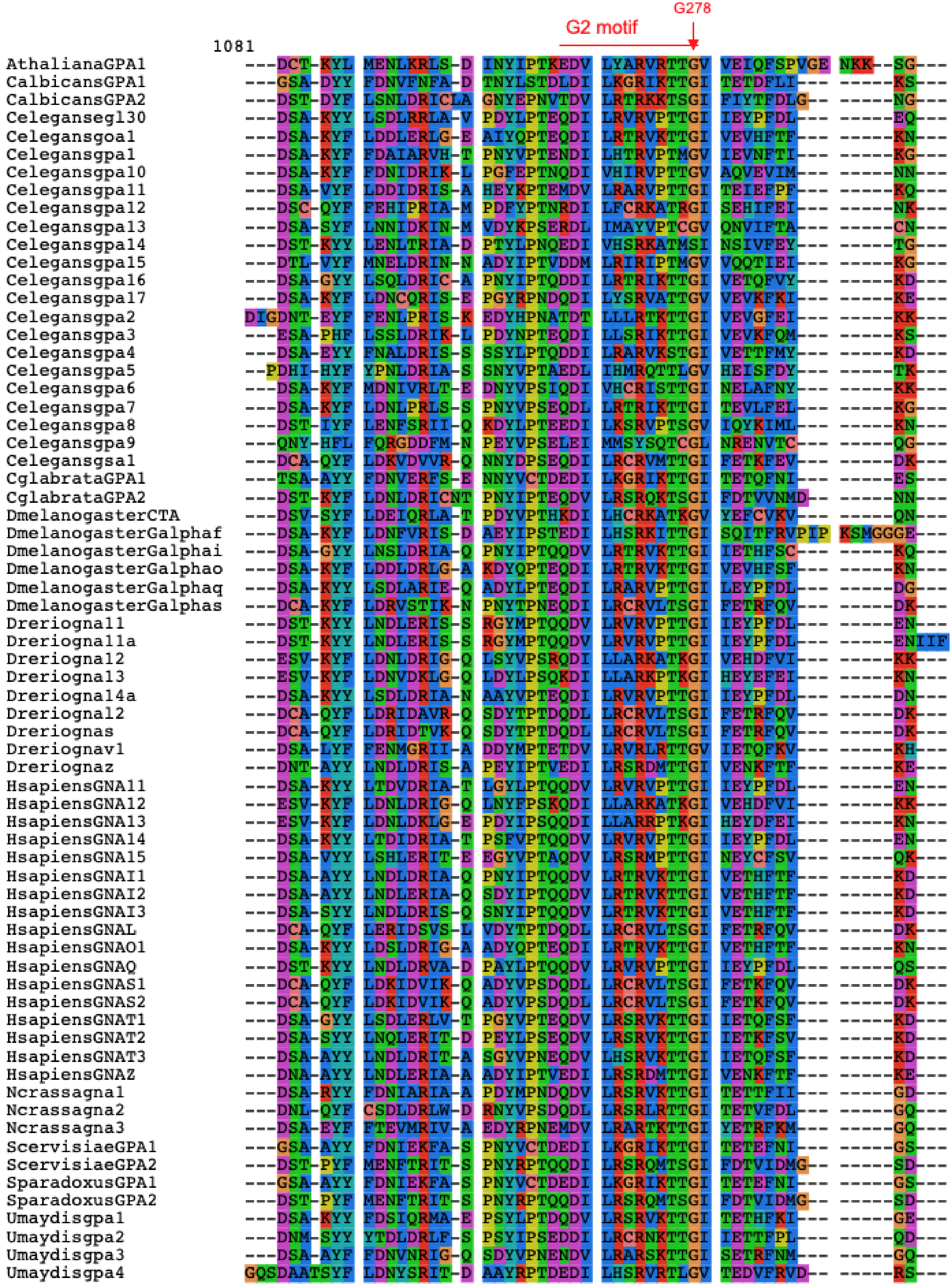
G278
G2 motif
1081
AthalianaGPA1 ---DCT-KYL MENLKRLS-D INYIPTKEDV LYARVRTTGV VEIQFSPVGE NKK--SG---
CalbicansGPA1 ---GSA-DYY FDNVFNFA-D TNYLSTDLDI LKGRIKTTGI TETDFLI--- -----KS---
CalbicansGPA2 ---DST-DYF LSNLDRICLA GNYEPNVTDV LRTRKKTSGI FIYTFDLG-- -----NG---
Celeganesegl30 ---DSA-KYY LSDLRRLA-V PDYLPTEQDI LRVRVPTTGI IEYPFDL--- -----EQ---
Celegansgoa1 ---DSA-KYF LDDLERLG-E AIYQPTEQDI LRTRVKTTGI VEVHFTF--- -----KN---
Celegansgpa1 ---DSA-KYF FDAIARVH-T PNYVPTENDI LHTRVPTMGV IEVNFTI--- -----KG---
Celegansgpa10 ---DSA-KYF FDNIDRIK-L PGFEPTNQDI VHIRVPTTGV AQVEVIM--- -----NN---
Celegansgpa11 ---DSA-VYF LDDIDRIS-A HEYKPTEMDV LRARVPTTGI TEIEFPF--- -----KQ---
Celegansgpa12 ---DSC-QYF FEHIPRIA-M PDFYPTNRDI LFCRKATRGI SEHIFEI--- -----NK---
Celegansgpa13 ---DSA-SYF LNNIDKIN-M VDYKPSERDL IMAYVPTCGV QNVIFTA--- -----CN---
Celegansgpa14 ---DST-KYY LENLTRIA-D PTYLPNQEDI VHSRKATMSI NSIVFEY--- -----TG---
Celegansgpa15 ---DTL-VYF MNELDRIN-N ADYIPTVDDM LRIRIPTMGV VQQTIEI--- -----KG---
Celegansgpa16 ---DSA-GYY LSQLDRIC-A PNYIPTQDDI LRTRIKTTGI VETQFVY--- -----KD---
Celegansgpa17 ---DSA-KYF LDNCQRIS-E PGYRPNDQDI LYSRVATTGV VEVKFKI--- -----KE---
Celegansgpa2 DIGDNT-EYF FENLPRIS-K EDYHPNATDT LLLRTKTTGI VEVGFEI--- -----KK---
Celegansgpa3 ---ESA-PHF LSSLDRIK-L PDYNPTEQDI LLSRIKTTGI VEVKFQM--- -----KS---
Celegansgpa4 ---DSA-EYY FNALDRIS-S SSYLPTQDDI LRARVKSTGI VETTFMY--- -----KD---
Celegansgpa5 --PDHI-HYF YPNLDRIA-S SNYVPTAEDL IHMRQTTLGV HEISFDY--- -----TK---
Celegansgpa6 ---DSA-KYF MDNIVRLT-E DNYVPSIQDI VHCRISTTGI NELAFNY--- -----KK---
Celegansgpa7 ---DSA-KYF LDNLPRLS-S PNYVPSEQDL LRTRIKTTGI TEVLFEL--- -----KG---
Celegansgpa8 ---DST-IYF LENFSRII-Q KDYLPTEEDI LKSRVPTSGV IQYKIML--- -----KN---
Celegansgpa9 ---QNY-HFL FQRGDDFM-N PEYVPSELEI MMSYSQTCGL NRENVTC--- -----QG---
Celegansgsa1 ---DCA-QYF LDKVDVVR-Q NNYDPSEQDI LRCRVMTTGI FETKFEV--- -----DK---
CglabrataGPA1 ---TSA-AYY FDNVERFS-E NNYVCTDEDI LKGRIKTTGI TETQFNI--- -----ES---
CglabrataGPA2 ---DST-KYF LDNLDRICNT PNYIPTEQDV LRSRQKTSGI FDTVVNMD-- -----NN---
DmelanogasterCTA ---DSV-SYF LDEIQRLA-T PDYVPTHKDI LHCRKATKGV YEFCVKV--- -----QN---
DmelanogasterGalphaf ---DSA-KYF LDNFVRIS-D AEYIPSTEDI LHSRKITTGI SQITFRVPIP KSMGGGE---
DmelanogasterGalphai ---DSA-GYY LNSLDRIA-Q PNYIPTQQDV LRTRVKTTGI IETHFSC--- -----KQ---
DmelanogasterGalphao ---DSA-KYF LDDLDRLG-A KDYQPTEQDI LRTRVKTTGI VEVHFSF--- -----KN---
DmelanogasterGalphaq ---DSA-KYY LSDLARIE-Q ADYLPTEQDI LRARVPTTGI LEYPFDL--- -----DG---
DmelanogasterGalphas ---DCA-KYF LDRVSTIK-N PNYTPNEQDI LRCRVLTSGI FETRFQV--- -----DK---
Dreriognal1 ---DST-KYY LNDLERIS-S RGYMPTQQDV LRVRVPTTGI IEYPFDL--- -----EN---
Dreriognal1a ---DST-KYY LNDLERIS-S RGYMPTQQDV LRVRVPTTGI IEYPFDL--- -----ENIIF
Dreriognal2 ---ESV-KYF LDNLDRIG-Q LSYVPSRQDI LLARKATKGI VEHDFVI--- -----KK---
Dreriognal3 ---ESV-KYF LDNVDKLG-Q LDYLPSQKDI LLARKPTKGI HEYEFEI--- -----KN---
Dreriognal4a ---DSA-KYY LSDLDRIA-N AAYVPTEQDI LRVRVPTTGI IEYPFDL--- -----DN---
Dreriognal2 ---DCA-QYF LDRIDAVR-Q SDYTPTDQDL LRCRVLTSGI FETRFQV--- -----DK---
Dreriognas ---DCA-QYF LDRIDTVK-Q SDYTPTDQDL LRCRVLTSGI FETRFQV--- -----DK---
Dreriognav1 ---DSA-LYF FENMGRII-A DDYMPTETDV LRVRLRTTGV IETQFKV--- -----KH---
Dreriognaz ---DNT-AYY LNDLDRIS-A PEYIPTVEDI LRSRDMTTGI VENKFTF--- -----KE---
HsapiensGNA11 ---DSA-KYY LTDVDRIA-T LGYLPTQQDV LRVRVPTTGI IEYPFDL--- -----EN---
HsapiensGNA12 ---ESV-KYF LDNLDRIG-Q LNYFPSKQDI LLARKATKGI VEHDFVI--- -----KK---
HsapiensGNA13 ---ESV-KYF LDNLDKLG-E PDYIPSQQDI LLARRPTKGI HEYDFEI--- -----KN---
HsapiensGNA14 ---DSA-KYY LTDIDRIA-T PSFVPTQQDV LRVRVPTTGI IEYPFDL--- -----EN---
HsapiensGNA15 ---DSA-VYY LSHLERIT-E EGYVPTAQDV LRSRMPTTGI NEYCFSV--- -----QK---
HsapiensGNAI1 ---DSA-AYY LNDLDRIA-Q PNYIPTQQDV LRTRVKTTGI VETHFTF--- -----KD---
HsapiensGNAI2 ---DSA-AYY LNDLERIA-Q SDYIPTQQDV LRTRVKTTGI VETHFTF--- -----KD---
HsapiensGNAI3 ---DSA-SYY LNDLDRIS-Q SNYIPTQQDV LRTRVKTTGI VETHFTF--- -----KD---
HsapiensGNAL ---DCA-QYF LERIDSVS-L VDYTPTDQDL LRCRVLTSGI FETRFQV--- -----DK---
HsapiensGNAO1 ---DSA-KYY LDSLDRIG-A ADYQPTEQDI LRTRVKTTGI VETHFTF--- -----KN---
HsapiensGNAQ ---DST-KYY LNDLDRVA-D PAYLPTQQDV LRVRVPTTGI IEYPFDL--- -----QS---
HsapiensGNAS1 ---DCA-QYF LDKIDVIK-Q ADYVPSDQDL LRCRVLTSGI FETKFQV--- -----DK---
HsapiensGNAS2 ---DCA-QYF LDKIDVIK-Q ADYVPSDQDL LRCRVLTSGI FETKFQV--- -----DK---
HsapiensGNAT1 ---DSA-GYY LSDLERLV-T PGYVPTEQDV LRSRVKTTGI IETQFSF--- -----KD---
HsapiensGNAT2 ---DSA-SYY LNQLERIT-D PEYLPSEQDV LRSRVKTTGI IETKFSV--- -----KD---
HsapiensGNAT3 ---DSA-AYY LNDLDRIT-A SGYVPNEQDV LHSRVKTTGI IETQFSF--- -----KD---
HsapiensGNAZ ---DNA-AYY LNDLERIA-A ADYIPTVEDI LRSRDMTTGI VENKFTF--- -----KE---
Ncrassagna1 ---DSA-RYY FDNIARIA-A PDYMPNDQDV LRSRVKTTGI TETTFII--- -----GD---
Ncrassagna2 ---DNL-QYF CSDLDRLW-D RNYVPSDQDL LRSRLRTTGI TETVFDL--- -----GQ---
Ncrassagna3 ---DSA-EYF FTEVMRIV-A EDYRPNEMDV LRARTKTTGI YETRFKM--- -----GQ---
ScervisiaeGPA1 ---GSA-AYY FDNIEKFA-S PNYVCTDEDI LKGRIKTTGI TETEFNI--- -----GS---
ScervisiaeGPA2 ---DST-PYF MENFTRIT-S PNYRPTQQDI LRSRQMTSGI FDTVIDMG-- -----SD---
SparadoxusGPA1 ---GSA-AYY FDNIEKFA-S PNYVCTDEDI LKGRIKTTGI TETEFNI--- -----GS---
SparadoxusGPA2 ---DST-PYF MENFTRIT-S PNYRPTQQDI LRSRQMTSGI FDTVIDMG-- -----SD---
Umaydisgpa1 ---DSA-KYY FDSIQRMA-E PSYLPTDQDV LRSRVKTTGI TETHFKI--- -----GE---
Umaydisgpa2 ---DNM-SYY YTDLDRLF-S PSYIPSEDDI LRCRNKTTGI IETTFPL--- -----QD---
Umaydisgpa3 ---DSA-AYF FDNVNRIG-Q SDYVPNENDV LRARSKTTGI SETRFNM--- -----GQ---
Umaydisgpa4 GQSDAATSYF LDNYSRIT-D AAYRPTDEDI LHSRVRTLGV TEDVFRVD-- -----RS---

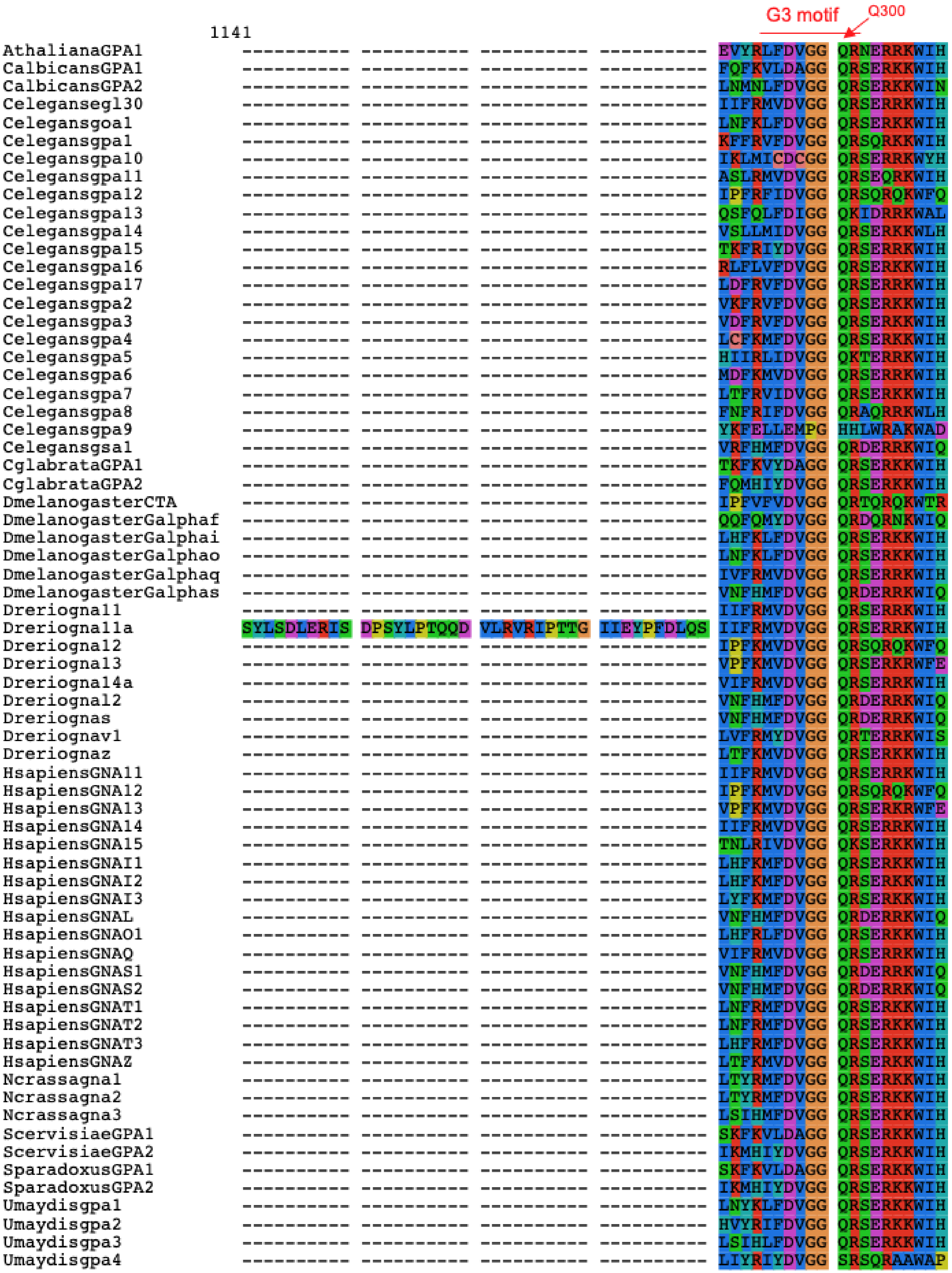

G3 motif
Q300
1141
AthalianaGPA1 ---------- ---------- ---------- ---------- EVYRLFDVGG QRNERRKWIH
CalbicansGPA1 ---------- ---------- ---------- ---------- FQFKVLDAGG QRSERKKWIH
CalbicansGPA2 ---------- ---------- ---------- ---------- LNMNLFDVGG QRSERKKWIN
Celegansegl30 ---------- ---------- ---------- ---------- IIFRMVDVGG QRSERRKWIH
Celegansgoa1 ---------- ---------- ---------- ---------- LNFKLFDVGG QRSERKKWIH
Celegansgpa1 ---------- ---------- ---------- ---------- KFFRVFDVGG QRSQRKKWIH
Celegansgpa10 ---------- ---------- ---------- ---------- IKLMICDCGG QRSERRKWYH
Celegansgpa11 ---------- ---------- ---------- ---------- ASLRMVDVGG QRSEQRKWIH
Celegansgpa12 ---------- ---------- ---------- ---------- IPFRFIDVGG QRSQRQKWFQ
Celegansgpa13 ---------- ---------- ---------- ---------- QSFQLFDIGG QKIDRRKWAL
Celegansgpa14 ---------- ---------- ---------- ---------- VSLLMIDVGG QRSERKKWLH
Celegansgpa15 ---------- ---------- ---------- ---------- TKFRIYDVGG QRSERRKWIH
Celegansgpa16 ---------- ---------- ---------- ---------- RLFLVFDVGG QRSERKKWIH
Celegansgpa17 ---------- ---------- ---------- ---------- LDFRVFDVGG QRSERRKWIH
Celegansgpa2 ---------- ---------- ---------- ---------- VKFRVFDVGG QRSERKKWIH
Celegansgpa3 ---------- ---------- ---------- ---------- VDFRVFDVGG QRSERKKWIH
Celegansgpa4 ---------- ---------- ---------- ---------- LCFKMFDVGG QRSERKKWIH
Celegansgpa5 ---------- ---------- ---------- ---------- HIIRLIDVGG QKTERRKWIH
Celegansgpa6 ---------- ---------- ---------- ---------- MDFKMVDVGG QRSERRKWIH
Celegansgpa7 ---------- ---------- ---------- ---------- LTFRVIDVGG QRSERKKWIH
Celegansgpa8 ---------- ---------- ---------- ---------- FNFRIFDVGG QRAQRRKWLH
Celegansgpa9 ---------- ---------- ---------- ---------- YKFELLEMPG HHLWRAKWAD
Celegansgsa1 ---------- ---------- ---------- ---------- VRFHMFDVGG QRDERRKWIQ
CglabrataGPA1 ---------- ---------- ---------- ---------- TKFKVYDAGG QRSERKKWIH
CglabrataGPA2 ---------- ---------- ---------- ---------- FQMHIYDVGG QRSERKKWIH
DmelanogasterCTA ---------- ---------- ---------- ---------- IPFVFVDVGG QRTQRQKWTR
DmelanogasterGalphaf ---------- ---------- ---------- ---------- QQFQMYDVGG QRDQRNKWIQ
DmelanogasterGalphai ---------- ---------- ---------- ---------- LHFKLFDVGG QRSERKKWIH
DmelanogasterGalphao ---------- ---------- ---------- ---------- LNFKLFDVGG QRSERKKWIH
DmelanogasterGalphaq ---------- ---------- ---------- ---------- IVFRMVDVGG QRSERRKWIH
DmelanogasterGalphas ---------- ---------- ---------- ---------- VNFHMFDVGG QRDERRKWIQ
Dreriogna11 ---------- ---------- ---------- ---------- IIFRMVDVGG QRSERRKWIH
Dreriogna11a SYLSDLERIS DPSYLPTQQD VLRVRIPTTG IIEYPFDLQS IIFRMVDVGG QRSERRKWIH
Dreriogna12 ---------- ---------- ---------- ---------- IPFKMVDVGG QRSQRQKWFQ
Dreriogna13 ---------- ---------- ---------- ---------- VPFKMVDVGG QRSERKRWFE
Dreriogna14a ---------- ---------- ---------- ---------- VIFRMVDVGG QRSERRKWIH
Dreriognal2 ---------- ---------- ---------- ---------- VNFHMFDVGG QRDERRKWIQ
Dreriognas ---------- ---------- ---------- ---------- VNFHMFDVGG QRDERRKWIQ
Dreriognav1 ---------- ---------- ---------- ---------- LVFRMYDVGG QRTERRKWIS
Dreriognaz ---------- ---------- ---------- ---------- LTFKMVDVGG QRSERKKWIH
HsapiensGNA11 ---------- ---------- ---------- ---------- IIFRMVDVGG QRSERRKWIH
HsapiensGNA12 ---------- ---------- ---------- ---------- IPFKMVDVGG QRSQRQKWFQ
HsapiensGNA13 ---------- ---------- ---------- ---------- VPFKMVDVGG QRSERKRWFE
HsapiensGNA14 ---------- ---------- ---------- ---------- IIFRMVDVGG QRSERRKWIH
HsapiensGNA15 ---------- ---------- ---------- ---------- TNLRIVDVGG QKSERKKWIH
HsapiensGNAI1 ---------- ---------- ---------- ---------- LHFKMFDVGG QRSERKKWIH
HsapiensGNAI2 ---------- ---------- ---------- ---------- LHFKMFDVGG QRSERKKWIH
HsapiensGNAI3 ---------- ---------- ---------- ---------- LYFKMFDVGG QRSERKKWIH
HsapiensGNAL ---------- ---------- ---------- ---------- VNFHMFDVGG QRDERRKWIQ
HsapiensGNAO1 ---------- ---------- ---------- ---------- LHFRLFDVGG QRSERKKWIH
HsapiensGNAQ ---------- ---------- ---------- ---------- VIFRMVDVGG QRSERRKWIH
HsapiensGNAS1 ---------- ---------- ---------- ---------- VNFHMFDVGG QRDERRKWIQ
HsapiensGNAS2 ---------- ---------- ---------- ---------- VNFHMFDVGG QRDERRKWIQ
HsapiensGNAT1 ---------- ---------- ---------- ---------- LNFRMFDVGG QRSERKKWIH
HsapiensGNAT2 ---------- ---------- ---------- ---------- LNFRMFDVGG QRSERKKWIH
HsapiensGNAT3 ---------- ---------- ---------- ---------- LHFRMFDVGG QRSERKKWIH
HsapiensGNAZ ---------- ---------- ---------- ---------- LTFKMVDVGG QRSERKKWIH
Ncrassagna1 ---------- ---------- ---------- ---------- LTYRMFDVGG QRSERKKWIH
Ncrassagna2 ---------- ---------- ---------- ---------- LTYRMFDVGG QRSERKKWIH
Ncrassagna3 ---------- ---------- ---------- ---------- LSIHMFDVGG QRSERKKWIH
ScervisiaeGPA1 ---------- ---------- ---------- ---------- SKFKVLDAGG QRSERKKWIH
ScervisiaeGPA2 ---------- ---------- ---------- ---------- IKMHIYDVGG QRSERKKWIH
SparadoxusGPA1 ---------- ---------- ---------- ---------- SKFKVLDAGG QRSERKKWIH
SparadoxusGPA2 ---------- ---------- ---------- ---------- IKMHIYDVGG QRSERKKWIH
Umaydisgpa1 ---------- ---------- ---------- ---------- LNYKLFDVGG QRSERKKWIH
Umaydisgpa2 ---------- ---------- ---------- ---------- HVYRIFDVGG QRSERKKWIH
Umaydisgpa3 ---------- ---------- ---------- ---------- LSIHLFDVGG QRSERKKWIH
Umaydisgpa4 ---------- ---------- ---------- ---------- LIYRIYDVGG SRSQRAAWAP

```
                           1201
AthalianaGPA1              LFE-GVTAVI FCAAISEYDQ TLFEDEQ--- ---------- ---------- -------KNR
CalbicansGPA1              CFE-DITAVL FVLAISEYDQ NLFEDER--- ---------- ---------- -------VNR
CalbicansGPA2              CFD-NVSTII FCVALSEYDN FLLEESN--- ---------- ---------- -------TNR
Celeganseg130              CFE-NVTSIM FLVALSEYDQ VLVECDN--- ---------- ---------- -------ENR
Celegansgoa1               CFE-DVTAII FCVAMSEYDQ VLHEDET--- ---------- ---------- -------TNR
Celegansgpa1               CFD-DAKAMI YVASLSEYDQ VLLEDNT--- ---------- ---------- -------TNR
Celegansgpa10              FFD-EADAVL FVAAISEFDQ KLAEDEE--- ---------- ---------- -------TNR
Celegansgpa11              CFD-NVNGVL FIAAISGYNL YDEDEEN--- ---------- --------RK DDGTPTKTNR
Celegansgpa12              CFT-DITSIL FMVASNEYDQ VILEDRR--- ---------- ---------- -------TNR
Celegansgpa13              QYE-GIDAIF FCIAISEYDQ VMSEDMV--- ---------- ---------- -------TNR
Celegansgpa14              LFD-DAKVVL FVIDLTGYAK RSEESRM--- ---------- ---------- ------ELSR
Celegansgpa15              LFD-NVNATI FISAINEFNQ KLNEDGQ--- ---------- ---------- -------TNR
Celegansgpa16              CFE-DVTALI FCVALSEYDM VLVEDCQ--- ---------- ---------- -------TNR
Celegansgpa17              CFD-NVESII FITAISEYDQ VLFEDET--- ---------- ---------- -------TNR
Celegansgpa2               CFE-DVNAII FIAALSEYNE VLFEDET--- ---------- ---------- -------TNR
Celegansgpa3               CFE-DVNAII FIAAISEYDQ VLFEDET--- ---------- ---------- -------TNR
Celegansgpa4               CFD-SVTAVI FCVALSEYDL RLAEDQT--- ---------- ---------- -------MNR
Celegansgpa5               FFE-GVTAVM FVCSLASFNQ TTEEEPKAFV WESSLNKVQN KVLVRSAGKA KVEKPGLINR
Celegansgpa6               CFD-NVDMIL FIVSMSDYDQ LDPEDNK--- ---------- ---------- -------YNR
Celegansgpa7               CFD-NVNAII FISSLSEYDQ TLREDNC--- ---------- ---------- -------TNR
Celegansgpa8               VFD-DVQAVL FITSLSEYDQ VLREDAT--- ---------- ---------- -------VNR
Celegansgpa9               YFD-DPNLVV FVIDLSELCD PAFYNKG--- ---------- ---------- -----YLENK
Celegansgsa1               CFN-DVTAII FVCASSSYNL VLWEDNT--- ---------- ---------- -------QNR
CglabrataGPA1              CFE-GITAVL FVLALSEYDQ MLFEDST--- ---------- ---------- -------VNR
CglabrataGPA2              CFD-NVTLVI FCVSLSEYDQ FLMEDKS--- ---------- ---------- -------QNR
DmelanogasterCTA           CFDSSVTSII FLVSSSEFDQ VLAEDRK--- ---------- ---------- -------TNR
DmelanogasterGalphaf       VFE-GIQAVL FLISCSEFDQ NLREDPS--- ---------- ---------- -------QNR
DmelanogasterGalphai       CFE-GVTAII FCVALSGYDL VLAEDEE--- ---------- ---------- -------MNR
DmelanogasterGalphao       CFE-DVTAII FCVAMSEYDQ VLHEDET--- ---------- ---------- -------TNR
DmelanogasterGalphaq       CFE-NVTSII FLVALSEYDQ ILFESDN--- ---------- ---------- -------ENR
DmelanogasterGalphas       CFN-DVTAII FVTACSSYNM VLREDPT--- ---------- ---------- -------QNR
Dreriogna11                CFE-NVTSIM FLVALSEYDQ VLVESDN--- ---------- ---------- -------ENR
Dreriogna11a               CFE-NVTSIM FLVALSEYDQ VLVESDN--- ---------- ---------- -------ENR
Dreriogna12                CFD-GITSIL FMVSSSEYDQ VLMEDRR--- ---------- ---------- -------TNR
Dreriogna13                CFD-SVTSIL FLVSSSEYDQ VLMEDRQ--- ---------- ---------- -------TNR
Dreriogna14a               CFE-NVTSII FLVALSEYDQ VLAECDN--- ---------- ---------- -------ENR
Dreriogna12                CFN-GI---- ---------- ---------- ---------- ---------- ----------
Dreriognas                 CFN-DVTAII FVVASSSYNM VLREDNQ--- ---------- ---------- -------TNR
Dreriognav1                CFE-YVRSVL FVVSLSGYDM TLVEDPS--- ---------- ---------- -------MNR
Dreriognaz                 CFE-GVTAII FCVELSGYDL KLYEDNQ--- ---------- ---------- -------TSR
HsapiensGNA11              CFE-NVTSIM FLVALSEYDQ VLVESDN--- ---------- ---------- -------ENR
HsapiensGNA12              CFD-GITSIL FMVSSSEYDQ VLMEDRR--- ---------- ---------- -------TNR
HsapiensGNA13              CFD-SVTSIL FLVSSSEFDQ VLMEDRL--- ---------- ---------- -------TNR
HsapiensGNA14              CFE-SVTSII FLVALSEYDQ VLAECDN--- ---------- ---------- -------ENR
HsapiensGNA15              CFE-NVIALI YLASLSEYDQ CLEENNQ--- ---------- ---------- -------ENR
HsapiensGNAI1              CFE-GVTAII FCVALSDYDL VLAEDEE--- ---------- ---------- -------MNR
HsapiensGNAI2              CFE-GVTAII FCVALSAYDL VLAEDEE--- ---------- ---------- -------MNR
HsapiensGNAI3              CFE-GVTAII FCVALSDYDL VLAEDEE--- ---------- ---------- -------MNR
HsapiensGNAL               CFN-DVTAII YVAACSSYNM VIREDNN--- ---------- ---------- -------TNR
HsapiensGNAO1              CFE-DVTAII FCVALSGYDQ VLHEDET--- ---------- ---------- -------TNR
HsapiensGNAQ               CFE-NVTSIM FLVALSEYDQ VLVESDN--- ---------- ---------- -------ENR
HsapiensGNAS1              CFN-DVTAII FVVASSSYNM VIREDNQ--- ---------- ---------- -------TNR
HsapiensGNAS2              CFN-DVTAII FVVASSSYNM VIREDNQ--- ---------- ---------- -------TNR
HsapiensGNAT1              CFE-GVTCII FIAALSAYDM VLVEDDE--- ---------- ---------- -------VNR
HsapiensGNAT2              CFE-GVTCII FCAALSAYDM VLVEDDE--- ---------- ---------- -------VNR
HsapiensGNAT3              CFE-GVTCII FCAALSAYDM VLVEDEE--- ---------- ---------- -------VNR
HsapiensGNAZ               CFE-GVTAII FCVELSGYDL KLYEDNQ--- ---------- ---------- -------TSR
Ncrassagna1                CFE-NVTTIL FLVAISEYDQ LLFEDET--- ---------- ---------- -------VNR
Ncrassagna2                CFE-NVNCLL FLVAISGYDQ CLVEDKD--- ---------- ---------- -------GNQ
Ncrassagna3                CFE-NVTSII FCVALSEYDQ VLLEESS--- ---------- ---------- -------QNR
ScervisiaeGPA1             CFE-GITAVL FVLAMSEYDQ MLFEDER--- ---------- ---------- -------VNR
ScervisiaeGPA2             CFD-NVTLVI FCVSLSEYDQ TLMEDKN--- ---------- ---------- -------QNR
SparadoxusGPA1             CFE-GITAVL FVLAMSEYDQ MLFEDER--- ---------- ---------- -------VNR
SparadoxusGPA2             CFD-NVTLVI FCVSLSEYDQ ALMEDKN--- ---------- ---------- -------QNR
Umaydisgpa1                CFE-NVTAII FLVAISEYDQ LLYEDEN--- ---------- ---------- -------VNR
Umaydisgpa2                CFE-NVTAVL FCVALSGYDS CLVEDKD--- ---------- ---------- -------SNQ
Umaydisgpa3                CFE-AVTSII FCVALSEYDQ VLLEESG--- ---------- ---------- -------QNR
Umaydisgpa4                FLD-DIESII FLAPLSAFDQ PLVEDCS--- ---------- ---------- -------TNR
```

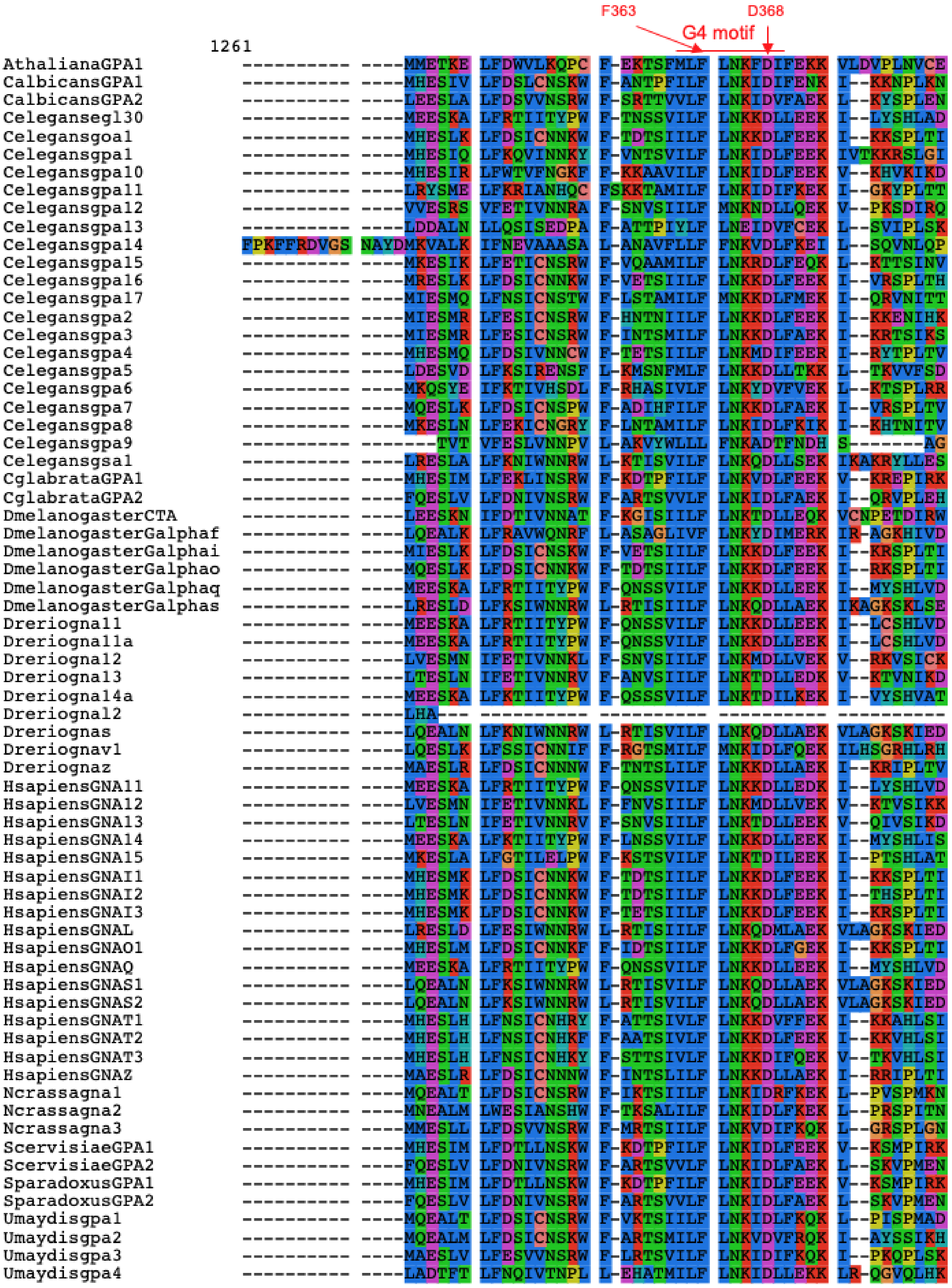

F363
D368
G4 motif
1261
AthalianaGPA1 ----------- ----MMETKE LFDWVLKQPC F-EKTSFMLF LNKFDIFEKK VLDVPLNVCE
CalbicansGPA1 ----------- ----MHESIV LFDSLCNSKW F-ANTPFILF LNKIDIFENK I--KKNPLKN
CalbicansGPA2 ----------- ----LEESLA LFDSVVNSRW F-SRTTVVLF LNKIDVFAEK L--KYSPLEN
Celeganseg130 ----------- ----MEESKA LFRTIITYPW F-TNSSVILF LNKKDLLEEK I--LYSHLAD
Celegansgoa1 ----------- ----MHESLK LFDSICNNKW F-TDTSIILF LNKKDLFEEK I--KKSPLTI
Celegansgpa1 ----------- ----MHESIQ LFKQVINNKY F-VNTSVILF LNKIDLFEEK IVTKKRSLGI
Celegansgpa10 ----------- ----MHESIR LFWTVFNGKF F-KKAAVILF LNKIDLFEEK V--KHVKIKD
Celegansgpa11 ----------- ----LRYSME LFKRIANHQC FSKKTAMILF LNKIDIFKEK I--GKYPLTT
Celegansgpa12 ----------- ----VVESRS VFETIVNNRA F-SNVSIILF MNKNDLLQEK V--PKSDIRQ
Celegansgpa13 ----------- ----LDDALN LLQSISEDPA F-ATTPIYLF LNEIDVFCEK L--SVIPLSK
Celegansgpa14 FPKFFRDVGS NAYDMKVALK IFNEVAAASA L-ANAVFLLF FNKVDLFKEI L--SQVNLQP
Celegansgpa15 ----------- ----MKESIK LFETICNSRW F-VQAAMILF LNKRDLFEQK L--KTTSINV
Celegansgpa16 ----------- ----MRESLK LFDSICNNKW F-VETSIILF LNKKDLFEEK I--VRSPLTH
Celegansgpa17 ----------- ----MIESMQ LFNSICNSTW F-LSTAMILF MNKKDLFMEK I--QRVNITT
Celegansgpa2 ----------- ----MIESMR LFESICNSRW F-HNTNIILF LNKKDLFEEK I--KKENIHK
Celegansgpa3 ----------- ----MIESMR LFESICNSRW F-INTSMILF LNKKDLFAEK I--KRTSIKS
Celegansgpa4 ----------- ----MHESMQ LFDSIVNNCW F-TETSIILF LNKMDIFEER I--RYTPLTV
Celegansgpa5 ----------- ----LDESVD LFKSIRENSF L-KMSNFMLF LNKKDLLTKK L--TKVVFSD
Celegansgpa6 ----------- ----MKQSYE IFKTIVHSDL F-RHASIVLF LNKYDVFVEK L--KTSPLRR
Celegansgpa7 ----------- ----MQESLK LFDSICNSPW F-ADIHFILF LNKKDLFAEK I--VRSPLTV
Celegansgpa8 ----------- ----MKESLN LFEKICNGRY F-LNTAMILF LNKIDLFKIK I--KHTNITV
Celegansgpa9 ----------- -------TVT VFESLVNNPV L-AKVYWLLL FNKADTFNDH S-------AG
Celegansgsa1 ----------- ----LRESLA LFKNIWNNRW L-KTISVILF LNKQDLLSEK IKAKRYLLES
CglabrataGPA1 ----------- ----MHESIM LFEKLINSRW F-KDTPFILF LNKVDLFEEK V--KREPIRK
CglabrataGPA2 ----------- ----FQESLV LFDNIVNSRW F-ARTSVVLF LNKIDLFAEK I--QRVPLEH
DmelanogasterCTA ----------- ----LEESKN IFDTIVNNAT F-KGISIILF LNKTDLLEQK VCNPETDIRW
DmelanogasterGalphaf ----------- ----LQEALK LFRAVWQNRF L-ASAGLIVF LNKYDIMERK IR-AGKHIVD
DmelanogasterGalphai ----------- ----MIESLK LFDSICNSKW F-VETSIILF LNKKDLFEEK I--KRSPLTI
DmelanogasterGalphao ----------- ----MQESLK LFDSICNNKW F-TDTSIILF LNKKDLFEEK I--RKSPLTI
DmelanogasterGalphaq ----------- ----MEESKA LFRTIITYPW F-QNSSVILF LNKKDLLEEK I--MYSHLVD
DmelanogasterGalphas ----------- ----LRESLD LFKSIWNNRW L-RTISIILF LNKQDLLAEK IKAGKSKLSE
Dreriogna11 ----------- ----MEESKA LFRTIITYPW F-QNSSVILF LNKKDLLEEK I--LCSHLVD
Dreriogna11a ----------- ----MEESKA LFRTIITYPW F-QNSSVILF LNKKDLLEEK I--LCSHLVD
Dreriogna12 ----------- ----LVESMN IFETIVNNKL F-SNVSIILF LNKMDLLVEK V--RKVSICK
Dreriogna13 ----------- ----LTESLN IFETIVNNRV F-ANVSIILF LNKTDLLEDK V--KTVNIKD
Dreriogna14a ----------- ----MEESKA LFKTIITYPW F-QSSSVILF LNKTDILKEK I--VYSHVAT
Dreriogna12 ----------- ----LHA--- ---------- ---------- ---------- ----------
Dreriognas ----------- ----LQEALN LFKNIWNNRW L-RTISVILF LNKQDLLAEK VLAGKSKIED
Dreriognav1 ----------- ----LQESLK LFSSICNNIF F-RGTSMILF MNKIDLFQEK ILHSGRHLRH
Dreriognaz ----------- ----MAESLR LFDSICNNNW F-TNTSLILF LNKKDLLAEK I--KRIPLTV
HsapiensGNA11 ----------- ----MEESKA LFRTIITYPW F-QNSSVILF LNKKDLLEDK I--LYSHLVD
HsapiensGNA12 ----------- ----LVESMN IFETIVNNKL F-FNVSIILF LNKMDLLVEK V--KTVSIKK
HsapiensGNA13 ----------- ----LTESLN IFETIVNNRV F-SNVSIILF LNKTDLLEEK V--QIVSIKD
HsapiensGNA14 ----------- ----MEESKA LFKTIITYPW F-LNSSVILF LNKKDLLEEK I--MYSHLIS
HsapiensGNA15 ----------- ----MKESLA LFGTILELPW F-KSTSVILF LNKTDILEEK I--PTSHLAT
HsapiensGNAI1 ----------- ----MHESMK LFDSICNNKW F-TDTSIILF LNKKDLFEEK I--KKSPLTI
HsapiensGNAI2 ----------- ----MHESMK LFDSICNNKW F-TDTSIILF LNKKDLFEEK I--THSPLTI
HsapiensGNAI3 ----------- ----MHESMK LFDSICNNKW F-TETSIILF LNKKDLFEEK I--KRSPLTI
HsapiensGNAL ----------- ----LRESLD LFESIWNNRW L-RTISIILF LNKQDMLAEK VLAGKSKIED
HsapiensGNAO1 ----------- ----MHESLM LFDSICNNKF F-IDTSIILF LNKKDLFGEK I--KKSPLTI
HsapiensGNAQ ----------- ----MEESKA LFRTIITYPW F-QNSSVILF LNKKDLLEEK I--MYSHLVD
HsapiensGNAS1 ----------- ----LQEALN LFKSIWNNRW L-RTISVILF LNKQDLLAEK VLAGKSKIED
HsapiensGNAS2 ----------- ----LQEALN LFKSIWNNRW L-RTISVILF LNKQDLLAEK VLAGKSKIED
HsapiensGNAT1 ----------- ----MHESLH LFNSICNHRY F-ATTSIVLF LNKKDVFFEK I--KKAHLSI
HsapiensGNAT2 ----------- ----MHESLH LFNSICNHKF F-AATSIVLF LNKKDLFEEK I--KKVHLSI
HsapiensGNAT3 ----------- ----MHESLH LFNSICNHKY F-STTSIVLF LNKKDIFQEK V--TKVHLSI
HsapiensGNAZ ----------- ----MAESLR LFDSICNNNW F-INTSLILF LNKKDLLAEK I--RRIPLTI
Ncrassagna1 ----------- ----MQEALT LFDSICNSRW F-IKTSIILF LNKIDRFKEK L--PVSPMKN
Ncrassagna2 ----------- ----MNEALM LWESIANSHW F-TKSALILF LNKIDLFKEK L--PRSPITN
Ncrassagna3 ----------- ----MMESLL LFDSVVNSRW F-MRTSIILF LNKVDIFKQK L--GRSPLGN
ScervisiaeGPA1 ----------- ----MHESIM LFDTLLNSKW F-KDTPFILF LNKIDLFEEK V--KSMPIRK
ScervisiaeGPA2 ----------- ----FQESLV LFDNIVNSRW F-ARTSVVLF LNKIDLFAEK L--SKVPMEN
SparadoxusGPA1 ----------- ----MHESIM LFDTLLNSKW F-KDTPFILF LNKIDLFEEK V--KSMPIRK
SparadoxusGPA2 ----------- ----FQESLV LFDNIVNSRW F-ARTSVVLF LNKIDLFAEK L--SKVPMEN
Umaydisgpa1 ----------- ----MQEALT LFDSICNSRW F-VKTSIILF LNKIDLFKQK L--PISPMAD
Umaydisgpa2 ----------- ----MQEALM LFDSICNSKW F-ARTSMILF LNKVDVFRQK I--AYSSIKH
Umaydisgpa3 ----------- ----MAESLV LFESVVNSRW F-LRTSVILF LNKIDIFKQK I--PKQPLSK
Umaydisgpa4 ----------- ----LADTFT LFNQIVTNPL L-EHATMILF LNKIDLLEKK LR-QGVQLHK

```
                     1321
AthalianaGPA1        W-FRDY---- -----QPVSS ---------- --GKQEIEHA YEFVKKK--- -----FEEL-
CalbicansGPA1        Y-FPDY---- --------DG ---------- --KPDDTNEA IKFFETN--- -----FLKI-
CalbicansGPA2        F-FPDY---- --------KG ---------- --G-SNISNG VKYILWR--- -----FNKL-
Celegansegl30        Y-FPEY---- --------DG ---------- --PPRDPIAA REFILKM--- -----FVDL-
Celegansgoa1         C-FPEY---- --------SG ---------- --R-QDYHEA SAYIQAQ--- -----FEAK-
Celegansgpa1         A-FESF---- --------SG ---------- --PSQDLNAA VAFVEKK--- -----YRSM-
Celegansgpa10        Y-FPKF---- --------EG ---------- --A-NTVSEG TKFFRRQ--- -----FREGI
Celegansgpa11        C-FKNY---- --------KG ---------- --V-NAFEPA CKYVTDR--- -----FSRL-
Celegansgpa12        Y-FTDF---- --------TG ---------- --DHTLVRDV QFFLVDK--- -----FEAS-
Celegansgpa13        Y-KPDF---- --------KG ---------- --G--DQDDA IDFMENL--- ----ACEAL-
Celegansgpa14        C-FSKF---- --------DG ---------- --E-NTYEET SKYICEK--- -----FIRA-
Celegansgpa15        L-FSTY---- --------QG ---------- --S-NDYAEC VAYIQMR--- -----FERL-
Celegansgpa16        C-FPEY---- --------TG ---------- --A-NNYEEA SAYIQQQ--- -----FEDM-
Celegansgpa17        A-FPDY---- --------EG ---------- --G-QNYEEA VSFIKQK--- -----FAEL-
Celegansgpa2         A-FPEY---- --------RG ---------- --E-QNYAET VAFIKTK--- -----FEAL-
Celegansgpa3         A-FPDY---- --------KG ---------- --A-QTYDES CRYIEEK--- -----FDGL-
Celegansgpa4         C-FPEY---- --------QG ---------- --G-MTITET STFIQSR--- -----FEIL-
Celegansgpa5         Y-FPDY---- --------KK WIT------- --NDNSDVSV AEFIENM--- -----FRE--
Celegansgpa6         S-FKNY---- --------EG ---------- --D-NSEESA REFIKKL--- -----FRRC-
Celegansgpa7         C-FPEY---- --------KG ---------- --Q-QNQTEC INYIQWK--- -----FEQL-
Celegansgpa8         A-LTSY---- --------KG ---------- --P-QECDSA LDYIRKR--- -----FISL-
Celegansgpa9         FDFRKL---- --------AN ---------- --HLDTADSA RSFYRSQ--- -----FTTK-
Celegansgsa1         F-FPEF---- --------EG YNLPNDAVFD NQEDKDVVRA KYFIRGE--- -----FLRI-
CglabrataGPA1        Y-FPDY---- --------PG ---------- --KVGDEKAG IKYFEKL--- -----FLSL-
CglabrataGPA2        Y-FPDY---- --------MG ---------- --G-KDINKA AKYILWR--- -----FVQL-
DmelanogasterCTA     Y-YPHF---- --------NG ---------- --NPHSVLDV QNFILQM--- -----FMSV-
DmelanogasterGalphaf Y-FPEYEDFC KRPQQ---DN ---------- --CFGESDWT KMFIKQKLVD ITQEPFKRH-
DmelanogasterGalphai C-FPEY---- --------TG ---------- --T-NTFEEA ANYIRMK--- -----FENL-
DmelanogasterGalphao C-FPEY---- --------TG ---------- --G-QEYGEA AAYIQAQ--- -----FEAK-
DmelanogasterGalphaq Y-FPEY---- --------DG ---------- --PKQDHAAA KQFVLKK--- -----YLAC-
DmelanogasterGalphas Y-FSEFNKYQ TPID----TG -----DAIME SNDDPEVIRA KYFIRDE--- -----FLRI-
Dreriogna11          Y-FPEF---- --------DG ---------- --PQNDAQSA REFILKM--- -----FVDL-
Dreriogna11a         Y-FPEF---- --------DG ---------- --PQNDAQSA REFILKM--- -----FVDL-
Dreriogna12          H-FSDF---- --------RG ---------- --DPHRLVDV QAYLVQC--- -----FNRK-
Dreriogna13          Y-FSEF---- --------TG ---------- --EPHNLQDV QKFLVEC--- -----FRNK-
Dreriogna14a         Y-FPEF---- --------TG ---------- --PKNDPKAA QEFILKM--- -----YQEE-
Dreriognal2          ---------- ---------- ---------- ---------- ---------- ----------
Dreriognas           Y-FPEFARYT TPDDATPEQG ---------- --EDPRVTRA KYFIRDE--- -----FLRI-
Dreriognav1          Y-LPQF---- --------RG ---------- --ADCDVDAA ARFIADM--- -----FVSL-
Dreriognaz           C-FADY---- --------KG ---------- --Q-NTYEEA AVYVQRQ--- -----FEDL-
HsapiensGNA11        Y-FPEF---- --------DG ---------- --PQRDAQAA REFILKM--- -----FVDL-
HsapiensGNA12        H-FPDF---- --------RG ---------- --DPHRLEDV QRYLVQC--- -----FDRK-
HsapiensGNA13        Y-FLEF---- --------EG ---------- --DPHCLRDV QKFLVEC--- -----FRNK-
HsapiensGNA14        Y-FPEY---- --------TG ---------- --PKQDVRAA RDFILKL--- -----YQDQ-
HsapiensGNA15        Y-FPSF---- --------QG ---------- --PKQDAEAA KRFILDM--- -----YTRMY
HsapiensGNAI1        C-YPEY---- --------AG ---------- --S-NTYEEA AAYIQCQ--- -----FEDL-
HsapiensGNAI2        C-FPEY---- --------TG ---------- --A-NKYDEA ASYIQSK--- -----FEDL-
HsapiensGNAI3        C-YPEY---- --------TG ---------- --S-NTYEEA AAYIQCQ--- -----FEDL-
HsapiensGNAL         Y-FPEYANYT VPEDATPDAG ---------- --EDPKVTRA KFFIRDL--- -----FLRI-
HsapiensGNAO1        C-FPEY---- --------TG ---------- --P-NTYEDA AAYIQAQ--- -----FESK-
HsapiensGNAQ         Y-FPEY---- --------DG ---------- --PQRDAQAA REFILKM--- -----FVDL-
HsapiensGNAS1        Y-FPEFARYT TPEDATPEPG ---------- --EDPRVTRA KYFIRDE--- -----FLRI-
HsapiensGNAS2        Y-FPEFARYT TPEDATPEPG ---------- --EDPRVTRA KYFIRDE--- -----FLRI-
HsapiensGNAT1        C-FPDY---- --------DG ---------- --P-NTYEDA GNYIKVQ--- -----FLEL-
HsapiensGNAT2        C-FPEY---- --------DG ---------- --N-NSYDDA GNYIKSQ--- -----FLDL-
HsapiensGNAT3        C-FPEY---- --------TG ---------- --P-NTFEDA GNYIKNQ--- -----FLDL-
HsapiensGNAZ         C-FPEY---- --------KG ---------- --Q-NTYEEA AVYIQRQ--- -----FEDL-
Ncrassagna1          Y-FPDY---- --------EG ---------- --G-DDYAAA CDYILNR--- -----FVSL-
Ncrassagna2          HGFTDY---- --------HG ---------- --PPDDSKQA SKYFMDK--- -----FRAL-
Ncrassagna3          Y-FPDY---- --------SG ---------- --G-NDVNKA AKYLLWR--- -----FNQV-
ScervisiaeGPA1       Y-FPDY---- --------QG ---------- --RVGDAEAG LKYFEKI--- -----FLSL-
ScervisiaeGPA2       Y-FPDY---- --------TG ---------- --G-SDINKA AKYILWR--- -----FVQL-
SparadoxusGPA1       Y-FPDY---- --------QG ---------- --RVGDAEAG LRYFEKI--- -----FLSL-
SparadoxusGPA2       Y-FPDY---- --------TG ---------- --G-SDINKA AKYILWR--- -----FVQL-
Umaydisgpa1          Y-FSDY---- --------TG ---------- --G-ADYNSA SEYIVNR--- -----FVSL-
Umaydisgpa2          Y-FPDY---- --------DG ---------- --DDQDFNAA RSYFKAR--- -----FCRL-
Umaydisgpa3          Y-FPEY---- --------SG ---------- --G-PDINKA AKYILWR--- -----FTQT-
Umaydisgpa4          Y-WPEY---- --------VG ---------- --D-NDFEAV WRWFRAK--- -----FRDAL
```

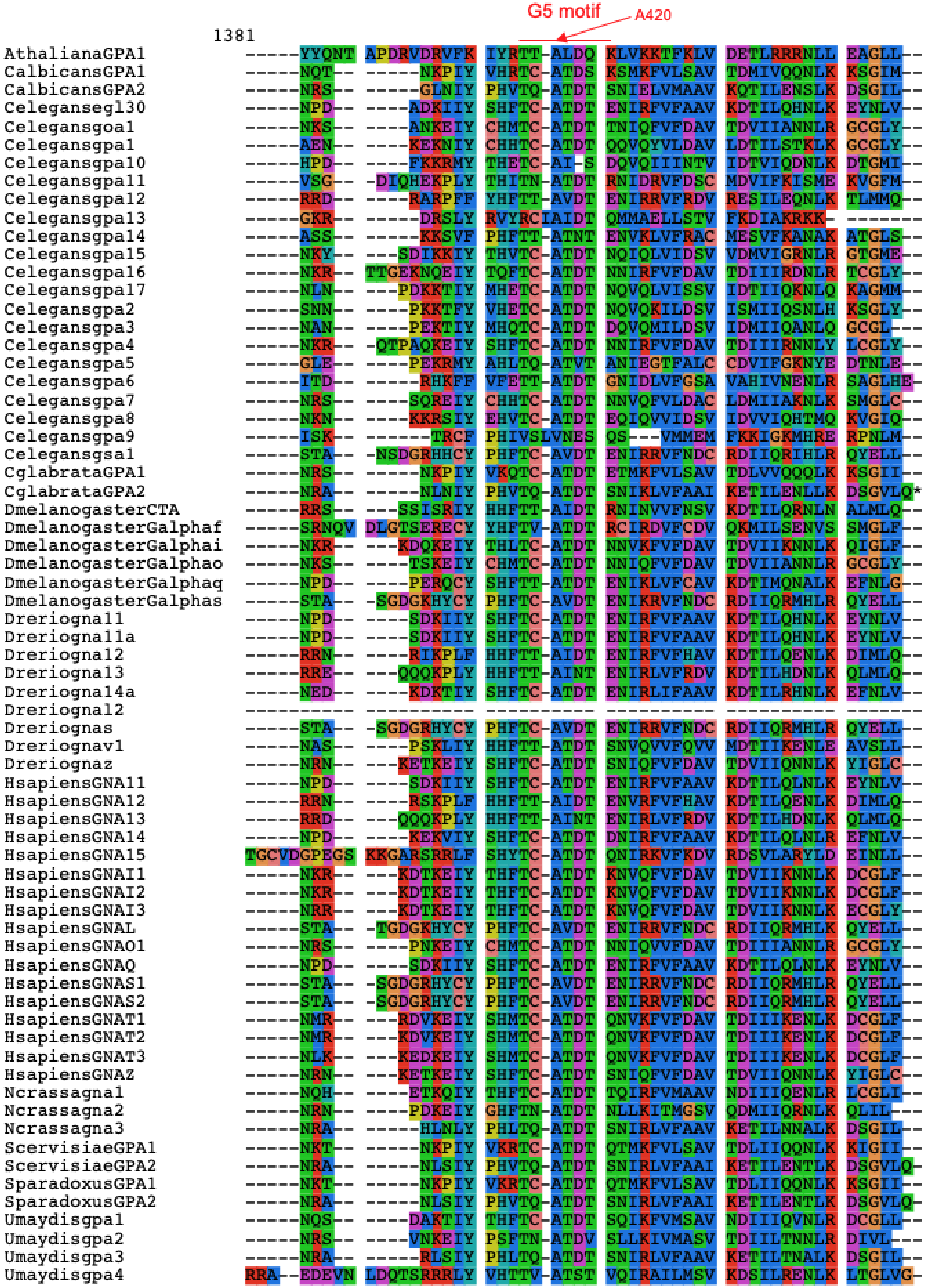
G5 motif
A420
1381
AthalianaGPA1 -----YYQNT APDRVDRVFK IYRTT-ALDQ KLVKKTFKLV DETLRRRNLL EAGLL--
CalbicansGPA1 -----NQT-- -----NKPIY VHRTC-ATDS KSMKFVLSAV TDMIVQQNLK KSGIM--
CalbicansGPA2 -----NRS-- -----GLNIY PHVTQ-ATDT SNIELVMAAV KQTILENSLK DSGIL--
Celegansegl30 -----NPD-- ----ADKIIY SHFTC-ATDT ENIRFVFAAV KDTILQHNLK EYNLV--
Celegansgoa1 -----NKS-- ----ANKEIY CHMTC-ATDT TNIQFVFDAV TDVIIANNLR GCGLY--
Celegansgpa1 -----AEN-- ----KEKNIY CHHTC-ATDT QQVQYVLDAV LDTILSTKLK GCGLY--
Celegansgpa10 -----HPD-- ----FKKRMY THETC-AI-S DQVQIIINTV IDTVIQDNLK DTGMI--
Celegansgpa11 -----VSG-- -DIQHEKPLY THITN-ATDT RNIDRVFDSC MDVIFKISME KVGFM--
Celegansgpa12 -----RRD-- ----RARPFF YHFTT-AVDT ENIRRVFRDV RESILEQNLK TLMMQ--
Celegansgpa13 -----GKR-- -----DRSLY RVYRCIAIDT QMMAELLSTV FKDIAKRKK- -------
Celegansgpa14 -----ASS-- -----KKSVF PHFTT-ATNT ENVKLVFRAC MESVFKANAK ATGLS--
Celegansgpa15 -----NKY-- ---SDIKKIY THVTC-ATDT NQIQLVIDSV VDMVIGRNLR GTGME--
Celegansgpa16 -----NKR-- TTGEKNQEIY TQFTC-ATDT NNIRFVFDAV TDIIIRDNLR TCGLY--
Celegansgpa17 -----NLN-- ---PDKKTIY MHETC-ATDT NQVQLVISSV IDTIIQKNLQ KAGMM--
Celegansgpa2 -----SNN-- ----PKKTFY VHETC-ATDT NQVQKILDSV ISMIIQSNLH KSGLY--
Celegansgpa3 -----NAN-- ----PEKTIY MHQTC-ATDT DQVQMILDSV IDMIIQANLQ GCGL---
Celegansgpa4 -----NKR-- -QTPAQKEIY SHFTC-ATDT NNIRFVFDAV TDIIIRNNLY LCGLY--
Celegansgpa5 -----GLE-- ----PEKRMY AHLTQ-ATVT ANIEGTFALC CDVIFGKNYE DTNLE--
Celegansgpa6 -----ITD-- -----RHKFF VFETT-ATDT GNIDLVFGSA VAHIVNENLR SAGLHE-
Celegansgpa7 -----NRS-- ----SQREIY CHHTC-ATDT NNVQFVLDAC LDMIIAKNLK SMGLC--
Celegansgpa8 -----NKN-- ----KKRSIY EHVTC-ATDT EQIQVVIDSV IDVVIQHTMQ KVGIQ--
Celegansgpa9 -----ISK-- ------TRCF PHIVSLVNES QS---VMMEM FKKIGKMHRE RPNLM--
Celegansgsa1 -----STA-- -NSDGRHHCY PHFTC-AVDT ENIRRVFNDC RDIIQRIHLR QYELL--
CglabrataGPA1 -----NRS-- -----NKPIY VKQTC-ATDT ETMKFVLSAV TDLVVQQQLK KSGII--
CglabrataGPA2 -----NRA-- -----NLNIY PHVTQ-ATDT SNIKLVFAAI KETILENLLK DSGVLQ*
DmelanogasterCTA -----RRS-- ---SSISRIY HHFTT-AIDT RNINVVFNSV KDTILQRNLN ALMLQ--
DmelanogasterGalphaf -----SRNQV DLGTSERECY YHFTV-ATDT RCIRDVFCDV QKMILSENVS SMGLF--
DmelanogasterGalphai -----NKR-- ---KDQKEIY THLTC-ATDT NNVKFVFDAV TDVIIKNNLK QIGLF--
DmelanogasterGalphao -----NKS-- ----TSKEIY CHMTC-ATDT NNIQFVFDAV TDVIIANNLR GCGLY--
DmelanogasterGalphaq -----NPD-- ----PERQCY SHFTT-ATDT ENIKLVFCAV KDTIMQNALK EFNLG--
DmelanogasterGalphas -----STA-- -SGDGKHYCY PHFTC-AVDT ENIKRVFNDC RDIIQRMHLR QYELL--
Dreriognal1 -----NPD-- ----SDKIIY SHFTC-ATDT ENIRFVFAAV KDTILQHNLK EYNLV--
Dreriognal1a -----NPD-- ----SDKIIY SHFTC-ATDT ENIRFVFAAV KDTILQHNLK EYNLV--
Dreriognal2 -----RRN-- ----RIKPLF HHFTT-AIDT ENIRFVFHAV KDTILQENLK DIMLQ--
Dreriognal3 -----RRE-- ---QQQKPLY HHFTT-AINT ENIRLVFRDV KDTILHDNLK QLMLQ--
Dreriognal4a -----NED-- ----KDKTIY SHFTC-ATDT ENIRLIFAAV KDTILRHNLK EFNLV--
Dreriognal2 ---------- ---------- ---------- ---------- ---------- -------
Dreriognas -----STA-- -SGDGRHYCY PHFTC-AVDT ENIRRVFNDC RDIIQRMHLR QYELL--
Dreriognav1 -----NAS-- ----PSKLIY HHFTT-ATDT SNVQVVFQVV MDTIIKENLE AVSLL--
Dreriognaz -----NRN-- ---KETKEIY SHFTC-ATDT SNIQFVFDAV TDVIIQNNLK YIGLC--
HsapiensGNA11 -----NPD-- ----SDKIIY SHFTC-ATDT ENIRFVFAAV KDTILQLNLK EYNLV--
HsapiensGNA12 -----RRN-- ----RSKPLF HHFTT-AIDT ENVRFVFHAV KDTILQENLK DIMLQ--
HsapiensGNA13 -----RRD-- ---QQQKPLY HHFTT-AINT ENIRLVFRDV KDTILHDNLK QLMLQ--
HsapiensGNA14 -----NPD-- ----KEKVIY SHFTC-ATDT DNIRFVFAAV KDTILQLNLR EFNLV--
HsapiensGNA15 TGCVDGPEGS KKGARSRRLF SHYTC-ATDT QNIRKVFKDV RDSVLARYLD EINLL--
HsapiensGNAI1 -----NKR-- ---KDTKEIY THFTC-ATDT KNVQFVFDAV TDVIIKNNLK DCGLF--
HsapiensGNAI2 -----NKR-- ---KDTKEIY THFTC-ATDT KNVQFVFDAV TDVIIKNNLK DCGLF--
HsapiensGNAI3 -----NRR-- ---KDTKEIY THFTC-ATDT KNVQFVFDAV TDVIIKNNLK ECGLY--
HsapiensGNAL -----STA-- -TGDGKHYCY PHFTC-AVDT ENIRRVFNDC RDIIQRMHLK QYELL--
HsapiensGNAO1 -----NRS-- ----PNKEIY CHMTC-ATDT NNIQVVFDAV TDIIIANNLR GCGLY--
HsapiensGNAQ -----NPD-- ----SDKIIY SHFTC-ATDT ENIRFVFAAV KDTILQLNLK EYNLV--
HsapiensGNAS1 -----STA-- -SGDGRHYCY PHFTC-AVDT ENIRRVFNDC RDIIQRMHLR QYELL--
HsapiensGNAS2 -----STA-- -SGDGRHYCY PHFTC-AVDT ENIRRVFNDC RDIIQRMHLR QYELL--
HsapiensGNAT1 -----NMR-- ---RDVKEIY SHMTC-ATDT QNVKFVFDAV TDIIIKENLK DCGLF--
HsapiensGNAT2 -----NMR-- ---KDVKEIY SHMTC-ATDT QNVKFVFDAV TDIIIKENLK DCGLF--
HsapiensGNAT3 -----NLK-- ---KEDKEIY SHMTC-ATDT QNVKFVFDAV TDIIIKENLK DCGLF--
HsapiensGNAZ -----NRN-- ---KETKEIY SHFTC-ATDT SNIQFVFDAV TDVIIQNNLK YIGLC--
Ncrassagna1 -----NQH-- ----ETKQIY THFTC-ATDT TQIRFVMAAV NDIIIQENLR LCGLI--
Ncrassagna2 -----NRN-- ----PDKEIY GHFTN-ATDT NLLKITMGSV QDMIIQRNLK QLIL---
Ncrassagna3 -----NRA-- -----HLNLY PHLTQ-ATDT SNIRLVFAAV KETILNNALK DSGIL--
ScervisiaeGPA1 -----NKT-- -----NKPIY VKRTC-ATDT QTMKFVLSAV TDLIIQQNLK KIGII--
ScervisiaeGPA2 -----NRA-- -----NLSIY PHVTQ-ATDT SNIRLVFAAI KETILENTLK DSGVLQ-
SparadoxusGPA1 -----NKT-- -----NKPIY VKRTC-ATDT QTMKFVLSAV TDLIIQQNLK KSGII--
SparadoxusGPA2 -----NRA-- -----NLSIY PHVTQ-ATDT SNIRLVFAAI KETILENTLK DSGVLQ-
Umaydisgpa1 -----NQS-- ----DAKTIY THFTC-ATDT SQIKFVMSAV NDIIIQVNLR DCGLL--
Umaydisgpa2 -----NRS-- ----VNKEIY PSFTN-ATDV SLLKIVMASV TDIILTNNLR DIVL---
Umaydisgpa3 -----NRA-- -----RLSIY PHLTQ-ATDT SNIRLVFAAV KETILTNALK DSGIL--
Umaydisgpa4 RRA--EDEVN LDQTSRRRLY VHTTV-ATST VQIRAILMSV KDSILRENLK LTGLVG-

Data S3. Seaview alignment of the Ras GTPase *RAS1* across 102 *Candida albicans* isolates, related to Figure 4. Amino acid change of interest is denoted by red arrows pointing at the column and row. Accession number and strain name for each strain is in *File S5*.

```
                      1
KE651087.1            MLREYKLVVV GGGGVGKSAL TIQLIQSHFV DEYDPTIEDS YRKQCTIDDQ QVLLDVLDTA
AJJE01000008.1        MLREYKLVVV GGGGVGKSAL TIQLIQSHFV DEYDPTIEDS YRKQCTIDDQ QVLLDVLDTA
AJJG01000018.1        MLREYKLVVV GGGGVGKSAL TIQLIQSHFV DEYDPTIEDS YRKQCTIDDQ QVLLDVLDTA
KE650847.1            MLREYKLVVV GGGGVGKSAL TIQLIQSHFV DEYDPTIEDS YRKQCTIDDQ QVLLDVLDTA
JAQJVS010000214.1     MLREYKLVVV GGGGVGKSAL TIQLIQSHFV DEYDPTIEDS YRKQCTIDDQ QVLLDVLDTA
JAMFPT010000137.1     MLREYKLVVV GGGGVGKSAL TIQLIQSHFV DEYDPTIEDS YRKQCTIDDQ QVLLDVLDTA
JSXQ01000010.1        MLREYKLVVV GGGGVGKSAL TIQLIQSHFV DEYDPTIEDS YRKQCTIDDQ QVLLDVLDTA
JAMFPV010000028.1     MLREYKLVVV GGGGVGKSAL TIQLIQSHFV DEYDPTIEDS YRKQCTIDDQ QVLLDVLDTA
AJJC01000012.1        MLREYKLVVV GGGGVGKSAL TIQLIQSHFV DEYDPTIEDS YRKQCTIDDQ QVLLDVLDTA
JADLIQ010000221.1     MLREYKLVVV GGGGVGKSAL TIQLIQSHFV DEYDPTIEDS YRKQCTIDDQ QVLLDVLDTA
NETO01000041.1        MLREYKLVVV GGGGVGKSAL TIQLIQSHFV DEYDPTIEDS YRKQCTIDDQ QVLLDVLDTA
KE650966.1            MLREYKLVVV GGGGVGKSAL TIQLIQSHFV DEYDPTIEDS YRKQCTIDDQ QVLLDVLDTA
KK667224.1            MLREYKLVVV GGGGVGKSAL TIQLIQSHFV DEYDPTIEDS YRKQCTIDDQ QVLLDVLDTA
JAQJVU010000255.1     MLREYKLVVV GGGGVGKSAL TIQLIQSHFV DEYDPTIEDS YRKQCTIDDQ QVLLDVLDTA
NETM01000037.1        MLREYKLVVV GGGGVGKSAL TIQLIQSHFV DEYDPTIEDS YRKQCTIDDQ QVLLDVLDTA
JADLIO010001687.1     MLREYKLVVV GGGGVGKSAL TIQLIQSHFV DEYDPTIEDS YRKQCTIDDQ QVLLDVLDTA
JAQJVN010000263.1     MLREYKLVVV GGGGVGKSAL TIQLIQSHFV DEYDPTIEDS YRKQCTIDDQ QVLLDVLDTA
JSXP01000007.1        MLREYKLVVV GGGGVGKSAL TIQLIQSHFV DEYDPTIEDS YRKQCTIDDQ QVLLDVLDTA
JADLIJ010001632.1     MLREYKLVVV GGGGVGKSAL TIQLIQSHFV DEYDPTIEDS YRKQCTIDDQ QVLLDVLDTA
NETN01000103.1        MLREYKLVVV GGGGVGKSAL TIQLIQSHFV DEYDPTIEDS YRKQCTIDDQ QVLLDVLDTA
JABWAD010000060.1     MLREYKLVVV GGGGVGKSAL TIQLIQSHFV DEYDPTIEIL IVNNVLLMIN KYYWMF*ILL
AJIZ01000014.1        MLREYKLVVV GGGGVGKSAL TIQLIQSHFV DEYDPTIEDS YRKQCTIDDQ QVLLDVLDTA
VBUY01000053.1        MLREYKLVVV GGGGVGKSAL TIQLIQSHFV DEYDPTIEDS YRKQCTIDDQ QVLLDVLDTA
KE650918.1            MLREYKLVVV GGGGVGKSAL TIQLIQSHFV DEYDPTIEDS YRKQCTIDDQ QVLLDVLDTA
CP032013.1            MLREYKLVVV GGGGVGKSAL TIQLIQSHFV DEYDPTIEDS YRKQCTIDDQ QVLLDVLDTA
JADLIN010001249.1     MLREYKLVVV GGGGVGKSAL TIQLIQSHFV DEYDPTIEDS YRKQCTIDDQ QVLLDVLDTA
JSXR01000016.1        MLREYKLVVV GGGGVGKSAL TIQLIQSHFV DEYDPTIEDS YRKQCTIDDQ QVLLDVLDTA
KE650780.1            MLREYKLVVV GGGGVGKSAL TIQLIQSHFV DEYDPTIEDS YRKQCTIDDQ QVLLDVLDTA
AJJF01000006.1        MLREYKLVVV GGGGVGKSAL TIQLIQSHFV DEYDPTIEDS YRKQCTIDDQ QVLLDVLDTA
JACXXM010000041.1     MLREYKLVVV GGGGVGKSAL TIQLIQSHFV DEYDPTIEDS YRKQCTIDDQ QVLLDVLDTA
CP017624.1            MLREYKLVVV GGGGVGKSAL TIQLIQSHFV DEYDPTIEDS YRKQCTIDDQ QVLLDVLDTA
JAMFPS010000366.1     MLREYKLVVV GGGGVGKSAL TIQLIQSHFV DEYDPTIEDS YRKQCTIDDQ QVLLDVLDTA
JAMADA010000074.1     MLREYKLVVV GGGGVGKSAL TIQLIQSHFV DEYDPTIEDS YRKQCTIDDQ QVLLDVLDTA
JADLIR010000106.1     MLREYKLVVV GGGGVGKSAL TIQLIQSHFV DEYDPTIEDS YRKQCTIDDQ QVLLDVLDTA
CP025151.1            MLREYKLVVV GGGGVGKSAL TIQLIQSHFV DEYDPTIEDS YRKQCTIDDQ QVLLDVLDTA
CP025159.1            MLREYKLVVV GGGGVGKSAL TIQLIQSHFV DEYDPTIEDS YRKQCTIDDQ QVLLDVLDTA
AJIU01000008.1        MLREYKLVVV GGGGVGKSAL TIQLIQSHFV DEYDPTIEDS YRKQCTIDDQ QVLLDVLDTA
AJIW01000013.1        MLREYKLVVV GGGGVGKSAL TIQLIQSHFV DEYDPTIEDS YRKQCTIDDQ QVLLDVLDTA
JAMACZ010000790.1     MLREYKLVVV GGGGVGKSAL TIQLIQSHFV DEYDPTIEDS YRKQCTIDDQ QVLLDVLDTA
KE650911.1            MLREYKLVVV GGGGVGKSAL TIQLIQSHFV DEYDPTIEDS YRKQCTIDDQ QVLLDVLDTA
JAKTXK010000203.1     MLREYKLVVV GGGGVGKSAL TIQLIQSHFV DEYDPTIEDS YRKQCTIDDQ QVLLDVLDTA
JAQJVY010000211.1     MLREYKLVVV GGGGVGKSAL TIQLIQSHFV DEYDPTIEDS YRKQCTIDDQ QVLLDVLDTA
JADLIK010000138.1     MLREYKLVVV GGGGVGKSAL TIQLIQSHFV DEYDPTIEDS YRKQCTIDDQ QVLLDVLDTA
JAHPZT010000270.1     MLREYKLVVV GGGGVGKSAL TIQLIQSHFV DEYDPTIEDS YRKQCTIDDQ QVLLDVLDTA
AJIQ01000011.1        MLREYKLVVV GGGGVGKSAL TIQLIQSHFV DEYDPTIEDS YRKQCTIDDQ QVLLDVLDTA
JADLIP010000819.1     MLREYKLVVV GGGGVGKSAL TIQLIQSHFV DEYDPTIEDS YRKQCTIDDQ QVLLDVLDTA
JAQJVT010000197.1     MLREYKLVVV GGGGVGKSAL TIQLIQSHFV DEYDPTIEDS YRKQCTIDDQ QVLLDVLDTA
AJIV01000014.1        MLREYKLVVV GGGGVGKSAL TIQLIQSHFV DEYDPTIEDS YRKQCTIDDQ QVLLDVLDTA
JAODHB010000006.1     MLREYKLVVV GGGGVGKSAL TIQLIQSHFV DEYDPTIEDS YRKQCTIDDQ QVLLDVLDTA
JAAGWN010000001.1     MLREYKLVVV GGGGVGKSAL TIQLIQSHFV DEYDPTIEDS YRKQCTIDDQ QVLLDVLDTA
JAQJVW010000188.1     MLREYKLVVV GGGGVGKSAL TIQLIQSHFV DEYDPTIEDS YRKQCTIDDQ QVLLDVLDTA
JPEW01000016.1        MLREYKLVVV GGGGVGKSAL TIQLIQSHFV DEYDPTIEDS YRKQCTIDDQ QVLLDVLDTA
CM029688.1            MLREYKLVVV GGGGVGKSAL TIQLIQSHFV DEYDPTIEDS YRKQCTIDDQ QVLLDVLDTA
CM016755.1            C*ENIN*LLL GGGGVGKSAL TIHLIQSHLV DEYDPTIEDS YRKQCTIDDQ QVLLDVLDTA
JAMFPW010000016.1     MLREYKLVVV GGGGVGKSAL TIQLIQSHFV DEYDPTIEDS YRKQCTIDDQ QVLLDVLDTA
VBVA01000076.1        MLREYKLVVV GGGGVGKSAL TIQLIQSHFV DEYDPTIEIL IVNNVLLMIN KYYWMF*ILL
KE651046.1            MLREYKLVVV GGGGVGKSAL TIQLIQSHFV DEYDPTIEDS YRKQCTIDDQ QVLLDVLDTA
JAQJVX010000229.1     MLREYKLVVV GGGGVGKSAL TIQLIQSHFV DEYDPTIEDS YRKQCTIDDQ QVLLDVLDTA
MUZS01002202.1        MLREYKLVVV GGGGVGKSAL TIQLIQSHFV DEYDPTIEDS YRKQCTIDDQ QVLLDVLDTA
AJJB01000009.1        MLREYKLVVV GGGGVGKSAL TIQLIQSHFV DEYDPTIEDS YRKQCTIDDQ QVLLDVLDTA
JAMFPU010000016.1     MLREYKLVVV GGGGVGKSAL TIQLIQSHFV DEYDPTIEDS YRKQCTIDDQ QVLLDVLDTA
CP025168.1            MLREYKLVVV GGGGVGKSAL TIQLIQSHFV DEYDPTIEDS YRKQCTIDDQ QVLLDVLDTA
CP025176.1            MLREYKLVVV GGGGVGKSAL TIQLIQSHFV DEYDPTIEDS YRKQCTIDDQ QVLLDVLDTA
AJIR01000008.1        MLREYKLVVV GGGGVGKSAL TIQLIQSHFV DEYDPTIEDS YRKQCTIDDQ QVLLDVLDTA
SCGY01000440.1        MLREYKLVVV GGGGVGKSAL TIQLIQSHFV DEYDPTIEDS YRKQCTIDDQ QVLLDVLDTA
JAHPZV010000627.1     MLREYKLVVV GGGGVGKSAL TIQLIQSHFV DEYDPTIEDS YRKQCTIDDQ QVLLDVLDTA
JAMFPO010000675.1     MLREYKLVVV GGGGVGKSAL TIQLIQSHFV DEYDPTIEDS YRKQCTIDDQ QVLLDVLDTA
JADLIL010000679.1     MLREYKLVVV GGGGVGKSAL TIQLIQSHFV DEYDPTIEDS YRKQCTIDDQ QVLLDVLDTA
JAHPZU010000362.1     MLREYKLVVV GGGGVGKSAL TIQLIQSHFV DEYDPTIEDS YRKQCTIDDQ QVLLDVLDTA
JADLIS010000901.1     MLREYKLVVV GGGGVGKSAL TIQLIQSHFV DEYDPTIEDS YRKQCTIDDQ QVLLDVLDTA
JAMFPP010000106.1     MLREYKLVVV GGGGVGKSAL TIQLIQSHFV DEYDPTIEDS YRKQCTIDDQ QVLLDVLDTA
JAQJVO010000188.1     MLREYKLVVV GGGGVGKSAL TIQLIQSHFV DEYDPTIEDS YRKQCTIDDQ QVLLDVLDTA
JAMACY010000037.1     MLREYKLVVV GGGGVGKSAL TIQLIQSHFV DEYDPTIEDS YRKQCTIDDQ QVLLDVLDTA
VBUW01000021.1        MLREYKLVVV GGGGVGKSAL TIQLIQSHFV DEYDPTIEDS YRKQCTIDDQ QVLLDVLDTA
JAMFPR010000117.1     MLREYKLVVV GGGGVGKSAL TIQLIQSHFV DEYDPTIEDS YRKQCTIDDQ QVLLDVLDTA
```

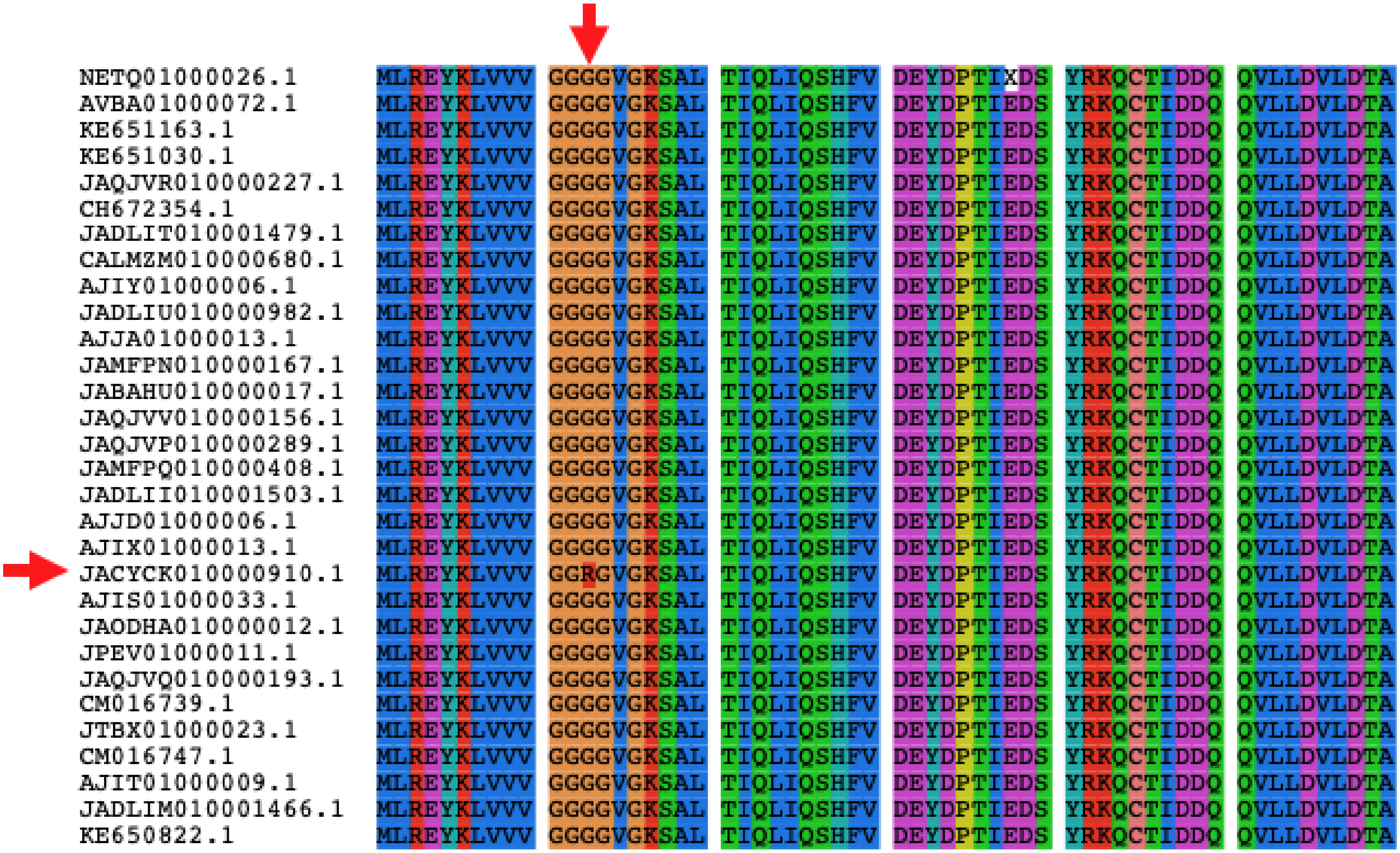
NETQ01000026.1 MLREYKLVVV GGGGVGKSAL TIQLIQSHFV DEYDPTIXDS YRKQCTIDDQ QVLLDVLDTA
AVBA01000072.1 MLREYKLVVV GGGGVGKSAL TIQLIQSHFV DEYDPTIEDS YRKQCTIDDQ QVLLDVLDTA
KE651163.1 MLREYKLVVV GGGGVGKSAL TIQLIQSHFV DEYDPTIEDS YRKQCTIDDQ QVLLDVLDTA
KE651030.1 MLREYKLVVV GGGGVGKSAL TIQLIQSHFV DEYDPTIEDS YRKQCTIDDQ QVLLDVLDTA
JAQJVR010000227.1 MLREYKLVVV GGGGVGKSAL TIQLIQSHFV DEYDPTIEDS YRKQCTIDDQ QVLLDVLDTA
CH672354.1 MLREYKLVVV GGGGVGKSAL TIQLIQSHFV DEYDPTIEDS YRKQCTIDDQ QVLLDVLDTA
JADLIT010001479.1 MLREYKLVVV GGGGVGKSAL TIQLIQSHFV DEYDPTIEDS YRKQCTIDDQ QVLLDVLDTA
CALMZM010000680.1 MLREYKLVVV GGGGVGKSAL TIQLIQSHFV DEYDPTIEDS YRKQCTIDDQ QVLLDVLDTA
AJIY01000006.1 MLREYKLVVV GGGGVGKSAL TIQLIQSHFV DEYDPTIEDS YRKQCTIDDQ QVLLDVLDTA
JADLIU010000982.1 MLREYKLVVV GGGGVGKSAL TIQLIQSHFV DEYDPTIEDS YRKQCTIDDQ QVLLDVLDTA
AJJA01000013.1 MLREYKLVVV GGGGVGKSAL TIQLIQSHFV DEYDPTIEDS YRKQCTIDDQ QVLLDVLDTA
JAMFPN010000167.1 MLREYKLVVV GGGGVGKSAL TIQLIQSHFV DEYDPTIEDS YRKQCTIDDQ QVLLDVLDTA
JABAHU010000017.1 MLREYKLVVV GGGGVGKSAL TIQLIQSHFV DEYDPTIEDS YRKQCTIDDQ QVLLDVLDTA
JAQJVV010000156.1 MLREYKLVVV GGGGVGKSAL TIQLIQSHFV DEYDPTIEDS YRKQCTIDDQ QVLLDVLDTA
JAQJVP010000289.1 MLREYKLVVV GGGGVGKSAL TIQLIQSHFV DEYDPTIEDS YRKQCTIDDQ QVLLDVLDTA
JAMFPQ010000408.1 MLREYKLVVV GGGGVGKSAL TIQLIQSHFV DEYDPTIEDS YRKQCTIDDQ QVLLDVLDTA
JADLII010001503.1 MLREYKLVVV GGGGVGKSAL TIQLIQSHFV DEYDPTIEDS YRKQCTIDDQ QVLLDVLDTA
AJJD01000006.1 MLREYKLVVV GGGGVGKSAL TIQLIQSHFV DEYDPTIEDS YRKQCTIDDQ QVLLDVLDTA
AJIX01000013.1 MLREYKLVVV GGGGVGKSAL TIQLIQSHFV DEYDPTIEDS YRKQCTIDDQ QVLLDVLDTA
JACYCK010000910.1 MLREYKLVVV GGRGVGKSAL TIQLIQSHFV DEYDPTIEDS YRKQCTIDDQ QVLLDVLDTA
AJIS01000033.1 MLREYKLVVV GGGGVGKSAL TIQLIQSHFV DEYDPTIEDS YRKQCTIDDQ QVLLDVLDTA
JAODHA010000012.1 MLREYKLVVV GGGGVGKSAL TIQLIQSHFV DEYDPTIEDS YRKQCTIDDQ QVLLDVLDTA
JPEV01000011.1 MLREYKLVVV GGGGVGKSAL TIQLIQSHFV DEYDPTIEDS YRKQCTIDDQ QVLLDVLDTA
JAQJVQ010000193.1 MLREYKLVVV GGGGVGKSAL TIQLIQSHFV DEYDPTIEDS YRKQCTIDDQ QVLLDVLDTA
CM016739.1 MLREYKLVVV GGGGVGKSAL TIQLIQSHFV DEYDPTIEDS YRKQCTIDDQ QVLLDVLDTA
JTBX01000023.1 MLREYKLVVV GGGGVGKSAL TIQLIQSHFV DEYDPTIEDS YRKQCTIDDQ QVLLDVLDTA
CM016747.1 MLREYKLVVV GGGGVGKSAL TIQLIQSHFV DEYDPTIEDS YRKQCTIDDQ QVLLDVLDTA
AJIT01000009.1 MLREYKLVVV GGGGVGKSAL TIQLIQSHFV DEYDPTIEDS YRKQCTIDDQ QVLLDVLDTA
JADLIM010001466.1 MLREYKLVVV GGGGVGKSAL TIQLIQSHFV DEYDPTIEDS YRKQCTIDDQ QVLLDVLDTA
KE650822.1 MLREYKLVVV GGGGVGKSAL TIQLIQSHFV DEYDPTIEDS YRKQCTIDDQ QVLLDVLDTA

```
                         61
KE651087.1               GQEEYLAMRE QYMRTGEGFL LVYSINSLNS FQELNSFYDQ ILRVKDSDNV PVLVVGNKCD
AJJE01000008.1           GQEEYLAMRE QYMRTGEGFL LVYSINSLNS FQELNSFYDQ ILRVKDSDNV PVLVVGNKCD
AJJG01000018.1           GQEEYLAMRE QYMRTGEGFL LVYSINSLNS FQELNSFYDQ ILRVKDSDNV PVLVVGNKCD
KE650847.1               GQEEYLAMRE QYMRTGEGFL LVYSINSLNS FQELNSFYDQ ILRVKDSDNV PVLVVGNKCD
JAQJVS010000214.1        GQEEYLAMRE QYMRTGEGFL LVYSINSLNS FQELNSFYDQ ILRVKDSDNV PVLVVGNKCD
JAMFPT010000137.1        GQEEYLAMRE QYMRTGEGFL LVYSINSLNS FQELNSFYDQ ILRVKDSDNV PVLVVGNKCD
JSXQ01000010.1           GQEEYLAMRE QYMRTGEGFL LVYSINSLNS FQELNSFYDQ ILRVKDSDNV PVLVVGNKCD
JAMFPV010000028.1        GQEEYLAMRE QYMRTGEGFL LVYSINSLNS FQELNSFYDQ ILRVKDSDNV PVLVVGNKCD
AJJC01000012.1           GQEEYLAMRE QYMRTGEGFL LVYSINSLNS FQELNSFYDQ ILRVKDSDNV PVLVVGNKCD
JADLIQ010000221.1        GQEEYLAMRE QYMRTGEGFL LVYSINSLNS FQELNSFYDQ ILRVKDSDNV PVLVVGNKCD
NETO01000041.1           GQEEYLAMRE QYMRTGEGFL LVYSINSLNS FQELNSFYDQ ILRVKDSDNV PVLVVGNKCD
KE650966.1               GQEEYLAMRE QYMRTGEGFL LVYSINSLNS FQELNSFYDQ ILRVKDSDNV PVLVVGNKCD
KK667224.1               GQEEYLAMRE QYMRTGEGFL LVYSINSLNS FQELNSFYDQ ILRVKDSDNV PVLVVGNKCD
JAQJVU010000255.1        GQEEYLAMRE QYMRTGEGFL LVYSINSLNS FQELNSFYDQ ILRVKDSDNV PVLVVGNKCD
NETM01000037.1           GQEEYLAMRE QYMRTGEGFL LVYSINSLNS FQELNSFYDQ ILRVKDSDNV PVLVVGNKCD
JADLIO010001687.1        GQEEYLAMRE QYMRTGEGFL LVYSINSLNS FQELNSFYDQ ILRVKDSDNV PVLVVGNKCD
JAQJVN010000263.1        GQEEYLAMRE QYMRTGEGFL LVYSINSLNS FQELNSFYDQ ILRVKDSDNV PVLVVGNKCD
JSXP01000007.1           GQEEYLAMRE QYMRTGEGFL LVYSINSLNS FQELNSFYDQ ILRVKDSDNV PVLVVGNKCD
JADLIJ010001632.1        GQEEYLAMRE QYMRTGEGFL LVYSINSLNS FQELNSFYDQ ILRVKDSDNV PVLVVGNKCD
NETN01000103.1           GQEEYLAMRE QYMRTGEGFL LVYSINSLNS FQELNSFYDQ ILRVKDSDNV PVLVVGNKCD
JABWAD010000060.1        DKKNIWP*EN NI*EPVKGFY *FILLIH*IL SKN*IHFMIK FYESKILIMF QF*LLVINVI
AJIZ01000014.1           GQEEYLAMRE QYMRTGEGFL LVYSINSLNS FQELNSFYDQ ILRVKDSDNV PVLVVGNKCD
VBUY01000053.1           GQEEYLAMRE QYMRTGEGFL LVYSINSLNS FQELNSFYDQ ILRVKDSDNV PVLVVGNKCD
KE650918.1               GQEEYLAMRE QYMRTGEGFL LVYSINSLNS FQELNSFYDQ ILRVKDSDNV PVLVVGNKCD
CP032013.1               GQEEYLAMRE QYMRTGEGFL LVYSINSLNS FQELNSFYDQ ILRVKDSDNV PVLVVGNKCD
JADLIN010001249.1        GQEEYLAMRE QYMRTGEGFL LVYSINSLNS FQELNSFYDQ ILRVKDSDNV PVLVVGNKCD
JSXR01000016.1           GQEEYLAMRE QYMRTGEGFL LVYSINSLNS FQELNSFYDQ ILRVKDSDNV PVLVVGNKCD
KE650780.1               GQEEYLAMRE QYMRTGEGFL LVYSINSLNS FQELNSFYDQ ILRVKDSDNV PVLVVGNKCD
AJJF01000006.1           GQEEYLAMRE QYMRTGEGFL LVYSINSLNS FQELNSFYDQ ILRVKDSDNV PVLVVGNKCD
JACXXM010000041.1        GQEEYLAMRE QYMRTGEGFL LVYSINSLNS FQELNSFYDQ ILRVKDSDNV PVLVVGNKCD
CP017624.1               GQEEYLAMRE QYMRTGEGFL LVYSINSLNS FQELNSFYDQ ILRVKDSDNV PVLVVGNKCD
JAMFPS010000366.1        GQEEYLAMRE QYMRTGEGFL LVYSINSLNS FQELNSFYDQ ILRVKDSDNV PVLVVGNKCD
JAMADA010000074.1        GQEEYLAMRE QYMRTGEGFL LVYSINSLNS FQELNSFYDQ ILRVKDSDNV PVLVVGNKCD
JADLIR010000106.1        GQEEYLAMRE QYMRTGEGFL LVYSINSLNS FQELNSFYDQ ILRVKDSDNV PVLVVGNKCD
CP025151.1               GQEEYLAMRE QYMRTGEGFL LVYSINSLNS FQELNSFYDQ ILRVKDSDNV PVLVVGNKCD
CP025159.1               GQEEYLAMRE QYMRTGEGFL LVYSINSLNS FQELNSFYDQ ILRVKDSDNV PVLVVGNKCD
AJIU01000008.1           GQEEYLAMRE QYMRTGEGFL LVYSINSLNS FQELNSFYDQ ILRVKDSDNV PVLVVGNKCD
AJIW01000013.1           GQEEYLAMRE QYMRTGEGFL LVYSINSLNS FQELNSFYDQ ILRVKDSDNV PVLVVGNKCD
JAMACZ010000790.1        GQEEYLAMRE QYMRTGEGFL LVYSINSLNS FQELNSFYDQ ILRVKDSDNV PVLVVGNKCD
KE650911.1               GQEEYLAMRE QYMRTGEGFL LVYSINSLNS FQELNSFYDQ ILRVKDSDNV PVLVVGNKCD
JAKTXK010000203.1        GQEEYLAMRE QYMRTGEGFL LVYSINSLNS FQELNSFYDQ ILRVKDSDNV PVLVVGNKCD
JAQJVY010000211.1        GQEEYLAMRE QYMRTGEGFL LVYSINSLNS FQELNSFYDQ ILRVKDSDNV PVLVVGNKCD
JADLIK010000138.1        GQEEYLAMRE QYMRTGEGFL LVYSINSLNS FQELNSFYDQ ILRVKDSDNV PVLVVGNKCD
JAHPZT010000270.1        GQEEYLAMRE QYMRTGEGFL LVYSINSLNS FQELNSFYDQ ILRVKDSDNV PVLVVGNKCD
AJIQ01000011.1           GQEEYLAMRE QYMRTGEGFL LVYSINSLNS FQELNSFYDQ ILRVKDSDNV PVLVVGNKCD
JADLIP010000819.1        GQEEYLAMRE QYMRTGEGFL LVYSINSLNS FQELNSFYDQ ILRVKDSDNV PVLVVGNKCD
JAQJVT010000197.1        GQEKYLAMRE QYMRTGEGFL LVYSINSLNS FQELNSFYDQ ILRVKDSDNV PVLVVGNKCD
AJIV01000014.1           GQEEYLAMRE QYMRTGEGFL LVYSINSLNS FQELNSFYDQ ILRVKDSDNV PVLVVGNKCD
JAODHB010000006.1        GQEEYLAMRE QYMRTGEGFL LVYSINSLNS FQELNSFYDQ ILRVKDSDNV PVLVVGNKCD
JAAGWN010000001.1        GQEEYLAMRE QYMRTGEGFL LVYSINSLNS FQELNSFYDQ ILRVKDSDNV PVLVVGNKCD
JAQJVW010000188.1        GQEEYLAMRE QYMRTGEGFL LVYSINSLNS FQELNSFYDQ ILRVKDSDNV PVLVVGNKCD
JPEW01000016.1           GQEEYLAMRE QYMRTGEGFL LVYSINSLNS FQELNSFYDQ ILRVKDSDNV PVLVVGNKCD
CM029688.1               GQEEYLAMRE QYMRTGEGFL LVYSINSLNS FQELNSFYDQ ILRVKDSDNV PVLVVGNKCD
CM016755.1               GQEEYLAMRE QYMRTGEGFL LVYSINSLNS FQELNSFYDQ ILRVKDSDNV PSFSCW**M*
JAMFPW010000016.1        GQEEYLAMRE QYMRTGEGFL LVYSINSLNS FQELNSFYDQ ILRVKDSDNV PVLVVGNKCD
VBVA01000076.1           DKKNIWP*EN NI*ER*RVFI SLFY*FIKFF PRIKFIL*SN FTSQRF**CS SFSCW**M*F
KE651046.1               GQEEYLAMRE QYMRTGEGFL LVYSINSLNS FQELNSFYDQ ILRVKDSDNV PVLVVGNKCD
JAQJVX010000229.1        GQEEYLAMRE QYMRTGEGFL LVYSINSLNS FQELNSFYDQ ILRVKDSDNV PVLVVGNKCD
MUZS01002202.1           GQEEYLAMRE QYMRTGEGFL LVYSINSLNS FQELNSFYDQ ILRVKDSDNV PVLVVGNKCD
AJJB01000009.1           GQEEYLAMRE QYMRTGEGFL LVYSINSLNS FQELNSFYDQ ILRVKDSDNV PVLVVGNKCD
JAMFPU010000016.1        GQEEYLAMRE QYMRTGEGFL LVYSINSLNS FQELNSFYDQ ILRVKDSDNV PVLVVGNKCD
CP025168.1               GQEEYLAMRE QYMRTGEGFL LVYSINSLNS FQELNSFYDQ ILRVKDSDNV PVLVVGNKCD
CP025176.1               GQEEYLAMRE QYMRTGEGFL LVYSINSLNS FQELNSFYDQ ILRVKDSDNV QF*LLVINVI
AJIR01000008.1           GQEEYLAMRE QYMRTGEGFL LVYSINSLNS FQELNSFYDQ ILRVKDSDNV PVLVVGNKCD
SCGY01000440.1           GQEEYLAMRE QYMRTGEGFL LVYSINSLNS FQELNSFYDQ ILRVKDSDNV PVLVVGNKCD
JAHPZV010000627.1        GQEEYLAMRE QYMRTGEGFL LVYSINSLNS FQELNSFYDQ ILRVKDSDNV PVLVVGNKCD
JAMFPO010000675.1        GQEEYLAMRE QYMRTGEGFL LVYSINSLNS FQELNSFYDQ
JADLIL010000679.1        GQEEYLAMRE QYMRTGEGFL LVYSINSLNS FQELNSFYDQ ILRVKDSDNV PVLVVGNKCD
JAHPZU010000362.1        GQEEYLAMRE QYMRTGEGFL LVYSINSLNS FQELNSFYDQ ILRVKDSDNV PVLVVGNKCD
JADLIS010000901.1        GQEEYLAMRE QYMRTGEGFL LVYSINSLNS FQELNSFYDQ ILRVKDSDNV PVLVVGNKCD
JAMFPP010000106.1        GQEEYLAMRE QYMRTGEGFL LVYSINSLNS FQELNSFYDQ ILRVKDSDNV PVLVVGNKCD
JAQJVO010000188.1        GQEEYLAMRE QYMRTGEGFL LVYSINSLNS FQELNSFYDQ ILRVKDSDNV PVLVVGNKCD
JAMACY010000037.1        GQEEYLAMRE QYMRTGEGFL LVYSINSLNS FQELNSFYDQ ILRVKDSDNV PVLVVGNKCD
VBUW01000021.1           GQEEYLAMRE QYMRTGEGFL LVYSINSLNS FQELNSFYDQ ILRVKDSDNV PVLVVGNKCD
JAMFPR010000117.1        GQEEYLAMRE QYMRTGEGFL LVYSINSLNS FQELNSFYDQ ILRVKDSDNV PVLVVGNKCD
NETQ01000026.1           GQEEYLAMRE QYMRXGEGFL LVYSINSLNS FQELNSFYDQ ILRVKDSDNV PVLVVGNKCD
AVBA01000072.1           GQEEYLAMRE QYMRTGEGFL LVYSINSLNS FQELNSFYDQ ILRVKDSDNV PVLVVGNKCD
KE651163.1               GQEEYLAMRE QYMRTGEGFL LVYSINSLNS FQELNSFYDQ ILRVKDSDNV PVLVVGNKCD
```

KE651030.1	GQEEYLAMRE	QYMRTGEGFL	LVYSINSLNS	FQELNSFYDQ	ILRVKDSDNV	PVLVVGNKCD
JAQJVR010000227.1	GQEEYLAMRE	QYMRTGEGFL	LVYSINSLNS	FQELNSFYDQ	ILRVKDSDNV	PVLVVGNKCD
CH672354.1	GQEEYLAMRE	QYMRTGEGFL	LVYSINSLNS	FQELNSFYDQ	ILRVKDSDNV	PVLVVGNKCD
JADLIT010001479.1	GQEEYLAMRE	QYMRTGEGFL	LVYSINSLNS	FQELNSFYDQ	ILRVKDSDNV	PVLVVGNKCD
CALMZM010000680.1	GQEEYLAMRE	QYMRTGEGFL	LVYSINSLNS	FQELNSFYDQ	ILRVKDSDNV	PVLVVGNKCD
AJIY01000006.1	GQEEYLAMRE	QYMRTGEGFL	LVYSINSLNS	FQELNSFYDQ	ILRVKDSDNV	PVLVVGNKCD
JADLIU010000982.1	GQEEYLAMRE	QYMRTGEGFL	LVYSINSLNS	FQELNSFYDQ	ILRVKDSDNV	PVLVVGNKCD
AJJA01000013.1	GQEEYLAMRE	QYMRTGEGFL	LVYSINSLNS	FQELNSFYDQ	ILRVKDSDNV	PVLVVGNKCD
JAMFPN010000167.1	GQEEYLAMRE	QYMRTGEGFL	LVYSINSLNS	FQELNSFYDQ	ILRVKDSDNV	PVLVVGNKCD
JABAHU010000017.1	GQEEYLAMRE	QYMRTGEGFF	LLVYSINSLN	SFQELNSFYD	QILRVKDSDN	VPVLVVGNKC
JAQJVV010000156.1	GQEEYLAMRE	QYMRTGEGFL	LVYSINSLNS	FQELNSFYDQ	ILRVKDSDNV	PVLVVGNKCD
JAQJVP010000289.1	GQEEYLAMRE	QYMRTGEGFL	LVYSINSLNS	FQELNSFYDQ	ILRVKDSDNV	PVLVVGNKCD
JAMFPQ010000408.1	GQEEYLAMRE	QYMRTGEGFL	LVYSINSLNS	FQELNSFYDQ	ILRVKDSDNV	PVLVVGNKCD
JADLII010001503.1	GQEEYLAMRE	QYMRTGEGFL	LVYSINSLNS	FQELNSFYDQ	ILRVKDSDNV	PVLVVGNKCD
AJJD01000006.1	GQEEYLAMRE	QYMRTGEGFL	LVYSINSLNS	FQELNSFYDQ	ILRVKDSDNV	PVLVVGNKCD
AJIX01000013.1	GQEEYLAMRE	QYMRTGEGFL	LVYSINSLNS	FQELNSFYDQ	ILRVKDSDNV	PVLVVGNKCD
JACYCK010000910.1	GQEEYLAMRE	QYMRTGEGFL	LVYSINSLNS	FQELNSFYDQ	ILRVKDSDNV	PVLVVGNKCD
AJIS01000033.1	GQEEYLAMRE	QYMRTGEGFL	LVYSINSLNS	FQELNSFYDQ	ILRVKDSDNV	PVLVVGNKCD
JAODHA010000012.1	GQEEYLAMRE	QYMRTGEGFL	LVYSINSLNS	FQELNSFYDQ	ILRVKDSDNV	PVLVVGNKCD
JPEV01000011.1	GQEEYLAMRE	QYMRTGEGFL	LVYSINSLNS	FQELNSFYDQ	ILRVKDSDNV	PVLVVGNKCD
JAQJVQ010000193.1	GQEEYLAMRE	QYMRTGEGFL	LVYSINSLNS	FQELNSFYDQ	ILRVKDSDNV	PVLVVGNKCD
CM016739.1	GQEEYLAMRE	QYMRTGEGFL	LVYSINSLNS	FQELNSFYDQ	ILRVKDSDNV	PVLVVGNKCD
JTBX01000023.1	GQEEYLAMRE	QYMRTGEGFL	LVYSINSLNS	FQELNSFYDQ	ILRVKDSDNV	PVLVVGNKCD
CM016747.1	GQEEYLAMRE	QYMRTGEGFL	LVYSINSLNS	FQELNSFYDQ	ILRVKDSDNV	PVLVVGNKCD
AJIT01000009.1	GQEEYLAMRE	QYMRTGEGFL	LVYSINSLNS	FQELNSFYDQ	ILRVKDSDNV	PVLVVGNKCD
JADLIM010001466.1	GQEEYLAMRE	QYMRTGEGFL	LVYSINSLNS	FQELNSFYDQ	ILRVKDSDNV	PVLVVGNKCD
KE650822.1	GQEEYLAMRE	QYMRTGEGFL	LVYSINSLNS	FQELNSFYDQ	ILRVKDSDNV	PVLVVGNKCD

```
                         121
KE651087.1               LEMERQVSYE DGLALANSFN CPFLETSAKQ RINVEEAFYG LVRNINQYNA KIAEAEKQQQ
AJJE01000008.1           LEMERQVSYE DGLALANSFN CPFLETSAKQ RINVEEAFYG LVRNINQYNA KIAEAEKQQQ
AJJG01000018.1           LEMERQVSYE DGLALANSFN CPFLETSAKQ RINVEEAFYG LVRNINQYNA KIAEAEKQQQ
KE650847.1               LEMERQVSYE DGLALANSFN CPFLETSAKQ RINVEEAFYG LVRNINQYNA KIAEAEKQQQ
JAQJVS010000214.1        LEMERQVSYE DGLALANSFN CPFLETSAKQ RINVEEAFYG LVRNINQYNA KIAEAEKQQQ
JAMFPT010000137.1        LEMERQVSYE DGLALANSFN CPFLETSAKQ RINVEEAFYG LVRNINQYNA KIAEAEKQQQ
JSXQ01000010.1           LEMERQVSYE DGLALANSFN CPFLETSAKQ RINVEEAFYG LVRNINQYNA KIAEAEKQQQ
JAMFPV010000028.1        LEMERQVSYE DGLALANSFN CPFLETSAKQ RINVEEAFYG LVRNINQYNA KIAEAEKQQQ
AJJC01000012.1           LEMERQVSYE DGLALANSFN CPFLETSAKQ RINVEEAFYG LVRNINQYNA KIAEAEKQQQ
JADLIQ010000221.1        LEMERQVSYE DGLALANSFN CPFLETSAKQ RINVEEAFYG LVRNINQYNA KIAEAEKQQQ
NETO01000041.1           LEMERQVSYE DGLALANSFN CPFLETSAKQ RINVEEAFYG LVRNINQYNA KIAEAEKQQQ
KE650966.1               LEMERQVSYE DGLALANSFN CPFLETSAKQ RINVEEAFYG LVRNINQYNA KIAEAEKQQQ
KK667224.1               LEMERQVSYE DGLALANSFN CPFLETSAKQ RINVEEAFYG LVRNINQYNA KIAEAEKQQQ
JAQJVU010000255.1        LEMERQVSYE DGLALANSFN CPFLETSAKQ RINVEEAFYG LVRNINQYNA KIAEAEKQQQ
NETM01000037.1           LEMERQVSYE DGLALANSFN CPFLETSAKQ RINVEEAFYG LVRNINQYNA KIAEAEKQQQ
JADLIO010001687.1        LEMERQVSYE DGLALANSFN CPFLETSAKQ RINVEEAFYG LVRNINQYNA KIAEAEKQQQ
JAQJVN010000263.1        LEMERQVSYE DGLALANSFN CPFLETSAKQ RINVEEAFYG LVRNINQYNA KIAEAEKQQQ
JSXP01000007.1           LEMERQVSYE DGLALANSFN CPFLETSAKQ RINVEEAFYG LVRNINQYNA KIAEAEKQQQ
JADLIJ010001632.1        LEMERQVSYQ DGLALANSFN CPFLETSAKQ RINVEEAFYG LVRNINQYNA KIAEAEKQQQ
NETN01000103.1           LEMERQVSYE DGLALANSFN CPFLETSAKQ RINVEEAFYG LVRNINQYNA KIAEAEKQQQ
JABWAD010000060.1        *KWKDKLVMK MD*HWLILSI VHF*KLLLNK ELMLKKHFMD **EILINIML KLLKLKNNNN
AJIZ01000014.1           LEMERQVSYE DGLALANSFN CPFLETSAKQ RINVEEAFYG LVRNINQYNA KIAEAEKQQQ
VBUY01000053.1           LEMERQVSYE DGLALANSFN CPFLETSAKQ RINVEEAFYG LVRNINQYNA KIAEAEKQQQ
KE650918.1               LEMERQVSYE DGLALANSFN CPFLETSAKQ RINVEEAFYG LVRNINQYNA KIAEAEKQQQ
CP032013.1               LEMERQVSYE DGLALANSFN CPFLETSAKQ RINVEEAFYG LVRNINQYNA KIAEAEKQQQ
JADLIN010001249.1        LEMERQVSYE DGLALANSFN CPFLETSAKQ RINVEEAFYG LVRNINQYNA KIAEAEKQQQ
JSXR01000016.1           LEMERQVSYE DGLALANSFN CPFLETSAKQ RINVEEAFYG LVRNINQYNA KIAEAEKQQQ
KE650780.1               LEMERQVSYE DGLALANSFN CPFLETSAKQ RINVEEAFYG LVRNINQYNA KIAEAEKQQQ
AJJF01000006.1           LEMERQVSYE DGLALANSFN CPFLETSAKQ RINVEEAFYG LVRNINQYNA KIAEAEKQQQ
JACXXM010000041.1        LEMERQVSYE DGLALANSFN CPFLETSAKQ RINVEEAFYG LVRNINQYNA KIAEAEKQQQ
CP017624.1               LEMERQVSYE DGLALANSFN CPFLETSAKQ RINVEEAFYG LVRNINQYNA KIAEAEKQQQ
JAMFPS010000366.1        LEMERQVSYE DGLALANSFN CPFLETSAKQ RINVEEAFYG LVRNINQYNA KIAEAEKQQQ
JAMADA010000074.1        LEMERQVSYE DGLALANSFN CPFLETSAKQ RINVEEAFYG LVRNINQYNA KIAEAEKQQQ
JADLIR010000106.1        LEMERQVSYE DGLALANSFN CPFLETSAKQ RINVEEAFYG LVRNINQYNA KIAEAEKQQQ
CP025151.1               LEMERQVSYE DGLALANSFN CPFLETSAKQ RINVEEAFYG LVRNINQYNA KIAEAEKQQQ
CP025159.1               LEMERQVSYQ DGLALANSFN CPFLETSAKQ RINVEEAFYG LVRNINQYNA KIAEAEKQQQ
AJIU01000008.1           LEMERQVSYE DGLALANSFN CPFLETSAKQ RINVEEAFYG LVRNINQYNA KIAEAEKQQQ
AJIW01000013.1           LEMERQVSYE DGLALANSFN CPFLETSAKQ RINVEEAFYG LVRNINQYNA KIAEAEKQQQ
JAMACZ010000790.1        LEMERQVSYE DGLALANSFN CPFLETSAKQ RINVEEAFYG LVRNINQYNA KIAEAEKQQQ
KE650911.1               LEMERQVSYE DGLALANSFN CPFLETSAKQ RINVEEAFYG LVRNINQYNA KIAEAEKQQQ
JAKTXK010000203.1        LEMERQVSYE DGLALANSFN CPFLETSAKQ RINVEEAFYG LVRNINQYNA KIAESEKQQQ
JAQJVY010000211.1        LEMERQVSYE DGLALANSFN CPFLETSAKQ RINVEEAFYG LVRNINQYNA KIAEAEKQQQ
JADLIK010000138.1        LEMERQVSYE DGLALANSFN CPFLETSAKQ RINVEEAFYG LVRNINQYNA KIAEAEKQQQ
JAHPZT010000270.1        LEMERQVSYE DGLALANSFN CPFLETSAKQ RINVEEAFYG LVRNINQYNA KIAEAEKQQQ
AJIQ01000011.1           LEMERQVSYE DGLALANSFN CPFLETSAKQ RINVEEAFYG LVRNINQYNA KIAEAEKQQQ
JADLIP010000819.1        LEMERQVSYE DGLALANSFN CPFLETSAKQ RINVEEAFYG LVRNINQYNA KIAEAEKQQQ
JAQJVT010000197.1        LEMERQVSYE DGLALANSFN CPFLETSAKQ RINVEEAFYG LVRNINQYNA KIAEAEKQQQ
AJIV01000014.1           LEMERQVSYE DGLALANSFN CPFLETSAKQ RINVEEAFYG LVRNINQYNA KIAEAEKQQQ
JAODHB010000006.1        LEMERQVSYQ DGLALANSFN CPFLETSAKQ RINVEEAFYG LVRNINQYNA KIAEAEKQQQ
JAAGWN010000001.1        LEMERQVSYQ DGLALANSFN CPFLETSAKQ RINVEEAFYG LVRNINQYNA KIAEAEKQQQ
JAQJVW010000188.1        LEMERQVSYE DGLALANSFN CPFLETSAKQ RINVEEAFYG LVRNINQYNA KIAEAEKQQQ
JPEW01000016.1           LEMERQVSYE DGLALANSFN CPFLETSAKQ RINVEEAFYG LVRNINQYNA KIAEAEKQQQ
CM029688.1               LEMERQVSYE DGLALANSFN CPFLETSAKQ RINVEEAFYG LVRNINQYNA KIAEAEKQQQ
CM016755.1               FRNGKTS*L* RWISIG*FFQ LSIFRNFC*T KN*C*RSILW ISKKY*SI*C *NC*S*KNNN
JAMFPW010000016.1        LEMERQVSYE DGLALANSFN CPFLETSAKQ RINVEEAFYG LVRNINQYNA KIAEAEKQQQ
VBVA01000076.1           RNGKTS*L*R WISIG*FFQL SIFRNFC*TK N*C*RSILWI SKKY*SI*C* NC*S*KTTTT
KE651046.1               LEMERQVSYE DGLALANSFN CPFLETSAKQ RINVEEAFYG LVRNINQYNA KIAEAEKQQQ
JAQJVX010000229.1        LEMERQVSYE DGLALANSFN CPFLETSAKQ RINVEEAFYG LVRNINQYNA KIAEAEKQQQ
MUZS01002202.1           LEMERQVSYE DGLALANSFN CPFLETSAKQ RINVEEAFYG LVRNINQYNA KIAEAEKQQQ
AJJB01000009.1           LEMERQVSYE DGLALANSFN CPFLETSAKQ RINVEEAFYG LVRNINQYNA KIAEAEKQQQ
JAMFPU010000016.1        LEMERQVSYE DGLALANSFN CPFLETSAKQ RINVEEAFYG LVRNINQYNA KIAEAEKQQQ
CP025168.1               LEMERQVSYE DGLALANSFN CPFLETSAKQ RINVEEAFYG LVRNINQYNA KIAEAEKQQQ
CP025176.1               *KWKDKLVIK MD*HWLILSI VHF*KLLLNK ELMLKKHFMD **EILINIML KLLKLKNNNN
AJIR01000008.1           LEMERQVSYE DGLALANSFN CPFLETSAKQ RINVEEAFYG LVRNINQYNA KIAEAEKQQQ
SCGY01000440.1           LEMERQVSYE DGLALANSFN CPFLETSAKQ RINVEEAFYG LVRNINQYNA KIAEAEKQQQ
JAHPZV010000627.1        LEMERQVSYE DGLALANSFN CPFLETSAKQ RINVEEAFYG LVRNINQYNA KIAEAEKQQQ
JAMFPO010000675.1
JADLIL010000679.1        LEMERQVSYE DGLALANSFN CPFLETSAKQ RINVEEAFYG LVRNINQYNA KIAEAEKQQQ
JAHPZU010000362.1        LEMERQVSYE DGLALANSFN CPFLETSAKQ RINVEEAFYG LVRNINQYNA KIAEAEKQQQ
JADLIS010000901.1        LEMERQVSYE DGLALANSFN CPFLETSAKQ RINVEEAFYG LVRNINQYNA KIAEAEKQQQ
JAMFPP010000106.1        LEMERQVSYE DGLALANSFN CPFLETSAKQ RINVEEAFYG LVRNINQYNA KIAEAEKQQQ
JAQJVO010000188.1        LEMERQVSYE DGLALANSFN CPFLETSAKQ RINVEEAFYG LVRNINQYNA KIAEAEKQQQ
JAMACY010000037.1        LEMERQVSYE DGLALANSFN CPFLETSAKQ RINVEEAFYG LVRNINQYNA KIAEAEKQQQ
VBUW01000021.1           LEMERQVSYE DGLALANSFN CPFLETSAKQ RINVEEAFYG LVRNINQYNA KIAEAEKQQQ
JAMFPR010000117.1        LEMERQVSYE DGLALANSFN CPFLETSAKQ RINVEEAFYG LVRNINQYNA KIAEAEKQQQ
NETQ01000026.1           LEMERQVSYE DGLALANSFN CPFLETSAKQ RINVEEAFYG LVRNINQYNA KIAEAEKQQQ
AVBA01000072.1           LEMERQVSYE DGLALANSFN CPFLETSAKQ RINVEEAFYG LVRNINQYNA KIAEAEKQQQ
KE651163.1               LEMERQVSYE DGLALANSFN CPFLETSAKQ RINVEEAFYG LVRNINQYNA KIAEAEKQQQ
```

```
KE651030.1            LEMERQVSYE DGLALANSFN CPFLETSAKQ RINVEEAFYG LVRNINQYNA KIAEAEKQQQ
JAQJVR010000227.1     LEMERQVSYE DGLALANSFN CPFLETSAKQ RINVEEAFYG LVRNINQYNA KIAEAEKQQQ
CH672354.1            LEMERQVSYE DGLALANSFN CPFLETSAKQ RINVEEAFYG LVRNINQYNA KIAEAEKQQQ
JADLIT010001479.1     LEMERQVSYE DGLALANSFN CPFLETSAKQ RINVEEAFYG LVRNINQYNA KIAEAEKQQQ
CALMZM010000680.1     LEMERQVSYE DGLALANSFN CPFLETSAKQ RINVEEAFYG LVRNINQYNA KIAEAEKQQQ
AJIY01000006.1        LEMERQVSYE DGLALANSFN CPFLETSAKQ RINVEEAFYG LVRNINQYNA KIAEAEKQQQ
JADLIU010000982.1     LEMERQVSYE DGLALANSFN CPFLETSAKQ RINVEEAFYG LVRNINQYNA KIAEAEKQQQ
AJJA01000013.1        LEMERQVSYE DGLALANSFN CPFLETSAKQ RINVEEAFYG LVRNINQYNA KIAEAEKQQQ
JAMFPN010000167.1     LEMERQVSYE DGLALANSFN CPFLETSAKQ RINVEEAFYG LVRNINQYNA KIAEAEKQQQ
JABAHU010000017.1     DLEMERQVSY EDGLALANSF NCPFFLETSA KQRINVEEAF YGLVRNINQY NAKIAEAEKN
JAQJVV010000156.1     LEMERQVSYE DGLALANSFN CPFLETSAKQ RINVEEAFYG LVRNINQYNA KIAEAEKQQQ
JAQJVP010000289.1     LEMERQVSYE DGLALANSFN CPFLETSAKQ RINVEEAFYG LVRNINQYNA KIAEAEKQQQ
JAMFPQ010000408.1     LEMERQVSYE DGLALANSFN CPFLETSAKQ RINVEEAFYG LVRNINQYNA KIAEAEKQQQ
JADLII010001503.1     LEMERQVSYE DGLALANSFN CPFLETSAKQ RINVEEAFYG LVRNINQYNA KIAE
AJJD01000006.1        LEMERQVSYE DGLALANSFN CPFLETSAKQ RINVEEAFYG LVRNINQYNA KIAEAEKQQQ
AJIX01000013.1        LEMERQVSYE DGLALANSFN CPFLETSAKQ RINVEEAFYG LVRNINQYNA KIAEAEKQQQ
JACYCK010000910.1     LEMERQVSYE DGLALANSFN CPFLETSAKQ RINVEEAFYG LVRNINQYNA KIAEAEKQQQ
AJIS01000033.1        LEMERQVSYE DGLALANSFN CPFLETSAKQ RINVEEAFYG LVRNINQYNA KIAEAEKQQQ
JAODHA010000012.1     LEMERQVSYQ DGLALANSFN CPFLETSAKQ RINVEEAFYG LVRNINQYNA KIAEAEKQQQ
JPEV01000011.1        LEMERQVSYE DGLALANSFN CPFLETSAKQ RINVEEAFYG LVRNINQYNA KIAEAEKQQQ
JAQJVQ010000193.1     LEMERQVSYE DGLALANSFN CPFLETSAKQ RINVEEAFYG LVRNINQYNA KIAEAEKQQQ
CM016739.1            LEMERQVSYE DGLALANSFN CPFLETSAKQ RINVEEAFYG LVRNINQYNA KIAEAEKQQQ
JTBX01000023.1        LEMERQVSYE DGLALANSFN CPFLETSAKQ RINVEEAFYG LVRNINQYNA KIAEAEKQQQ
CM016747.1            LEMERQVSYE DGLALANSFN CPFLETSAKQ RINVEEAFYG LVRNINQYNA KIAEAEKQQQ
AJIT01000009.1        LEMERQVSYE DGLALANSFN CPFLETSAKQ RINVEEAFYG LVRNINQYNA KIAEAEKQQQ
JADLIM010001466.1     LEMERQVSYE DGLALANSFN CPFLETSAKQ RINVEEAFYG LVRNINQYNA KIAEAEKQQQ
KE650822.1            LEMERQVSYE DGLALANSFN CPFLETSAKQ RINVEEAFYG LVRNINQYNA KIAEAEKQQQ
```

	181					
KE651087.1	QQQQQQNANQ	QGQDQYGQQK	DNQQSQFNNQ	INNNNNTSAV	NGGVSSDGII	DQNGNGGVSS
AJJE01000008.1	QQQQQQQNAN	QQGQDQYGQQ	KDNQQSQFNN	QINNNNNTSA	VNGGVSSDGI	IDQNGNGGVS
AJJG01000018.1	QQQQQQNANQ	QGQDQYGQQK	DNQQSQFNNQ	INNNNNNTSA	VNGGVSSDGI	IDQNGNGGVS
KE650847.1	QQQQQQNANQ	QGQDQYGQQK	DNQQSQFNNQ	INNNNNTSAV	NGGVSSDGII	DQNGNGGVSS
JAQJVS010000214.1	QQQQQQNANQ	QGQDQYGQQK	DNQQSQFNNQ	INNNNNTSAV	NGGVSSDGII	DQNGNGGVSS
JAMFPT010000137.1	QQQQQQNANQ	QGQDQYGQQK	DNQQSQFNNQ	INNNNNTSAV	NGGVSSDGII	DQNGNGGVSS
JSXQ01000010.1	QQQQQQNANQ	QGQDQYGQQK	DNQQSQFNNQ	INNNNNNTSA	VNGGVSSDGI	IDQNGNGGVS
JAMFPV010000028.1	QQQQQQNANQ	QGQDQYGQQK	DNQQSQFNNQ	INNNNNTSAV	NGGVSSDGII	DQNGNGGVSS
AJJC01000012.1	QQQQQQNANQ	QGQDQYGQQK	DNQQSQFNNQ	INNNNNNTSA	VNGGVSSDGI	IDQNGNGGVS
JADLIQ010000221.1	QQQQQQNANQ	QGQDQYGQQK	DNQQSQFNNQ	INNNNNNTSA	VNGGVSSDGI	IDQNGNGGVS
NETO01000041.1	QQQQQQNANQ	QGQDQYGQQK	DNQQSQFNNQ	INNNNNTSAV	NGGVSSDGII	DQNGNGGVSS
KE650966.1	QQQQQQNANQ	QGQDQYGQQK	DNQQSQFNNQ	INNNNNTSAV	NGGVSSDGII	DQNGNGGVSS
KK667224.1	QQQQQQNANQ	QGQDQYGQQK	DNQQSQFNNQ	INNNNNNTSA	VNGGVSSDGI	IDQNGNGGVS
JAQJVU010000255.1	QQQQQQNANQ	QGQDQYGQQK	DNQQSQFNNQ	INNNNNNTSA	VNGGVSSDGI	IDQNGNGGVS
NETM01000037.1	QQQQQQNANQ	QGQDQYGQQK	DNQQSQFNNQ	INNNNNTSAV	NGGVSSDGII	DQNGNGGVSS
JADLIO010001687.1	QQQQQQNANQ	QGQDQYGQQK	DNQQSQFNNQ	INNNNNTSAV	NGGVSSDGII	DQNGNGGVSS
JAQJVN010000263.1	QQQQQQNANQ	QGQDQYGQQK	DNQQSQFNNQ	INNNNNNTSA	VNGGVSSDGI	IDQNGNGGVS
JSXP01000007.1	QQQQQQNANQ	QGQDQYGQQK	DNQQSQFNNQ	INNNNNTSAV	NGGVSSDGII	DQNGNGGVSS
JADLIJ010001632.1	QQQQQQNANQ	QGQDQYGQQK	DNQQSQFNNQ	INNNNNTSAV	NGGVSSDGII	DQNGNGGVSS
NETN01000103.1	QQQQQQNANQ	QGQDQYGQQK	DNQQSQFNNQ	INNNNNTSAV	NGGVSSDGII	DQNGNGGVSS
JABWAD010000060.1	SNNNNKMQIN	KVKTNMDNKK	IINNHNLIIK	*IIIIIILVL	LMVV*VVME*	*IKMVMEVFL
AJIZ01000014.1	QQQQQQNANQ	QGQDQYGQQK	DNQQSQFNNQ	INNNNNNTSA	VNGGVSSDGI	IDQNGNGSVS
VBUY01000053.1	QQQQQQNANQ	QGQDQYGQQK	DNQQSQFNNQ	INNNNNNTSA	VNGGVSSDGI	IDQNGNGGVS
KE650918.1	QQQQQQNANQ	QGQDQYGQQK	DNQQSQFNNQ	INNNNNNTSA	VNGGVSSDGI	IDQNGNGGVS
CP032013.1	QQQQQQNANQ	QGQDQYGQQK	DNQQSQFNNQ	INNNNNTSAV	NGGVSSDGII	DQNGNGGVSS
JADLIN010001249.1	QQQQQQNANQ	QGQDQYGQQK	DNQQSQFNNQ	INNNNNNTSA	VNGGVSSDGI	IDQNGNGGVS
JSXR01000016.1	QQQQQQNANQ	QGQDQYGQQK	DNQQSQFNNQ	INNNNNNTSA	VNGGVSSDGI	IDQNGNGGVS
KE650780.1	QQQQQQNANQ	QGQDQYGQQK	DNQQSQFNNQ	INNNNNNTSA	VNGGVSSDGI	IDQNGNGGVS
AJJF01000006.1	QQQQQQNANQ	QGQDQYGQQK	DNQQSQFNNQ	INNNNNTSAV	NGGVSSDGII	DQNGNGGVSS
JACXXM010000041.1	QQQQQQNANQ	QGQDQYGQQK	DNQQSQFNNQ	INNNNNNTSA	VNGGVSSDGI	IDQNGNGSVS
CP017624.1	QQQQQQNANQ	QGQDQYGQQK	DNQQSQFNNQ	INNNNNTSAV	NGGVSSDGII	DQNGNGGVSS
JAMFPS010000366.1	QQQQQQNANQ	QGQDQYGQQK	DNQQSQFNNQ	INNNNNNTSA	VNGGVSSDGI	IDQNGNGGVS
JAMADA010000074.1	QQQQQQNANQ	QGQDQYGQQK	DNQQSQFNNQ	INNNNNNTSA	VNGGVSSDGI	IDQNGNGGVS
JADLIR010000106.1	QQQQQQNANQ	QGQDQYGQQK	DNQQSQFNNQ	INNNNNNTSA	VNGGVSSDGI	IDQNGNGGVS
CP025151.1	QQQQQQNANQ	QGQDQYGQQK	DNQQSQFNNQ	INNNNNTSAV	NGGVSSDGII	DQNGNGGVSS
CP025159.1	QQQQQQNANQ	QGQDQYGQQK	DNQQSQFNNQ	INNNNNNTSA	VNGGVSSDGI	IDQNGNGGVS
AJIU01000008.1	QQQQQQNANQ	QGQDQYGQQK	DNQQSQFNNQ	INNNNNNTSA	VNGGVSSDGI	IDQNGNGGVS
AJIW01000013.1	QQQQQQNANQ	QGQDQYGQQK	DNQQSQFNNQ	INNNNNNTSA	VNGGVSSDGI	IDQNGNGGVS
JAMACZ010000790.1	QQQQQQNANQ	QGQDQYGQQK	DNQQSQFNNQ	INNNNNNTSA	VNGGVSSDGI	IDQNGNGSVS
KE650911.1	QQQQQQNANQ	QGQDQYGQQK	DNQQSQFNNQ	INNNNNNTSA	VNGGVSSDGI	IDQNGNGSVS
JAKTXK010000203.1	QNANQQGQDQ	YGQQKDNQQS	QFNNQINNNN	NNTSAVNGGV	SSDGIIDQNG	NGGVSSGQAN
JAQJVY010000211.1	QQQQQQNANQ	QGQDQYGQQK	DNQQSQFNNQ	INNNNNNTSA	VNGGVSSDGI	IDQNGNGGVS
JADLIK010000138.1	QQQQQQNANQ	QGQDQYGQQK	DNQQSQFNNQ	INNNNNNTSA	VNGGVSSDGI	IDQNGNGGVS
JAHPZT010000270.1	QQQQQQNANQ	QGQDQYGQQK	DNQQSQFNNQ	INNNNNTSAV	NGGVSSDGII	DQNGNGGVSS
AJIQ01000011.1	QQQQQQNANQ	QGQDQYGQQK	DNQQSQFNNQ	INNNNNNTSA	VNGGVSSDGI	IDQNGNGGVS
JADLIP010000819.1	QQQQQQNANQ	QGQDQYGQQK	DNQQSQFNNQ	INNNNNNTSA	VNGGVSSDGI	IDQNGNGGVS
JAQJVT010000197.1	QQQQQQNANQ	QGQDQYGQQK	DNQQSQFNNQ	INNNNNTSAV	NGGVSSDGII	DQNGNGGVSS
AJIV01000014.1	QQQQQQNANQ	QGQDQYGQQK	DNQQSQFNNQ	INNNNNTSAV	NGGVSSDGII	DQNGNGGVSS
JAODHB010000006.1	QQQQQQNANQ	QGQDQYGQQK	DNQQSQFNNQ	INNNNNNTSA	VNGGVSSDGI	IDQNGNGGVS
JAAGWN010000001.1	QQQQQQNANQ	QGQDQYGQQK	DNQQSQFNNQ	INNNNNNTSA	VNGGVSSDGI	IDQNGNGGVS
JAQJVW010000188.1	QQQQQQNANQ	QGQDQYGQQK	DNQQSQFNNQ	INNNNNTSAV	NGGVSSDGII	DQNGNGGVSS
JPEW01000016.1	QQQQQQNANQ	QGQDQYGQQK	DNQQSQFNNQ	INNNNNNTSA	VNGGVSSDGI	IDQNGNGGVS
CM029688.1	QQQQQQNANQ	QGQDQYGQQK	DNQQSQFNNQ	INNNNNNTSA	VNGGVSSDGI	IDQNGNGGVS
CM016755.1	NSNNNNKMQI	NKVKTNMDNK	KIINNHNLII	K*IIILIILV	LLMVV*VVME	**IKMVMEVF
JAMFPW010000016.1	QQQQQQNANQ	QGQDQYGQQK	DNQQSQFNNQ	INNNNNTSAV	NGGVSSDGII	DQNGNGGVSS
VBVA01000076.1	ATTTTKCKST	RSRPIWTTKD	NQQSQFNNQI	NNNNNTSAVN	GGVSSDGIID	QNGNGGVSSG
KE651046.1	QQQQQQNANQ	QGQDQYGQQK	DNQQSQFNNQ	INNNNNTSAV	NGGVSSDGII	DQNGNGGVSS
JAQJVX010000229.1	QQNANQQGQD	QYGQQKDNQQ	SQFNNQINNN	NNTSAVNGGV	SSDGIIDQNG	NGGVSSGQAN
MUZS01002202.1	QQQQQQNANQ	QGQDQYGQQK	DNQQSQFNNQ	INNNNNTSAV	NGGVSSDGII	DQNGNGGVSS
AJJB01000009.1	QQQQQQNANQ	QGQDQYGQQK	DNQQSQFNNQ	INNNNNNTSA	VNGGVSSDGI	IDQNGNGSVS
JAMFPU010000016.1	QQQQQQNANQ	QGQDQYGQQK	DNQQSQFNNQ	INNNNNTSAV	NGGVSSDGII	DQNGNGGVSS
CP025168.1	QQQQQQNANQ	QGQDQYGQQK	DNQQSQFNNQ	INNNNNTSAV	NGGVSSDGII	DQNGNGGVSS
CP025176.1	SNNNNKMQIN	KVKTNMDNKK	IINNHNLIIK	*IIIIIILVL	LMVV*VVME*	*IKMVMEVFL
AJIR01000008.1	QQQQQQNANQ	QGQDQYGQQK	DNQQSQFNNQ	INNNNNTSAV	NGGVSSDGII	DQNGNGGVSS
SCGY01000440.1	QQQQQQNANQ	QGQDQYGQQK	DNQQSQFNNQ	INNNNNTSAV	NGGVSSDGII	DQNGNGGVSS
JAHPZV010000627.1	QQQQQQNANQ	QGQDQYGQQK	DNQQSQFNNQ	INNNNNTSAV	NGGVSSDGII	DQNGNGGVSS
JAMFPO010000675.1						
JADLIL010000679.1	QQQQQQNANQ	QGQDQYGQQK	DNQQSQFNNQ	INNNNNNTSA	VNGGVSSDGI	IDQNGNGGVS
JAHPZU010000362.1	QQQQQQNANQ	QGQDQYGQQK	DNQQSQFNNQ	INNNNNTSAV	NGGVSSDGII	DQNGNGGVSS
JADLIS010000901.1	QQQQQQNANQ	QGQDQYGQQK	DNQQSQFNNQ	INNNNNTSAV	NGGVSSDGII	DQNGNGGVSS
JAMFPP010000106.1	QQQQQQNANQ	QGQDQYGQQK	DNQQSQFNNQ	INNNNNNTSA	VNGGVSSDGI	IDQNGNGGVS
JAQJVO010000188.1	QQQQQQNANQ	QGQDQYGQQK	DNQQSQFNNQ	INNNNNNTSA	VNGGVSSDGI	IDQNGNGGVS
JAMACY010000037.1	QQQQQQNANQ	QGQDQYGQQK	DNQQSQFNNQ	INNNNNTSAV	NGGVSSDGII	DQNGNGGVSS
VBUW01000021.1	QQQQQQNANQ	QGQDQYGQQK	DNQQSQFNNQ	INNNNNNTSA	VNGGVSSDGI	IDQNGNGGVS
JAMFPR010000117.1	QQQQQQNANQ	QGQDQYGQQK	DNQQSQFNNQ	INNNNNTSAV	NGGVSSDGII	DQNGNGGVSS
NETQ01000026.1	QQQQQQNANQ	QGQDQYGQQK	DNQQSQFNNQ	INNNNNNTSA	VNGGVSSDGI	IDQNGNGGVS
AVBA01000072.1	QQQQQQNANQ	QGQDQYGQQK	DNQQSQFNNQ	INNNNNTSAV	NGGVSSDGII	DQNGNGGVSS
KE651163.1	QQQQQQNANQ	QGQDQYGQQK	DNQQSQFNNQ	INNNNNTSAV	NGGVSSDGII	DQNGNGGVSS

KE651030.1	QQQQQQNANQ	QGQDQYGQQK	DNQQSQFNNQ	INNNNNTSAV	NGGVSSDGII	DQNGNGGVSS
JAQJVR010000227.1	QQQQQQNANQ	QGQDQYGQQK	DNQQSQFNNQ	INNNNNTSAV	NGGVSSDGII	DQNGNGGVSS
CH672354.1	QQQQQQNANQ	QGQDQYGQQK	DNQQSQFNNQ	INNNNNTSAV	NGGVSSDGII	DQNGNGGVSS
JADLIT010001479.1	QQQQQQNANQ	QGQDQYGQQK	DNQQSQFNNQ	INNNNNNTSA	VNGGVSSDGI	IDQNGNGGVS
CALMZM010000680.1	QQQQQQNANQ	QGQDQYGQQK	DNQQSQFNNQ	INNNNNTSAV	NGGVSSDGII	DQNGNGGVSS
AJIY01000006.1	QQQQQQNANQ	QGQDQYGQQK	DNQQSQFNNQ	INNNNNTSAV	NGGVSSDGII	DQNGNGGVSS
JADLIU010000982.1	QQQQQQNANQ	QGQDQYGQQK	DNQQSQFNNQ	INNNNNNTSA	VNGGVSSDGI	IDQNGNGGVS
AJJA01000013.1	QQQQQQNANQ	QGQDQYGQQK	DNQQSQFNNQ	INNNNNNTSA	VNGGVSSDGI	IDQNGNGGVS
JAMFPN010000167.1	QQQQQQNANQ	QGQDQYGQQK	DNQQSQFNNQ	INNNNNNTSA	VNGGVSSDGI	IDQNGNGGVS
JABAHU010000017.1	NNNSNNNNKM	QINKVKTNMD	NKKR*STITI	**SNK****Y	*CC*WWCK**	WNNRSKW*WR
JAQJVV010000156.1	QQQQQQNANQ	QGQDQYGQQK	DNQQSQFNNQ	INNNNNNTSA	VNGGVSSDGI	IDQNGNGGVS
JAQJVP010000289.1	QQQQQQNANQ	QGQDQYGQQK	DNQQSQFNNQ	INNNNNTSAV	NGGVSSDGII	DQNGNGGVSS
JAMFPQ010000408.1	QQQQQQNANQ	QGQDQYGQQK	DNQQSQFNNQ	INNNNNNTSA	VNGGVSSDGI	IDQNGNGGVS
JADLII010001503.1						
AJJD01000006.1	QQQQQQNANQ	QGQDQYGQQK	DNQQSQFNNQ	INNNNNTSAV	NGGVSSDGII	DQNGNGGVSS
AJIX01000013.1	QQQQQQNANQ	QGQDQYGQQK	DNQQSQFNNQ	INNNNNNTSA	VNGGVSSDGI	IDQNGNGGVS
JACYCK010000910.1	QQQQQQNANQ	QGQDQYGQQK	DNQQSQFNNQ	INNNNNTSAV	NGGVSSDGII	DQNGNGGVSS
AJIS01000033.1	QQQQQQNANQ	QGQDQYGQQK	DNQQSQFNNQ	INNNNNNTSA	VNGGVSSDGI	IDQNGNGGVS
JAODHA010000012.1	QQQQQQNANQ	QGQDQYGQQK	DNQQSQFNNQ	IK****Y*CC	*WWCK**WNN	RSKW*WRCFF
JPEV01000011.1	QQQQQQNANQ	QGQDQYGQQK	DNQQSQFNNQ	INNNNNNTSA	VNGGVSSDGI	IDQNGNGGVS
JAQJVQ010000193.1	QQQQQQNANQ	QGQDQYGQQK	DNQQSQFNNQ	INNNNNNTSA	VNGGVSSDGI	IDQNGNGSVS
CM016739.1	QQQQQQNANQ	QGQDQYGQQK	DNQQSQFNNQ	INNNNNNTSA	VNGGVSSDGI	IDQNGNGGVS
JTBX01000023.1	QQQQQQNANQ	QGQDQYGQQK	DNQQSQFNNQ	INNNNNTSAV	NGGVSSDGII	DQNGNGGVSS
CM016747.1	QQQQQQNANQ	QGQDQYGQQK	DNQQSQFNNQ	INNNNNNTSA	VNGGVSSDGI	IDQNGNGGVS
AJIT01000009.1	QQQQQQNANQ	QGQDQYGQQK	DNQQSQFNNQ	INNNNNTSAV	NGGVSSDGII	DQNGNGGVSS
JADLIM010001466.1	QQQQQQNANQ	QGQDQYGQQK	DNQQSQFNNQ	INNNNNNTSA	VNGGVSSDGI	IDQNGNGGVS
KE650822.1	QQQQQQNANQ	QGQDQYGQQK	DNQQSQFNNQ	INNNNNTSAV	NGGVSSDGII	DQNGNGGVSS

241

KE651087.1	GQANLPNQSQ	SQSQRQQQQQ	QQEPQQQSEN	QFSGQKQSSS	KSKNGCCVIV	*
AJJE01000008.1	SGQANLPNQS	QSQSQRQQQQ	QQQEPQQQSE	NQFSGQKQSS	SKSKNGCCVI	V
AJJG01000018.1	SGQANLPNQS	QSQRQQQQQQ	QEPQQQSENQ	FSGQKQSSSK	SKNGCCVIV*	A
KE650847.1	GQANLPNQSQ	SQSQRQQQQQ	QQEPQQQSEN	QFSGQKQSSS	KSKNGCCVIV	*
JAQJVS010000214.1	GQANLPNQSQ	SQSQRQQQQQ	QQEPQQQSEN	QFSGQKQSSS	KSKNGCCVIV	*
JAMFPT010000137.1	GQANLPNQSQ	SQSQRQQQQQ	QQEPQQQSEN	QFSGQKQSSS	KSKNGCCVIV	*
JSXQ01000010.1	SGQANLPNQS	QSQSQRQQQQ	QQQEPQQQSE	NQFSGQKQSS	SKSKNGCCVI	V
JAMFPV010000028.1	GQANLPNQSQ	SQSQRQQQQQ	QQEPQQQSEN	QFSGQKQSSS	KSKNGCCVIV	*
AJJC01000012.1	SGQANLPNQS	QSQSQRQQQQ	QQQEPQQQSE	NQFSGQKQSS	SKSKNGCCVI	V
JADLIQ010000221.1	SGQANLPNQS	QSQRQQQQQQ	QEPQQQSENQ	FSGQKQSSSK	SKNGCCVIV*	A
NETO01000041.1	GQANLPNQSQ	SQSQRQQQQQ	QQEPQQQSEN	QFSGQKQSSS	KSKNGCCVIV	*
KE650966.1	GQANLPNQSQ	SQRQQQQQQQ	EPQQQSENQF	SGQKQSSSKS	KNGCCVIV*A	N
KK667224.1	SGQANLPNQS	QSQSQRQQQQ	QQQEPQQQSE	NQFSGQKQSS	SKSKNGCCVI	V
JAQJVU010000255.1	SGQANLPNQS	QSQRQQQQQQ	QEPQQQSENQ	FSGQKQSSSK	SKNGCCVIV*	A
NETM01000037.1	GQANLPNQSQ	SQSQRQQQQQ	QQEPQQQSEN	QFSGQKQSSS	KSKNGCCVIV	*
JADLIO010001687.1	GQANLPNQSQ	SQSQRQQQQQ	QQEPQQQSEN	QFSGQKQSSS	KSKNGCCVIV	*
JAQJVN010000263.1	SGQANLPNQS	QSQSQRQQQQ	QQQEPQQQSE	NQFSGQKQSS	SKSKNGCCVI	V
JSXP01000007.1	GQANLPNQSQ	SQSQRQQQQQ	QQEPQQQSEN	QFSGQKQSSS	KSKNGCCVIV	*
JADLIJ010001632.1	GQANLPNQSQ	SQSQRQQQQQ	QQEPQQQSEN	QFSGQKQSSS	KSKNGCCVIV	*
NETN01000103.1	GQANLPNQSQ	SQSQRQQQQQ	QQEPQQQSEN	QFSGQKQSSS	KSKNGCCVIV	*
JABWAD010000060.1	LVKQIFQINH	NHNHKDNNNS	NNKNHNNNLK	INFLVKNNLA	LNQRMDVVLL	F
AJIZ01000014.1	SGQANLPNQS	QSQRQQQQQQ	QEPQQQSENQ	FSGQKQSSSK	SKNGCCVIV*	A
VBUY01000053.1	SGQANLPNQS	QSQRQQQQQQ	QEPQQQSENQ	FSGQKQSSSK	SKNGCCVIV*	A
KE650918.1	SGQANLPNQS	QSQSQRQQQQ	QQQEPQQQSE	NQFSGQKQSS	SKSKNGCCVI	V
CP032013.1	GQANLPNQSQ	SQRQQQQQQQ	EPQQQSENQF	SGQKQSSSKS	KNGCCVIV*A	N
JADLIN010001249.1	SGQANLPNQS	QSQSQRQQQQ	QQQEPQQQSE	NQFSGQKQSS	SKSKNGCCVI	V
JSXR01000016.1	SGQANLPNQS	QSQSQRQQQQ	QQQEPQQQSE	NQFSGQKQSS	SKSKNGCCVI	V
KE650780.1	SGQANLPNQS	QSQRQQQQQQ	QEPQQQSENQ	FSGQKQSSSK	SKNGCCVIV*	A
AJJF01000006.1	GQANLPNQSQ	SQSQRQQQQQ	QQEPQQQSEN	QFSGQKQSSS	KSKNGCCVIV	*
JACXXM010000041.1	SGQANLPNQS	QSQRQQQQQQ	QEPQQQSENQ	FSGQKQSSSK	SKNGCCVIV*	A
CP017624.1	GQANLPNQSQ	SQSQRQQQQQ	QQEPQQQSEN	QFSGQKQSSS	KSKNGCCVIV	*
JAMFPS010000366.1	SGQANLPNQS	QSQSQRQQQQ	QQQEPQQQSE	NQFSGQKQSS	SKSKNGCCVI	V
JAMADA010000074.1	SGQANLPNQS	QSQSQRQQQQ	QQQEPQQQSE	NQFSGQKQSS	SKSKNGCCVI	V
JADLIR010000106.1	SGQANLPNQS	QSQRQQQQQQ	QEPQQQSENQ	FSGQKQSSSK	SKNGCCVIV*	A
CP025151.1	GQANLPNQSQ	SQSQRQQQQQ	QQEPQQQSEN	QFSGQKQSSS	KSKNGCCVIV	*
CP025159.1	SGQANLPNQS	QSQSQRQQQQ	QQQEPQQQSE	NQFSGQKQSS	SKSKNGCCVI	V
AJIU01000008.1	SGQANLPNQS	QSQSQRQQQQ	QQQEPQQQSE	NQFSGQKQSS	SKSKNGCCVI	V
AJIW01000013.1	SGQANLPNQS	QSQSQRQQQQ	QQQEPQQQSE	NQFSGQKQSS	SKSKNGCCVI	V
JAMACZ010000790.1	SGQANLPNQS	QSQRQQQQQQ	QEPQQQSENQ	FSGQKQSSSK	SKNGCCVIV*	A
KE650911.1	SGQANLPNQS	QSQRQQQQQQ	QEPQQQSENQ	FSGQKQSSSK	SKNGCCVIV*	A
JAKTXK010000203.1	LPNQSQSQSQ	RQQQQQQQEP	QQQSENQFSG	QKQSSSKSKN	GCCVIV*AN*	K
JAQJVY010000211.1	SGQANLPNQS	QSQSQRQQQQ	QQQEPQQQSE	NQFSGQKQSS	SKSKNGCCVI	V
JADLIK010000138.1	SGQANLPNQS	QSQRQQQQQQ	QEPQQQSENQ	FSGQKQSSSK	SKNGCCVIV*	A
JAHPZT010000270.1	GQANLPNQSQ	SQSQRQQQQQ	QQEPQQQSEN	QFSGQKQSSS	KSKNGCCVIV	*
AJIQ01000011.1	SGQANLPNQS	QSQSQRQQQQ	QQQEPQQQSE	NQFSGQKQSS	SKSKNGCCVI	V
JADLIP010000819.1	SGQANLPNQS	QSQSQRQQQQ	QQQEPQQQSE	NQFSGQKQSS	SKSKNGCCVI	V
JAQJVT010000197.1	GQANLPNQSQ	SQSQRQQQQQ	QQEPQQQSEN	QFSGQKQSSS	KSKNGCCVIV	*
AJIV01000014.1	GQANLPNQSQ	SQSQRQQQQQ	QQEPQQQSEN	QFSGQKQSSS	KSKNGCCVIV	*
JAODHB010000006.1	SGQANLPNQS	QSQSQRQQQQ	QQQEPQQQSE	NQFSGQKQSS	SKSKNGCCVI	V
JAAGWN010000001.1	SGQANLPNQS	QSQSQRQQQQ	QQQEPQQQSE	NQFSGQKQSS	SKSKNGCCVI	V
JAQJVW010000188.1	GQANLPNQSQ	SQSQRQQQQQ	QQEPQQQSEN	QFSGQKQSSS	KSKNGCCVIV	*
JPEW01000016.1	SGQANLPNQS	QSQRQQQQQQ	QEPQQQSENQ	FSGQKQSSSK	SKNGCCVIV*	A
CM029688.1	SGQANLPNQS	QSQSQRQQQQ	QQQEPQQQSE	NQFSGQKQSS	SKSKNGCCVI	V
CM016755.1	LLVKQIFQIN	HNHKDNNNSN	NKNHNNNLKI	NFWSKTI*L*	IKEWMLCYCL	S
JAMFPW010000016.1	GQANLPNQSQ	SQSQRQQQQQ	QQEPQQQSEN	QFSGQKQSSS	KSKNGCCVIV	*
VBVA01000076.1	QANLPNQSQS	QSQRQQQQQQ	QEPQQQSENQ	FSGQKQSSSK	SKNGCCVIV*	A
KE651046.1	GQANLPNQSQ	SQSQRQQQQQ	QQEPQQQSEN	QFSGQKQSSS	KSKNGCCVIV	*
JAQJVX010000229.1	LPNTITITKT	TTTATTRTTT	TI*KSIFWSK	TI*L*IKEWM	LCYCLS*LKS	S
MUZS01002202.1	GQANLPNQSQ	SQSQRQQQQQ	QQEPQQQSEN	QFSGQKQSSS	KSKNGCCVIV	*
AJJB01000009.1	SGQANLPNQS	QSQRQQQQQQ	QEPQQQSENQ	FSGQKQSSSK	SKNGCCVIV*	A
JAMFPU010000016.1	GQANLPNQSQ	SQSQRQQQQQ	QQEPQQQSEN	QFSGQKQSSS	KSKNGCCVIV	*
CP025168.1	GQANLPNQSQ	SQSQRQQQQQ	QQEPQQQSEN	QFSGQKQSSS	KSKNGCCVIV	*
CP025176.1	LVKQIFQINH	NHNHKDNNNS	NNKNHNNNLK	INFLVKNNLA	LNQRMDVVLL	F
AJIR01000008.1	GQANLPNQSQ	SQSQRQQQQQ	QQEPQQQSEN	QFSGQKQSSS	KSKNGCCVIV	*
SCGY01000440.1	GQANLPNQSQ	SQSQRQQQQQ	QQEPQQQSEN	QFSGQKQSSS	KSKNGCCVIV	*
JAHPZV010000627.1	GQANLPNQSQ	SQSQRQQQQQ	QQEPQQQSEN	QFSGQKQSSS	KSKNGCCVIV	*
JAMFPO010000675.1						
JADLIL010000679.1	SGQANLPNQS	QSQSQRQQQQ	QQQEPQQQSE	NQFSGQKQSS	SKSKNGCCVI	V
JAHPZU010000362.1	GQANLPNQSQ	SQSTFSG*CQ	ITTNQQQH*L	VTTTTVTIMK	QTYEIFHFFT	V
JADLIS010000901.1	GQANLPNQSQ	SQSQRQQQQQ	QQEPQQQSEN	QFSGQKQSSS	KSKNGCCVIV	*
JAMFPP010000106.1	SGQANLPNQS	QSQSQRQQQQ	QQQEPQQQSE	NQFSGQKQSS	SKSKNGCCVI	V
JAQJVO010000188.1	SGQANLPNQS	QSQSQRQQQQ	QQQEPQQQSE	NQFSGQKQSS	SKSKNGCCVI	V
JAMACY010000037.1	GQANLPNQLQ	SQSQRQQQQQ	QQEPQQQSEN	QFSGQKQSSS	KSKNGCCVIV	*
VBUW01000021.1	SGQANLPNQS	QSQSQRQQQQ	QQQEPQQQSE	NQFSGQKQSS	SKSKNGCCVI	V
JAMFPR010000117.1	GQANLPNQSQ	SQSQRQQQQQ	QQEPQQQSEN	QFSGQKQSSS	KSKNGCCVIV	*
NETQ01000026.1	SGQANLPNQS	QSQSQRQQQQ	QQQEPQQQSE	NQFSGQKQSS	SKSKNGCCVI	V
AVBA01000072.1	GQANLPNQSQ	SQSQRQQQQQ	QQEPQQQSEN	QFSGQKQSSS	KSKNGCCVIV	*
KE651163.1	GQANLPNQSQ	SQSQRQQQQQ	QQEPQQQSEN	QFSGQKQSSS	KSKNGCCVIV	*

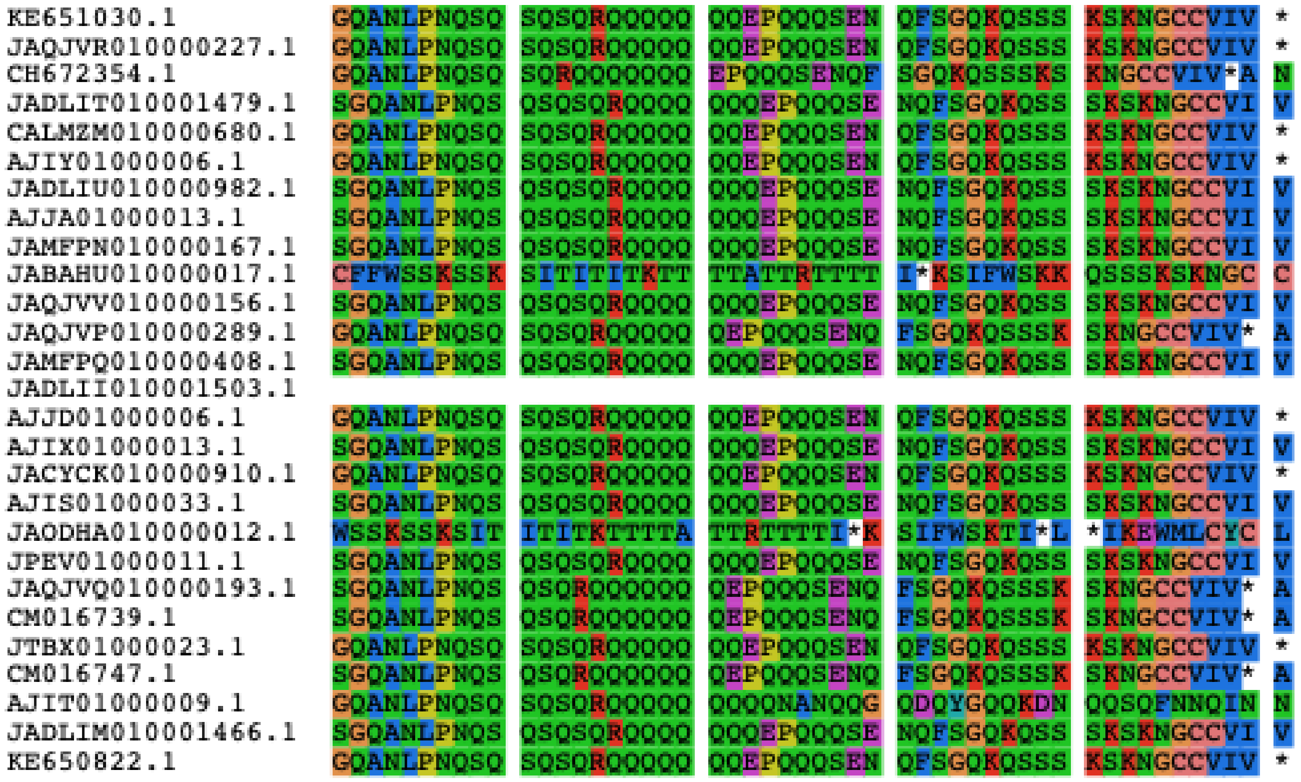
KE651030.1 GQANLPNQSQ SQSQRQQQQQ QQEPQQQSEN QFSGQKQSSS KSKNGCCVIV *
JAQJVR010000227.1 GQANLPNQSQ SQSQRQQQQQ QQEPQQQSEN QFSGQKQSSS KSKNGCCVIV *
CH672354.1 GQANLPNQSQ SQRQQQQQQQ EPQQQSENQF SGQKQSSSKS KNGCCVIV*A N
JADLIT010001479.1 SGQANLPNQS QSQSQRQQQQ QQQEPQQQSE NQFSGQKQSS SKSKNGCCVI V
CALMZM010000680.1 GQANLPNQSQ SQSQRQQQQQ QQEPQQQSEN QFSGQKQSSS KSKNGCCVIV *
AJIY01000006.1 GQANLPNQSQ SQSQRQQQQQ QQEPQQQSEN QFSGQKQSSS KSKNGCCVIV *
JADLIU010000982.1 SGQANLPNQS QSQSQRQQQQ QQQEPQQQSE NQFSGQKQSS SKSKNGCCVI V
AJJA01000013.1 SGQANLPNQS QSQSQRQQQQ QQQEPQQQSE NQFSGQKQSS SKSKNGCCVI V
JAMFPN010000167.1 SGQANLPNQS QSQSQRQQQQ QQQEPQQQSE NQFSGQKQSS SKSKNGCCVI V
JABAHU010000017.1 CFFWSSKSSK SITITITKTT TTATTRTTTT I*KSIFWSKK QSSSKSKNGC C
JAQJVV010000156.1 SGQANLPNQS QSQSQRQQQQ QQQEPQQQSE NQFSGQKQSS SKSKNGCCVI V
JAQJVP010000289.1 GQANLPNQSQ SQSQRQQQQQ QEPQQQSENQ FSGQKQSSSK SKNGCCVIV* A
JAMFPQ010000408.1 SGQANLPNQS QSQSQRQQQQ QQQEPQQQSE NQFSGQKQSS SKSKNGCCVI V
JADLII010001503.1
AJJD01000006.1 GQANLPNQSQ SQSQRQQQQQ QQEPQQQSEN QFSGQKQSSS KSKNGCCVIV *
AJIX01000013.1 SGQANLPNQS QSQSQRQQQQ QQQEPQQQSE NQFSGQKQSS SKSKNGCCVI V
JACYCK010000910.1 GQANLPNQSQ SQSQRQQQQQ QQEPQQQSEN QFSGQKQSSS KSKNGCCVIV *
AJIS01000033.1 SGQANLPNQS QSQSQRQQQQ QQQEPQQQSE NQFSGQKQSS SKSKNGCCVI V
JAODHA010000012.1 WSSKSSKSIT ITITKTTTTA TTRTTTTI*K SIFWSKTI*L *IKEWMLCYC L
JPEV01000011.1 SGQANLPNQS QSQSQRQQQQ QQQEPQQQSE NQFSGQKQSS SKSKNGCCVI V
JAQJVQ010000193.1 SGQANLPNQS QSQRQQQQQQ QEPQQQSENQ FSGQKQSSSK SKNGCCVIV* A
CM016739.1 SGQANLPNQS QSQRQQQQQQ QEPQQQSENQ FSGQKQSSSK SKNGCCVIV* A
JTBX01000023.1 GQANLPNQSQ SQSQRQQQQQ QQEPQQQSEN QFSGQKQSSS KSKNGCCVIV *
CM016747.1 SGQANLPNQS QSQRQQQQQQ QEPQQQSENQ FSGQKQSSSK SKNGCCVIV* A
AJIT01000009.1 GQANLPNQSQ SQSQRQQQQQ QQQQNANQQG QDQYGQQKDN QQSQFNNQIN N
JADLIM010001466.1 SGQANLPNQS QSQSQRQQQQ QQQEPQQQSE NQFSGQKQSS SKSKNGCCVI V
KE650822.1 GQANLPNQSQ SQSQRQQQQQ QQEPQQQSEN QFSGQKQSSS KSKNGCCVIV *
